# Genome-Wide Copy Number Variations Inferred from SNP Genotyping Arrays Using a Large White and Minzhu Intercross Population

**DOI:** 10.1371/journal.pone.0074879

**Published:** 2013-10-01

**Authors:** Ligang Wang, Xin Liu, Longchao Zhang, Hua Yan, Weizhen Luo, Jing Liang, Duxue Cheng, Shaokang Chen, Xiaojun Ma, Xin Song, Kebin Zhao, Lixian Wang

**Affiliations:** 1 Key Laboratory of Farm Animal Genetic Resources and Germplasm Innovation of Ministry of Agriculture, Institute of Animal Science, Chinese Academy of Agricultural Sciences, Beijing, China; 2 College of Animal Science and Technology, State Key Laboratory of Agrobiotechnology, China Agricultural University, Beijing, China; 3 College of Veterinary Medicine, Sichuan Agricultural University, Ya'an, Sichuan, China; National Institute of Environmental Health Sciences, United States of America

## Abstract

Copy number variations (CNVs) are one of the main contributors to genetic diversity in animals and are broadly distributed in the genomes of swine. Investigating the performance and evolutionary impacts of pig CNVs requires comprehensive knowledge of their structure and function within and between breeds. In the current study, 4 different programs (i.e., GADA, PennCNV, QuantiSNP, and cnvPartition) were used to analyze Porcine SNP60 genotyping data of 585 pigs from one Large White × Minzhu intercross population to detect copy number variant regions (CNVRs). Overlapping CNVRs recalled by at least 2 programs were used to construct a powerful and comprehensive CNVR map, which contained249 CNVRs (i.e., 70 gains, 43 losses, and 136 gains/losses) and covered 26.22% of the regions in the swine genome. Ten CNVRs, representing different predicted statuses, were selected for validation *via* quantitative real-time PCR (QPCR); 9/10 CNVRs (i.e., 90%) were validated. When being traced back to the F0 generation, 58 events were identified in only Minzhu F0 parents and 2 events were identified in only Large White F0 parents. A series of CNVR function analyses were performed. Some of the CNVRs functions were predicted, and several interesting CNVRs for meat quality traits and hematological parameters were obtained. A comprehensive and lower false rate genome-wide CNV map was constructed for Large White and Minzhu pig genomes in this study. Our results may provide an important basis for determining the relationship between CNVRs and important qualitative and quantitative traits. In addition, it can help to further understand genetic processes in pigs.

## Introduction

The pig (*Sus scrofa*) is not only an economically important livestock worldwide but also an ideal animal model for human disease research because its genome is similar in size and organization. Copy number variations (CNVs) are global genetic structural variations in human and animal genomes, and they defined as a segment of large DNA [kilobases (Kb) to megabases (Mb) in length] presenting with copy-number differences through the comparison of 2 or more genomes [1]–[6]. CNVs occupy a significant portion of all pig genomic variations, CNVs can directly impact gene expression by changing gene dosage or indirectly affecting gene expression by disrupting the regulation of gene expression [1], [7]–[11]. Many studies have shown that CNVs play important roles in normal phenotypic variability and disease susceptibility [1], [12]–[18]. They are considered promising markers for identifying economic- and disease-related traits in domestic animals [19].

At present, several technologies containing comparative genomic hybridization (CGH) arrays, clone and PCR-product arrays, oligonucleotide arrays, and SNP genotyping arrays can be used for detecting genome-wide CNVs [20]. By using CGH techniques, Fadista et al. [21] found 37 CNV regions (CNVRs) among 12 Duroc boars. Using Porcine SNP60 BeadChips, Ramayo-Caldas et al. [22] and Wang et al. [19] have identified 49 CNVRs and 382 CNVRs, respectively, in the pig genome. Validation experiments have been conducted using real-time quantitative PCR (QPCR) in each of these 3 studies. Four out of 10 (40%), 5/7 (71.43%), and 12/18 (66.67%) of the analyzed CNVRs were validated. The abundance of CNVRs detected in pigs is far less than that detected in other species (∼12%, 4%, and 4.6% in human [2], dog [23] and cattle [6] genome sequences, respectively.).

In the present study, weconstructed a Large White × Minzhu intercross population and measured various traits [24], [25]. Each individual was genotyped using an Illumina PorcineSNP60 Beadchip. The goal of this study was to construct a more accurate and comprehensive map of CNVs in the pig genome in order to determine the relationship between CNVRs and some important qualitative and quantitative traits and provide useful information for understanding the genetic processes of pigs. In this study, 4 different programs (i.e., GADA, PennCNV, QuantiSNP, and cnvPartition) [26]–[29]were used to analyze Porcine SNP60 genotyping data of 619 pigs from one Large White × Minzhu intercross population to detect CNVRs. A number of integrative analyse were also conducted.

## Results

### CNV detection

In this study, a total of 585 samples were processed using the Illumina Porcine SNP60 BeadChip and passed through a series of quality control measures for CNV detection. The initial number of CNVs identified by GADA, PennCNV, QuantiSNP, and cnvPartition was 4678, 1550, 3485, and 316, respectively. CNVRs that overlapped on more than one contig and contained gaps due to the high error rate of this preliminary assembly were discarded. By aggregating overlapping CNVs, a total of 660, 505, 966, and 60 CNVRs were identified by the 4 programs (Table S1 in File S1). The average lengths of these CNVRs were 1.88 Mb, 0.21 Mb, 1.05 Mb, and 2.57 Mb. For all the results of these 4 algorithms, the average length of the regions, which contained both duplication and deletion CNVs, were much larger than the total average lengths (i.e., 5.00 Mb, 0.41 Mb, 3.73 Mb, and 3.80 Mb).

**Figure 1:**
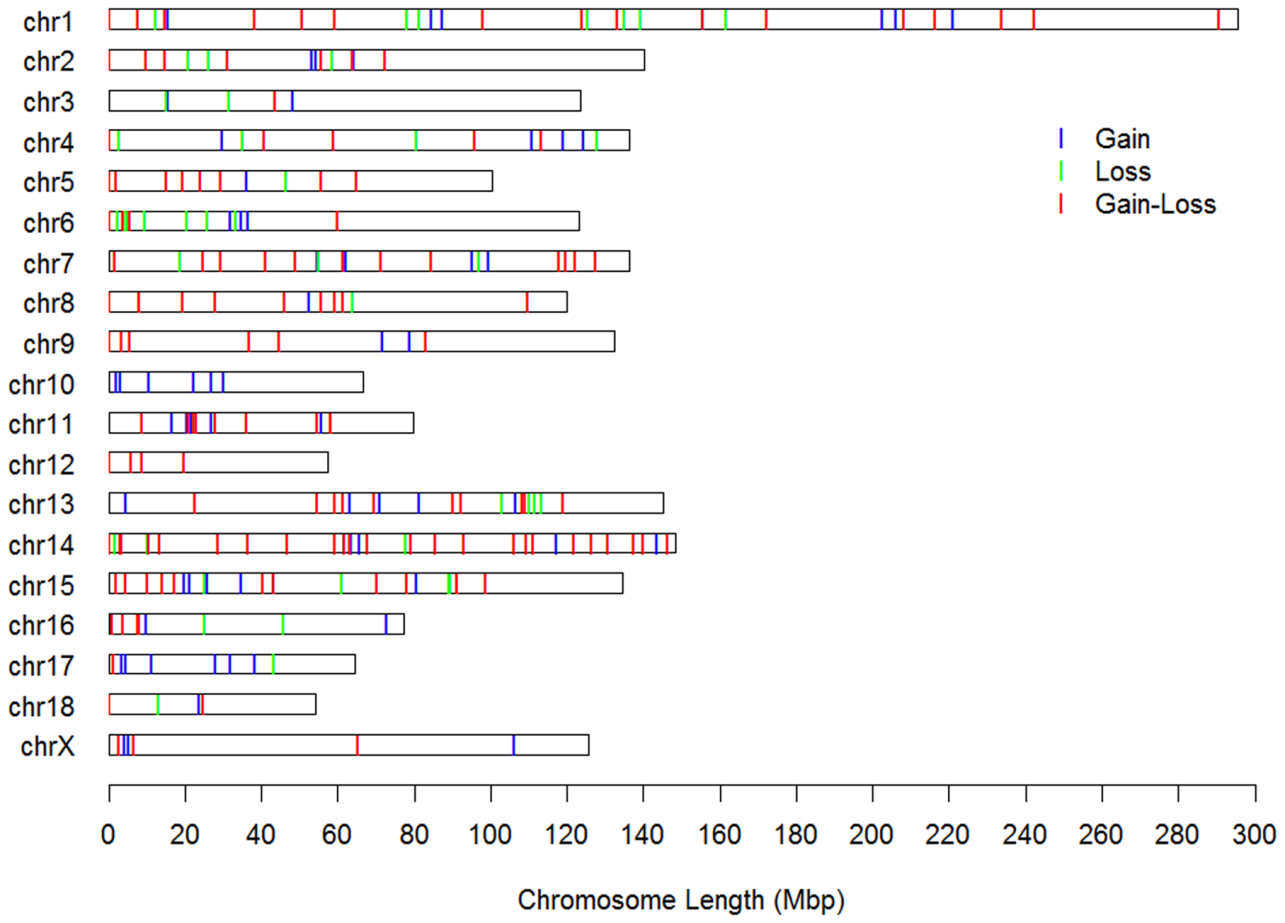
Distribution of CNVRs in pig autosomal and X chromosomes. Red, green and blue lines represent Gain, loss and either gain or loss predicted status. Y-axis values are chromosome names, and X-axis values are chromosome position in Mb, which are proportional to real size of swine genome sequence assembly (9.2) (http://www.ensembl.org/Sus_scrofa/Info/Index).

CNVRs containing overlapping CNVs recalled by at least 2 programs were selected for further analyses. Finally, a total of 249 CNVRs (i.e., 70 gains, 43 losses, and 136 gains/losses) covering a 560.30-Mb (26.22%) region of the swine genome (Table S2 in File S1) were identified. These CNVRs ranged from 29.20 kb to 27.29 Mb (with a median size of 845.98 kb). Overlaps between the CNVRs detected by each program (GADA, PennCNV, QuantiSNP, and cnvPartition) and the 249 overlapped CNVRs are 341/660(51.67%), 301/505(59.60%), 522/996(52.41%), and 39/60 (65.00%). When traced back to the F0 generation, 233 and 84 CNVRs could be commonly detected in Minzhu F0 parents and Large White F0 parents (Table 1). Most of the CNVRs (88.33%) detected in the F0 parents could overlap with those detected in the F2 populations. Fifty-eight events were identified only in Minzhu F0 parents, and 2 events were identified only in Large White F0 parents (Table 1, Figure S1 and Figure S2 in File S2). The locations and characteristics of all CNVRs on the autosomal and × chromosomes are shown in Figure 1 and the 60 unique CNVRs detected in F0 parents are shown in Table 2.

**Table 1:** Sample sizes and the CNVR numbers detected in F0 and F2 generation.

Generation	Breed	Sample size	CNVRs number	Unique CNVRs
F0	Minzhu pig	19	233	58
F0	Large- White	5	84	2
F2	Crossbreed	506	249	–

Unique CNVR means CNVR only detected in this breed.

**Table 2:** Unique CNVRs in F0 Minzhu pig and F0 Large-White.

CNVR NO.	Chr	Start	End	Length (Kb)	Status	Breed
1	1	27137	4021371	3994.234	Gain-Loss	Minzhu pig
4	1	14913515	16205785	1292.27	Gain-Loss	Minzhu pig
6	1	38187823	39892124	1704.301	Gain-Loss	Minzhu pig
8	1	59146777	60116291	969.514	Gain-Loss	Minzhu pig
9	1	78080106	78512135	432.029	Loss	Large-White
13	1	97861336	102022988	4161.652	Gain-Loss	Large-White
14	1	123741378	125430919	1689.541	Gain-Loss	Minzhu pig
23	1	202360098	202460519	100.421	Gain	Minzhu pig
28	1	233648692	235129449	1480.757	Gain-Loss	Minzhu pig
30	1	290536560	295554054	5017.494	Gain-Loss	Minzhu pig
31	2	42783	6186192	6143.409	Gain-Loss	Minzhu pig
32	2	9612578	14884403	5271.825	Gain-Loss	Minzhu pig
39	2	55639944	57533047	1893.103	Gain-Loss	Minzhu pig
51	4	29558904	29848274	289.37	Gain	Minzhu pig
53	4	40547805	53260169	12712.364	Gain-Loss	Minzhu pig
55	4	80557625	80683923	126.298	Loss	Minzhu pig
62	5	33971	2576759	2542.788	Gain-Loss	Minzhu pig
64	5	14977039	23972011	8994.972	Gain-Loss	Minzhu pig
67	5	29271805	32046817	2775.012	Gain-Loss	Minzhu pig
71	5	64939963	73159278	8219.315	Gain-Loss	Minzhu pig
72	6	26646	2373898	2347.252	Gain-Loss	Minzhu pig
83	6	34507867	35720036	1212.169	Gain	Minzhu pig
84	6	36433467	37058702	625.235	Gain	Minzhu pig
89	7	29484113	29946052	461.939	Gain-Loss	Minzhu pig
93	7	54923282	59344416	4421.134	Loss	Minzhu pig
97	7	71399968	85475026	14075.058	Gain-Loss	Minzhu pig
99	7	95002189	95880768	878.579	Gain	Minzhu pig
104	7	122242402	123874154	1631.752	Gain-Loss	Minzhu pig
107	8	7879866	8054169	174.303	Gain-Loss	Minzhu pig
110	8	27976730	29061313	1084.583	Gain-Loss	Minzhu pig
111	8	46183156	47937663	1754.507	Gain-Loss	Minzhu pig
112	8	52363564	53392239	1028.675	Gain	Minzhu pig
118	9	27950	3729624	3701.674	Gain-Loss	Minzhu pig
120	9	5559852	6597228	1037.376	Gain-Loss	Minzhu pig
122	9	44705850	45388279	682.429	Gain-Loss	Minzhu pig
125	9	82946155	92571240	9625.085	Gain-Loss	Minzhu pig
137	11	22392667	36576409	14183.742	Gain-Loss	Minzhu pig
148	12	8839980	20037607	11197.627	Gain-Loss	Minzhu pig
149	12	19662620	37002457	17339.837	Gain-Loss	Minzhu pig
160	13	92117925	119407655	27289.73	Gain-Loss	Minzhu pig
169	14	45833	7887586	7841.753	Gain-Loss	Minzhu pig
175	14	13242399	21656861	8414.462	Gain-Loss	Minzhu pig
176	14	28551814	36527320	7975.506	Gain-Loss	Minzhu pig
178	14	46604684	56259408	9654.724	Gain-Loss	Minzhu pig
185	14	67634813	77654554	10019.741	Gain-Loss	Minzhu pig
192	14	110998360	113178611	2180.251	Gain-Loss	Minzhu pig
197	14	137251412	148678088	11426.676	Gain-Loss	Minzhu pig
209	15	25744213	27098633	1354.42	Gain	Minzhu pig
211	15	40187988	43203855	3015.867	Gain-Loss	Minzhu pig
223	16	823535	1293412	469.877	Gain-Loss	Minzhu pig
225	16	3590605	4172931	582.326	Gain-Loss	Minzhu pig
230	16	45805292	50607024	4801.732	Loss	Minzhu pig
231	16	72854046	74717034	1862.988	Gain	Minzhu pig
232	17	1347911	2345614	997.703	Gain-Loss	Minzhu pig
233	17	3231266	3510331	279.065	Gain	Minzhu pig
235	17	11096541	11214207	117.666	Gain	Minzhu pig
246	X	5054064	18192210	13138.146	Gain	Minzhu pig
247	X	6734423	24477135	17742.712	Gain-Loss	Minzhu pig
248	X	65177195	71802709	6625.514	Gain-Loss	Minzhu pig
249	X	106109244	117864445	11755.201	Gain	Minzhu pig

Unique CNVR means CNVR only detected in this breed.

Positions are retrieved from the swine genome sequence assembly (9.2) (http://www.ensembl.org/Sus_scrofa/Info/Index).

### CNVR analysis

By using the BioMart data management system, 142 CNVRs (57.03%) containing 1857 annotated genes from the Ensembl Genes 64 Database (Table S3 in File S1) were detected. These genes were primarily identified as protein-coding (1533, 82.55%) biotypes, and the remainder were miRNA (62), pseudogenes (60), retrotransposed (4), snoRNA (65), snRNA (94), rRNA (16), and miscRNA (23) biotypes. Compared tothe genes reported in the Database of Genomic Variants (DGV), a total of 703 genes (37.86%) belonging to 2166 human genomic variant regions were detected (Table S4 in File S1). Compared to previous research, 19/49 CNVRs (38.78%) in Ramayo's report, 14/37 CNVRs (37.83%) in Fadista's report, and 168/382 CNVRs (43.98%) in Wang's report were identical to or overlapped with our results [19], [21], [22].

Using the online Gene Functional Classification and Annotation Tool in the database for Annotation, Visualization and Integrated Discovery (DAVID, http://david.abcc.ncifcrf.gov) [30], 7 Benjiamini correction, statistically significant Gene Ontology (GO) [31] terms (Table S5 in File S1) and 4 Benjiamini correction statistically significant Kyoto Encyclopedia of Genes and Genomes (KEGG) pathways (Table S6 in File S1)were identified [32]. The detected genes in significant GO terms were mainly involved in alternative splicing, splice variants, phosphoproteins, cytoplasm RNA-binding, translation regulation, and membrane-enclosed lumen significant GO terms. The detected genes in KEGG pathways were mainly involved in axon guidance endocytosis homologous recombination and the ErbB signaling pathway. Furthermore, 116 CNVRs (46.6%) overlapped with 1345 QTLs (Table S7 in File S1) in the pig QTLdb database [33]. These overlapped QTLs were mainly related to meat quality traits (59.33%) and the remainders were related to exterior, health, meat quality, productive and reproduction traits.

In our previous studies, genome-wide association studies (GWAS) with meat quality, production and health traits were performed using the same population [24], [25]. Combining analyses found that a total number of 27, 22, 4, 3, 10, 3, and 2 genome-wide significant SNPs associated with intramuscular fat (IMF), marbling, moisture, color score, lean meat in ham, lean meat weight, and mean corpuscular volume (MCV), respectively (Table S8–S14 in File S1), were located in 6 CNVRs identified in this study. Moreover, most of these CNVRs (i.e., 4/6) only appeared in Minzhu pigs and not in the Large White pig F0 generation.

### Validation by quantitative PCR

**Figure 2:**
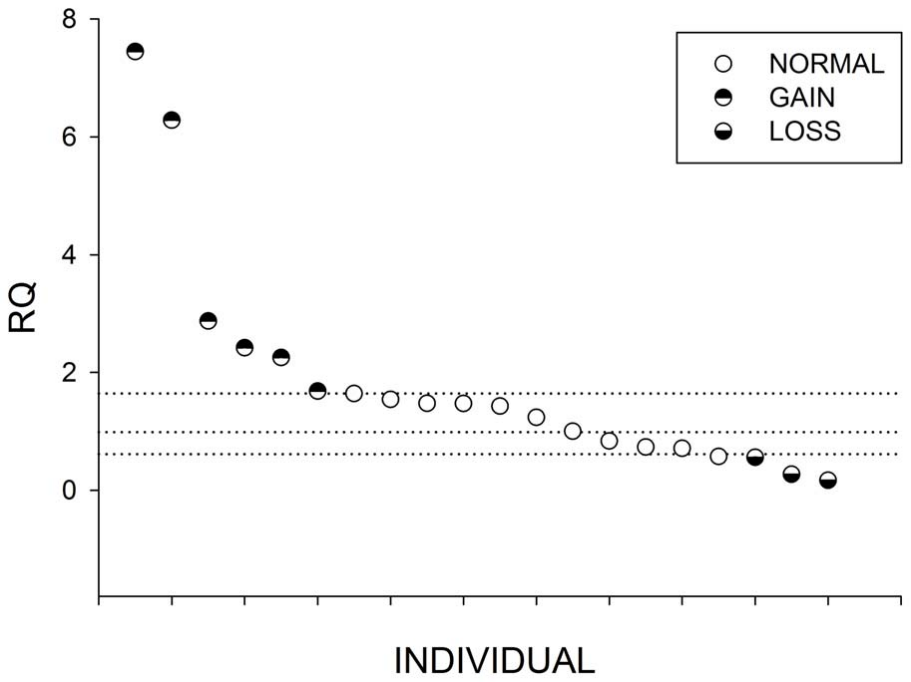
Relative quantification (RQ) value by Quantitative PCR (QPCR) for CNVR64. Twenty animals with Relative quantification (RQ) value are showed in this figure. Each dot represents the relative copy number in comparison to the reference individual. Y-axis shows the RQ obtained by QPCR. Samples with RQ about 1 denote normal individuals (two copy), samples with RQ below 0.59 (ln^1.5^) denote copy number loss individuals, and samples with RQ about 1.59 (ln^3^) or more denote copy number gain individuals (≧three copy).

**Figure 3:**
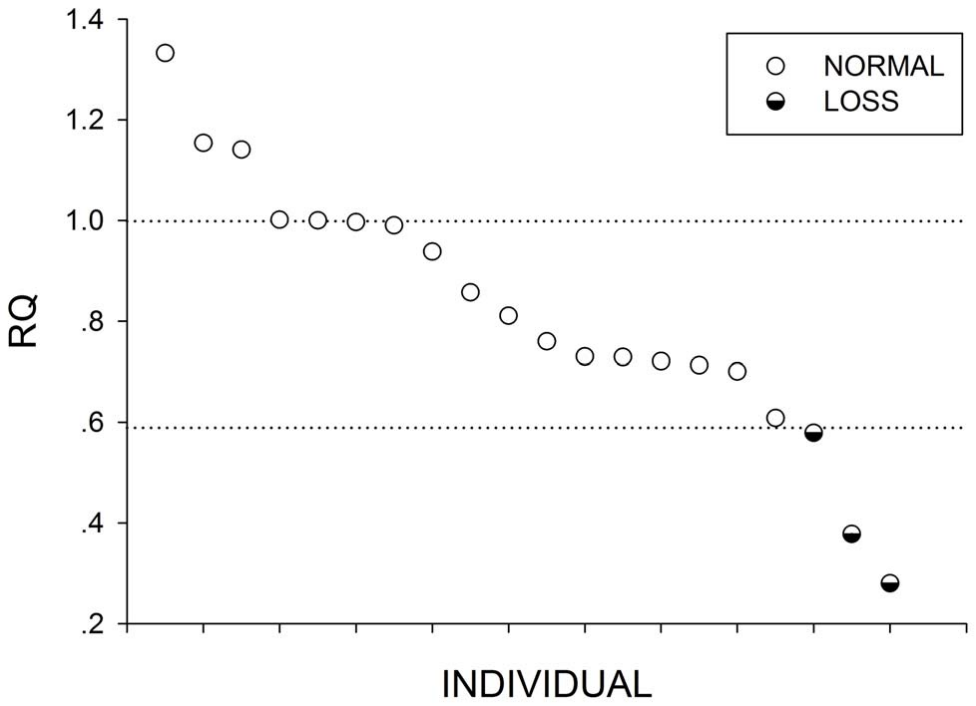
Relative quantification (RQ) value by Quantitative PCR (QPCR) for CNVR79. Twenty animals with Relative quantification (RQ) value are showed in this figure. Each dot represents the relative copy number in comparison to the reference individual. Y-axis shows the RQ obtained by QPCR. Samples with RQ about 1 denote normal individuals (two copy), samples with RQ below 0.59 (ln^1.5^) denote copy number loss individuals.

Ten genomic regions (i.e., CNVR3, 16, 42, 64, 67, 79, 86, 167, 184, 243) were selected to be validated by quantitative real-time PCR (QPCR) from the 249 CNVRs detected using the 4 programs (Table 3). These 10 CNVRs, ranged from 82.99 to 8994.97 kb, were selected sub-randomly, and represented different predicted statuses of copy numbers (i.e., gain, loss, and gain/loss). As shown in Table 3, nine of these CNVRs (90%) could be detected by QPCR (i.e., CNVR3, 16, 42, 64, 67, 79, 86, 167, and 243). In addition, as shown in Figures 2, 3, and S3–S10 in File S2, the copy number in the CNVRs varied among individuals. Among these 9 CNVRs, although CNVR3 could be detected loss status in program prediction results, it can be detected both gain and loss status in QPCR validation.

**Table 3:** Results of QPCR Validation.

CNVR No.	Chr.	Start	End	validated	Validated Type	Detected Type	Genes
CNVR3	1	12381202	13318271	YES	Gain-loss	Loss	*TIAM2*
CNVR16	1	133318245	135237989	YES	Gain-loss	Gain-loss	*ELL3*
CNVR42	2	64108598	64269234	YES	Gain	Gain	*C1orf150*
CNVR64	5	14977039	23972011	YES	Gain-loss	Gain-loss	*F1SGK0_PIG*
CNVR67	5	29271805	32046817	YES	Gain-loss	Gain-loss	*HMGA2*
CNVR79	6	20427689	21016514	YES	Loss	Loss	*CES1*
CNVR86	7	1545143	2308802	YES	Gain-loss	Gain-loss	*ECI2*
CNVR167	13	113339066	113574977	YES	Loss	Loss	*Retrotransposed*
CNVR184	14	65608238	65691232	NO	–	Gain	–
CNVR243	18	24738187	25927458	YES	Gain-loss	Gain-loss	–

*TIAM2* is T-cell lymphoma invasion and metastasis 2; *ELL3* is elongation factor RNA polymerase II-like 3; *C1orf150* is chromosome 1 open reading frame 150; *F1SGK0_PIG* is an Uncharacterized gene; *HMGA2* is high mobility group AT-hook 2; *CES1* is liver carboxylesterase; *ECI2* is enoyl-CoA delta isomerase 2.

## Discussion

In the current study, 4 different programs (i.e., GADA, PennCNV, QuantiSNP, and CnvPartition) were used to detect CNVRs. These 4 programs calculated CNVs by using different algorithms as follows: (1) GADA uses a Sparse Bayesian Learning model (SBL), (2) PennCNV use Hidden Markov Models (HMM), (3) QuantiSNP uses Hidden Markov Models with Bayes Factor and (4) cnvPartition uses Gaussian Distribution Models.

Each of these programs had their own weaknesses (i.e., GADA is weak in the accuracy of Illumina, PennCNV has no way of ranking events due to likelihood, QuantiSNP has limited support for further event analysis and cnvPartition may miss events) [29]. Therefore, following the recommendations for increasing the frequency and decreasing the rate of false positives from Winchester et al. [29], the CNVRs, which were detected by at least 2 algorithms, were selected for use in the present research. Furthermore, in this study, a 3-generation resource population was produced by intercrossing Large White boars and Minzhu pig sows from 2007 to 2011. The population size in the current study was larger (i.e., 619 individuals) than previous studies on pigs and may decrease false CNVs. As a result, better QPCR validation was obtained than that reported by Fasista et al., Ramayo-Caldas et al. and Wang et al. (50.00%, 71.43%, and 66.67%, respectively) [19], [21], [22].

The special genetic background also cannot be ignored. CNVs in animals have been reported to have breed-specific characteristics [5], [19]. Similar to previous reports, after analyzing CNV delivery in the F0 generation, 58 and 2 CNVRs were detected only in Minzhu and Large White pigs, respectively. The use of a Minzhu × Large White intercross population and 4 CNVs detection programs in this research may have minimized overlapping rates (from 38.78% to 43.98%). Another reason for the lower overlapping rates could be the different platforms we used. The SNP genotyping and CGH arrays, for instance, were different in calling technology, resolution differences, and genome coverage [19]. When the PennCNV programs were used both in this study and in the study of Wang et al. [19], 207 CNVRs (54.19%) overlapped.The low overlapping rates were also encountered in the studies of pigs and other mammals [5], [6], [19], [34], [35].

CNVRs identified in unrelated pig samples from different genetic backgrounds are important criteria in retaining CNVRs for downstream analysis. As the breed-specific CNVRs may contribute to breed differences, we first analyzed the traits and CNVR differences in the F0 parents. The Minzhu pig is a breed indigenous to northeast China. Average environmental temperatures of 4°C/year are experienced in this region and, in response, the Minzhu pig breed has good stress resistance and has developed excellent characteristics of fat deposition, [i.e., back fat thickness of 5.1 cm and 5% IMF in the longissimus muscle (LM) at 240 d of age]. Compared to the Minzhu pig, the Large-White pig has a higher rate of lean meat and faster growing rates. Under the supposition that some of the CNVRs only detected in Minzhu pigs and Large-White pigs affected these traits, we selected these CNVRs for further analyse. In order to minimize the number of these CNVRs, GO, KEGG, QTL, and comparative genomic analyse were conducted simultaneously. Oure analyses identified some interesting CNVRs.

One of these CNVRs was CNVR149 (Chr. 12, 19662620: 37002457), which only appeared in the F0 Minzhu pig generation (gain status) and contained 70 protein-coding, 4 miRNA, 3 pseudogenes, 8 snoRNA, 10 snRNA, and 2 rRNA genes (Table S15 in File S1). Most of the genome-wide significant SNPs associated with IMF (27/38, 71.05%) and marbling (22/37, 59.46%) were located in these domains. There were also 4 genome-wide significant SNPs associated with color score and 22 QTLs [33], [36], [37] related to meat quality located in these domains. Furthermore, while not using the same population, María et al. (2011) also found genome-wide significant SNPs associated with IMF in this domain [38]. Moreover, among the genes contained in this domain, spermatogenesis associated 20 (*SPATA20*) is one of the putative transcripts expressed in significantly different levels during bovine intramuscular adipocyte differentiation profiled [39]. We inferred that this CNVR is positively associated with meat quality by changing the gene dosage or disrupting the regulation of gene expression. In addition, the copy number polymorphism (CNP) genotyping using next-generation sequencing [40] in this region is in the pipeline.

Another interesting CNVR is CNVR31 (Chr. 2, 42783∶6186192). This CNVR, also, only appeared in the F0 Minzhu pig generation and contained 62 protein-coding, 3 miRNA, 1 pseudogene, and 1 snRNA gene (Table S16 in File S1). Most of the genome-wide significant SNPs associated with lean meat in ham (10/23, 43.48%) and lean meat weight (3/14, 21.43%) were located in these domains. In this region, 4 members of the fibroblast growth factor (FGF) family (*FGF3*, *4, 19*) genes were identified. The FGF family is involved in numerous cellular processes including growth, angiogenesis, and development [41]–[44]. Transgenic mice overexpressing human *FGF19* have an increased metabolic rate and decreased adiposity [45], [46]. There were also 5 QTLs [33], [47], [48] related to traits of production in this region. Therefore, we inferred that this CNVR may have effects on lean meat.

Other CNVRs, such as CNVR109 (Chr. 8, 19534783:19709874) and CNVR110 (Chr. 8, 27976730:29061313), were also interesting. There was 1 genome-wide significant SNPs associated with MCV located in these two regions respectively. There were also 4 healthy related QTLs [49] located in these regions, which indicated the potential immune-related function of these CNVRs.

## Conclusions

By using the Porcine SNP60 Genotyping BeadChip and an F2 pig resource population, we identified 249 CNVRs and generated a powerful and comprehensive CNVR map of the pig genome. Nine out of 10 CNVRs were validated by QPCR, indicating that our detection was highly efficient. Fifty-eight potential Minzhu pig breed-specific and 2 potential Large White pig breed-specific CNVRs were also identified. In addition, we obtained several interesting CNVRs with the integration of previously gathered QTL and SNP data for the pig families, or other populations. Our work provides an important basis for understanding pig genetic processes and obtained several interesting CNVRs for meat quality traits and hematological parameters.

## Materials and Methods

### Ethics Statement

All animal procedures were performed according to the guidelines developed by the China Council on Animal Care, and all protocols were approved by the Animal Care and Use Committee of Beijing, China. The approval ID or permit numbers were *SYXK (Beijing) 2008–007* and *SYXK (Beijing) 2008–008*.

### Animals

In this study, an F2 resource population was produced by intercrossing Large White boars and Minzhu pig sows during the period of 2007 to 2011. Five Large White boars were mated with 19 Minzhu pig sows. The resulting F1 generation, comprising 9 sires and 46 dams were mated (avoiding full-sib mating) to produce 576 F2 animals in 3 parities. Most sows were mated to the same boar for all 3 litters to provide large, full-sib populations. Male pigs of the F2 generation were castrated. All F2 animals were reared under identical feeding conditions at the pig research station of the Institute of Animal Science at the Chinese Academy of Agricultural Sciences.

### Genotyping and quality control

Genomic DNA was extracted from ear tissue according to standard protocols. Genotyping was performed using the PorcineSNP60 Genotyping BeadChip technology (Illumina), which contained 62,163 SNPs across the whole genome. BEADSTUDIO software (Illumina) was used to call the genotypes for all samples. Data were quality controlled for sample call rate, SNP call rate, minor allele frequency (MAF) and deviations from Hardy Weinberg Equilibrium (HWE). SNPs were excluded according to the following criteria: (1) call rate<90%, (2) MAF<3%, and (3) significant divergence from HWE with *P*-values lower than 10^−6^. At the second step of the iterative procedure, individuals were excluded with call rates<90%.

The final data set that passed the quality control procedure and was used in the analysis contained 48,238 SNPs and 506 F2 individuals. The distribution of SNPs after quality control and the average distance between adjacent SNPs on each chromosome are shown in Table S1 in File S1.

### CNV detection

Beadstudio software (Illumina) was used to export the total signal intensity (Log R Ratio, LRR) and allelic intensity ratio (B Allele Freq, BAF) to employ GADA, PennCNV, and QuantiSNP. The version of the SNPs physical position on chromosomes derived from the Ensembl website was 9.2. The cnvPartition analysis Plug-in of Beadstudio Software (Illumina) was used for CNV detection. The minimum probe count was set to 3 and all other parameters used the default settings.

We used R statistical programming language version 2.9.2 [50] and the multiple array analysis mode of GADA to perform CNV detection, with 0.8 for sparseness hyperparameter (α) of the sparse Bayesian learning (SBL) model and 4 for the critical value of backward elimination (BE). The minimum number of SNPs at each segment was 3. Except for the LRR and BAF, to launch QuantiSNP, we also needed a genderfile. We generated the genderfile following the manufacturer's instructions and used the command line to run the QuantiSNP software with the default parameters. Then, the knock-out CNVs appeared in only one individual and the ones that contained less than 3 SNPs.

The PennCNV program also needs more information, such as the population frequency of the B allele (pfb) of SNPs, the pedigree information, and the gcmodel file. The pfb file we used was calculated based on the BAF for each marker. The pedigree information used was compiled following the manufacturer's instructions. The pig gcmodel file used was generated by calculating the GC content of the 1-Mb genomic regions surrounding each marker. The CNV detection by PennCNV was performed using the default parameters. Additionally, after calling, CNVs presented in only one individual were also knocked out.

In order to balance false positives and power, we knocked out the CNVs, which were called only in one algorithm and presented in only one individual. Then, we aggregated overlapping CNVs to be copy number variable regions (CNVRs). The F0 generation of Minzhu pigs and Large-white pigs were calculated separately.

### CNVR analysis

Genes within the detected CNVRs were retrieved from the Ensembl Genes 64 Database using the BioMart (http://www.biomart.org) software. Gene Ontology (GO) and Kyoto Encyclopedia of Genes and Genomes (KEGG) pathway analyse were carried out from the database for Annotation, Visualization and Integrated Discovery (DAVID, http://david.abcc.ncifcrf.gov). A little program named overlapping was written by Visual Basic to retrieve the QTLs within the CNVRs from the pig QTLdb (http://www.animalgenome.org/cgi-bin/QTLdb/SS/index). Some of the GWAS data we used in this paper was retrieved from the paper of LUO et al. [24]; the others were calculated using the method reported in the paper of LUO et al. [24], [25]. All gene positions were transformed to fit the style of Ensembl Genes 64.

### Quantitative real time PCR

The Quantitative real time PCR amplification was performed using the default conditions in 384-well optical PCR plates using an ABI 7900HT instrument (Applied Biosystems, Inc., Foster City, CA). TaqMan primer/probe sets were designed to query random CNVs using the Primer 3 web tool (http://frodo.wi.mit.edu/primer3/). For each assay, 15 ng of genomic DNA was assayed in quadruplicate in 15-µL reactions containing a 1× final concentration of the TaqMan Universal Master Mix (ABI part number 4304437), and 150 nM each for the primers and probes. The SDS 2.4 software was used to analyze the results. The glucagon gene (GCG) [51] was used as the single copy control. Copy number was calculated by the 2−ΔΔCT method [52], [53], where ΔCT is the cycle threshold (CT) of the target region minus the CT of the control region. In addition, 2−ΔΔCT compares the ΔCT value of samples with the CNV to the calibrator without the CNV. The PCR cycle was as follows: 2 min at 50°C, 10 min at 95°C, and 40 cycles of 15 sec at 95°C and 1 min at 60°C. A list of the 11 probes used in the study is shown in Table S17 in File S1.

## Supporting Information

File S1 **Additional tables:** Table S1: CNVRs identified by GADA, PennCNV, QuantiSNP and CnvPartition. Table S2: Description of the 249 CNVRs detected in the swine genome. Table S3: Genes in all the CNVRs retrieved from Ensembl Genes 64 Database. Table S4: Genes searched in DGV. Table S5: Significant GO terms of the Genes. Table S6: Significant KEGG pathways of the Genes. Table S7: List of the overlapping QTLs. Table S8: Genome-wide significant SNPs associated with intramuscular fat (IMF). Table S9: Genome-wide significant SNPs associated with marbling. Table S10: Genome-wide significant SNPs associated with moisture. Table S11: Genome-wide significant SNPs associated with color score. Table S12: Genome-wide significant SNPs associated with lean meat in ham. Table S13: Genome-wide significant SNPs associated with lean meat weight. Table S14: Genome-wide significant SNPs associated mean corpuscular volume (MCV). Table S15: Genes in CNVR149. Table S16: Genes in CNVR31. Table S17: Primers and probes used in QPCR validation.
(DOCX)

File S2 **Additional Figures:** Figure S1: Distribution of CNVRs in Minzhu pig F0 generation. Figure S2: Distribution of CNVRs in Large White pig F0 generation. Figure S3: Relative quantification (RQ) value by Quantitative PCR (QPCR) for CNVR3. Figure S4: Relative quantification (RQ) value by Quantitative PCR (QPCR) for CNVR16. Figure S5: Relative quantification (RQ) value by Quantitative PCR (QPCR) for CNVR42. Figure S6: Relative quantification (RQ) value by Quantitative PCR (QPCR) for CNVR67. Figure S7: Relative quantification (RQ) value by Quantitative PCR (QPCR) for CNVR86. Figure S8: Relative quantification (RQ) value by Quantitative PCR (QPCR) for CNVR167. Figure S9: Relative quantification (RQ) value by Quantitative PCR (QPCR) for CNVR184. Figure S10: Relative quantification (RQ) value by Quantitative PCR (QPCR) for CNVR243.
(DOCX)
