# Supplementary material for: Genome-Wide Copy Number Variations Inferred from SNP Genotyping Arrays Using a Large White and Minzhu Intercross Population
Source: PLoS One. 2013 Oct 1;8(10):e74879. doi: 10.1371/journal.pone.0074879 (PMC3787955; doi:10.1371/journal.pone.0074879)
Supplement: File S1 — Additional tables: Table S1: CNVRs identified by GADA, PennCNV, QuantiSNP and CnvPartition. Table S2: Description of the 249 CNVRs detected in the swine genome. Table S3: Genes in all the CNVRs retrieved from Ensembl Genes 64 Database. Table S4: Genes searched in DGV. Table S5: Significant GO terms of the Genes. Table S6: Significant KEGG pathways of the Genes. Table S7: List of the overlapping QTLs. Table S8: Genome-wide significant SNPs associated with intramuscular fat (IMF). Table S9: Genome-wide significant SNPs associated with marbling. Table S10: Genome-wide significant SNPs associated with moisture. Table S11: Genome-wide significant SNPs associated with color score. Table S12: Genome-wide significant SNPs associated with lean meat in ham. Table S13: Genome-wide significant SNPs associated with lean meat weight. Table S14: Genome-wide significant SNPs associated mean corpuscular volume (MCV). Table S15: Genes in CNVR149. Table S16: Genes in CNVR31. Table S17: Primers and probes used in QPCR validation. (DOCX) [file pone.0074879.s001.docx]

**Table S1:** CNVRs identified by GADA, PennCNV, QuantiSNP and CnvPartition

|  | Number | | | | Cover Range (Mb) | | | |
| --- | --- | --- | --- | --- | --- | --- | --- | --- |
|  | Total | Gain | Loss | Both | Total | Gain | Loss | Both |
| GADA | 660 | 226 | 330 | 104 | 1240.79 | 202.86 | 518.12 | 519.80 |
| PennCNV | 505 | 311 | 151 | 43 | 108.40 | 57.32 | 33.55 | 17.53 |
| QuantiSNP | 966 | 460 | 339 | 167 | 1015.05 | 300.67 | 91.63 | 622.74 |
| CnvPartition | 60 | 16 | 40 | 4 | 154.37 | 74.78 | 64.39 | 15.20 |

**Table S2:** Description of the 249 CNVRs detected in the swine genome

| CNVR No. | Chr | Start | End | Length(Kb) | Type |
| --- | --- | --- | --- | --- | --- |
| 1 | 1 | 27137 | 4021371 | 3994.234 | Gain-Loss |
| 2 | 1 | 7495071 | 7841348 | 346.277 | Gain-Loss |
| 3 | 1 | 12381202 | 13318271 | 937.069 | Loss |
| 4 | 1 | 14913515 | 16205785 | 1292.27 | Gain-Loss |
| 5 | 1 | 15371031 | 16386303 | 1015.272 | Gain |
| 6 | 1 | 38187823 | 39892124 | 1704.301 | Gain-Loss |
| 7 | 1 | 50546882 | 53921857 | 3374.975 | Gain-Loss |
| 8 | 1 | 59146777 | 60116291 | 969.514 | Gain-Loss |
| 9 | 1 | 78080106 | 78512135 | 432.029 | Loss |
| 10 | 1 | 81368054 | 81557600 | 189.546 | Loss |
| 11 | 1 | 84545694 | 84736433 | 190.739 | Gain |
| 12 | 1 | 87104293 | 87133489 | 29.196 | Gain |
| 13 | 1 | 97861336 | 102022988 | 4161.652 | Gain-Loss |
| 14 | 1 | 123741378 | 125430919 | 1689.541 | Gain-Loss |
| 15 | 1 | 125261701 | 125430919 | 169.218 | Loss |
| 16 | 1 | 133189092 | 135237989 | 2048.897 | Gain-Loss |
| 17 | 1 | 134861405 | 135180400 | 318.995 | Loss |
| 18 | 1 | 139269219 | 139413719 | 144.5 | Loss |
| 19 | 1 | 155346222 | 156292470 | 946.248 | Gain-Loss |
| 20 | 1 | 155382583 | 156292470 | 909.887 | Gain |
| 21 | 1 | 161574623 | 162662337 | 1087.714 | Loss |
| 22 | 1 | 172242183 | 173322208 | 1080.025 | Gain-Loss |
| 23 | 1 | 202360098 | 202460519 | 100.421 | Gain |
| 24 | 1 | 205847031 | 207582470 | 1735.439 | Gain |
| 25 | 1 | 208228776 | 208395667 | 166.891 | Gain-Loss |
| 26 | 1 | 216277027 | 217902817 | 1625.79 | Gain-Loss |
| 27 | 1 | 220749696 | 221072973 | 323.277 | Gain |
| 28 | 1 | 233648692 | 235129449 | 1480.757 | Gain-Loss |
| 29 | 1 | 242082040 | 244585427 | 2503.387 | Gain-Loss |
| 30 | 1 | 290536560 | 295554054 | 5017.494 | Gain-Loss |
| 31 | 2 | 42783 | 6186192 | 6143.409 | Gain-Loss |
| 32 | 2 | 9612578 | 14884403 | 5271.825 | Gain-Loss |
| 33 | 2 | 14848378 | 14964157 | 115.779 | Gain-Loss |
| 34 | 2 | 20945054 | 21549021 | 603.967 | Loss |
| 35 | 2 | 26150053 | 26900214 | 750.161 | Loss |
| 36 | 2 | 30997755 | 31417643 | 419.888 | Gain-Loss |
| 37 | 2 | 53139398 | 53267309 | 127.911 | Gain |
| 38 | 2 | 54160085 | 55006068 | 845.983 | Gain |
| 39 | 2 | 55639944 | 57533047 | 1893.103 | Gain-Loss |
| 40 | 2 | 58539767 | 58893490 | 353.723 | Loss |
| 41 | 2 | 63696971 | 66246314 | 2549.343 | Gain-Loss |
| 42 | 2 | 64108598 | 64269234 | 160.636 | Gain |
| 43 | 2 | 72343520 | 73718746 | 1375.226 | Gain-Loss |
| 44 | 3 | 15167349 | 15239537 | 72.188 | Loss |
| 45 | 3 | 15474321 | 15556575 | 82.254 | Gain |
| 46 | 3 | 31383127 | 31580596 | 197.469 | Loss |
| 47 | 3 | 43680902 | 58891371 | 15210.469 | Gain-Loss |
| 48 | 3 | 48199203 | 49047619 | 848.416 | Gain |
| 49 | 4 | 79881 | 2769382 | 2689.501 | Gain-Loss |
| 50 | 4 | 2806586 | 4665175 | 1858.589 | Loss |
| 51 | 4 | 29558904 | 29848274 | 289.37 | Gain |
| 52 | 4 | 34958980 | 35374343 | 415.363 | Loss |
| 53 | 4 | 40547805 | 53260169 | 12712.364 | Gain-Loss |
| 54 | 4 | 58827461 | 59808656 | 981.195 | Gain-Loss |
| 55 | 4 | 80557625 | 80683923 | 126.298 | Loss |
| 56 | 4 | 95631307 | 97672295 | 2040.988 | Gain-Loss |
| 57 | 4 | 110797722 | 110878636 | 80.914 | Gain |
| 58 | 4 | 113312122 | 114024841 | 712.719 | Gain-Loss |
| 59 | 4 | 118746428 | 119812514 | 1066.086 | Gain |
| 60 | 4 | 124221572 | 124392474 | 170.902 | Gain |
| 61 | 4 | 127826157 | 131718122 | 3891.965 | Loss |
| 62 | 5 | 33971 | 2576759 | 2542.788 | Gain-Loss |
| 63 | 5 | 1967579 | 8183268 | 6215.689 | Gain-Loss |
| 64 | 5 | 14977039 | 23972011 | 8994.972 | Gain-Loss |
| 65 | 5 | 19408876 | 20163230 | 754.354 | Gain-Loss |
| 66 | 5 | 23927817 | 23988021 | 60.204 | Gain-Loss |
| 67 | 5 | 29271805 | 32046817 | 2775.012 | Gain-Loss |
| 68 | 5 | 36112409 | 36473502 | 361.093 | Gain |
| 69 | 5 | 46410229 | 46475049 | 64.82 | Loss |
| 70 | 5 | 55592002 | 56265934 | 673.932 | Gain-Loss |
| 71 | 5 | 64939963 | 73159278 | 8219.315 | Gain-Loss |
| 72 | 6 | 26646 | 2373898 | 2347.252 | Gain-Loss |
| 73 | 6 | 2240368 | 2373898 | 133.53 | Loss |
| 74 | 6 | 3613848 | 5713364 | 2099.516 | Gain-Loss |
| 75 | 6 | 4245266 | 4309223 | 63.957 | Loss |
| 76 | 6 | 4912365 | 4972426 | 60.061 | Loss |
| 77 | 6 | 5678485 | 5735912 | 57.427 | Gain-Loss |
| 78 | 6 | 9535524 | 9765104 | 229.58 | Loss |
| 79 | 6 | 20427689 | 21016514 | 588.825 | Loss |
| 80 | 6 | 25675237 | 25706324 | 31.087 | Loss |
| 81 | 6 | 31948568 | 32312059 | 363.491 | Gain |
| 82 | 6 | 33253968 | 33669051 | 415.083 | Loss |
| 83 | 6 | 34507867 | 35720036 | 1212.169 | Gain |
| 84 | 6 | 36433467 | 37058702 | 625.235 | Gain |
| 85 | 6 | 59752433 | 64456285 | 4703.852 | Gain-Loss |
| 86 | 7 | 1545143 | 2308802 | 763.659 | Gain-Loss |
| 87 | 7 | 18601625 | 18680179 | 78.554 | Loss |
| 88 | 7 | 24798851 | 25857148 | 1058.297 | Gain-Loss |
| 89 | 7 | 29484113 | 29946052 | 461.939 | Gain-Loss |
| 90 | 7 | 41192060 | 42929573 | 1737.513 | Gain-Loss |
| 91 | 7 | 48940404 | 53953630 | 5013.226 | Gain-Loss |
| 92 | 7 | 54483290 | 54787295 | 304.005 | Gain |
| 93 | 7 | 54923282 | 59344416 | 4421.134 | Loss |
| 94 | 7 | 61296573 | 62080435 | 783.862 | Gain-Loss |
| 95 | 7 | 61912839 | 62126647 | 213.808 | Gain |
| 96 | 7 | 62126647 | 65028537 | 2901.89 | Gain |
| 97 | 7 | 71399968 | 85475026 | 14075.058 | Gain-Loss |
| 98 | 7 | 84533940 | 89103828 | 4569.888 | Gain-Loss |
| 99 | 7 | 95002189 | 95880768 | 878.579 | Gain |
| 100 | 7 | 96847982 | 96987818 | 139.836 | Loss |
| 101 | 7 | 99496691 | 101342180 | 1845.489 | Gain |
| 102 | 7 | 117783601 | 119915830 | 2132.229 | Gain-Loss |
| 103 | 7 | 119774712 | 120258833 | 484.121 | Gain-Loss |
| 104 | 7 | 122242402 | 123874154 | 1631.752 | Gain-Loss |
| 105 | 7 | 127352695 | 133307931 | 5955.236 | Gain-Loss |
| 106 | 8 | 17610 | 2380350 | 2362.74 | Gain-Loss |
| 107 | 8 | 7879866 | 8054169 | 174.303 | Gain-Loss |
| 108 | 8 | 8054169 | 13302756 | 5248.587 | Gain-Loss |
| 109 | 8 | 19534783 | 19709874 | 175.091 | Gain-Loss |
| 110 | 8 | 27976730 | 29061313 | 1084.583 | Gain-Loss |
| 111 | 8 | 46183156 | 47937663 | 1754.507 | Gain-Loss |
| 112 | 8 | 52363564 | 53392239 | 1028.675 | Gain |
| 113 | 8 | 55541534 | 55723870 | 182.336 | Gain-Loss |
| 114 | 8 | 59102882 | 59775286 | 672.404 | Gain-Loss |
| 115 | 8 | 61412977 | 61629673 | 216.696 | Gain-Loss |
| 116 | 8 | 63738936 | 64265372 | 526.436 | Loss |
| 117 | 8 | 109693339 | 117177230 | 7483.891 | Gain-Loss |
| 118 | 9 | 27950 | 3729624 | 3701.674 | Gain-Loss |
| 119 | 9 | 3427753 | 5500684 | 2072.931 | Gain-Loss |
| 120 | 9 | 5559852 | 6597228 | 1037.376 | Gain-Loss |
| 121 | 9 | 36868460 | 38215047 | 1346.587 | Gain-Loss |
| 122 | 9 | 44705850 | 45388279 | 682.429 | Gain-Loss |
| 123 | 9 | 71508571 | 72136711 | 628.14 | Gain |
| 124 | 9 | 78839781 | 83686965 | 4847.184 | Gain |
| 125 | 9 | 82946155 | 92571240 | 9625.085 | Gain-Loss |
| 126 | 10 | 2077873 | 2135005 | 57.132 | Gain |
| 127 | 10 | 3080469 | 3219342 | 138.873 | Gain |
| 128 | 10 | 10482926 | 11241624 | 758.698 | Gain |
| 129 | 10 | 22380285 | 22564699 | 184.414 | Gain |
| 130 | 10 | 26713246 | 26915492 | 202.246 | Gain |
| 131 | 10 | 30135698 | 30193417 | 57.719 | Gain |
| 132 | 11 | 8646131 | 9325340 | 679.209 | Gain-Loss |
| 133 | 11 | 16453500 | 16686741 | 233.241 | Gain |
| 134 | 11 | 20485485 | 20592188 | 106.703 | Gain |
| 135 | 11 | 20813752 | 21557759 | 744.007 | Gain-Loss |
| 136 | 11 | 21557759 | 21849418 | 291.659 | Gain |
| 137 | 11 | 22392667 | 36576409 | 14183.742 | Gain-Loss |
| 138 | 11 | 22960537 | 25079466 | 2118.929 | Gain-Loss |
| 139 | 11 | 26905253 | 27102932 | 197.679 | Gain |
| 140 | 11 | 27888543 | 30650779 | 2762.236 | Gain-Loss |
| 141 | 11 | 36204388 | 53062866 | 16858.478 | Gain-Loss |
| 142 | 11 | 54661077 | 54837097 | 176.02 | Gain-Loss |
| 143 | 11 | 55669471 | 55841427 | 171.956 | Gain |
| 144 | 11 | 57959672 | 58171775 | 212.103 | Gain-Loss |
| 145 | 11 | 58171775 | 58334625 | 162.85 | Gain-Loss |
| 146 | 12 | 17787 | 9768790 | 9751.003 | Gain-Loss |
| 147 | 12 | 5994579 | 13664145 | 7669.566 | Gain-Loss |
| 148 | 12 | 8839980 | 20037607 | 11197.627 | Gain-Loss |
| 149 | 12 | 19662620 | 37002457 | 17339.837 | Gain-Loss |
| 150 | 13 | 4569357 | 4697917 | 128.56 | Gain |
| 151 | 13 | 22576371 | 25283315 | 2706.944 | Gain-Loss |
| 152 | 13 | 54640025 | 56463750 | 1823.725 | Gain-Loss |
| 153 | 13 | 59200476 | 60852912 | 1652.436 | Gain-Loss |
| 154 | 13 | 61203462 | 81519755 | 20316.293 | Gain-Loss |
| 155 | 13 | 63080978 | 63353046 | 272.068 | Gain |
| 156 | 13 | 69569108 | 70025008 | 455.9 | Gain-Loss |
| 157 | 13 | 70918623 | 71205247 | 286.624 | Gain |
| 158 | 13 | 81179972 | 81519755 | 339.783 | Gain |
| 159 | 13 | 90162285 | 90842628 | 680.343 | Gain-Loss |
| 160 | 13 | 92117925 | 119407655 | 27289.73 | Gain-Loss |
| 161 | 13 | 102911825 | 103704825 | 793 | Loss |
| 162 | 13 | 106491729 | 107292844 | 801.115 | Gain |
| 163 | 13 | 108252023 | 109808296 | 1556.273 | Gain-Loss |
| 164 | 13 | 108940017 | 109155136 | 215.119 | Gain-Loss |
| 165 | 13 | 109911384 | 110440261 | 528.877 | Loss |
| 166 | 13 | 111599538 | 111927175 | 327.637 | Loss |
| 167 | 13 | 113339066 | 113574977 | 235.911 | Loss |
| 168 | 13 | 119068108 | 121753674 | 2685.566 | Gain-Loss |
| 169 | 14 | 45833 | 7887586 | 7841.753 | Gain-Loss |
| 170 | 14 | 1555931 | 1732661 | 176.73 | Loss |
| 171 | 14 | 3128681 | 3538704 | 410.023 | Gain-Loss |
| 172 | 14 | 3538704 | 3635926 | 97.222 | Gain-Loss |
| 173 | 14 | 9976351 | 10458229 | 481.878 | Loss |
| 174 | 14 | 10641829 | 12600024 | 1958.195 | Gain-Loss |
| 175 | 14 | 13242399 | 21656861 | 8414.462 | Gain-Loss |
| 176 | 14 | 28551814 | 36527320 | 7975.506 | Gain-Loss |
| 177 | 14 | 36553547 | 46794621 | 10241.074 | Gain-Loss |
| 178 | 14 | 46604684 | 56259408 | 9654.724 | Gain-Loss |
| 179 | 14 | 59331065 | 59487577 | 156.512 | Gain-Loss |
| 180 | 14 | 61635660 | 61788686 | 153.026 | Gain-Loss |
| 181 | 14 | 61788686 | 63163312 | 1374.626 | Gain |
| 182 | 14 | 63078700 | 63464256 | 385.556 | Gain-Loss |
| 183 | 14 | 63609066 | 63760594 | 151.528 | Gain |
| 184 | 14 | 65608238 | 65691232 | 82.994 | Gain |
| 185 | 14 | 67634813 | 77654554 | 10019.741 | Gain-Loss |
| 186 | 14 | 77693712 | 78947214 | 1253.502 | Loss |
| 187 | 14 | 78965047 | 85401739 | 6436.692 | Gain-Loss |
| 188 | 14 | 85427385 | 91231413 | 5804.028 | Gain-Loss |
| 189 | 14 | 92850014 | 104407170 | 11557.156 | Gain-Loss |
| 190 | 14 | 106139896 | 107085009 | 945.113 | Gain-Loss |
| 191 | 14 | 109258870 | 109528732 | 269.862 | Gain-Loss |
| 192 | 14 | 110998360 | 113178611 | 2180.251 | Gain-Loss |
| 193 | 14 | 116979953 | 120492714 | 3512.761 | Gain |
| 194 | 14 | 121585633 | 126165714 | 4580.081 | Gain-Loss |
| 195 | 14 | 126463335 | 127585364 | 1122.029 | Gain-Loss |
| 196 | 14 | 130747700 | 132810552 | 2062.852 | Gain-Loss |
| 197 | 14 | 137251412 | 148678088 | 11426.676 | Gain-Loss |
| 198 | 14 | 139821449 | 141452615 | 1631.166 | Gain-Loss |
| 199 | 14 | 143260794 | 143422907 | 162.113 | Gain |
| 200 | 14 | 146269092 | 148678088 | 2408.996 | Gain-Loss |
| 201 | 15 | 1788025 | 1967493 | 179.468 | Gain-Loss |
| 202 | 15 | 4382615 | 4625432 | 242.817 | Gain-Loss |
| 203 | 15 | 10297095 | 11112985 | 815.89 | Gain-Loss |
| 204 | 15 | 14002523 | 15789301 | 1786.778 | Gain-Loss |
| 205 | 15 | 17239062 | 17944045 | 704.983 | Gain-Loss |
| 206 | 15 | 19829025 | 19950540 | 121.515 | Gain |
| 207 | 15 | 21005071 | 21180279 | 175.208 | Gain |
| 208 | 15 | 24975412 | 25168180 | 192.768 | Loss |
| 209 | 15 | 25744213 | 27098633 | 1354.42 | Gain |
| 210 | 15 | 34782319 | 34860095 | 77.776 | Gain |
| 211 | 15 | 40187988 | 43203855 | 3015.867 | Gain-Loss |
| 212 | 15 | 43203855 | 44562391 | 1358.536 | Gain |
| 213 | 15 | 43203855 | 47741689 | 4537.834 | Gain-Loss |
| 214 | 15 | 60863969 | 61762207 | 898.238 | Loss |
| 215 | 15 | 70130527 | 73067537 | 2937.01 | Gain-Loss |
| 216 | 15 | 77861484 | 79210937 | 1349.453 | Gain-Loss |
| 217 | 15 | 80667803 | 81348577 | 680.774 | Gain |
| 218 | 15 | 88904825 | 89397638 | 492.813 | Loss |
| 219 | 15 | 89340225 | 89397638 | 57.413 | Loss |
| 220 | 15 | 91021780 | 91102992 | 81.212 | Gain-Loss |
| 221 | 15 | 91102992 | 95934160 | 4831.168 | Gain-Loss |
| 222 | 15 | 98582871 | 106982553 | 8399.682 | Gain-Loss |
| 223 | 16 | 823535 | 1293412 | 469.877 | Gain-Loss |
| 224 | 16 | 1002908 | 1394666 | 391.758 | Gain-Loss |
| 225 | 16 | 3590605 | 4172931 | 582.326 | Gain-Loss |
| 226 | 16 | 7694530 | 8098889 | 404.359 | Gain-Loss |
| 227 | 16 | 8080655 | 8582416 | 501.761 | Gain-Loss |
| 228 | 16 | 9756163 | 9966221 | 210.058 | Gain |
| 229 | 16 | 25069154 | 25481257 | 412.103 | Loss |
| 230 | 16 | 45805292 | 50607024 | 4801.732 | Loss |
| 231 | 16 | 72854046 | 74717034 | 1862.988 | Gain |
| 232 | 17 | 1347911 | 2345614 | 997.703 | Gain-Loss |
| 233 | 17 | 3231266 | 3510331 | 279.065 | Gain |
| 234 | 17 | 4403261 | 5562602 | 1159.341 | Gain |
| 235 | 17 | 11096541 | 11214207 | 117.666 | Gain |
| 236 | 17 | 27742508 | 28649954 | 907.446 | Gain |
| 237 | 17 | 31763157 | 31981492 | 218.335 | Gain |
| 238 | 17 | 38180287 | 38431142 | 250.855 | Gain |
| 239 | 17 | 43310924 | 43624088 | 313.164 | Loss |
| 240 | 18 | 58753 | 3835215 | 3776.462 | Gain-Loss |
| 241 | 18 | 13064157 | 13895350 | 831.193 | Loss |
| 242 | 18 | 23460140 | 23573113 | 112.973 | Gain |
| 243 | 18 | 24738187 | 25927458 | 1189.271 | Gain-Loss |
| 244 | x | 2792657 | 3723909 | 931.252 | Gain-Loss |
| 245 | x | 4040001 | 4662691 | 622.69 | Gain |
| 246 | x | 5054064 | 18192210 | 13138.146 | Gain |
| 247 | x | 6734423 | 24477135 | 17742.712 | Gain-Loss |
| 248 | x | 65177195 | 71802709 | 6625.514 | Gain-Loss |
| 249 | x | 106109244 | 117864445 | 11755.201 | Gain |

**Table S3:** Genes in all the CNVRs retrieved from Ensembl Genes 64 Database

| Chromosome Name | Gene Start (bp) | Gene End (bp) | Gene Biotype | Associated Gene Name |
| --- | --- | --- | --- | --- |
| 1 | 399819 | 708754 | protein_coding | WDR27 |
| 1 | 440195 | 666100 | protein_coding | TCTE3 |
| 1 | 926901 | 949670 | protein_coding | THBS2 |
| 1 | 1155336 | 1155422 | miRNA |  |
| 1 | 3360697 | 3368706 | protein_coding | T |
| 1 | 3682966 | 3683065 | snRNA | U6 |
| 1 | 3953692 | 4177717 | protein_coding | PDE10A |
| 1 | 6518045 | 7514266 | protein_coding | PARK2 |
| 1 | 7552878 | 7674727 | protein_coding | D0G0C1_PIG |
| 1 | 12300862 | 12417973 | protein_coding | TIAM2 |
| 1 | 12448463 | 12449122 | protein_coding | C3VMW6_PIG |
| 1 | 12971397 | 13016929 | protein_coding | SCAF8 |
| 1 | 15924855 | 15968565 | protein_coding | RMND1 |
| 1 | 16002629 | 16010213 | protein_coding | ZBTB2 |
| 1 | 39276124 | 39295821 | protein_coding | HDDC2 |
| 1 | 39434595 | 39531471 | protein_coding | RNF217 |
| 1 | 39633228 | 39633542 | misc_RNA | 7SK |
| 1 | 51172578 | 51172684 | snRNA | U6 |
| 1 | 51586217 | 51726374 | protein_coding | BAI3 |
| 1 | 52527875 | 52866036 | protein_coding | COL19A1 |
| 1 | 53048513 | 53166595 | protein_coding | FAM135A |
| 1 | 53243221 | 53352644 | protein_coding | SMAP1 |
| 1 | 53749260 | 53762044 | protein_coding | OGFRL1 |
| 1 | 59288100 | 59288255 | snRNA | U1 |
| 1 | 59774444 | 59774540 | miRNA |  |
| 1 | 59901989 | 59902531 | protein_coding | PNRC1 |
| 1 | 59959287 | 59985402 | protein_coding | PM20D2 |
| 1 | 78009825 | 78182648 | protein_coding | LACE1 |
| 1 | 78223324 | 78349962 | protein_coding | Q2XUT2_PIG |
| 1 | 87012811 | 87200539 | protein_coding | F1RQM4_PIG |
| 1 | 98347915 | 98452067 | protein_coding | SETBP1 |
| 1 | 98800585 | 98800887 | misc_RNA | 7SK |
| 1 | 99064230 | 99123148 | protein_coding | SLC14A2 |
| 1 | 99161725 | 99183172 | protein_coding | SLC14A1 |
| 1 | 99284559 | 99290281 | protein_coding | SIGLEC15 |
| 1 | 99745958 | 99757458 | protein_coding | F1RPS8_PIG |
| 1 | 99827399 | 99872158 | protein_coding | C18orf25 |
| 1 | 100899228 | 100899611 | protein_coding | ZBTB7C |
| 1 | 101379400 | 101610813 | protein_coding | CTIF |
| 1 | 123878479 | 123922632 | protein_coding | FAM214A |
| 1 | 123932964 | 123952385 | protein_coding | ARP19_PIG |
| 1 | 124405686 | 124451142 | protein_coding | GNB5 |
| 1 | 124460310 | 124462934 | protein_coding | BCL2L10 |
| 1 | 124668854 | 124921995 | protein_coding | LEO1 |
| 1 | 125042676 | 125059238 | protein_coding | LYSMD2 |
| 1 | 125241287 | 125313638 | protein_coding | DMXL2 |
| 1 | 133305454 | 133347944 | protein_coding | CASC4 |
| 1 | 133677750 | 133724354 | protein_coding | FRMD5 |
| 1 | 133817037 | 133831668 | protein_coding | MFAP1 |
| 1 | 133835904 | 133840398 | protein_coding | SERINC4 |
| 1 | 133841669 | 133841769 | miRNA |  |
| 1 | 133856999 | 133860422 | protein_coding | ELL3 |
| 1 | 133890106 | 133904423 | protein_coding | CATSPER2 |
| 1 | 133905958 | 133906075 | rRNA | 5S_rRNA |
| 1 | 133911582 | 133928042 | protein_coding | STRC |
| 1 | 133941808 | 133986378 | protein_coding | PPIP5K1 |
| 1 | 134062654 | 134062722 | miRNA |  |
| 1 | 134068598 | 134101046 | protein_coding | TP53BP1 |
| 1 | 134176671 | 134186378 | protein_coding | ZSCAN29 |
| 1 | 134217565 | 134219622 | protein_coding | LCMT2 |
| 1 | 134383649 | 134404828 | protein_coding | EPB42 |
| 1 | 134517219 | 134599326 | protein_coding | UBR1 |
| 1 | 134643557 | 134764211 | protein_coding | TTBK2 |
| 1 | 134776546 | 134786882 | protein_coding | CDAN1 |
| 1 | 135138453 | 135144910 | protein_coding | LRRC57 |
| 1 | 155432891 | 155487316 | protein_coding | ZNF516 |
| 1 | 155448552 | 155448673 | miRNA |  |
| 1 | 156237051 | 156240158 | protein_coding | TSHZ1 |
| 1 | 172292133 | 172329373 | protein_coding | AAGAB |
| 1 | 172527891 | 172706294 | protein_coding | IQCH |
| 1 | 172599737 | 172604587 | protein_coding | C15orf61 |
| 1 | 172974539 | 172982727 | protein_coding | SKOR1 |
| 1 | 173268973 | 173355949 | protein_coding | PIAS1 |
| 1 | 206192199 | 206192828 | protein_coding | TUSC1 |
| 1 | 207322647 | 207326193 | protein_coding | IZUMO3 |
| 1 | 216336590 | 216510638 | protein_coding | PSIP1 |
| 1 | 216840600 | 216893578 | protein_coding | TTC39B |
| 1 | 217060624 | 217076052 | protein_coding | FREM1 |
| 1 | 217235840 | 217236148 | protein_coding | CER1 |
| 1 | 233860965 | 233876054 | protein_coding | MAMDC2 |
| 1 | 234047725 | 234071076 | protein_coding | KLF9_PIG |
| 1 | 234133341 | 234133433 | miRNA |  |
| 1 | 243320848 | 243391371 | protein_coding | TLE4 |
| 1 | 243377663 | 243377803 | snoRNA | SNORA67 |
| 1 | 290553326 | 290555475 | protein_coding | KATNAL2 |
| 1 | 290810029 | 290825625 | protein_coding | RBFA |
| 1 | 290863346 | 290888776 | protein_coding | ADNP2 |
| 1 | 290997073 | 291013175 | protein_coding | TXNL4A |
| 1 | 291035927 | 291092499 | protein_coding | PQLC1 |
| 1 | 291920369 | 291931818 | protein_coding | TM2D3 |
| 1 | 292004446 | 292004552 | snRNA | U6 |
| 1 | 292192711 | 292222288 | protein_coding | PCSK6 |
| 1 | 292344640 | 292356715 | protein_coding | SNRPA1 |
| 1 | 292750263 | 292751171 | pseudogene |  |
| 1 | 292960972 | 292961927 | pseudogene |  |
| 1 | 293008098 | 293008938 | protein_coding | OR5AS1 |
| 1 | 293195862 | 293196810 | pseudogene |  |
| 1 | 294107535 | 294114747 | protein_coding | EGFL7 |
| 1 | 294113077 | 294113135 | miRNA |  |
| 1 | 294124723 | 294131498 | protein_coding | KIAA1984 |
| 1 | 294154261 | 294158159 | protein_coding | MAMDC4 |
| 1 | 294205870 | 294206000 | snoRNA | SNORA17 |
| 1 | 294207193 | 294207315 | snoRNA | SNORA17 |
| 1 | 294218229 | 294223476 | protein_coding | TRAF2 |
| 1 | 294236972 | 294238512 | protein_coding | A0SEH3_PIG |
| 1 | 294245121 | 294252161 | protein_coding | C9orf86 |
| 1 | 294295401 | 294295745 | protein_coding | NRARP |
| 1 | 294343524 | 294347323 | protein_coding | FAM166A |
| 1 | 294347800 | 294347910 | miRNA |  |
| 1 | 294355182 | 294356788 | protein_coding | NELF |
| 1 | 294406204 | 294431198 | protein_coding | EXD3 |
| 1 | 294439810 | 294440604 | protein_coding | RNF208 |
| 1 | 294451590 | 294452000 | protein_coding | TMEM203 |
| 1 | 295508153 | 295509081 | protein_coding | OR1N1 |
| 2 | 162398 | 167169 | protein_coding | PIDD |
| 2 | 176014 | 178780 | protein_coding | CEND_PIG |
| 2 | 185919 | 191739 | protein_coding | PDDC1 |
| 2 | 238775 | 261809 | protein_coding | DEAF1 |
| 2 | 282142 | 290232 | protein_coding | SECR_PIG |
| 2 | 291676 | 294411 | protein_coding | A0ZVR0_PIG |
| 2 | 331299 | 331396 | miRNA | ssc-mir-210 |
| 2 | 564821 | 565855 | pseudogene |  |
| 2 | 1273564 | 1283402 | protein_coding | SHANK2 |
| 2 | 1354813 | 1355118 | protein_coding | Q56VC2_PIG |
| 2 | 1686278 | 1688039 | protein_coding | FGF4 |
| 2 | 1742745 | 1746468 | protein_coding | FGF19 |
| 2 | 1757505 | 1765881 | protein_coding | FGF3 |
| 2 | 1833419 | 1840936 | protein_coding | ORAOV1 |
| 2 | 2298255 | 2314866 | protein_coding | TPCN2 |
| 2 | 2397331 | 2398278 | protein_coding | MRGPRD |
| 2 | 2436853 | 2448144 | protein_coding | MRPL21 |
| 2 | 2540801 | 2641517 | protein_coding | MTL5 |
| 2 | 2964562 | 2974104 | protein_coding | C11orf24 |
| 2 | 3012437 | 3110208 | protein_coding | SUV420H1 |
| 2 | 3133038 | 3293093 | protein_coding | TBX10 |
| 2 | 3133576 | 3190746 | protein_coding | CHKA |
| 2 | 3294498 | 3309019 | protein_coding | NUDT8 |
| 2 | 3331731 | 3350753 | protein_coding | F1RVM9_PIG |
| 2 | 3406772 | 3408962 | protein_coding | CDK2AP2 |
| 2 | 3411264 | 3423542 | protein_coding | PITPNM1 |
| 2 | 3438571 | 3443453 | protein_coding | TMEM134 |
| 2 | 3456129 | 3456956 | protein_coding | GPR152 |
| 2 | 3468942 | 3469568 | protein_coding | PTPRCAP |
| 2 | 3501365 | 3504471 | protein_coding | F1RUX8_PIG |
| 2 | 3562263 | 3564074 | protein_coding | POLD4 |
| 2 | 3943363 | 3948616 | protein_coding | PC |
| 2 | 4043508 | 4051227 | protein_coding | SPTBN2 |
| 2 | 4121849 | 4163669 | protein_coding | CCS |
| 2 | 4282080 | 4286831 | protein_coding | NPAS4 |
| 2 | 4318313 | 4327076 | protein_coding | PELI3 |
| 2 | 4331535 | 4371006 | protein_coding | DPP3 |
| 2 | 4432363 | 4436964 | protein_coding | RIN1 |
| 2 | 4475173 | 4479363 | protein_coding | YIF1A |
| 2 | 4686131 | 4694266 | protein_coding | CATSPER1 |
| 2 | 4707899 | 4710966 | protein_coding | EIF1AD |
| 2 | 4790094 | 4790849 | protein_coding | C11orf68 |
| 2 | 4817859 | 4823304 | protein_coding | FIBP |
| 2 | 4832868 | 4865376 | protein_coding | EFEMP2 |
| 2 | 4873775 | 4876747 | protein_coding | COFILIN |
| 2 | 4961454 | 4963251 | protein_coding | RNASEH2C |
| 2 | 5053264 | 5066070 | protein_coding | SCYL1 |
| 2 | 5450772 | 5450875 | snRNA | U6 |
| 2 | 5471274 | 5473282 | protein_coding | RS30_PIG |
| 2 | 5475869 | 5477068 | protein_coding | ZNHIT2 |
| 2 | 5504232 | 5509330 | protein_coding | CDCA5 |
| 2 | 5528118 | 5538804 | protein_coding | NAALADL1 |
| 2 | 5621411 | 5642123 | protein_coding | C11orf85 |
| 2 | 5646978 | 5647633 | protein_coding | GPHA2 |
| 2 | 5685208 | 5685291 | miRNA |  |
| 2 | 5685399 | 5685508 | miRNA |  |
| 2 | 5699171 | 5726031 | protein_coding | EHD1 |
| 2 | 5734333 | 5752406 | protein_coding | CDC42BPG |
| 2 | 5762005 | 5765616 | protein_coding | MEN1 |
| 2 | 5767117 | 5778518 | protein_coding | MAP4K2 |
| 2 | 5792161 | 5805583 | protein_coding | SF1 |
| 2 | 5810420 | 5821868 | protein_coding | F1RQQ7_PIG |
| 2 | 5823661 | 5839410 | protein_coding | RASGRP2 |
| 2 | 5852514 | 5951864 | protein_coding | NRXN2 |
| 2 | 6156279 | 6159332 | protein_coding | C11orf20 |
| 2 | 6161598 | 6168487 | protein_coding | F1RQP2_PIG |
| 2 | 6170436 | 6173823 | protein_coding | PRDX5 |
| 2 | 9625649 | 9643068 | protein_coding | GIF |
| 2 | 9797003 | 9872891 | protein_coding | PATL1 |
| 2 | 9819983 | 9820105 | miRNA |  |
| 2 | 9892169 | 9925521 | protein_coding | F1RMJ9_PIG |
| 2 | 10019957 | 10020903 | pseudogene |  |
| 2 | 10039519 | 10040345 | pseudogene |  |
| 2 | 10278894 | 10281047 | protein_coding | MPEG1 |
| 2 | 10414084 | 10420349 | protein_coding | GLYATL2 |
| 2 | 10448380 | 10467284 | protein_coding | GLYAT |
| 2 | 10619294 | 10619438 | pseudogene |  |
| 2 | 11028804 | 11029390 | pseudogene |  |
| 2 | 11224958 | 11232495 | protein_coding | BTBD18 |
| 2 | 11246439 | 11254785 | protein_coding | MED19 |
| 2 | 11294609 | 11298941 | protein_coding | YPEL4 |
| 2 | 12783803 | 12783906 | snRNA | U6 |
| 2 | 12881796 | 12881986 | snRNA | U2 |
| 2 | 12949347 | 12982287 | protein_coding | AGBL2 |
| 2 | 13068848 | 13074399 | protein_coding | PTPMT1 |
| 2 | 13089123 | 13090796 | protein_coding | FAM180B |
| 2 | 13144668 | 13159069 | protein_coding | CELF1 |
| 2 | 13279591 | 13287617 | protein_coding | SLC39A13 |
| 2 | 13357967 | 13365882 | protein_coding | ACP2 |
| 2 | 13405836 | 13413834 | protein_coding | PACSIN3 |
| 2 | 13414544 | 13424685 | protein_coding | ARFGAP2 |
| 2 | 13505544 | 13505642 | snRNA | U6 |
| 2 | 13667626 | 13706373 | protein_coding | LRP4 |
| 2 | 13964296 | 13990453 | protein_coding | F1SIB0_PIG |
| 2 | 14057381 | 14057491 | snoRNA | SNORD67 |
| 2 | 14062249 | 14062346 | snoRNA | SNORD67 |
| 2 | 14123252 | 14139957 | protein_coding | ARHGAP1 |
| 2 | 14204224 | 14213480 | protein_coding | HARBI1 |
| 2 | 14223249 | 14232715 | protein_coding | DGKZ |
| 2 | 14234375 | 14236470 | protein_coding | D3K5N3_PIG |
| 2 | 14569968 | 14840508 | protein_coding | PHF21A |
| 2 | 14854637 | 14860353 | protein_coding | PEX16 |
| 2 | 14863004 | 14863760 | protein_coding | C11orf94 |
| 2 | 26215319 | 26230799 | protein_coding | PRRG4 |
| 2 | 26292906 | 26334078 | protein_coding | QSER1 |
| 2 | 26751078 | 26751228 | pseudogene |  |
| 2 | 26773970 | 26774055 | miRNA |  |
| 2 | 30907352 | 31012103 | protein_coding | LGR4 |
| 2 | 31019014 | 31056128 | protein_coding | CCDC34 |
| 2 | 53126801 | 53152421 | protein_coding | TYK2 |
| 2 | 53159984 | 53167142 | protein_coding | ICAM3 |
| 2 | 53167362 | 53179202 | protein_coding | RAVER1 |
| 2 | 53180692 | 53185449 | protein_coding | FDX1L |
| 2 | 53238043 | 53275859 | protein_coding | F1S3I7_PIG |
| 2 | 54240581 | 54241070 | pseudogene |  |
| 2 | 55929184 | 55934339 | protein_coding | WDR83 |
| 2 | 55979609 | 55989030 | protein_coding | ASNA1 |
| 2 | 56008241 | 56011610 | protein_coding | B4YSE2_PIG |
| 2 | 56013371 | 56016339 | protein_coding | DNASE2 |
| 2 | 56026367 | 56031148 | protein_coding | BEST2 |
| 2 | 56058510 | 56059554 | pseudogene |  |
| 2 | 56069015 | 56075585 | protein_coding | F1SDX8_PIG |
| 2 | 56113769 | 56115760 | protein_coding | SYCE2 |
| 2 | 56138308 | 56142589 | protein_coding | DAND5 |
| 2 | 56180425 | 56250362 | protein_coding | F1SDA0_PIG |
| 2 | 56285653 | 56289452 | protein_coding | D4N5N1_PIG |
| 2 | 56291244 | 56297121 | protein_coding | RAD23A |
| 2 | 56366058 | 56366723 | protein_coding | IER2 |
| 2 | 56797201 | 56813176 | protein_coding | CCDC130 |
| 2 | 56822887 | 56826748 | protein_coding | C19orf53 |
| 2 | 56833996 | 56857014 | protein_coding | ZSWIM4 |
| 2 | 56889312 | 56889408 | miRNA | ssc-mir-181c |
| 2 | 56889474 | 56889605 | miRNA |  |
| 2 | 56890994 | 56893899 | protein_coding | NANOS3 |
| 2 | 56916863 | 56933585 | protein_coding | CC2D1A |
| 2 | 56955436 | 56963124 | protein_coding | DCAF15 |
| 2 | 57015987 | 57018545 | protein_coding | REL3_PIG |
| 2 | 57019256 | 57041607 | protein_coding | IL27RA |
| 2 | 57169360 | 57186527 | protein_coding | CD97 |
| 2 | 57238620 | 57264277 | protein_coding | NDUFB7 |
| 2 | 57305113 | 57306333 | protein_coding | PTGER1 |
| 2 | 64155617 | 64155850 | protein_coding | C1orf150 |
| 2 | 64686221 | 64699249 | protein_coding | TRIM58 |
| 2 | 65617924 | 65618181 | protein_coding | ZNF692 |
| 2 | 65645480 | 65657194 | protein_coding | SH3BP5L |
| 2 | 72677073 | 72716900 | protein_coding | D0G6X8_PIG |
| 2 | 72774741 | 72782022 | protein_coding | NSA2 |
| 2 | 73236691 | 73258160 | protein_coding | F1S2I4_PIG |
| 2 | 73451370 | 73451483 | rRNA | 5S_rRNA |
| 2 | 73478095 | 73542057 | protein_coding | ANKDD1B |
| 3 | 15183774 | 15198528 | protein_coding | BGLR_PIG |
| 3 | 43855839 | 43885293 | protein_coding | ST6GAL2 |
| 3 | 43974258 | 43985344 | protein_coding | C2orf40 |
| 3 | 44145878 | 44155955 | protein_coding | NCK2 |
| 3 | 46936771 | 46949532 | protein_coding | MFSD9 |
| 3 | 47646110 | 47670156 | protein_coding | IL1RL2 |
| 3 | 48504604 | 48572483 | protein_coding | RFX8 |
| 3 | 48582289 | 48636307 | protein_coding | CREG2 |
| 3 | 48719552 | 48734844 | protein_coding | C2orf29 |
| 3 | 48826867 | 49011811 | protein_coding | TBC1D8 |
| 3 | 49597731 | 49631933 | protein_coding | CHST10 |
| 3 | 49676255 | 49704839 | protein_coding | LONRF2 |
| 3 | 50472779 | 50518327 | protein_coding | REV1 |
| 3 | 50741024 | 50748745 | protein_coding | MRPL30 |
| 3 | 50772783 | 50788131 | protein_coding | MITD1 |
| 3 | 50821112 | 50911378 | protein_coding | TSGA10 |
| 3 | 51060788 | 51079966 | protein_coding | C2orf55 |
| 3 | 51156062 | 51252902 | protein_coding | MGAT4A |
| 3 | 51268814 | 51278875 | protein_coding | COA5 |
| 3 | 51652178 | 51779803 | protein_coding | TMEM131 |
| 3 | 51763191 | 51763457 | misc_RNA | 7SK |
| 3 | 51974950 | 52260205 | protein_coding | FAM178B |
| 3 | 52065783 | 52074136 | protein_coding | SEMA4C |
| 3 | 52076536 | 52088376 | protein_coding | ANKRD39 |
| 3 | 52131944 | 52148592 | protein_coding | LMAN2L |
| 3 | 52166600 | 52171312 | protein_coding | ANKRD23 |
| 3 | 52496620 | 52579270 | protein_coding | EIF2AK3 |
| 3 | 52716117 | 52821936 | protein_coding | CD8B |
| 3 | 52763223 | 52766114 | protein_coding | FABPL_PIG |
| 3 | 52871768 | 52906901 | protein_coding | RMND5A |
| 3 | 52992955 | 53014866 | protein_coding | RNF103 |
| 3 | 53378880 | 53467925 | protein_coding | REEP1 |
| 3 | 53578822 | 53658397 | protein_coding | F1SVC3_PIG |
| 3 | 53909861 | 53914687 | protein_coding | NKL_PIG |
| 3 | 53976698 | 53987113 | protein_coding | A7MAK5_PIG |
| 3 | 54029193 | 54040001 | protein_coding | ATOH8 |
| 3 | 54044733 | 54045914 | protein_coding | VAMP5 |
| 3 | 54049028 | 54050862 | protein_coding | RNF181 |
| 3 | 54069003 | 54084558 | protein_coding | USP39 |
| 3 | 54090565 | 54094950 | protein_coding | TGOLN2 |
| 3 | 54114670 | 54145739 | protein_coding | ELMOD3 |
| 3 | 54190886 | 54196554 | protein_coding | SH2D6 |
| 3 | 54737640 | 54738656 | protein_coding | TYB10_PIG |
| 3 | 54911164 | 54946361 | protein_coding | F1SNZ7_PIG |
| 3 | 57389262 | 57389527 | misc_RNA | 7SK |
| 4 | 338129 | 350394 | protein_coding | ZNF34 |
| 4 | 440052 | 441495 | protein_coding | FOXH1 |
| 4 | 449645 | 462836 | protein_coding | CYHR1 |
| 4 | 479519 | 484656 | protein_coding | VPS28 |
| 4 | 490722 | 495137 | protein_coding | SLC39A4 |
| 4 | 499626 | 508181 | protein_coding | CPSF1 |
| 4 | 538974 | 542119 | protein_coding | FBXL6 |
| 4 | 584181 | 586642 | protein_coding | CYC1 |
| 4 | 592686 | 595749 | protein_coding | MAF1 |
| 4 | 595866 | 683192 | protein_coding | KIAA1875 |
| 4 | 957963 | 985401 | protein_coding | HEATR7A |
| 4 | 1451764 | 1491370 | protein_coding | DENND3 |
| 4 | 1573343 | 1582321 | protein_coding | PTK2 |
| 4 | 1712609 | 2190793 | protein_coding | TRAPPC9 |
| 4 | 3269610 | 3358600 | protein_coding | FAM135B |
| 4 | 3679694 | 3679911 | pseudogene |  |
| 4 | 29418871 | 29582043 | protein_coding | RSPO2 |
| 4 | 35169256 | 35201595 | protein_coding | RRM2B |
| 4 | 40685100 | 40693748 | protein_coding | PTDSS1 |
| 4 | 40784491 | 40828708 | protein_coding | MTERFD1 |
| 4 | 40832391 | 40838170 | protein_coding | UQCRB |
| 4 | 41914598 | 41933543 | protein_coding | C8orf37 |
| 4 | 42100428 | 42107040 | protein_coding | TP53INP1 |
| 4 | 42631931 | 42695753 | protein_coding | KIAA1429 |
| 4 | 43065408 | 43076341 | protein_coding | GEM |
| 4 | 43120544 | 43202644 | protein_coding | CDH17 |
| 4 | 43617528 | 43643888 | protein_coding | FAM92A1 |
| 4 | 43655200 | 43655462 | pseudogene |  |
| 4 | 44283186 | 44366668 | protein_coding | C8orf83 |
| 4 | 45117043 | 45318025 | protein_coding | RUNX1T1 |
| 4 | 46360835 | 46424350 | protein_coding | TMEM55A |
| 4 | 47050724 | 47071443 | protein_coding | TMEM64 |
| 4 | 47870808 | 47893791 | protein_coding | B3VFB6_PIG |
| 4 | 47957663 | 47976943 | protein_coding | NBN |
| 4 | 48800836 | 48800936 | rRNA | 5S_rRNA |
| 4 | 48869790 | 48869893 | rRNA | 5S_rRNA |
| 4 | 50225464 | 50382640 | protein_coding | MMP16 |
| 4 | 51946984 | 52023562 | protein_coding | CNGB3 |
| 4 | 52105222 | 52143168 | protein_coding | FAM82B |
| 4 | 53187533 | 53197072 | protein_coding | CA1 |
| 4 | 58931803 | 58934647 | protein_coding | HEY1 |
| 4 | 58969515 | 58969621 | snRNA | U6 |
| 4 | 59631743 | 59631848 | snRNA | U6 |
| 4 | 59739634 | 59798926 | protein_coding | IL7_PIG |
| 4 | 95935308 | 95935924 | pseudogene |  |
| 4 | 96025067 | 96042589 | protein_coding | CD5L |
| 4 | 96046240 | 96074811 | protein_coding | FCRL1 |
| 4 | 96091589 | 96132057 | protein_coding | FCRL3 |
| 4 | 96174027 | 96189991 | protein_coding | FCRL4 |
| 4 | 96799050 | 96852957 | protein_coding | ARHGEF11 |
| 4 | 96937361 | 96950761 | protein_coding | F1RHJ5_PIG |
| 4 | 96975677 | 96984979 | protein_coding | SH2D2A |
| 4 | 97034245 | 97043525 | protein_coding | HDGF |
| 4 | 97059936 | 97064585 | protein_coding | ISG20L2 |
| 4 | 97081032 | 97087113 | protein_coding | F1RHI8_PIG |
| 4 | 97238167 | 97252128 | protein_coding | BCAN |
| 4 | 97273615 | 97275865 | protein_coding | HAPLN2 |
| 4 | 97316353 | 97323706 | protein_coding | TTC24 |
| 4 | 97329396 | 97332301 | protein_coding | APOA1BP |
| 4 | 97531326 | 97531414 | miRNA | ssc-mir-9-1 |
| 4 | 97601379 | 97656994 | protein_coding | TCPG_PIG |
| 4 | 97662587 | 97665559 | protein_coding | C1orf85 |
| 4 | 110770295 | 110827764 | protein_coding | SYT6 |
| 4 | 110843830 | 110843909 | miRNA |  |
| 4 | 113312454 | 113322817 | protein_coding | PEPB_PIG |
| 4 | 113553784 | 113568329 | protein_coding | DENND2D |
| 4 | 113604028 | 113624088 | protein_coding | DRAM2 |
| 4 | 113705267 | 113710293 | protein_coding | LRIF1 |
| 4 | 113840448 | 113840552 | snRNA | U6 |
| 4 | 113914483 | 113915919 | protein_coding | KCNA3 |
| 4 | 127967653 | 127995417 | protein_coding | BCAR3 |
| 4 | 128569280 | 128579565 | protein_coding | FAM69A |
| 4 | 128665273 | 128810918 | protein_coding | EVI5 |
| 4 | 128849119 | 128856292 | protein_coding | GFI1 |
| 4 | 128954136 | 129039420 | protein_coding | GLMN |
| 4 | 129228108 | 129228492 | pseudogene |  |
| 4 | 129336808 | 129506717 | protein_coding | TGFBR3 |
| 4 | 129781480 | 129851869 | protein_coding | HFM1 |
| 4 | 130070302 | 130094250 | protein_coding | ZNF644 |
| 4 | 131106043 | 131106323 | misc_RNA | 7SK |
| 5 | 115309 | 121710 | protein_coding | PKDREJ |
| 5 | 126757 | 134285 | protein_coding | C22orf40 |
| 5 | 259205 | 283748 | protein_coding | ATXN10 |
| 5 | 422624 | 422725 | snRNA | U6 |
| 5 | 527240 | 527346 | snRNA | U6 |
| 5 | 927612 | 927676 | miRNA |  |
| 5 | 1072079 | 1102094 | protein_coding | PHF21B |
| 5 | 1460374 | 1461093 | protein_coding | LDOC1L |
| 5 | 1606991 | 1621473 | protein_coding | KIAA1644 |
| 5 | 1678491 | 1711481 | protein_coding | PARVB |
| 5 | 1722676 | 1746963 | protein_coding | PARVG |
| 5 | 1964991 | 2127081 | protein_coding | EFCAB6 |
| 5 | 1997682 | 1997803 | rRNA | 5S_rRNA |
| 5 | 2170487 | 2367358 | protein_coding | SCUBE1 |
| 5 | 2428901 | 2432187 | protein_coding | TSPO |
| 5 | 2455372 | 2505617 | protein_coding | TTLL1 |
| 5 | 2502656 | 2679140 | protein_coding | PACSIN2 |
| 5 | 2689009 | 2743987 | protein_coding | ARFGAP3 |
| 5 | 2835494 | 2858808 | protein_coding | CYB5R3 |
| 5 | 3105324 | 3110333 | protein_coding | F1SRG2_PIG |
| 5 | 3122858 | 3130834 | protein_coding | NAGA |
| 5 | 3435637 | 3444127 | protein_coding | NHP2L1 |
| 5 | 3474339 | 3497158 | protein_coding | PPPDE2 |
| 5 | 3510467 | 3521513 | protein_coding | PMM1 |
| 5 | 3542222 | 3556797 | protein_coding | POLR3H |
| 5 | 3613869 | 3616629 | protein_coding | MEI1 |
| 5 | 3637584 | 3656975 | protein_coding | L3MBTL2 |
| 5 | 3838049 | 3839269 | pseudogene |  |
| 5 | 3846661 | 3879365 | protein_coding | ST13 |
| 5 | 4066104 | 4066206 | snoRNA | snoU13 |
| 5 | 4147383 | 4147460 | miRNA |  |
| 5 | 4225678 | 4243378 | protein_coding | MKL1 |
| 5 | 4717895 | 4864079 | protein_coding | ENTHD1 |
| 5 | 5054999 | 5058430 | protein_coding | RPS19BP1 |
| 5 | 5237453 | 5237516 | snoRNA | SNORD43 |
| 5 | 5240518 | 5240611 | snoRNA | snoU83B |
| 5 | 5353997 | 5356390 | protein_coding | APOBEC3F |
| 5 | 5409889 | 5428631 | protein_coding | NPTXR |
| 5 | 5439045 | 5450464 | protein_coding | DNAL4_PIG |
| 5 | 5479126 | 5494767 | protein_coding | SUN2 |
| 5 | 5656591 | 5667018 | protein_coding | B2CAW6_PIG |
| 5 | 5993095 | 6039477 | protein_coding | TMEM184B |
| 5 | 6230165 | 6283395 | protein_coding | PLA2G6 |
| 5 | 6283965 | 6303901 | protein_coding | BAIAP2L2 |
| 5 | 6357460 | 6359833 | protein_coding | SLC16A8 |
| 5 | 6445847 | 6454909 | protein_coding | Q6PU48_PIG |
| 5 | 6471938 | 6478829 | protein_coding | C22orf23 |
| 5 | 6756169 | 6851030 | protein_coding | TRIOBP |
| 5 | 6883939 | 6914392 | protein_coding | CYTH4 |
| 5 | 7074316 | 7080037 | protein_coding | MPST |
| 5 | 7100429 | 7111159 | protein_coding | KCTD17 |
| 5 | 7325480 | 7342788 | protein_coding | PVALB1 |
| 5 | 7363643 | 7384518 | protein_coding | IFT27 |
| 5 | 7581330 | 7599506 | protein_coding | CACNG2 |
| 5 | 7628707 | 7645055 | protein_coding | EIF3D |
| 5 | 7704458 | 7717661 | protein_coding | TXN2 |
| 5 | 15176248 | 15197625 | protein_coding | POU6F1 |
| 5 | 15234969 | 15294617 | protein_coding | CELA1_PIG |
| 5 | 15298248 | 15378045 | protein_coding | BIN2 |
| 5 | 15383458 | 15403175 | protein_coding | SMAGP |
| 5 | 15957758 | 15960053 | protein_coding | ANKRD33 |
| 5 | 16023608 | 16057075 | protein_coding | F1SGJ9_PIG |
| 5 | 16067495 | 16074633 | protein_coding | GRASP |
| 5 | 16237783 | 16237858 | miRNA |  |
| 5 | 16714898 | 16722754 | protein_coding | F1SGG2_PIG |
| 5 | 16789336 | 16789408 | miRNA |  |
| 5 | 16871347 | 16873949 | protein_coding | ZNF740 |
| 5 | 16969902 | 16984781 | protein_coding | SPRYD3 |
| 5 | 17017819 | 17019171 | protein_coding | MFSD5 |
| 5 | 17027793 | 17050650 | protein_coding | ESPL1 |
| 5 | 17059988 | 17065529 | protein_coding | C12orf10 |
| 5 | 17203786 | 17208521 | protein_coding | PRR13 |
| 5 | 17219199 | 17241964 | protein_coding | PCBP2 |
| 5 | 17221279 | 17221381 | snRNA | U6 |
| 5 | 17267545 | 17272606 | protein_coding | TARBP2 |
| 5 | 17804056 | 17815030 | protein_coding | CBX5 |
| 5 | 17966352 | 17970679 | protein_coding | Q06A94_PIG |
| 5 | 18080339 | 18099538 | protein_coding | COPZ1 |
| 5 | 18088729 | 18088818 | miRNA | MIR148B |
| 5 | 18292784 | 18318461 | protein_coding | PDE1B |
| 5 | 18565674 | 18566669 | protein_coding | NEUROD4 |
| 5 | 20213711 | 20216204 | protein_coding | ORMDL2 |
| 5 | 20226035 | 20229728 | protein_coding | TMEM198 |
| 5 | 20284080 | 20308013 | protein_coding | DGKA_PIG |
| 5 | 20332627 | 20338207 | protein_coding | CDK2 |
| 5 | 20416470 | 20417777 | protein_coding | ZC3H10 |
| 5 | 20423893 | 20440455 | protein_coding | ESYT1 |
| 5 | 20477435 | 20480574 | protein_coding | MYL6_PIG |
| 5 | 20546900 | 20676966 | protein_coding | OBFC2B |
| 5 | 20553964 | 20559810 | protein_coding | SLC39A5 |
| 5 | 20578456 | 20578531 | miRNA |  |
| 5 | 20587725 | 20591158 | protein_coding | COQ10A |
| 5 | 20698852 | 20712565 | protein_coding | PAN2 |
| 5 | 20713522 | 20716976 | protein_coding | CNPY2 |
| 5 | 20721361 | 20722926 | protein_coding | IL23A_PIG |
| 5 | 20979495 | 20990348 | protein_coding | SPRYD4 |
| 5 | 21185853 | 21199676 | protein_coding | E5F1H4_PIG |
| 5 | 21374983 | 21377063 | protein_coding | NDUFA4L2 |
| 5 | 21618578 | 21619286 | protein_coding | B8Q504_PIG |
| 5 | 21956856 | 21959987 | protein_coding | 9-Mar |
| 5 | 21974411 | 21975865 | protein_coding | AGAP2 |
| 5 | 21979299 | 22007996 | protein_coding | OS9 |
| 5 | 22038376 | 22038482 | snRNA | U6 |
| 5 | 22062455 | 22105919 | protein_coding | XRCC6BP1 |
| 5 | 29310864 | 29381426 | protein_coding | LEMD3 |
| 5 | 29406217 | 29561170 | protein_coding | MSRB3 |
| 5 | 29805695 | 29805785 | miRNA |  |
| 5 | 29963950 | 29964040 | miRNA |  |
| 5 | 30069586 | 30083180 | protein_coding | HMGA2 |
| 5 | 30104034 | 30104153 | miRNA |  |
| 5 | 30310013 | 30310133 | rRNA | 5S_rRNA |
| 5 | 30401221 | 30401621 | pseudogene |  |
| 5 | 30472898 | 30545160 | protein_coding | IRAK3 |
| 5 | 30591883 | 30636962 | protein_coding | HELB |
| 5 | 55953038 | 56011319 | protein_coding | GRIN2B |
| 5 | 56252181 | 56253014 | protein_coding | F1SQ83_PIG |
| 5 | 65172375 | 65180689 | protein_coding | CECR5 |
| 5 | 65217498 | 65222396 | protein_coding | IL17RA |
| 5 | 65417079 | 65432963 | protein_coding | CECR2 |
| 5 | 65471948 | 65530801 | protein_coding | BCL2L13 |
| 5 | 67148076 | 67217875 | protein_coding | C12orf40 |
| 5 | 67498973 | 67499115 | snoRNA | SNORA67 |
| 5 | 67768872 | 67911260 | protein_coding | F1SHN7_PIG |
| 5 | 67947833 | 67977247 | protein_coding | LOC396897 |
| 5 | 69164686 | 69235000 | protein_coding | PDZRN4 |
| 5 | 69633907 | 69637848 | protein_coding | ZCRB1 |
| 5 | 69649053 | 69763125 | protein_coding | PPHLN1 |
| 5 | 69956834 | 69957174 | misc_RNA | 7SK |
| 5 | 70896710 | 70907659 | protein_coding | TWF1 |
| 5 | 72208598 | 72257972 | protein_coding | ANO6 |
| 5 | 72250924 | 72251032 | rRNA | 5S_rRNA |
| 5 | 72548767 | 72548910 | protein_coding | ARID2 |
| 5 | 73096930 | 73097880 | protein_coding | AMIGO2 |
| 6 | 36752 | 392496 | protein_coding | SPIRE2 |
| 6 | 130905 | 132406 | protein_coding | DBNDD1 |
| 6 | 178404 | 182210 | protein_coding | DEF8 |
| 6 | 380948 | 384224 | protein_coding | TRAPPC2L |
| 6 | 558798 | 568734 | protein_coding | KLHDC4 |
| 6 | 635969 | 636103 | snoRNA | SNORA70 |
| 6 | 804217 | 824752 | protein_coding | D7RA29_PIG |
| 6 | 910339 | 910425 | miRNA |  |
| 6 | 1383572 | 1399454 | protein_coding | MTHFSD |
| 6 | 1808261 | 1810310 | protein_coding | C16orf74 |
| 6 | 1817835 | 1879692 | protein_coding | F1S6I3_PIG |
| 6 | 2350755 | 2381053 | protein_coding | ZDHHC7 |
| 6 | 4440703 | 4440998 | misc_RNA | 7SK |
| 6 | 9562201 | 9562425 | protein_coding | EXOSC6 |
| 6 | 9585893 | 9585998 | snRNA | U6 |
| 6 | 9757914 | 9818204 | protein_coding | SF3B3 |
| 6 | 20634236 | 20659554 | protein_coding | CES1 |
| 6 | 31931677 | 31953785 | protein_coding | ZFP30 |
| 6 | 32089211 | 32089502 | misc_RNA | 7SK |
| 6 | 32291611 | 32292632 | pseudogene |  |
| 6 | 33318952 | 33319140 | protein_coding | LEUTX |
| 6 | 33533100 | 33560034 | protein_coding | AKT2 |
| 6 | 33573677 | 33574819 | protein_coding | TTC9B |
| 6 | 33615478 | 33623040 | protein_coding | Q8SQ68_PIG |
| 6 | 34639079 | 34644403 | protein_coding | D3K5J4_PIG |
| 6 | 34648631 | 34665650 | protein_coding | F1RGF9_PIG |
| 6 | 34671296 | 34674226 | protein_coding | RABAC1 |
| 6 | 34876750 | 34879693 | protein_coding | TEX101 |
| 6 | 34884420 | 34886452 | protein_coding | PB1_PIG |
| 6 | 34896709 | 34899322 | protein_coding | ZNF575 |
| 6 | 34943586 | 34945915 | protein_coding | ZNF576 |
| 6 | 34955504 | 34957372 | protein_coding | SRRM5 |
| 6 | 35144416 | 35145807 | protein_coding | IRGC |
| 6 | 35219429 | 35231028 | protein_coding | ZNF45 |
| 6 | 35266806 | 35273669 | protein_coding | ZNF404 |
| 6 | 35350245 | 35354566 | protein_coding | LYPD5 |
| 6 | 35357926 | 35358015 | miRNA |  |
| 6 | 36120469 | 36442882 | protein_coding | MYPOP |
| 6 | 36494878 | 36577230 | protein_coding | ARHGAP35 |
| 6 | 36596654 | 36613339 | protein_coding | NPAS1 |
| 6 | 36696319 | 36765843 | protein_coding | SAE1 |
| 6 | 36884128 | 36908481 | protein_coding | DHX34 |
| 6 | 59768386 | 59817202 | protein_coding | PUM1 |
| 6 | 59775173 | 59775249 | snoRNA | SNORD103 |
| 6 | 59799904 | 59799988 | snoRNA | SNORD103 |
| 6 | 59813178 | 59813262 | snoRNA | SNORD103 |
| 6 | 59860661 | 59869360 | protein_coding | F1STL1_PIG |
| 6 | 60197500 | 60204913 | protein_coding | SNRNP40 |
| 6 | 60283424 | 60283546 | protein_coding | ZCCHC17 |
| 6 | 60435685 | 60445620 | protein_coding | F1SVA1_PIG |
| 6 | 60701999 | 60726958 | protein_coding | SPOCD1 |
| 6 | 60803106 | 60944551 | protein_coding | LCK |
| 6 | 60828960 | 60831341 | protein_coding | IQCC |
| 6 | 60841813 | 60848666 | protein_coding | EIF3I |
| 6 | 60860606 | 60870635 | protein_coding | F1SV89_PIG |
| 6 | 60914156 | 60926352 | protein_coding | TXLNA |
| 6 | 60933562 | 61020268 | protein_coding | CCDC28B |
| 6 | 60987324 | 61025768 | protein_coding | F1SV83_PIG |
| 6 | 61580932 | 61622167 | protein_coding | C1orf94 |
| 6 | 62043511 | 62047903 | protein_coding | DNAJC8 |
| 6 | 62279128 | 62279252 | snoRNA | SNORD22 |
| 6 | 62304808 | 62332390 | protein_coding | EIF2C4 |
| 6 | 62366408 | 62394026 | protein_coding | EIF2C1 |
| 6 | 62425783 | 62451997 | protein_coding | EIF2C3 |
| 6 | 62557601 | 62561852 | protein_coding | TEKT2 |
| 6 | 62562518 | 62568158 | protein_coding | ADPRHL2 |
| 6 | 63569864 | 63583222 | protein_coding | UTP11L |
| 6 | 63789363 | 63792095 | protein_coding | FHL3 |
| 7 | 2295124 | 2311768 | protein_coding | ECI2 |
| 7 | 24782402 | 24919545 | protein_coding | GABBR1 |
| 7 | 24788009 | 24800654 | protein_coding | TRIM31 |
| 7 | 24826735 | 24831445 | protein_coding | RNF39 |
| 7 | 25033268 | 25034201 | pseudogene |  |
| 7 | 25149876 | 25149982 | snRNA | U6 |
| 7 | 25559262 | 25560246 | pseudogene |  |
| 7 | 25812933 | 25813903 | pseudogene |  |
| 7 | 29526372 | 29538307 | protein_coding | BRD2 |
| 7 | 29618573 | 29623114 | protein_coding | SLC39A7 |
| 7 | 29623336 | 29625502 | protein_coding | A5D9P1_PIG |
| 7 | 29626546 | 29626654 | miRNA |  |
| 7 | 29627224 | 29631243 | protein_coding | RING1 |
| 7 | 29721149 | 29844146 | protein_coding | F1CK18_PIG |
| 7 | 42015385 | 42021278 | protein_coding | NCR2 |
| 7 | 42231748 | 42262989 | protein_coding | FOXP4 |
| 7 | 42301210 | 42317353 | protein_coding | MDFI |
| 7 | 42432315 | 42441949 | protein_coding | FRS3 |
| 7 | 42654242 | 42663929 | protein_coding | BYSL |
| 7 | 42788034 | 43043911 | protein_coding | C6orf132 |
| 7 | 42924669 | 42931696 | protein_coding | GUCA1A |
| 7 | 49307564 | 49307670 | snRNA | U6 |
| 7 | 49649644 | 49692739 | protein_coding | CD2AP |
| 7 | 50698989 | 50707004 | protein_coding | GLYATL3 |
| 7 | 50867689 | 50868270 | protein_coding | C6orf141 |
| 7 | 51793918 | 51856199 | protein_coding | TFAP2D |
| 7 | 53111388 | 53111473 | miRNA |  |
| 7 | 53115243 | 53115303 | miRNA |  |
| 7 | 53148682 | 53152259 | protein_coding | B6E279_PIG |
| 7 | 53320437 | 53367073 | protein_coding | MPRB_PIG |
| 7 | 53521458 | 53521563 | snRNA | U6 |
| 7 | 53528844 | 53528952 | snRNA | U6 |
| 7 | 53665590 | 53683767 | protein_coding | WDR61 |
| 7 | 53773239 | 53782420 | protein_coding | ACSBG1 |
| 7 | 53811144 | 53835612 | protein_coding | CIB2 |
| 7 | 53922220 | 53938438 | protein_coding | ADAMTS7 |
| 7 | 54559073 | 54572235 | protein_coding | KIAA1024 |
| 7 | 54972717 | 55039562 | protein_coding | ZFAND6 |
| 7 | 55053120 | 55093313 | protein_coding | FAH |
| 7 | 55310116 | 55451812 | protein_coding | ARNT2 |
| 7 | 55854836 | 55926659 | protein_coding | KIAA1199 |
| 7 | 55969877 | 55971948 | protein_coding | MESDC1 |
| 7 | 56064191 | 56076877 | protein_coding | C15orf26 |
| 7 | 56143279 | 56239320 | protein_coding | IL16 |
| 7 | 56936980 | 56985581 | protein_coding | EFTUD1 |
| 7 | 57711952 | 57722671 | protein_coding | BNC1 |
| 7 | 57922322 | 57969419 | protein_coding | BTBD1 |
| 7 | 58045433 | 58139101 | protein_coding | HOMER2 |
| 7 | 58209987 | 58236433 | protein_coding | FSD2 |
| 7 | 58299532 | 58336612 | protein_coding | AP3B2 |
| 7 | 58347244 | 58423465 | protein_coding | CPEB1 |
| 7 | 58427129 | 58430051 | protein_coding | F1RI94_PIG |
| 7 | 58632413 | 58635336 | protein_coding | F1RI94_PIG |
| 7 | 58919231 | 58953476 | protein_coding | BLM |
| 7 | 59014676 | 59026106 | protein_coding | FES |
| 7 | 59041792 | 59058903 | protein_coding | MAN2A2 |
| 7 | 59072075 | 59086989 | protein_coding | UNC45A |
| 7 | 59088118 | 59097518 | protein_coding | RCCD1 |
| 7 | 59138137 | 59174466 | protein_coding | UROC1 |
| 7 | 59181434 | 59212781 | protein_coding | ZXDC |
| 7 | 59256149 | 59285824 | protein_coding | CCDC37 |
| 7 | 61107888 | 61320540 | protein_coding | F1SJZ8_PIG |
| 7 | 61322219 | 61327168 | protein_coding | SEMA4B |
| 7 | 61394207 | 61395361 | protein_coding | C15orf58 |
| 7 | 61421723 | 61542327 | protein_coding | NRG4 |
| 7 | 61544874 | 61548907 | protein_coding | FBXO22 |
| 7 | 62335310 | 62352710 | protein_coding | RCN2 |
| 7 | 62397796 | 62440268 | protein_coding | PSTPIP1 |
| 7 | 62747561 | 62773842 | protein_coding | HMG20A |
| 7 | 63247819 | 63248373 | protein_coding | IMP3 |
| 7 | 63263657 | 63290647 | protein_coding | SNUPN |
| 7 | 63302924 | 63419418 | protein_coding | PTPN9 |
| 7 | 63452446 | 63501381 | protein_coding | SIN3A |
| 7 | 63504976 | 63517672 | protein_coding | MAN2C1 |
| 7 | 63519768 | 63519842 | miRNA |  |
| 7 | 63600177 | 63606335 | protein_coding | COMMD4 |
| 7 | 63602641 | 63602744 | snRNA | U6 |
| 7 | 63701811 | 63701904 | snRNA | U6 |
| 7 | 63871729 | 63872328 | protein_coding | RPP25 |
| 7 | 63934411 | 64198467 | protein_coding | SCAMP2 |
| 7 | 64200489 | 64207015 | protein_coding | ULK3 |
| 7 | 64329125 | 64393560 | protein_coding | EDC3 |
| 7 | 64613269 | 64626634 | protein_coding | UBL7 |
| 7 | 64750793 | 64773183 | protein_coding | STRA6 |
| 7 | 64878679 | 64886961 | protein_coding | STOML1 |
| 7 | 71790521 | 71816367 | protein_coding | EGLN3 |
| 7 | 73239999 | 73240105 | snRNA | U6 |
| 7 | 74313816 | 74313931 | rRNA | 5S_rRNA |
| 7 | 74443384 | 74558332 | protein_coding | HEATR5A |
| 7 | 74706414 | 74808309 | protein_coding | HECTD1 |
| 7 | 74840427 | 74840728 | misc_RNA | 7SK |
| 7 | 75842547 | 75881221 | protein_coding | PRKD2 |
| 7 | 78238676 | 78238779 | snRNA | U6 |
| 7 | 78272769 | 78272872 | snRNA | U6 |
| 7 | 78363342 | 78363445 | snRNA | U6 |
| 7 | 79250352 | 79284819 | protein_coding | NOVA1 |
| 7 | 80545142 | 80867194 | protein_coding | STXBP6 |
| 7 | 81022391 | 81025806 | protein_coding | F1SGS3_PIG |
| 7 | 81192117 | 81194765 | protein_coding | SDR39U1 |
| 7 | 81206829 | 81209341 | protein_coding | A8S2C3_PIG |
| 7 | 81323833 | 81326724 | protein_coding | B2KSJ1_PIG |
| 7 | 81333076 | 81343098 | protein_coding | DHRS1 |
| 7 | 81362672 | 81368874 | protein_coding | PGTA_PIG |
| 7 | 81397743 | 81400706 | protein_coding | TINF2 |
| 7 | 81410697 | 81423296 | protein_coding | NEDD8 |
| 7 | 81426795 | 81431185 | protein_coding | CHMP4A |
| 7 | 81451210 | 81459574 | protein_coding | IPO4 |
| 7 | 81495365 | 81498701 | protein_coding | Q95292_PIG |
| 7 | 81499990 | 81502663 | protein_coding | FAM158A |
| 7 | 81860348 | 81866536 | protein_coding | JPH4 |
| 7 | 81868597 | 81883284 | protein_coding | AP1G2 |
| 7 | 82044661 | 82045905 | protein_coding | BCL2L2 |
| 7 | 82057753 | 82061913 | protein_coding | PABPN1 |
| 7 | 82104640 | 82107317 | protein_coding | CMTM5 |
| 7 | 82187904 | 82188009 | snRNA | U6 |
| 7 | 82233276 | 82298502 | protein_coding | SLC7A8 |
| 7 | 82358403 | 82358828 | protein_coding | C14orf119 |
| 7 | 82432218 | 82469416 | protein_coding | A8HG48_PIG |
| 7 | 82597008 | 82597763 | retrotransposed | |
| 7 | 82719922 | 82734065 | protein_coding | ABHD4 |
| 7 | 83941921 | 83942875 | pseudogene |  |
| 7 | 84237840 | 84237949 | snoRNA | snoU6-53 |
| 7 | 84242610 | 84242721 | snoRNA | snoU6-53 |
| 7 | 84620470 | 84620579 | snoRNA | snoU6-53 |
| 7 | 84625248 | 84625359 | snoRNA | snoU6-53 |
| 7 | 84782422 | 84782782 | pseudogene |  |
| 7 | 84885611 | 84889464 | protein_coding | TMEM55B |
| 7 | 84892936 | 84900518 | protein_coding | OSGEP |
| 7 | 84914657 | 84916479 | protein_coding | KLHL33 |
| 7 | 84953173 | 84968950 | protein_coding | TEP1 |
| 7 | 84990293 | 84990620 | misc_RNA | RNaseP_nuc |
| 7 | 85002136 | 85002284 | snoRNA | SNORA79 |
| 7 | 85008453 | 85013405 | protein_coding | CCNB1IP1 |
| 7 | 85017072 | 85037070 | protein_coding | TTC5 |
| 7 | 85130207 | 85144526 | protein_coding | PARP2 |
| 7 | 86406551 | 86407488 | pseudogene |  |
| 7 | 86990827 | 86991764 | pseudogene |  |
| 7 | 87714603 | 87714706 | snoRNA | snoU13 |
| 7 | 88011136 | 88037300 | protein_coding | SLC12A6 |
| 7 | 88062025 | 88122264 | protein_coding | C7H15orf29 |
| 7 | 88195236 | 88229559 | protein_coding | AVEN |
| 7 | 95127584 | 95461543 | protein_coding | SLCO3A1 |
| 7 | 95755715 | 95787915 | protein_coding | ST8SIA2 |
| 7 | 99493545 | 99516098 | protein_coding | EIF2S1 |
| 7 | 99678445 | 99710443 | protein_coding | PLEKHH1 |
| 7 | 99998793 | 100042480 | protein_coding | RAD51B |
| 7 | 100916496 | 100960471 | protein_coding | ACTN1 |
| 7 | 101283646 | 101302416 | protein_coding | EXD2 |
| 7 | 118178168 | 118232788 | protein_coding | SPATA7 |
| 7 | 118332378 | 118379210 | protein_coding | ZC3H14 |
| 7 | 118605713 | 118657306 | protein_coding | TTC8 |
| 7 | 118713580 | 118713907 | misc_RNA | 7SK |
| 7 | 118721119 | 118721218 | snRNA | U6 |
| 7 | 119621566 | 119707343 | protein_coding | TDP1 |
| 7 | 122379060 | 122412679 | protein_coding | GOLGA5 |
| 7 | 122483936 | 122496243 | protein_coding | CHGA |
| 7 | 122793174 | 122794882 | protein_coding | CN109_PIG |
| 7 | 122809305 | 122826265 | protein_coding | UBR7 |
| 7 | 122946116 | 122947299 | protein_coding | COX8C |
| 7 | 123234135 | 123348801 | protein_coding | UNC79 |
| 7 | 123499063 | 123520224 | protein_coding | ASB2 |
| 7 | 123639810 | 123658207 | protein_coding | OTUB2 |
| 7 | 123691798 | 123696840 | protein_coding | Q6IEA3_PIG |
| 7 | 128265338 | 128265422 | miRNA |  |
| 7 | 129353743 | 129353824 | miRNA |  |
| 7 | 129422138 | 129422236 | snRNA | U6 |
| 7 | 130435919 | 130436008 | miRNA |  |
| 7 | 130745569 | 130753746 | protein_coding | TNFAIP2 |
| 7 | 131302725 | 131303687 | protein_coding | ANKRD9 |
| 7 | 131441195 | 131441446 | protein_coding | F1SA07_PIG |
| 7 | 131456242 | 131587586 | protein_coding | CINP |
| 7 | 131508878 | 131681515 | protein_coding | MOK |
| 7 | 131781004 | 131781347 | pseudogene |  |
| 7 | 131807953 | 131809819 | protein_coding | HSP90AA1 |
| 7 | 131853872 | 131889927 | protein_coding | DYNC1H1 |
| 7 | 132767188 | 132788195 | protein_coding | PPP1R13B |
| 7 | 132799815 | 132807086 | protein_coding | XRCC3 |
| 7 | 133006420 | 133086612 | protein_coding | TDRD9 |
| 7 | 133126052 | 133133061 | protein_coding | PLD4 |
| 7 | 133166385 | 133222232 | protein_coding | C14orf79 |
| 7 | 133194262 | 133214665 | protein_coding | KIAA0284 |
| 8 | 72934 | 332448 | protein_coding | IGF2-AS |
| 8 | 81119 | 82980 | protein_coding | MFSD7 |
| 8 | 168043 | 178763 | protein_coding | SLBP |
| 8 | 206623 | 211094 | protein_coding | TMEM129 |
| 8 | 324513 | 331029 | protein_coding | WHSC2 |
| 8 | 394877 | 399289 | protein_coding | NAT8L |
| 8 | 752967 | 762300 | protein_coding | RNF4 |
| 8 | 919341 | 923975 | protein_coding | TNIP2 |
| 8 | 959244 | 977790 | protein_coding | ADD1 |
| 8 | 1040237 | 1115691 | protein_coding | GRK4 |
| 8 | 1164042 | 1436474 | protein_coding | HDH |
| 8 | 1555101 | 1567527 | protein_coding | DOK7 |
| 8 | 1721296 | 1722357 | pseudogene |  |
| 8 | 1878818 | 1878955 | protein_coding | EVC2 |
| 8 | 2339427 | 2340959 | protein_coding | WFS1 |
| 8 | 7889160 | 7905206 | protein_coding | B8XTR5_PIG |
| 8 | 8304699 | 8305412 | protein_coding | FGFBP1 |
| 8 | 8460721 | 8460925 | pseudogene |  |
| 8 | 9724158 | 9765343 | protein_coding | NCAPG |
| 8 | 11826140 | 12034965 | protein_coding | SLIT2 |
| 8 | 11840680 | 11840789 | miRNA |  |
| 8 | 27958847 | 28012412 | protein_coding | B5U335_PIG |
| 8 | 28568243 | 28568519 | misc_RNA | 7SK |
| 8 | 28569096 | 28681663 | protein_coding | GRXCR1 |
| 8 | 46421930 | 46421998 | snoRNA | snR39B |
| 8 | 46564763 | 46564847 | miRNA |  |
| 8 | 46708001 | 46723173 | protein_coding | REST |
| 8 | 46766993 | 46767435 | pseudogene |  |
| 8 | 46997795 | 47003296 | protein_coding | HOP_PIG |
| 8 | 47171356 | 47217595 | protein_coding | F1RTV4_PIG |
| 8 | 47339179 | 47347656 | protein_coding | AASDH |
| 8 | 47591109 | 47591209 | snRNA | U6 |
| 8 | 47618086 | 47692809 | protein_coding | NMU_PIG |
| 8 | 47695056 | 47727197 | protein_coding | PDCL2 |
| 8 | 47922112 | 47932289 | protein_coding | CLOCK |
| 8 | 59211677 | 59212019 | misc_RNA | 7SK |
| 8 | 59305978 | 59309197 | protein_coding | IL8_PIG |
| 8 | 59528574 | 59530500 | protein_coding | CXCL2 |
| 8 | 59550947 | 59595287 | protein_coding | MTHFD2L |
| 8 | 59653735 | 59668303 | protein_coding | EREG |
| 8 | 61420722 | 61421504 | protein_coding | STBD1 |
| 8 | 63917092 | 63917360 | misc_RNA | 7SK |
| 8 | 63998580 | 64007824 | protein_coding | FGB |
| 8 | 64037168 | 64037230 | snRNA | U6 |
| 8 | 111523763 | 111524079 | pseudogene |  |
| 8 | 111724020 | 111726347 | protein_coding | GPRIN3 |
| 8 | 111994156 | 112016343 | protein_coding | FAM13A |
| 8 | 112057551 | 112058138 | protein_coding | F1RW65_PIG |
| 8 | 112296288 | 112514103 | protein_coding | Q6QAS3_PIG |
| 8 | 112884827 | 112929202 | protein_coding | SPARCL1 |
| 8 | 113121323 | 113144966 | protein_coding | HSD17B13 |
| 8 | 113837962 | 113973958 | protein_coding | MAPK10 |
| 8 | 114890629 | 115137885 | protein_coding | WDFY3 |
| 8 | 116120483 | 116136247 | protein_coding | FAM175A |
| 8 | 116144462 | 116179090 | protein_coding | HELQ |
| 8 | 116198637 | 116235950 | protein_coding | HPSE |
| 8 | 116245643 | 116265218 | protein_coding | COQ2 |
| 8 | 116364509 | 116392802 | protein_coding | PLAC8 |
| 8 | 116445134 | 116445209 | miRNA |  |
| 8 | 116475182 | 116538563 | protein_coding | LIN54 |
| 8 | 116586687 | 116636903 | protein_coding | SEC31A |
| 8 | 116649525 | 116752791 | protein_coding | F1RVD3_PIG |
| 8 | 116864757 | 116869761 | protein_coding | HNRPDL |
| 8 | 116910628 | 116915701 | protein_coding | HNRNPD |
| 9 | 268151 | 268690 | protein_coding | ASCL3 |
| 9 | 399189 | 442728 | protein_coding | SCUBE2 |
| 9 | 678798 | 703168 | protein_coding | TMEM41B |
| 9 | 776735 | 821582 | protein_coding | RIC3 |
| 9 | 1799065 | 1811317 | protein_coding | ZNF215 |
| 9 | 2151364 | 2152307 | pseudogene |  |
| 9 | 2254382 | 2254487 | snRNA | U6 |
| 9 | 2561209 | 2564779 | protein_coding | ARFIP2 |
| 9 | 2575350 | 2590957 | protein_coding | TRIM3 |
| 9 | 2597412 | 2606196 | protein_coding | HPX |
| 9 | 2612617 | 2614989 | protein_coding | SMPD1 |
| 9 | 2703568 | 2708959 | protein_coding | CNGA4 |
| 9 | 2732679 | 2743001 | protein_coding | FAM160A2 |
| 9 | 2822586 | 2827636 | protein_coding | C11orf42 |
| 9 | 2855596 | 2856408 | pseudogene |  |
| 9 | 2892750 | 2893700 | protein_coding | OR56A1 |
| 9 | 3490297 | 3491232 | pseudogene |  |
| 9 | 3533358 | 3535199 | protein_coding | UBQLNL |
| 9 | 3537888 | 3539846 | protein_coding | UBQLN3 |
| 9 | 3732032 | 3732974 | pseudogene |  |
| 9 | 4384816 | 4385756 | pseudogene |  |
| 9 | 4465287 | 4466255 | protein_coding | OR51S1 |
| 9 | 4609189 | 4610142 | protein_coding | OR51M1 |
| 9 | 4662264 | 4669953 | protein_coding | TRIM68 |
| 9 | 4873189 | 4873481 | pseudogene |  |
| 9 | 5122888 | 5130672 | protein_coding | TRIM21 |
| 9 | 5682301 | 5824335 | protein_coding | NUP98 |
| 9 | 5851938 | 5856786 | protein_coding | ART5 |
| 9 | 6069351 | 6108112 | protein_coding | LRTOMT |
| 9 | 6250720 | 6264875 | protein_coding | FOLR1 |
| 9 | 6284369 | 6284475 | snRNA | U6 |
| 9 | 37246242 | 37286547 | protein_coding | RDX |
| 9 | 37456102 | 37493239 | protein_coding | ARHGAP20 |
| 9 | 38034147 | 38034355 | pseudogene |  |
| 9 | 38085689 | 38090173 | protein_coding | C11orf93 |
| 9 | 38210599 | 38210682 | miRNA |  |
| 9 | 38211158 | 38211234 | miRNA |  |
| 9 | 44791000 | 44792913 | protein_coding | UPK2 |
| 9 | 44799463 | 44810577 | protein_coding | FOXR1 |
| 9 | 44820879 | 44830008 | protein_coding | CCDC84 |
| 9 | 44832756 | 44836873 | protein_coding | TRAPPC4 |
| 9 | 44878915 | 44887550 | protein_coding | A0SNU8_PIG |
| 9 | 44934537 | 44943438 | protein_coding | C2CD2L |
| 9 | 44948711 | 44959738 | protein_coding | HINFP |
| 9 | 44999418 | 45012768 | protein_coding | NLRX1 |
| 9 | 45017359 | 45118067 | protein_coding | CBL |
| 9 | 45155745 | 45157046 | protein_coding | RNF26 |
| 9 | 79533339 | 79533851 | protein_coding | Q95JA6_PIG |
| 9 | 80385487 | 80426599 | protein_coding | LRRC72 |
| 9 | 80471534 | 80479275 | protein_coding | TSPAN13 |
| 9 | 81249109 | 81249199 | miRNA |  |
| 9 | 82730209 | 82730954 | protein_coding | TWIST1 |
| 9 | 82860496 | 82860990 | protein_coding | FERD3L |
| 9 | 83375291 | 83484643 | protein_coding | TWISTNB |
| 9 | 84678801 | 84945458 | protein_coding | DNAH11 |
| 9 | 85764702 | 85764775 | snoRNA | SNORD93 |
| 9 | 85801970 | 85806347 | protein_coding | A4D862_PIG |
| 9 | 87033401 | 87033507 | snRNA | U6 |
| 9 | 87074539 | 87097481 | protein_coding | C7orf23 |
| 9 | 87316672 | 87402750 | protein_coding | KIAA1324L |
| 9 | 88927566 | 89085644 | protein_coding | SEMA3D |
| 9 | 89599310 | 89750785 | protein_coding | SEMA3A |
| 9 | 90294078 | 90375999 | protein_coding | SEMA3E |
| 9 | 90684965 | 91078418 | protein_coding | PCLO |
| 9 | 91463880 | 91464156 | misc_RNA | 7SK |
| 10 | 26696160 | 26859821 | protein_coding | F1S4K0_PIG |
| 10 | 30134896 | 30250964 | protein_coding | FRMD3 |
| 11 | 8582224 | 8907912 | protein_coding | PDS5B |
| 11 | 8628328 | 8647966 | protein_coding | N4BP2L2 |
| 11 | 16445682 | 16535071 | protein_coding | INTS6 |
| 11 | 16679342 | 16729532 | protein_coding | FAM124A |
| 11 | 20509594 | 20603421 | protein_coding | LCP1 |
| 11 | 20888843 | 20889145 | misc_RNA | 7SK |
| 11 | 20941987 | 20942386 | protein_coding | FAM194B |
| 11 | 21135666 | 21170899 | protein_coding | NUFIP1 |
| 11 | 21398956 | 21510318 | protein_coding | TSC22D1 |
| 11 | 21600874 | 21629114 | protein_coding | SERP2 |
| 11 | 22289352 | 22479921 | protein_coding | CCDC122 |
| 11 | 23177327 | 23185533 | protein_coding | C13orf30 |
| 11 | 24548524 | 24548815 | misc_RNA | 7SK |
| 11 | 24578630 | 24613800 | protein_coding | MTRF1 |
| 11 | 24619554 | 24621596 | protein_coding | KBTBD7 |
| 11 | 24693865 | 24695952 | protein_coding | KBTBD6 |
| 11 | 27151836 | 27155805 | protein_coding | PCDH8 |
| 11 | 32057724 | 32161615 | protein_coding | TDRD3 |
| 11 | 32683059 | 32683301 | protein_coding | CIRH1A |
| 11 | 33014166 | 33014298 | snoRNA | SNORA31 |
| 11 | 37331801 | 37331907 | snRNA | U6 |
| 11 | 43555014 | 43555095 | snRNA | U6 |
| 11 | 44217359 | 44233478 | protein_coding | MZT1 |
| 11 | 44268878 | 44281099 | protein_coding | DIS3 |
| 11 | 44338871 | 44452351 | protein_coding | PIBF1 |
| 11 | 44387713 | 44387851 | snoRNA | SNORA70 |
| 11 | 44596280 | 44731528 | protein_coding | KLF5 |
| 11 | 46685207 | 46685314 | snRNA | U6 |
| 11 | 46768886 | 46768992 | snRNA | U6 |
| 11 | 46784189 | 46822736 | protein_coding | UCHL3 |
| 11 | 46983287 | 47022830 | protein_coding | LMO7 |
| 11 | 48023540 | 48212816 | protein_coding | IRG1 |
| 11 | 48051903 | 48072745 | protein_coding | CLN5 |
| 11 | 48596276 | 48660061 | protein_coding | SCEL |
| 11 | 48695576 | 48695888 | misc_RNA | 7SK |
| 11 | 49510114 | 49510215 | snRNA | U6 |
| 11 | 50776070 | 50776154 | miRNA |  |
| 11 | 51842157 | 51842290 | snoRNA | SNORA70 |
| 11 | 51988866 | 51988999 | snoRNA | SNORA70 |
| 12 | 362061 | 369417 | protein_coding | FN3KRP |
| 12 | 527340 | 554187 | protein_coding | TBCD |
| 12 | 557531 | 565425 | protein_coding | METRNL |
| 12 | 715061 | 718654 | protein_coding | CHMP6 |
| 12 | 778874 | 783621 | protein_coding | AATK |
| 12 | 857667 | 860904 | protein_coding | AZI1 |
| 12 | 1005085 | 1011049 | protein_coding | NPTX1 |
| 12 | 1208159 | 1228628 | protein_coding | SLC26A11 |
| 12 | 1285295 | 1311016 | protein_coding | F1RZ83_PIG |
| 12 | 1364313 | 1384348 | protein_coding | SLC26A11 |
| 12 | 1511910 | 1565945 | protein_coding | TBC1D16 |
| 12 | 1873679 | 1917802 | protein_coding | C0JPM4_PIG |
| 12 | 1933672 | 1965370 | protein_coding | F1RZ74_PIG |
| 12 | 1975893 | 2044159 | protein_coding | CYTH1 |
| 12 | 2779655 | 2779731 | snRNA | U6 |
| 12 | 2880672 | 2880859 | snoRNA | SCARNA16 |
| 12 | 2923982 | 2960376 | protein_coding | MGAT5B |
| 12 | 3157987 | 3161180 | protein_coding | ST6GALNAC1 |
| 12 | 3263364 | 3264545 | protein_coding | AANAT |
| 12 | 3271777 | 3271854 | snoRNA | snoR38 |
| 12 | 3273466 | 3273548 | snoRNA | snoR38 |
| 12 | 3274049 | 3274120 | snoRNA | snoR38 |
| 12 | 3503944 | 3533869 | protein_coding | PRPSAP1 |
| 12 | 3584864 | 3653790 | protein_coding | RNF157 |
| 12 | 3701028 | 3721735 | protein_coding | EXOC7 |
| 12 | 3726699 | 3760988 | protein_coding | SRP68 |
| 12 | 3768167 | 3784729 | protein_coding | EVPL |
| 12 | 3896541 | 3900635 | protein_coding | TRIM47 |
| 12 | 3925666 | 3940296 | protein_coding | UNC13D |
| 12 | 3983032 | 3986455 | protein_coding | H33_PIG |
| 12 | 4121930 | 4190624 | protein_coding | B6E241_PIG |
| 12 | 4214992 | 4226772 | protein_coding | C8C419_PIG |
| 12 | 4229002 | 4233542 | protein_coding | MIF4GD |
| 12 | 4237067 | 4252581 | protein_coding | GGA3 |
| 12 | 4297804 | 4311546 | protein_coding | SUMO2_PIG |
| 12 | 4320628 | 4339131 | protein_coding | HN1 |
| 12 | 4446958 | 4465855 | protein_coding | C17orf28 |
| 12 | 4487621 | 4489266 | protein_coding | NT5C |
| 12 | 4533960 | 4562906 | protein_coding | USH1G |
| 12 | 4577962 | 4591031 | protein_coding | FADS6 |
| 12 | 4596602 | 4607144 | protein_coding | FDXR |
| 12 | 4619001 | 4623731 | protein_coding | GRIN2C |
| 12 | 4683111 | 4687325 | protein_coding | NAT9 |
| 12 | 4724324 | 4743698 | protein_coding | CD300LF |
| 12 | 4859156 | 4867151 | protein_coding | CD300LB |
| 12 | 5527833 | 5644640 | protein_coding | SDK2 |
| 12 | 5769195 | 5770268 | protein_coding | CDC42EP4 |
| 12 | 5839731 | 5857212 | protein_coding | FAM104A |
| 12 | 5949691 | 6369897 | protein_coding | SLC39A11 |
| 12 | 9477044 | 9477150 | snRNA | U6 |
| 12 | 9577504 | 9619013 | protein_coding | ABCA6 |
| 12 | 9924353 | 9938270 | protein_coding | FAM20A |
| 12 | 9995973 | 10032669 | protein_coding | D7RA30_PIG |
| 12 | 10201158 | 10240160 | protein_coding | GNA13 |
| 12 | 11336894 | 11343225 | protein_coding | CACNG5 |
| 12 | 11407711 | 11468596 | protein_coding | CACNG4 |
| 12 | 11479591 | 11490680 | protein_coding | CCG1_PIG |
| 12 | 11663406 | 11663513 | snRNA | U6 |
| 12 | 11749168 | 12020183 | protein_coding | PITPNC1 |
| 12 | 12044442 | 12066913 | protein_coding | NOL11 |
| 12 | 12236539 | 12292016 | protein_coding | PECA1_PIG |
| 12 | 12375975 | 12435179 | protein_coding | TEX2 |
| 12 | 12476768 | 12508945 | protein_coding | ERN1 |
| 12 | 12521729 | 12540776 | protein_coding | ICAM-2 |
| 12 | 12626857 | 12630260 | protein_coding | CD79B |
| 12 | 12638816 | 12640578 | protein_coding | D6BJT5_PIG |
| 12 | 12665578 | 12674428 | protein_coding | SMARCD2 |
| 12 | 12775235 | 12801493 | protein_coding | STRADA |
| 12 | 12806315 | 12807617 | protein_coding | LIMD2 |
| 12 | 13021772 | 13031579 | protein_coding | CYB561 |
| 12 | 13460285 | 13705158 | protein_coding | 10-Mar |
| 12 | 13885420 | 13885747 | pseudogene |  |
| 12 | 14557060 | 14672909 | protein_coding | CDC27 |
| 12 | 14826946 | 14969812 | protein_coding | KIAA1267 |
| 12 | 15222412 | 15275558 | protein_coding | F1RRS6_PIG |
| 12 | 15917430 | 15917696 | pseudogene |  |
| 12 | 15936753 | 15942869 | protein_coding | ARHGAP27 |
| 12 | 16056443 | 16059333 | protein_coding | C17orf46 |
| 12 | 16148233 | 16168914 | protein_coding | PLCD3 |
| 12 | 16272483 | 16279004 | protein_coding | C1QL1 |
| 12 | 16336840 | 16377246 | protein_coding | EFTUD2 |
| 12 | 16407255 | 16408586 | protein_coding | CXG1_PIG |
| 12 | 16489508 | 16490839 | protein_coding | CXG1_PIG |
| 12 | 16563922 | 16564028 | snRNA | U6 |
| 12 | 16567218 | 16661365 | protein_coding | C17orf104 |
| 12 | 16786785 | 16788311 | protein_coding | GPATCH8 |
| 12 | 16808514 | 16812389 | protein_coding | GRN |
| 12 | 16909566 | 16918923 | protein_coding | RUNDC3A |
| 12 | 16936904 | 16948100 | protein_coding | UBTF |
| 12 | 16959701 | 16959811 | protein_coding | TMUB2 |
| 12 | 17013175 | 17014427 | protein_coding | PYY_PIG |
| 12 | 17035625 | 17039969 | protein_coding | G6PC3 |
| 12 | 17176856 | 17181061 | protein_coding | NAGS |
| 12 | 17209085 | 17234253 | protein_coding | MPP3 |
| 12 | 17248235 | 17260637 | protein_coding | DUSP3 |
| 12 | 17267138 | 17270726 | protein_coding | SOST |
| 12 | 17343490 | 17344088 | protein_coding | MEOX1 |
| 12 | 17447100 | 17477589 | protein_coding | DHX8 |
| 12 | 17574940 | 17581102 | protein_coding | CNTNAP1 |
| 12 | 17642587 | 17654626 | protein_coding | BECN1 |
| 12 | 17663493 | 17664611 | protein_coding | CCDC56 |
| 12 | 17777665 | 17798308 | protein_coding | FAM134C |
| 12 | 17801019 | 17806889 | protein_coding | PSMC3IP |
| 12 | 17923290 | 17946321 | protein_coding | E0AD96_PIG |
| 12 | 18153996 | 18182276 | protein_coding | STAT5B |
| 12 | 18320991 | 18322387 | protein_coding | OREX_PIG |
| 12 | 18325882 | 18342708 | protein_coding | KCNH4 |
| 12 | 18370962 | 18379665 | protein_coding | KAT2A |
| 12 | 18380106 | 18388791 | protein_coding | DHX58 |
| 12 | 18458466 | 18488732 | protein_coding | DNAJC7 |
| 12 | 18557550 | 18601933 | protein_coding | ACL |
| 12 | 18603034 | 18610457 | protein_coding | KLHL11 |
| 12 | 18622989 | 18631118 | protein_coding | NT5C3L |
| 12 | 18646045 | 18648277 | protein_coding | F1S0K9_PIG |
| 12 | 18738941 | 18741519 | protein_coding | EIF1 |
| 12 | 18862215 | 18864446 | protein_coding | F1S0K9_PIG |
| 12 | 19392090 | 19401272 | protein_coding | K1C20_PIG |
| 12 | 19464951 | 19475221 | protein_coding | KRT28 |
| 12 | 19482935 | 19488062 | protein_coding | KRT27 |
| 12 | 19494970 | 19501621 | protein_coding | KRT26 |
| 12 | 19509204 | 19515860 | protein_coding | KRT25 |
| 12 | 19577558 | 19589498 | protein_coding | Q861S1_PIG |
| 12 | 19608048 | 19621464 | protein_coding | SMARCE1 |
| 12 | 19744986 | 19781793 | protein_coding | TNS4 |
| 12 | 19750959 | 19764757 | protein_coding | CDC6 |
| 12 | 19831692 | 19858974 | protein_coding | TOP2A_PIG |
| 12 | 19871215 | 20049785 | protein_coding | F1RXD0_PIG |
| 12 | 19935124 | 19944988 | protein_coding | MSL1 |
| 12 | 19950609 | 19970663 | protein_coding | CASC3 |
| 12 | 19975310 | 19988087 | protein_coding | RAPGEFL1 |
| 12 | 20006236 | 20047678 | protein_coding | WIPF2 |
| 12 | 20066410 | 20115048 | protein_coding | F1RXC4_PIG |
| 12 | 20149317 | 20151340 | protein_coding | CSF3_PIG |
| 12 | 20259192 | 20259269 | miRNA |  |
| 12 | 20287488 | 20287591 | snoRNA | SNORD124 |
| 12 | 20429240 | 20521874 | protein_coding | IKZF3 |
| 12 | 20553936 | 20555633 | protein_coding | MIEN1 |
| 12 | 20579679 | 20587050 | protein_coding | PPP1R1B |
| 12 | 20587727 | 20613581 | protein_coding | STARD3 |
| 12 | 20615599 | 20616848 | protein_coding | TCAP |
| 12 | 20618539 | 20620121 | protein_coding | PNMT_PIG |
| 12 | 20672273 | 20673418 | pseudogene |  |
| 12 | 20802497 | 20902249 | protein_coding | MED1 |
| 12 | 21054363 | 21060781 | protein_coding | STAC2 |
| 12 | 21078286 | 21099106 | protein_coding | CACNB1 |
| 12 | 21113802 | 21174151 | protein_coding | PLXDC1 |
| 12 | 21306343 | 21392895 | protein_coding | NPEPPS |
| 12 | 21418408 | 21445001 | protein_coding | KPNB1 |
| 12 | 21457362 | 21472440 | protein_coding | TBKBP1 |
| 12 | 21485201 | 21661479 | protein_coding | SP2 |
| 12 | 21499519 | 21511736 | protein_coding | TBX21 |
| 12 | 21593394 | 21599806 | protein_coding | LRRC46 |
| 12 | 21743207 | 21743292 | miRNA |  |
| 12 | 21743509 | 21754904 | protein_coding | COPZ2 |
| 12 | 21831008 | 21883082 | protein_coding | SKAP1 |
| 12 | 21912862 | 21939181 | protein_coding | CBX1 |
| 12 | 22434918 | 22435019 | miRNA |  |
| 12 | 22603567 | 22633877 | protein_coding | CALCOCO2 |
| 12 | 22662246 | 22662479 | snoRNA | snoU89 |
| 12 | 22664940 | 22667646 | protein_coding | F1RWF6_PIG |
| 12 | 22684979 | 22685108 | snoRNA | SNORA11 |
| 12 | 22922300 | 22932267 | protein_coding | SPOP |
| 12 | 22987595 | 23005905 | protein_coding | NGFR |
| 12 | 23394660 | 23427719 | protein_coding | MYST2 |
| 12 | 23813841 | 23829053 | protein_coding | LUC7L3 |
| 12 | 23879855 | 23884702 | protein_coding | WFIKKN2 |
| 12 | 23976973 | 23992345 | protein_coding | ANKRD40 |
| 12 | 24135232 | 24155023 | protein_coding | MYCBPAP |
| 12 | 24160771 | 24166981 | protein_coding | EPN3 |
| 12 | 24171778 | 24181644 | protein_coding | SPATA20 |
| 12 | 24210110 | 24211162 | protein_coding | ACSF2 |
| 12 | 24214235 | 24222582 | protein_coding | RSAD1 |
| 12 | 24292491 | 24307183 | protein_coding | LRRC59 |
| 12 | 24316379 | 24322636 | protein_coding | MRPL27 |
| 12 | 24759158 | 24764661 | protein_coding | SAMD14 |
| 12 | 24858243 | 24858349 | snRNA | U6 |
| 12 | 25059937 | 25089119 | protein_coding | NDKB_PIG |
| 12 | 25170730 | 25209079 | protein_coding | UTP18 |
| 12 | 28744823 | 28760307 | protein_coding | TOM1L1 |
| 12 | 29242356 | 29269303 | protein_coding | MMD |
| 12 | 30352923 | 30616668 | protein_coding | ANKFN1 |
| 12 | 30727119 | 30728003 | protein_coding | B8XVN4_PIG |
| 12 | 30731742 | 30731860 | rRNA | 5S_rRNA |
| 12 | 30970298 | 30981372 | protein_coding | COIL |
| 12 | 31001929 | 31007597 | protein_coding | TRIM25 |
| 12 | 31010743 | 31036581 | protein_coding | DGKE |
| 12 | 31150066 | 31150975 | pseudogene |  |
| 12 | 31313144 | 31548463 | protein_coding | MSI2 |
| 12 | 31625448 | 31625557 | rRNA | 5S_rRNA |
| 12 | 31821607 | 31831017 | protein_coding | VEZF1 |
| 12 | 32016578 | 32018530 | protein_coding | SRSF1_PIG |
| 12 | 32308701 | 32313938 | protein_coding | DYNLL2 |
| 12 | 32515991 | 32574585 | protein_coding | LPO |
| 12 | 32981853 | 33014598 | protein_coding | RAD51C |
| 12 | 33049038 | 33049201 | snRNA | U1 |
| 12 | 33058276 | 33058439 | snRNA | U1 |
| 12 | 33059376 | 33059539 | snRNA | U1 |
| 12 | 33062609 | 33062772 | snRNA | U1 |
| 12 | 33068688 | 33068840 | snRNA | U1 |
| 12 | 33069794 | 33069957 | snRNA | U1 |
| 12 | 33081674 | 33081888 | snoRNA | U3 |
| 12 | 33085443 | 33085657 | snoRNA | U3 |
| 12 | 33090841 | 33091004 | snRNA | U1 |
| 12 | 33272112 | 33295911 | protein_coding | PPM1E |
| 12 | 33527929 | 33528035 | snRNA | U6 |
| 12 | 33579056 | 33580010 | pseudogene |  |
| 12 | 33917589 | 33963642 | protein_coding | DHX40 |
| 12 | 33985185 | 34054684 | protein_coding | C0MHR2_PIG |
| 12 | 34069469 | 34200586 | protein_coding | VMP1 |
| 12 | 34201565 | 34201656 | miRNA | ssc-mir-21 |
| 12 | 35277764 | 35322510 | protein_coding | BCAS3 |
| 12 | 35623636 | 35683948 | protein_coding | APPBP2 |
| 12 | 35660135 | 35660258 | snoRNA | SNORA17 |
| 12 | 35966771 | 35966898 | snoRNA | SNORA11 |
| 12 | 36018428 | 36098860 | protein_coding | F1S201_PIG |
| 12 | 36181764 | 36247051 | protein_coding | GGNBP2 |
| 12 | 36214783 | 36214910 | snoRNA | SNORA11 |
| 12 | 36259817 | 36265133 | protein_coding | MRM1 |
| 12 | 36781687 | 36883430 | protein_coding | AATF |
| 12 | 36928552 | 36928658 | snRNA | U6 |
| 13 | 22891589 | 22939546 | protein_coding | Abhd5 |
| 13 | 23336890 | 23336951 | miRNA |  |
| 13 | 23480639 | 23512665 | protein_coding | CDCP1 |
| 13 | 23596004 | 23655601 | protein_coding | ZDHHC3 |
| 13 | 23690741 | 23694407 | protein_coding | TMEM42 |
| 13 | 23829804 | 23832119 | protein_coding | ZNF197 |
| 13 | 23840256 | 23846305 | protein_coding | ZNF35 |
| 13 | 23875476 | 23877044 | protein_coding | ZNF502 |
| 13 | 23888001 | 23888813 | protein_coding | ZNF501 |
| 13 | 23973543 | 23974519 | pseudogene |  |
| 13 | 23989788 | 23993322 | protein_coding | ZNF445 |
| 13 | 24189843 | 24289441 | protein_coding | LARS2 |
| 13 | 24444364 | 24462037 | protein_coding | Q6YT47_PIG |
| 13 | 24506685 | 24511451 | protein_coding | Q6YT44_PIG |
| 13 | 24757645 | 24783308 | protein_coding | PTH1R_PIG |
| 13 | 24884468 | 25042580 | protein_coding | NBEAL2 |
| 13 | 54816916 | 55014949 | protein_coding | SETD5 |
| 13 | 54924763 | 54945977 | protein_coding | THUMPD3 |
| 13 | 55142055 | 55188251 | protein_coding | MTMR14 |
| 13 | 55213950 | 55227458 | protein_coding | BRPF1 |
| 13 | 55229424 | 55235330 | protein_coding | OGG1 |
| 13 | 55270515 | 55279072 | protein_coding | ARPC4 |
| 13 | 55321269 | 55364966 | protein_coding | IL17RE |
| 13 | 55342148 | 55344699 | protein_coding | JAGN1 |
| 13 | 55366120 | 55381913 | protein_coding | IL17RC |
| 13 | 55382148 | 55390735 | protein_coding | CRELD1 |
| 13 | 55418189 | 55492273 | protein_coding | FANCD2 |
| 13 | 55441839 | 55441942 | snRNA | U6 |
| 13 | 55499318 | 55508146 | protein_coding | BRK1 |
| 13 | 55518347 | 55524222 | protein_coding | VHL |
| 13 | 55605987 | 55610508 | protein_coding | GHRL |
| 13 | 55724725 | 55732990 | protein_coding | SEC13 |
| 13 | 56032507 | 56080590 | protein_coding | ATG7 |
| 13 | 56263117 | 56280465 | protein_coding | F1SQA4_PIG |
| 13 | 59196108 | 59279101 | protein_coding | C3orf37 |
| 13 | 59216637 | 59233111 | protein_coding | RPN1 |
| 13 | 59265857 | 59266002 | pseudogene |  |
| 13 | 59417915 | 59418610 | protein_coding | DNAJB8 |
| 13 | 59574621 | 59650012 | protein_coding | EEFSEC |
| 13 | 59739363 | 59777961 | protein_coding | RUVBL1 |
| 13 | 60084220 | 60122864 | protein_coding | MCM2 |
| 13 | 60190890 | 60207100 | protein_coding | CHST13 |
| 13 | 60289078 | 60325135 | protein_coding | ACPP |
| 13 | 60410825 | 60519221 | protein_coding | DNAJC13 |
| 13 | 60583954 | 60589111 | protein_coding | A3F9C6_PIG |
| 13 | 60643343 | 60792925 | protein_coding | UBA5 |
| 13 | 61191406 | 61392774 | protein_coding | TMEM108 |
| 13 | 61360915 | 61373350 | protein_coding | CDV3 |
| 13 | 61412890 | 61412999 | misc_RNA | Y_RNA |
| 13 | 62050889 | 62055013 | protein_coding | ANAPC13 |
| 13 | 62080184 | 62113698 | protein_coding | CEP63 |
| 13 | 62448832 | 62468599 | protein_coding | EPHB1 |
| 13 | 63080884 | 63260780 | protein_coding | PPP2R3A |
| 13 | 63113091 | 63113160 | snoRNA | SNORD112 |
| 13 | 63938123 | 63938227 | protein_coding | IL20RB |
| 13 | 64925838 | 64956102 | protein_coding | ARMC8 |
| 13 | 65314459 | 65334402 | protein_coding | MRPS22 |
| 13 | 65418765 | 65418895 | snoRNA | SNORA33 |
| 13 | 66135315 | 66280571 | protein_coding | CLSTN2 |
| 13 | 66262848 | 66263188 | pseudogene |  |
| 13 | 66367961 | 66393613 | protein_coding | TRIM42 |
| 13 | 66824893 | 66840366 | protein_coding | ACPL2 |
| 13 | 66986260 | 66989715 | protein_coding | ZBTB38 |
| 13 | 67052013 | 67145729 | protein_coding | RASA2 |
| 13 | 67169983 | 67170720 | retrotransposed | |
| 13 | 67333892 | 67340309 | protein_coding | RNF7 |
| 13 | 67365647 | 67416131 | protein_coding | GRK7_PIG |
| 13 | 67907065 | 67907360 | misc_RNA | 7SK |
| 13 | 68029290 | 68098554 | protein_coding | C0JJ14_PIG |
| 13 | 68336312 | 68337445 | protein_coding | PAQR9 |
| 13 | 69098551 | 69098708 | snRNA | U1 |
| 13 | 70453913 | 70454387 | pseudogene |  |
| 13 | 71944979 | 71952916 | protein_coding | ZIC4 |
| 13 | 72120351 | 72120650 | misc_RNA | 7SK |
| 13 | 72404930 | 72448842 | protein_coding | CBPB1_PIG |
| 13 | 72514649 | 72515728 | protein_coding | AGTR1_PIG |
| 13 | 72593975 | 72597627 | protein_coding | GYG1 |
| 13 | 72674278 | 72708887 | protein_coding | HPS3 |
| 13 | 73108683 | 73108868 | protein_coding | TM4SF4 |
| 13 | 73626553 | 73675763 | protein_coding | TSC22D2 |
| 13 | 74060524 | 74102592 | protein_coding | MED12L |
| 13 | 74714248 | 74714553 | pseudogene |  |
| 13 | 74915628 | 74956490 | protein_coding | MBNL1 |
| 13 | 75379457 | 75464466 | protein_coding | RAP2B |
| 13 | 76287386 | 76287503 | rRNA | 5S_rRNA |
| 13 | 76301389 | 76398061 | protein_coding | MME |
| 13 | 76450713 | 76450830 | rRNA | 5S_rRNA |
| 13 | 76632206 | 76632311 | snRNA | U6 |
| 13 | 77150724 | 77156269 | protein_coding | PTX3 |
| 13 | 77947418 | 77972047 | protein_coding | IQCJ |
| 13 | 78265396 | 78311800 | protein_coding | SCHIP1 |
| 13 | 78412462 | 78420147 | protein_coding | IL12A_PIG |
| 13 | 78782572 | 78821617 | protein_coding | SMC4 |
| 13 | 78787511 | 78787608 | miRNA | ssc-mir-15b |
| 13 | 78787668 | 78787744 | miRNA | ssc-mir-16-2 |
| 13 | 78873415 | 78913688 | protein_coding | IFT80 |
| 13 | 79948322 | 79948466 | snoRNA | SNORA76 |
| 13 | 80316191 | 80316294 | snRNA | U6 |
| 13 | 90259259 | 90268451 | protein_coding | TTC14 |
| 13 | 90276465 | 90276568 | snRNA | U6 |
| 13 | 90487561 | 90549886 | protein_coding | FXR1 |
| 13 | 92107683 | 92213773 | protein_coding | YEATS2 |
| 13 | 92760005 | 92815018 | protein_coding | VPS8 |
| 13 | 93284305 | 93330918 | protein_coding | MAP3K13 |
| 13 | 93417739 | 93455241 | protein_coding | SENP2 |
| 13 | 93990036 | 94009031 | protein_coding | DNAJB11 |
| 13 | 94077968 | 94091697 | protein_coding | FETUB |
| 13 | 94118856 | 94155314 | protein_coding | KNG1 |
| 13 | 94158498 | 94158620 | snoRNA | SNORA27 |
| 13 | 94180998 | 94187232 | protein_coding | A6M930_PIG |
| 13 | 94182264 | 94182332 | snoRNA | snR39B |
| 13 | 94183771 | 94183897 | snoRNA | SNORA63 |
| 13 | 94184153 | 94184330 | snoRNA | SNORA81 |
| 13 | 94184607 | 94184737 | snoRNA | SNORA63 |
| 13 | 94184911 | 94185052 | snoRNA | SNORA4 |
| 13 | 94331082 | 94333622 | protein_coding | RTP1 |
| 13 | 94493416 | 94497383 | protein_coding | RTP4 |
| 13 | 95666070 | 95770781 | protein_coding | TPRG1 |
| 13 | 95981925 | 95982016 | snoRNA | U3 |
| 13 | 96789203 | 96832766 | protein_coding | A5JHN9_PIG |
| 13 | 96875924 | 96941480 | protein_coding | CCDC50 |
| 13 | 98158676 | 98158800 | rRNA | 5S_rRNA |
| 13 | 98453045 | 98474220 | protein_coding | HRASLS |
| 13 | 99303945 | 99305219 | protein_coding | FAM43A |
| 13 | 99660657 | 99679529 | protein_coding | APOD |
| 13 | 99935816 | 100335344 | protein_coding | DLG1 |
| 13 | 100347976 | 100371822 | protein_coding | MFI2 |
| 13 | 100603224 | 100649430 | protein_coding | PCYT1A |
| 13 | 100677915 | 100689006 | protein_coding | ZDHHC19 |
| 13 | 100772881 | 100799950 | protein_coding | TFRC |
| 13 | 100843879 | 100844069 | snRNA | U2 |
| 13 | 101026439 | 101057563 | protein_coding | MUC4 |
| 13 | 101160312 | 101290862 | protein_coding | LRCH3 |
| 13 | 101247042 | 101247168 | snoRNA | SNORA31 |
| 13 | 101342916 | 101346452 | protein_coding | RPL35A |
| 13 | 101350741 | 101362549 | protein_coding | LMLN |
| 13 | 101613738 | 101764960 | protein_coding | HEG1 |
| 13 | 101661653 | 101678573 | protein_coding | SLC12A8 |
| 13 | 101825813 | 101829076 | pseudogene |  |
| 13 | 101834422 | 101856869 | protein_coding | MUC13 |
| 13 | 101914435 | 101965525 | protein_coding | F1SQ56_PIG |
| 13 | 102657133 | 102917415 | protein_coding | MYLK |
| 13 | 103228425 | 103248063 | protein_coding | SEC22A |
| 13 | 103339909 | 103357650 | protein_coding | PDIA5 |
| 13 | 103501887 | 103549385 | protein_coding | DIRC2 |
| 13 | 103676560 | 103738614 | protein_coding | HSPBAP1 |
| 13 | 103893450 | 103901772 | protein_coding | DTX3L |
| 13 | 103935093 | 103935202 | snRNA | U6 |
| 13 | 103961666 | 103995796 | protein_coding | CD86 |
| 13 | 104107538 | 104107609 | snoRNA | SNORD112 |
| 13 | 104224695 | 104276371 | protein_coding | IQCB1 |
| 13 | 104289968 | 104381181 | protein_coding | GOLGB1 |
| 13 | 104386937 | 104415076 | protein_coding | HCLS1 |
| 13 | 104662327 | 104667438 | protein_coding | LRRC58 |
| 13 | 104725467 | 104789904 | protein_coding | GPR156 |
| 13 | 104854597 | 105138252 | protein_coding | GSK3B |
| 13 | 104974812 | 104979179 | protein_coding | NR1I2 |
| 13 | 105345992 | 105356336 | protein_coding | COX17_PIG |
| 13 | 105363332 | 105382558 | protein_coding | POPDC2 |
| 13 | 105512070 | 105524170 | protein_coding | TIMMDC1 |
| 13 | 105556673 | 105590207 | protein_coding | TMEM39A |
| 13 | 105963273 | 105991286 | protein_coding | IGSF11 |
| 13 | 108903143 | 108939281 | protein_coding | KIAA1407 |
| 13 | 109150674 | 109172711 | protein_coding | NAA50 |
| 13 | 109214813 | 109237832 | protein_coding | KIAA2018 |
| 13 | 109373770 | 109374878 | protein_coding | BOC |
| 13 | 109513400 | 109524631 | protein_coding | C3orf17 |
| 13 | 109781535 | 109818740 | protein_coding | CCDC80 |
| 13 | 109888655 | 109918847 | protein_coding | F1SLS7_PIG |
| 13 | 109994412 | 109995030 | pseudogene |  |
| 13 | 111141197 | 111166125 | protein_coding | PVRL3 |
| 13 | 111588741 | 111625422 | protein_coding | CD96 |
| 13 | 111799878 | 111959723 | protein_coding | MORC1 |
| 13 | 112119404 | 112120668 | protein_coding | RETNLB |
| 13 | 112357709 | 112415974 | protein_coding | IFT57 |
| 13 | 113490625 | 113491035 | retrotransposed | |
| 13 | 113830563 | 113914544 | protein_coding | ALCAM |
| 13 | 114333110 | 114333271 | snRNA | U1 |
| 13 | 115082807 | 115122972 | protein_coding | ZPLD1 |
| 13 | 115258594 | 115258724 | snoRNA | SNORA18 |
| 13 | 115422869 | 115445761 | protein_coding | CEP97 |
| 13 | 115468295 | 115506129 | protein_coding | FAM55C |
| 13 | 115533573 | 115543664 | protein_coding | NFKBIZ |
| 13 | 115580960 | 115587885 | protein_coding | RPL24 |
| 13 | 116121170 | 116348369 | protein_coding | ABI3BP |
| 13 | 116382124 | 116404839 | protein_coding | TFG |
| 13 | 116500631 | 116519460 | protein_coding | LNP1 |
| 13 | 116756853 | 116776650 | protein_coding | NIT2 |
| 13 | 118083243 | 118083364 | snoRNA | SNORA31 |
| 13 | 118552383 | 118564776 | protein_coding | CPOX |
| 13 | 118617372 | 118624795 | protein_coding | CLDND1 |
| 13 | 121532964 | 121543863 | protein_coding | PROS1 |
| 14 | 625773 | 625881 | snRNA | U6atac |
| 14 | 1102193 | 1107817 | protein_coding | CKS2 |
| 14 | 1109426 | 1152891 | protein_coding | SECISBP2 |
| 14 | 1365346 | 1366898 | protein_coding | D3Y270_PIG |
| 14 | 2118859 | 2118960 | snRNA | U6 |
| 14 | 2301720 | 2301821 | snRNA | U6 |
| 14 | 2351612 | 2352211 | protein_coding | LOC780430 |
| 14 | 2618466 | 2676342 | protein_coding | SYK |
| 14 | 3826422 | 3856471 | protein_coding | F1RN00_PIG |
| 14 | 6282245 | 6286053 | protein_coding | DOK2 |
| 14 | 6289454 | 6307086 | protein_coding | EPB49 |
| 14 | 6315415 | 6331040 | protein_coding | FAM160B2 |
| 14 | 6387471 | 6399944 | protein_coding | PSPC_PIG |
| 14 | 6474013 | 6480810 | protein_coding | POLR3D |
| 14 | 6592445 | 6636672 | protein_coding | SLC39A14 |
| 14 | 6695108 | 6748444 | protein_coding | F1RMA8_PIG |
| 14 | 6782366 | 6841003 | protein_coding | PDLIM2 |
| 14 | 6800399 | 6823405 | protein_coding | SORBS3 |
| 14 | 6853274 | 6866704 | protein_coding | KIAA1967 |
| 14 | 10337015 | 10424743 | protein_coding | 2ABA_PIG |
| 14 | 10609850 | 10685028 | protein_coding | DPYSL2 |
| 14 | 11248055 | 11313269 | protein_coding | PTK2B |
| 14 | 11336349 | 11412305 | protein_coding | EPHX2 |
| 14 | 11418875 | 11451350 | protein_coding | GGLO_PIG |
| 14 | 11525574 | 11560904 | protein_coding | ESCO2 |
| 14 | 11841298 | 11947449 | protein_coding | ELP3 |
| 14 | 12274326 | 12364898 | protein_coding | FZD3 |
| 14 | 12507675 | 12556035 | protein_coding | EXTL3 |
| 14 | 15243339 | 15296498 | protein_coding | D0G6X9_PIG |
| 14 | 15435874 | 15471971 | protein_coding | FBXO8 |
| 14 | 16038129 | 16040277 | protein_coding | HAND2 |
| 14 | 16305098 | 16306865 | protein_coding | HMGB2 |
| 14 | 17546945 | 17547055 | miRNA |  |
| 14 | 19523625 | 19546705 | protein_coding | AADAT |
| 14 | 19598450 | 19611544 | protein_coding | MFAP3L |
| 14 | 19653047 | 19653508 | pseudogene |  |
| 14 | 20059588 | 20246063 | protein_coding | NEK1 |
| 14 | 20537396 | 20578855 | protein_coding | SH3RF1 |
| 14 | 21051313 | 21051593 | misc_RNA | 7SK |
| 14 | 21173014 | 21252506 | protein_coding | DDX60 |
| 14 | 21490946 | 21857061 | protein_coding | SPOCK3 |
| 14 | 28690014 | 28917234 | protein_coding | LOC733643 |
| 14 | 29081567 | 29081673 | snRNA | U6 |
| 14 | 29178409 | 29193158 | protein_coding | CCDC92 |
| 14 | 29399105 | 29399390 | pseudogene |  |
| 14 | 29535490 | 29556662 | protein_coding | RILPL1 |
| 14 | 29570340 | 29584262 | protein_coding | C12orf65 |
| 14 | 29690784 | 29703678 | protein_coding | SETD8 |
| 14 | 29749232 | 29799089 | protein_coding | MPHOSPH9 |
| 14 | 29870515 | 29958520 | protein_coding | PITPNM2 |
| 14 | 30038728 | 30068605 | protein_coding | VPS37B |
| 14 | 30181175 | 30182197 | protein_coding | B9UM27_PIG |
| 14 | 30202013 | 30269099 | protein_coding | KNTC1 |
| 14 | 30214055 | 30232767 | protein_coding | RSRC2 |
| 14 | 30236476 | 30257234 | protein_coding | ZCCHC8 |
| 14 | 30241556 | 30241690 | snoRNA | SNORA9 |
| 14 | 30378835 | 30504101 | protein_coding | CLIP1 |
| 14 | 30603111 | 30613395 | protein_coding | LRRC43 |
| 14 | 30617711 | 30617844 | miRNA |  |
| 14 | 30617978 | 30618051 | miRNA |  |
| 14 | 30629389 | 30630042 | protein_coding | B3GNT4 |
| 14 | 30822865 | 30822971 | snRNA | U6 |
| 14 | 30950303 | 30961998 | protein_coding | Q06AT7_PIG |
| 14 | 31045706 | 31058890 | protein_coding | MORN3 |
| 14 | 31097322 | 31097422 | snRNA | U6 |
| 14 | 31299502 | 31347494 | protein_coding | ANAPC5 |
| 14 | 31348504 | 31405079 | protein_coding | CAMKK2 |
| 14 | 31564609 | 31653974 | protein_coding | IFT81 |
| 14 | 31836321 | 31841814 | protein_coding | GPN3 |
| 14 | 31876737 | 31886595 | protein_coding | C12orf24 |
| 14 | 31896749 | 31930523 | protein_coding | RAD9B |
| 14 | 32003878 | 32034906 | protein_coding | TCTN1 |
| 14 | 32031345 | 32031452 | miRNA |  |
| 14 | 32184521 | 32235330 | protein_coding | CCDC63 |
| 14 | 32684696 | 32711754 | protein_coding | SH2B3 |
| 14 | 33050756 | 33051455 | pseudogene |  |
| 14 | 33556321 | 33556703 | pseudogene |  |
| 14 | 34282374 | 34282454 | snRNA | U6 |
| 14 | 34521823 | 34744996 | protein_coding | TAOK3 |
| 14 | 34791193 | 34836013 | protein_coding | VSIG10 |
| 14 | 34841058 | 34861290 | protein_coding | WSB2 |
| 14 | 34908689 | 35328918 | protein_coding | KSR2 |
| 14 | 34954098 | 34954221 | miRNA |  |
| 14 | 34993613 | 34993719 | snRNA | U6 |
| 14 | 35577905 | 35615224 | protein_coding | FBXO21 |
| 14 | 35603071 | 35603218 | snRNA | U12 |
| 14 | 35646147 | 35702683 | protein_coding | TESC |
| 14 | 35877326 | 35877598 | protein_coding | HRK |
| 14 | 35976703 | 35992574 | protein_coding | C12orf49 |
| 14 | 36125861 | 36125959 | snRNA | U6 |
| 14 | 36579069 | 36649083 | protein_coding | MED13L |
| 14 | 37979782 | 37990959 | protein_coding | TBX3 |
| 14 | 38174956 | 38223080 | protein_coding | TBX5 |
| 14 | 38359135 | 38359241 | snRNA | U6 |
| 14 | 38482035 | 38601479 | protein_coding | RBM19 |
| 14 | 38915894 | 38952993 | protein_coding | DTX1 |
| 14 | 38997324 | 39004361 | protein_coding | CCDC42B |
| 14 | 39062494 | 39069827 | protein_coding | IQCD |
| 14 | 39086164 | 39116473 | protein_coding | TPCN1 |
| 14 | 39137441 | 39149442 | protein_coding | OAS2 |
| 14 | 39468945 | 39520006 | protein_coding | C12orf51 |
| 14 | 39574569 | 39726723 | protein_coding | NAA25 |
| 14 | 39759052 | 39759160 | snRNA | U6 |
| 14 | 39812424 | 39843035 | protein_coding | ALDH2_PIG |
| 14 | 39859435 | 39892903 | protein_coding | B7U6F1_PIG |
| 14 | 39965965 | 39966070 | snRNA | U6 |
| 14 | 40150998 | 40268797 | protein_coding | CCDC64 |
| 14 | 40367283 | 40375055 | protein_coding | PXN |
| 14 | 40380802 | 40385791 | protein_coding | RLA0_PIG |
| 14 | 40430181 | 40439002 | protein_coding | F1RJK2_PIG |
| 14 | 40530686 | 40539834 | protein_coding | SRSF9 |
| 14 | 40550770 | 40552174 | protein_coding | TRIAP1 |
| 14 | 40565869 | 40585268 | protein_coding | RNF10 |
| 14 | 40574175 | 40575162 | pseudogene |  |
| 14 | 40640432 | 40651116 | protein_coding | CABP1 |
| 14 | 40665604 | 40675468 | protein_coding | MLEC |
| 14 | 40688019 | 40699379 | protein_coding | UNC119B |
| 14 | 40706674 | 40720741 | protein_coding | F1RJH2_PIG |
| 14 | 41030504 | 41050007 | protein_coding | Q4PJJ8_PIG |
| 14 | 41095713 | 41102518 | protein_coding | ANKRD13A |
| 14 | 41158158 | 41210960 | protein_coding | GIT2 |
| 14 | 41232088 | 41326221 | protein_coding | C12orf34 |
| 14 | 41253890 | 41277076 | protein_coding | GLTP |
| 14 | 41586464 | 41599398 | protein_coding | MMAB |
| 14 | 41671712 | 41696531 | protein_coding | KCTD10 |
| 14 | 41849454 | 41856816 | protein_coding | FOXN4 |
| 14 | 42038720 | 42043216 | protein_coding | ALKBH2 |
| 14 | 42103688 | 42179583 | protein_coding | SVOP |
| 14 | 42107294 | 42107453 | snRNA | U1 |
| 14 | 42222827 | 42284518 | protein_coding | SSH1 |
| 14 | 42321730 | 42402815 | protein_coding | CORO1C |
| 14 | 42615819 | 42618212 | protein_coding | FICD |
| 14 | 42651169 | 42652260 | protein_coding | CML1_PIG |
| 14 | 43176343 | 43237875 | protein_coding | SGSM1 |
| 14 | 43505444 | 43511833 | protein_coding | CRYBB3 |
| 14 | 43702868 | 43714419 | protein_coding | CRYBB2 |
| 14 | 43976749 | 44165377 | protein_coding | MYO18B |
| 14 | 44545677 | 44621675 | protein_coding | SEZ6L |
| 14 | 44776764 | 44783838 | protein_coding | ASPHD2 |
| 14 | 44815970 | 44838730 | protein_coding | SRRD |
| 14 | 44954612 | 44961093 | protein_coding | CRYBA4 |
| 14 | 46441826 | 46441990 | snRNA | U1 |
| 14 | 46709925 | 46717776 | protein_coding | ZNRF3 |
| 14 | 46758562 | 46802303 | protein_coding | KREMEN1 |
| 14 | 46891209 | 46993291 | protein_coding | EWSR1 |
| 14 | 46935769 | 46955338 | protein_coding | EMID1 |
| 14 | 47530966 | 47533981 | protein_coding | UQCR10 |
| 14 | 47647544 | 47794758 | protein_coding | MTMR3 |
| 14 | 47853415 | 47876819 | protein_coding | HORMAD2 |
| 14 | 48150445 | 48158791 | protein_coding | CCDC157 |
| 14 | 48179234 | 48199044 | protein_coding | SEC14L2 |
| 14 | 48201842 | 48205269 | protein_coding | MTFP1 |
| 14 | 48302979 | 48320699 | protein_coding | TCN2 |
| 14 | 48325509 | 48331197 | protein_coding | SLC35E4 |
| 14 | 48349175 | 48354755 | protein_coding | C5orf52 |
| 14 | 48418210 | 48543952 | protein_coding | F1RPE0_PIG |
| 14 | 48898094 | 48923133 | protein_coding | LIMK2 |
| 14 | 48979363 | 48979531 | pseudogene |  |
| 14 | 49011480 | 49037635 | protein_coding | DRG1 |
| 14 | 49115036 | 49188468 | protein_coding | SFI1 |
| 14 | 49307821 | 49408102 | protein_coding | DEPDC5 |
| 14 | 49332410 | 49332516 | snRNA | U6 |
| 14 | 49745853 | 49932451 | protein_coding | LAC_PIG |
| 14 | 50049081 | 50071848 | protein_coding | GNAZ |
| 14 | 50087986 | 50099343 | protein_coding | RAB36 |
| 14 | 50407704 | 50505710 | protein_coding | SPECC1L |
| 14 | 50623355 | 50626970 | protein_coding | FAM211B |
| 14 | 50651248 | 50661604 | protein_coding | C22orf13 |
| 14 | 50885555 | 50887617 | protein_coding | DERL3 |
| 14 | 50936155 | 50938053 | protein_coding | CHCHD10 |
| 14 | 50942827 | 50944083 | protein_coding | VPREB3 |
| 14 | 50948794 | 50950140 | protein_coding | ZNF70 |
| 14 | 51035200 | 51052555 | protein_coding | F1RL05_PIG |
| 14 | 51213645 | 51236463 | protein_coding | ERK2 |
| 14 | 51264367 | 51280167 | protein_coding | YPEL1 |
| 14 | 51334545 | 51336293 | protein_coding | YDJC |
| 14 | 51460746 | 51464326 | protein_coding | HIC2 |
| 14 | 51588223 | 51603171 | protein_coding | SNAP29 |
| 14 | 51625285 | 51651739 | protein_coding | CRKL |
| 14 | 51661019 | 51671982 | protein_coding | AIFM3 |
| 14 | 51673087 | 51685452 | protein_coding | LZTR1 |
| 14 | 51719911 | 51728095 | protein_coding | F1RKA0_PIG |
| 14 | 51767126 | 51790870 | protein_coding | SMPD4 |
| 14 | 51875915 | 51908622 | protein_coding | KLHL22 |
| 14 | 51924446 | 51935107 | protein_coding | SCARF2 |
| 14 | 52048808 | 52049902 | protein_coding | TSSK1B |
| 14 | 52053941 | 52055017 | protein_coding | TSSK2 |
| 14 | 52244529 | 52269749 | protein_coding | CDC45 |
| 14 | 52597967 | 52598066 | snoRNA | U3 |
| 14 | 52639564 | 52656710 | protein_coding | COMT |
| 14 | 52703712 | 52703787 | miRNA | ssc-mir-185 |
| 14 | 52706174 | 52717838 | protein_coding | C22orf25 |
| 14 | 52728045 | 52742722 | protein_coding | DGCR8 |
| 14 | 52728153 | 52728210 | miRNA |  |
| 14 | 52748488 | 52754653 | protein_coding | RANBP1 |
| 14 | 52753820 | 52753945 | snoRNA | SNORA77 |
| 14 | 52757424 | 52769401 | protein_coding | ZDHHC8 |
| 14 | 54709891 | 54717308 | protein_coding | ZP4 |
| 14 | 55075707 | 55075813 | snoRNA | SNORA25 |
| 14 | 55562496 | 55562580 | rRNA | 5S_rRNA |
| 14 | 55827439 | 55827541 | misc_RNA | Y_RNA |
| 14 | 56026771 | 56075441 | protein_coding | HEATR1 |
| 14 | 61696429 | 61713771 | protein_coding | FAM89A |
| 14 | 61737986 | 61820490 | protein_coding | TTC13 |
| 14 | 61845981 | 61879993 | protein_coding | C1orf198 |
| 14 | 62006500 | 62040103 | protein_coding | COG2 |
| 14 | 62554361 | 62585055 | protein_coding | TAF5L |
| 14 | 62652910 | 62705275 | protein_coding | NUP133 |
| 14 | 62713314 | 62716162 | protein_coding | ACTS_PIG |
| 14 | 63277635 | 63303380 | protein_coding | ZNF25 |
| 14 | 63388112 | 63396358 | protein_coding | ZNF248 |
| 14 | 63420198 | 63458558 | protein_coding | BMS1 |
| 14 | 67690434 | 67813348 | protein_coding | C10orf107 |
| 14 | 67947025 | 68183610 | protein_coding | ARID5B |
| 14 | 68440905 | 68465103 | protein_coding | ZNF365 |
| 14 | 68948048 | 68948860 | protein_coding | ADO |
| 14 | 69264823 | 69302547 | protein_coding | NRBF2 |
| 14 | 69890847 | 69942509 | protein_coding | REEP3 |
| 14 | 73310095 | 73481395 | protein_coding | LRRTM3 |
| 14 | 74220079 | 74245927 | protein_coding | SIRT1 |
| 14 | 74307561 | 74411966 | protein_coding | HERC4 |
| 14 | 74509863 | 74521076 | protein_coding | HNRNPH3 |
| 14 | 74616492 | 74828703 | protein_coding | TET1 |
| 14 | 74878550 | 74878614 | snoRNA | SNORD98 |
| 14 | 74990609 | 75130244 | protein_coding | STOX1 |
| 14 | 75136525 | 75177551 | protein_coding | DDX50 |
| 14 | 75186560 | 75207640 | protein_coding | DDX21 |
| 14 | 75218179 | 75244927 | protein_coding | KIAA1279 |
| 14 | 75329507 | 75346802 | protein_coding | SRGN |
| 14 | 75365753 | 75388011 | protein_coding | VPS26A |
| 14 | 75394156 | 75421289 | protein_coding | SUPV3L1 |
| 14 | 75648115 | 75709042 | protein_coding | TSPAN15 |
| 14 | 75942650 | 75995076 | protein_coding | F1SUE8_PIG |
| 14 | 76447309 | 76494374 | protein_coding | EIF4EBP2 |
| 14 | 76595303 | 76634042 | protein_coding | KIAA1274 |
| 14 | 76771145 | 76804964 | protein_coding | ADAMTS14 |
| 14 | 77302358 | 77387225 | protein_coding | UNC5B |
| 14 | 77405063 | 77447879 | protein_coding | SLC29A3 |
| 14 | 77772954 | 77915348 | protein_coding | CDH23 |
| 14 | 78310507 | 78320584 | protein_coding | ANAPC16 |
| 14 | 78345537 | 78347197 | protein_coding | DDIT4 |
| 14 | 78779843 | 79116317 | protein_coding | MCU |
| 14 | 79123091 | 79143513 | protein_coding | OIT3 |
| 14 | 79207207 | 79324904 | protein_coding | FAM149B1 |
| 14 | 79699240 | 79706115 | protein_coding | SYNPO2L |
| 14 | 79709549 | 79718718 | protein_coding | MYOZ1_PIG |
| 14 | 79778202 | 79801010 | protein_coding | SEC24C |
| 14 | 79809667 | 79811230 | protein_coding | CHCHD1 |
| 14 | 79813943 | 79828231 | protein_coding | KIAA0913 |
| 14 | 79931353 | 79937213 | protein_coding | F1SU38_PIG |
| 14 | 80002216 | 80113814 | protein_coding | VINC_PIG |
| 14 | 80215283 | 80533486 | protein_coding | ADK |
| 14 | 80673634 | 80673715 | miRNA |  |
| 14 | 80747464 | 80811705 | protein_coding | KAT6B |
| 14 | 80897338 | 80950958 | protein_coding | SAMD8 |
| 14 | 80983232 | 80998329 | protein_coding | VDAC2 |
| 14 | 81145889 | 81150190 | protein_coding | ZNF503-AS2 |
| 14 | 81722863 | 81734006 | protein_coding | C10orf11 |
| 14 | 82088097 | 82088203 | snRNA | U6 |
| 14 | 83839069 | 83846368 | protein_coding | F1S2E5_PIG |
| 14 | 84802334 | 85175686 | protein_coding | ZMIZ1 |
| 14 | 85205810 | 85212642 | protein_coding | F1S2E3_PIG |
| 14 | 85508342 | 85523061 | protein_coding | SFTPD_PIG |
| 14 | 85679684 | 85691469 | protein_coding | DYDC2 |
| 14 | 85731576 | 85753309 | protein_coding | FAM213A |
| 14 | 85816502 | 85845941 | protein_coding | TSPAN14 |
| 14 | 85862244 | 85946762 | protein_coding | SH2D4B |
| 14 | 86025598 | 86026141 | pseudogene |  |
| 14 | 87515199 | 87778937 | protein_coding | NRG3 |
| 14 | 88426081 | 88426184 | snRNA | U6 |
| 14 | 88720505 | 88735626 | protein_coding | GHITM |
| 14 | 88748258 | 88866537 | protein_coding | C10orf99 |
| 14 | 88763717 | 88763826 | snRNA | U6 |
| 14 | 88874691 | 88874800 | snRNA | U6 |
| 14 | 88876118 | 88898171 | protein_coding | CDHR1 |
| 14 | 88931225 | 88941989 | protein_coding | RGR |
| 14 | 89022007 | 89119743 | protein_coding | FAM190B |
| 14 | 93192845 | 93453553 | protein_coding | WDFY4 |
| 14 | 93629299 | 93629405 | snRNA | U6 |
| 14 | 93753249 | 93757532 | protein_coding | C10orf71 |
| 14 | 93935272 | 93935363 | miRNA |  |
| 14 | 93957673 | 94007899 | protein_coding | CLAT_PIG |
| 14 | 94018187 | 94027763 | protein_coding | C10orf53 |
| 14 | 94616209 | 94733974 | protein_coding | ZFAND4 |
| 14 | 94749760 | 94875780 | protein_coding | 8-Mar |
| 14 | 95912350 | 95927577 | protein_coding | CXCL12 |
| 14 | 97288202 | 97289303 | pseudogene |  |
| 14 | 100197944 | 100670231 | protein_coding | PCDH15 |
| 14 | 101464157 | 101467971 | protein_coding | MBL2 |
| 14 | 102912705 | 102912806 | snoRNA | snoU13 |
| 14 | 103519490 | 103590115 | protein_coding | A1CF |
| 14 | 103817282 | 103909542 | protein_coding | SGMS1 |
| 14 | 106148385 | 106149806 | protein_coding | IFIT1 |
| 14 | 106165402 | 106166847 | protein_coding | IFIT5 |
| 14 | 109460106 | 109621572 | protein_coding | EXOC6 |
| 14 | 110861592 | 111099030 | protein_coding | PLCE1 |
| 14 | 111145778 | 111145876 | snRNA | U6 |
| 14 | 111215465 | 111240295 | protein_coding | TBC1D12 |
| 14 | 111627139 | 111667418 | protein_coding | F1SC62_PIG |
| 14 | 111721928 | 111757286 | protein_coding | CP242_PIG |
| 14 | 111792256 | 111792688 | pseudogene |  |
| 14 | 112352301 | 112444933 | protein_coding | F1SC45_PIG |
| 14 | 112529740 | 112560616 | protein_coding | CC2D2B |
| 14 | 112668936 | 112673411 | protein_coding | ZNF518B |
| 14 | 112807695 | 112841436 | protein_coding | DNTT |
| 14 | 117246780 | 117447356 | protein_coding | BTRC |
| 14 | 117481044 | 117501046 | protein_coding | DPCD |
| 14 | 118061589 | 118064143 | protein_coding | A5A775_PIG |
| 14 | 118117579 | 118133378 | protein_coding | PPRC1 |
| 14 | 118135379 | 118146123 | protein_coding | NOLC1 |
| 14 | 118208287 | 118211145 | protein_coding | D0G6S7_PIG |
| 14 | 118227136 | 118361091 | protein_coding | GBF1 |
| 14 | 118388488 | 118392800 | protein_coding | NFKB2 |
| 14 | 118463701 | 118465668 | protein_coding | CUEDC2 |
| 14 | 118471588 | 118471686 | miRNA | ssc-mir-146b |
| 14 | 118489409 | 118503360 | protein_coding | TMEM180 |
| 14 | 118526054 | 118648733 | protein_coding | SUFU |
| 14 | 118662408 | 118675520 | protein_coding | TRIM8 |
| 14 | 118861339 | 118874214 | protein_coding | C10orf26 |
| 14 | 118908266 | 118944708 | protein_coding | AS3MT |
| 14 | 118966775 | 119133301 | protein_coding | CNNM2 |
| 14 | 119419172 | 119435453 | protein_coding | TAF5 |
| 14 | 119596316 | 119614632 | protein_coding | NEURL |
| 14 | 120006006 | 120060599 | protein_coding | SLK |
| 14 | 120171544 | 120175306 | protein_coding | SFR1 |
| 14 | 120314308 | 120324732 | protein_coding | GSTO1_PIG |
| 14 | 120414318 | 120436593 | protein_coding | CCDC147 |
| 14 | 121781219 | 121781376 | snRNA | U1 |
| 14 | 122929230 | 122929316 | miRNA |  |
| 14 | 124058756 | 124058904 | snoRNA | SNORA62 |
| 14 | 126055114 | 126185351 | protein_coding | ADD3 |
| 14 | 126513163 | 126524943 | protein_coding | F1S5M6_PIG |
| 14 | 126576623 | 126615913 | protein_coding | SMC3 |
| 14 | 126813654 | 126868038 | protein_coding | RBM20 |
| 14 | 126902476 | 126931666 | protein_coding | PDCD4 |
| 14 | 126999861 | 127046472 | protein_coding | SHOC2 |
| 14 | 127073819 | 127075213 | protein_coding | ADA2A_PIG |
| 14 | 131030054 | 131064184 | protein_coding | FAM160B1 |
| 14 | 131290888 | 131394587 | protein_coding | ATRNL1 |
| 14 | 132368461 | 132418098 | protein_coding | C10orf96 |
| 14 | 132562702 | 132606475 | protein_coding | F1S4T8_PIG |
| 14 | 132614445 | 132629698 | protein_coding | B8XY18_PIG |
| 14 | 138232827 | 138336900 | protein_coding | BTBD16 |
| 14 | 138385176 | 138438885 | protein_coding | PLEKHA1 |
| 14 | 138468111 | 138528927 | protein_coding | HTRA1 |
| 14 | 138631259 | 138656610 | protein_coding | SAMD4B |
| 14 | 138675810 | 138677628 | protein_coding | D0VE66_PIG |
| 14 | 138683137 | 138691683 | protein_coding | PLEKHG2 |
| 14 | 138780274 | 138782182 | protein_coding | C10orf120 |
| 14 | 138975726 | 138987606 | protein_coding | PSTK |
| 14 | 139147449 | 139159629 | protein_coding | BUB3 |
| 14 | 139681386 | 139700467 | protein_coding | GPR26 |
| 14 | 140693803 | 140716055 | protein_coding | F1SDP3_PIG |
| 14 | 140756329 | 140873324 | protein_coding | LHPP |
| 14 | 140888912 | 140889025 | rRNA | 5S_rRNA |
| 14 | 141032847 | 141061590 | protein_coding | FAM175B |
| 14 | 141156260 | 141201145 | protein_coding | ZRANB1 |
| 14 | 142036154 | 142079891 | protein_coding | C10orf137 |
| 14 | 142126292 | 142138279 | protein_coding | BCCIP |
| 14 | 142192568 | 142293599 | protein_coding | FANK1 |
| 14 | 143384673 | 143470128 | protein_coding | DOCK1 |
| 14 | 144091014 | 144092731 | protein_coding | FOXI2 |
| 14 | 144447776 | 144472914 | protein_coding | PTPRE |
| 14 | 145068079 | 145068185 | rRNA | 5S_rRNA |
| 14 | 145738712 | 146018685 | protein_coding | MGMT |
| 14 | 146334011 | 146369522 | protein_coding | GLRX3 |
| 14 | 147024599 | 147031026 | protein_coding | TCERG1L |
| 14 | 147161054 | 147174410 | protein_coding | STK32C |
| 14 | 147411997 | 147574963 | protein_coding | INPP5A |
| 14 | 147689228 | 147732584 | protein_coding | TTC40 |
| 14 | 147821963 | 147837179 | protein_coding | GPR123 |
| 14 | 147853056 | 147931692 | protein_coding | KNDC1 |
| 14 | 147937297 | 147938633 | protein_coding | UTF1 |
| 14 | 147999190 | 148004381 | protein_coding | ZNF511 |
| 14 | 148105589 | 148108724 | protein_coding | SYCE1 |
| 14 | 148247553 | 148248478 | pseudogene |  |
| 14 | 148480842 | 148492810 | protein_coding | ECHS1 |
| 14 | 148497020 | 148500641 | protein_coding | C10orf125 |
| 15 | 4309981 | 4502153 | protein_coding | GTDC1 |
| 15 | 14081271 | 14084327 | protein_coding | CXCR4_PIG |
| 15 | 14422370 | 14458242 | protein_coding | UBXN4 |
| 15 | 14858763 | 14947562 | protein_coding | ZRANB3 |
| 15 | 15103608 | 15153604 | protein_coding | RAB3GAP1 |
| 15 | 15195030 | 15233168 | protein_coding | YSK4 |
| 15 | 15463454 | 15504357 | protein_coding | ACMSD |
| 15 | 15543730 | 15915160 | protein_coding | TMEM163 |
| 15 | 17855025 | 17920112 | protein_coding | NCKAP5 |
| 15 | 17922739 | 17958489 | protein_coding | LYPD1 |
| 15 | 19864063 | 19864169 | snRNA | U6 |
| 15 | 41989098 | 42127901 | protein_coding | TRAPPC11 |
| 15 | 42171975 | 42375631 | protein_coding | STOX2 |
| 15 | 42806968 | 42815526 | protein_coding | F1RSZ5_PIG |
| 15 | 42918315 | 42985195 | protein_coding | ACSL1 |
| 15 | 42999826 | 43027571 | protein_coding | MLF1IP |
| 15 | 43356384 | 43356773 | protein_coding | HELT |
| 15 | 43427369 | 43628381 | protein_coding | SNX25 |
| 15 | 43718678 | 43737823 | protein_coding | C15H4orf47 |
| 15 | 43848819 | 43851851 | protein_coding | ANKRD37 |
| 15 | 44284517 | 44293098 | protein_coding | TLR3 |
| 15 | 44323851 | 44323980 | snoRNA | SNORA31 |
| 15 | 44327768 | 44351706 | protein_coding | FAM149A |
| 15 | 44379050 | 44629057 | protein_coding | Q866A8_PIG |
| 15 | 44964565 | 44971596 | protein_coding | TM2D2 |
| 15 | 45168231 | 45168478 | pseudogene |  |
| 15 | 45762923 | 45773350 | protein_coding | BAG4 |
| 15 | 45787298 | 45824613 | protein_coding | DDHD2 |
| 15 | 45997228 | 46004106 | protein_coding | GOT1L1 |
| 15 | 46024628 | 46055647 | protein_coding | RAB11FIP1 |
| 15 | 46065741 | 46069947 | protein_coding | BRF2 |
| 15 | 61058427 | 61119399 | protein_coding | PKP4 |
| 15 | 61234634 | 61258629 | protein_coding | DAPL1 |
| 15 | 61502268 | 61597464 | protein_coding | TANC1 |
| 15 | 70818151 | 70830936 | protein_coding | NOSTRIN |
| 15 | 70866337 | 70873047 | protein_coding | G6PC2 |
| 15 | 71311502 | 71329392 | protein_coding | KBTBD10 |
| 15 | 71377171 | 71407147 | protein_coding | PPIG |
| 15 | 71442427 | 71443152 | protein_coding | PHOSPHO2 |
| 15 | 71458515 | 71478199 | protein_coding | KLHL23 |
| 15 | 71511519 | 71520569 | protein_coding | SSB |
| 15 | 71579984 | 71648113 | protein_coding | UBR3 |
| 15 | 71824819 | 71923011 | protein_coding | MYO15A |
| 15 | 72286554 | 72301194 | protein_coding | GORASP2 |
| 15 | 78735660 | 78757695 | protein_coding | RBM45 |
| 15 | 78768996 | 78769158 | snRNA | U1 |
| 15 | 79113489 | 79120097 | protein_coding | DFNB59 |
| 15 | 79139511 | 79160090 | protein_coding | PLEKHA3 |
| 15 | 88917303 | 89006719 | protein_coding | PMS1 |
| 15 | 89313671 | 89313955 | protein_coding | C2orf88 |
| 15 | 94440434 | 94441904 | protein_coding | C2orf66 |
| 15 | 94877804 | 94893752 | protein_coding | COQ10B |
| 15 | 94913554 | 94915922 | protein_coding | F1SMZ6_PIG |
| 15 | 94930691 | 94956685 | protein_coding | MOBKL3 |
| 15 | 95061179 | 95062960 | protein_coding | MARS2 |
| 15 | 95226541 | 95232941 | protein_coding | BOLL |
| 15 | 95398577 | 95423950 | protein_coding | PLCL1 |
| 15 | 98644855 | 98655515 | protein_coding | STRADB |
| 15 | 98672201 | 98701320 | protein_coding | TRAK2 |
| 15 | 98710461 | 98737344 | protein_coding | ALS2CR12 |
| 15 | 98741656 | 98744520 | protein_coding | F1SI34_PIG |
| 15 | 99584694 | 99585976 | protein_coding | NOP16 |
| 15 | 99623146 | 99680923 | protein_coding | B3GEL9_PIG |
| 15 | 99731752 | 99871405 | protein_coding | FAM117B |
| 15 | 99993494 | 100192972 | protein_coding | ALS2CR8 |
| 15 | 100516676 | 100516787 | retrotransposed | |
| 15 | 100631088 | 100631278 | snRNA | U2 |
| 15 | 100704141 | 100709270 | protein_coding | CTLA4 |
| 15 | 102225858 | 102225892 | snoRNA | snoZ196 |
| 15 | 102226215 | 102226347 | snoRNA | SNORA41 |
| 15 | 102355337 | 102358519 | protein_coding | ZDBF2 |
| 15 | 102464691 | 102650958 | protein_coding | ADAM23 |
| 15 | 102787625 | 102807918 | protein_coding | FASTKD2 |
| 15 | 102985837 | 103014779 | protein_coding | CPO |
| 15 | 103644981 | 103655071 | protein_coding | METTL21A |
| 15 | 103977848 | 104018507 | protein_coding | PLEKHM3 |
| 15 | 104557307 | 104557496 | snRNA | U2 |
| 15 | 105467444 | 105544481 | protein_coding | MAP2 |
| 15 | 106077349 | 106077455 | snRNA | U6 |
| 15 | 106091157 | 106091263 | snRNA | U6 |
| 15 | 106098526 | 106098658 | snoRNA | SNORA70 |
| 15 | 106340959 | 106352733 | protein_coding | LANCL1 |
| 15 | 106465468 | 106551048 | protein_coding | CPS1 |
| 16 | 25136193 | 25154589 | protein_coding | HMGCS1 |
| 16 | 25369752 | 25390146 | protein_coding | C5orf34 |
| 16 | 25409590 | 25459558 | protein_coding | NNT |
| 16 | 45838185 | 45931543 | protein_coding | MAP1B |
| 16 | 45875114 | 45875180 | miRNA |  |
| 16 | 46039843 | 46092746 | protein_coding | PTCD2 |
| 16 | 46117946 | 46136424 | protein_coding | ZNF366 |
| 16 | 47720623 | 47784065 | protein_coding | CPEB4 |
| 16 | 48332183 | 48344135 | protein_coding | STC2 |
| 16 | 48624823 | 48624926 | snRNA | U6 |
| 16 | 48644987 | 48667333 | protein_coding | ERGIC1 |
| 16 | 48676058 | 48684478 | protein_coding | F2Z5N5_PIG |
| 16 | 48691321 | 48697547 | protein_coding | LOC733646 |
| 16 | 48835441 | 48838351 | protein_coding | F1RS00_PIG |
| 16 | 49182275 | 49266006 | protein_coding | SH3PXD2B |
| 16 | 49319605 | 49383853 | protein_coding | UBTD2 |
| 16 | 72900438 | 72908115 | protein_coding | MED10 |
| 16 | 73488379 | 73501262 | protein_coding | KIAA0947 |
| 16 | 74102369 | 74102458 | snRNA | U6 |
| 17 | 3375773 | 3375897 | snoRNA | SNORA40 |
| 17 | 3479612 | 3479736 | snoRNA | SNORA40 |
| 17 | 4503558 | 4585021 | protein_coding | EFHA2 |
| 17 | 4632474 | 4668850 | protein_coding | ZDHHC2 |
| 17 | 4692610 | 4730303 | protein_coding | VPS37A |
| 17 | 5066629 | 5186685 | protein_coding | PDGFRL |
| 17 | 5235094 | 5235209 | snRNA | U6atac |
| 17 | 5324105 | 5324220 | snRNA | U6atac |
| 17 | 27759350 | 27759817 | protein_coding | C20orf79 |
| 17 | 28607874 | 28773547 | protein_coding | SLC24A3 |
| 17 | 31844132 | 31844214 | miRNA |  |
| 17 | 31933714 | 31934136 | protein_coding | NXT1 |
| 17 | 38216545 | 38220001 | protein_coding | COMMD7 |
| 17 | 38365920 | 38389646 | protein_coding | DNMT3B |
| 17 | 38395379 | 38426921 | protein_coding | Q2XVP5_PIG |
| 17 | 43302744 | 43347401 | protein_coding | CTNNBL1 |
| 17 | 43354373 | 43368453 | protein_coding | VSTM2L |
| 17 | 43500949 | 43554594 | protein_coding | RPRD1B |
| 18 | 91259 | 132891 | protein_coding | F1SHW4_PIG |
| 18 | 905504 | 1007568 | protein_coding | LMBR1 |
| 18 | 1142696 | 1169728 | protein_coding | RNF32 |
| 18 | 1734991 | 1744780 | protein_coding | SHH |
| 18 | 1964304 | 1970939 | protein_coding | CNPY1 |
| 18 | 13156510 | 13175831 | protein_coding | SLC35B4 |

**Table S4:** Genes searched in DGV

| Gene | Variation | Position |
| --- | --- | --- |
| NCK2 | Variation_0021 | chr2:106345204..106485786 |
| NR1I2 | Variation_0035 | chr3:119443790..119608882 |
| CLSTN2 | Variation_0036 | chr3:139780680..139958764 |
| DLG1 | Variation_0043 | chr3:196892658..197063838 |
| BRD2 | Variation_0076 | chr6:32850752..32999280 |
| SMAP1 | Variation_0086 | chr6:71543379..71627678 |
| TWIST1 | Variation_0100 | chr7:19139165..19177498 |
| FERD3L | Variation_0101 | chr7:19178786..19210119 |
| SEMA3E | Variation_0105 | chr7:83220454..83367572 |
| TTC39B | Variation_0127 | chr9:15230016..15411786 |
| PAN2 | Variation_0164 | chr12:56553078..56902907 |
| NAA25 | Variation_0166 | chr12:112504265..112680932 |
| DBNDD1 | Variation_0200 | chr16:90042193..90164923 |
| CALCOCO2 | Variation_0208 | chr17:46920868..47071888 |
| ERN1 | Variation_0210 | chr17:62118842..62273472 |
| CYTH1 | Variation_0212 | chr17:76603410..76723331 |
| CPS1 | Variation_0271 | chr2:211528918..211658981 |
| NPEPPS | Variation_0328 | chr17:45616396..45637602 |
| SLCO3A1 | Variation_0461 | chr15:92622348..92625190 |
| SLCO3A1 | Variation_0462 | chr15:92644907..92648729 |
| SLCO3A1 | Variation_0463 | chr15:92651953..92655904 |
| CES1 | Variation_0488 | chr16:55850175..55855602 |
| SLC39A11 | Variation_0503 | chr17:70849835..70879053 |
| KATNAL2 | Variation_0506 | chr18:44550838..44552021 |
| EXD3 | Variation_0654 | chr9:140210789..140228645 |
| CD2AP | Variation_0718 | chr6:47307024..47461399 |
| SH2D4B | Variation_0762 | chr10:82393435..82549936 |
| KIAA1267 | Variation_0801 | chr17:44079480..44240711 |
| PITPNC1 | Variation_0806 | chr17:65389627..65550905 |
| MAPK10 | Variation_0981 | chr4:86976387..86979952 |
| CCDC50 | Variation_1068 | chr3:191067863..191069984 |
| STOX1 | Variation_1230 | chr10:70618511..70620366 |
| STRC | Variation_1282 | chr15:43888976..43939642 |
| HFM1 | Variation_1547 | chr1:91790876..91801207 |
| CCDC50 | Variation_1659 | chr3:191067244..191069984 |
| SLIT2 | Variation_1667 | chr4:20562080..20563108 |
| DDHD2 | Variation_1778 | chr8:38108133..38110215 |
| KIAA1429 | Variation_1798 | chr8:95558694..95571443 |
| PTK2 | Variation_1806 | chr8:142002897..142004193 |
| CECR2 | Variation_2007 | chr22:18016508..18018604 |
| KIAA1644 | Variation_2027 | chr22:44638701..44652886 |
| SGMS1 | Variation_2156 | chr10:52326566..52397589 |
| SH2D4B | Variation_2161 | chr10:82393435..82549936 |
| MRM1 | Variation_2223 | chr17:34875147..35040221 |
| KIAA1267 | Variation_2226 | chr17:44079480..44240711 |
| C22orf25 | Variation_2261 | chr22:19879187..20055258 |
| SEZ6L | Variation_2273 | chr22:26513140..26694294 |
| TMEM108 | Variation_2474 | chr3:133020950..133032892 |
| CLSTN2 | Variation_2475 | chr3:140009220..140460228 |
| DOK7 | Variation_2494 | chr4:3472162..3510598 |
| MAPK10 | Variation_2533 | chr4:87075759..87138765 |
| MDFI | Variation_2626 | chr6:41598524..41641489 |
| PARK2 | Variation_2662 | chr6:161935676..161936709 |
| PARK2 | Variation_2663 | chr6:162490938..162507690 |
| PARK2 | Variation_2664 | chr6:162735881..162739765 |
| PDGFRL | Variation_2743 | chr8:17339101..17603857 |
| TRAPPC9 | Variation_2777 | chr8:140967628..140977205 |
| BMS1 | Variation_2862 | chr10:43104068..43352995 |
| PCDH15 | Variation_2868 | chr10:56436508..56473754 |
| CDH23 | Variation_2878 | chr10:73237903..73286155 |
| C10orf11 | Variation_2879 | chr10:77536046..77736622 |
| NRG3 | Variation_2882 | chr10:84044632..84058927 |
| NRG3 | Variation_2883 | chr10:84400714..84437193 |
| NRG3 | Variation_2884 | chr10:84571922..84618651 |
| ATRNL1 | Variation_2891 | chr10:117414868..117471615 |
| DOCK1 | Variation_2893 | chr10:128991629..129118884 |
| QSER1 | Variation_2915 | chr11:32792784..32958609 |
| OR5AS1 | Variation_2927 | chr11:55689863..55849156 |
| CHKA | Variation_2931 | chr11:67472905..67891289 |
| PDZRN4 | Variation_2982 | chr12:41780758..41816002 |
| STRC | Variation_3079 | chr15:43831923..44058634 |
| SLCO3A1 | Variation_3095 | chr15:92581551..92615246 |
| PCSK6 | Variation_3099 | chr15:101720143..102022334 |
| MRM1 | Variation_3143 | chr17:34944593..35030559 |
| CTNNBL1 | Variation_3216 | chr20:36302612..36326562 |
| MYO18B | Variation_3240 | chr22:26135889..26150584 |
| ABI3BP | Variation_3440 | chr3:100613387..100776497 |
| CLSTN2 | Variation_3450 | chr3:139925764..140524730 |
| HRASLS | Variation_3470 | chr3:192796377..192967734 |
| TMEM129 | Variation_3476 | chr4:1625392..1814775 |
| ZNF518B | Variation_3480 | chr4:10343699..10545800 |
| SPOCK3 | Variation_3525 | chr4:167891496..168190991 |
| KIAA0947 | Variation_3540 | chr5:5447098..5764620 |
| MDFI | Variation_3609 | chr6:41588789..41699453 |
| BAI3 | Variation_3619 | chr6:69595492..69598351 |
| PARK2 | Variation_3649 | chr6:162190756..162411250 |
| PARK2 | Variation_3650 | chr6:162858322..163140375 |
| SNORD93 | Variation_3670 | chr7:22755584..22920910 |
| PDGFRL | Variation_3724 | chr8:17291180..17641530 |
| MMP16 | Variation_3739 | chr8:88963829..89133704 |
| CYHR1 | Variation_3752 | chr8:145523693..145697678 |
| MAMDC4 | Variation_3785 | chr9:139250829..139826485 |
| EXD3 | Variation_3786 | chr9:140197784..140341751 |
| PCDH15 | Variation_3801 | chr10:55360612..55586930 |
| PCDH15 | Variation_3802 | chr10:56408027..56566137 |
| SNORD98 | Variation_3808 | chr10:70457719..70631312 |
| C10orf11 | Variation_3811 | chr10:77420559..77770449 |
| BCCIP | Variation_3825 | chr10:127453900..127786702 |
| DOCK1 | Variation_3826 | chr10:128908525..129239022 |
| NUP98 | Variation_3833 | chr11:3236512..3801429 |
| QSER1 | Variation_3843 | chr11:32714643..33049940 |
| CHKA | Variation_3856 | chr11:67524937..67859490 |
| ARID2 | Variation_3887 | chr12:46149338..46349891 |
| LMO7 | Variation_3914 | chr13:76321610..76542003 |
| STRC | Variation_3960 | chr15:43823158..44148641 |
| PCSK6 | Variation_3983 | chr15:101732816..101916486 |
| PCSK6 | Variation_3984 | chr15:101889682..102055455 |
| MRM1 | Variation_4032 | chr17:34962048..35147394 |
| NPEPPS | Variation_4039 | chr17:45520374..45764956 |
| RAD51C | Variation_4042 | chr17:56642376..56794949 |
| AKT2 | Variation_4080 | chr19:40612420..40839520 |
| C22orf25 | Variation_4117 | chr22:20026493..20309447 |
| CACNG2 | Variation_4126 | chr22:36906563..37042034 |
| KIAA1644 | Variation_4130 | chr22:44643821..44701301 |
| TSGA10 | Variation_4301 | chr2:99533833..99709999 |
| NCKAP5 | Variation_4308 | chr2:133874257..133906766 |
| ZDHHC3 | Variation_4333 | chr3:44970199..45190848 |
| ALCAM | Variation_4348 | chr3:105070894..105255030 |
| HPS3 | Variation_4355 | chr3:148807113..148853218 |
| MED12L | Variation_4356 | chr3:150692637..150889738 |
| RAD23A | Variation_4400 | chr19:12847630..13058491 |
| DNAH11 | Variation_4527 | chr7:21489100..21688016 |
| CYHR1 | Variation_4613 | chr8:145565803..145769410 |
| CER1 | Variation_4615 | chr9:14596657..14739902 |
| ZMIZ1 | Variation_4704 | chr10:80952259..81076777 |
| LHPP | Variation_4715 | chr10:126246143..126465030 |
| STK32C | Variation_4717 | chr10:133926469..134072099 |
| DEAF1 | Variation_4725 | chr11:631027..758602 |
| CELF1 | Variation_4741 | chr11:47517955..47681310 |
| PATL1 | Variation_4746 | chr11:59396407..59431115 |
| NRXN2 | Variation_4752 | chr11:64315369..64485209 |
| MEN1 | Variation_4753 | chr11:64487183..64620199 |
| PC | Variation_4754 | chr11:66634462..66805980 |
| FGF4 | Variation_4755 | chr11:69423683..69588478 |
| PDZRN4 | Variation_4776 | chr12:41776841..41981332 |
| MED13L | Variation_4787 | chr12:116475925..116648486 |
| CCDC122 | Variation_4801 | chr13:44290974..44499299 |
| ANKRD9 | Variation_4847 | chr14:102903636..103223539 |
| PSTPIP1 | Variation_4905 | chr15:77235896..77393640 |
| TRAPPC2L | Variation_4975 | chr16:88833868..89011311 |
| TRAPPC2L | Variation_4976 | chr16:88846302..89033052 |
| AATF | Variation_5001 | chr17:35166406..35381639 |
| CDC27 | Variation_5015 | chr17:45063935..45252624 |
| NGFR | Variation_5016 | chr17:47456771..47628470 |
| CACNG4 | Variation_5027 | chr17:64855019..65023735 |
| DNMT3B | Variation_5133 | chr20:31343123..31495798 |
| CTNNBL1 | Variation_5136 | chr20:36445761..36537911 |
| C22orf25 | Variation_5168 | chr22:19887966..20069970 |
| KLHL22 | Variation_5169 | chr22:20759359..20951242 |
| TXN2 | Variation_5183 | chr22:36731924..36906812 |
| SLC16A8 | Variation_5185 | chr22:38388465..38549017 |
| TMEM184B | Variation_5186 | chr22:38459503..38636510 |
| UROC1 | Variation_5213 | chr3:126185386..126236700 |
| PARK2 | Variation_5226 | chr6:162049757..162202795 |
| PARK2 | Variation_5228 | chr6:162523835..162805800 |
| EFTUD1 | Variation_5326 | chr15:82293064..82426400 |
| PCSK6 | Variation_5327 | chr15:101887391..102134120 |
| MSI2 | Variation_5335 | chr17:55496728..55523315 |
| EVC2 | Variation_5377 | chr4:5582866..5647287 |
| SNRNP40 | Variation_5483 | chr1:31731974..31741142 |
| ZNF644 | Variation_5506 | chr1:91436237..91442512 |
| KIAA1274 | Variation_5602 | chr10:72283011..72286387 |
| RBM20 | Variation_5612 | chr10:112563598..112568976 |
| ATRNL1 | Variation_5614 | chr10:117497668..117504642 |
| QSER1 | Variation_5624 | chr11:32986328..32987490 |
| PHF21A | Variation_5625 | chr11:45993457..45998686 |
| NAA25 | Variation_5691 | chr12:112472575..112476739 |
| KSR2 | Variation_5698 | chr12:118179174..118186925 |
| NOVA1 | Variation_5730 | chr14:26941944..26951194 |
| TP53BP1 | Variation_5779 | chr15:43774977..43777774 |
| FRMD5 | Variation_5780 | chr15:44385556..44390318 |
| PIAS1 | Variation_5792 | chr15:68426021..68428945 |
| KIAA1199 | Variation_5798 | chr15:81124703..81131630 |
| MYCBPAP | Variation_5868 | chr17:48600071..48607858 |
| TOM1L1 | Variation_5869 | chr17:53029916..53036091 |
| GNA13 | Variation_5873 | chr17:63034829..63044439 |
| PITPNC1 | Variation_5875 | chr17:65679306..65684454 |
| CDC42EP4 | Variation_5879 | chr17:71293145..71295593 |
| CDC42EP4 | Variation_5880 | chr17:71299811..71305177 |
| TSHZ1 | Variation_5924 | chr18:72943502..72949746 |
| USP39 | Variation_5988 | chr2:85860999..85866615 |
| PMS1 | Variation_6016 | chr2:190726489..190729383 |
| ALS2CR8 | Variation_6021 | chr2:203845386..203851591 |
| AIFM3 | Variation_6143 | chr22:21327983..21332394 |
| YPEL1 | Variation_6144 | chr22:22052566..22058785 |
| SETD5 | Variation_6155 | chr3:9441529..9448291 |
| ATG7 | Variation_6158 | chr3:11416397..11420159 |
| ATG7 | Variation_6159 | chr3:11416531..11420159 |
| LRRC58 | Variation_6206 | chr3:120066250..120069861 |
| ZXDC | Variation_6208 | chr3:126181918..126190563 |
| MAP3K13 | Variation_6223 | chr3:185113165..185118693 |
| TPRG1 | Variation_6224 | chr3:188995493..189000004 |
| MUC4 | Variation_6229 | chr3:195464997..195473757 |
| EREG | Variation_6279 | chr4:75229227..75231385 |
| HSD17B13 | Variation_6291 | chr4:88238744..88241534 |
| PARK2 | Variation_6523 | chr6:162177505..162181256 |
| ESCO2 | Variation_6600 | chr8:27660151..27662804 |
| BAG4 | Variation_6611 | chr8:38033200..38038040 |
| FREM1 | Variation_6680 | chr9:14778629..14780431 |
| SYK | Variation_6694 | chr9:93638584..93643961 |
| SYK | Variation_6695 | chr9:93638589..93643961 |
| C9orf86 | Variation_6727 | chr9:139706126..139711197 |
| C10orf26 | Variation_6843 | chr10:104540462..104542935 |
| FANK1 | Variation_6849 | chr10:127613562..127617477 |
| MGMT | Variation_6850 | chr10:131429608..131430611 |
| TCERG1L | Variation_6851 | chr10:132909251..132912646 |
| BMS1 | Variation_6863 | chr10:43328713..43330061 |
| WDFY4 | Variation_6869 | chr10:50056451..50059418 |
| PCDH15 | Variation_6874 | chr10:56448656..56469572 |
| ZNF365 | Variation_6877 | chr10:64257169..64258705 |
| C10orf11 | Variation_6880 | chr10:77850968..77867169 |
| C10orf11 | Variation_6881 | chr10:78247916..78258692 |
| ZMIZ1 | Variation_6886 | chr10:81003814..81004851 |
| NRG3 | Variation_6889 | chr10:83883784..83889019 |
| SHANK2 | Variation_6937 | chr11:70875083..70932650 |
| TMEM41B | Variation_6956 | chr11:9311131..9328869 |
| STRC | Variation_7061 | chr15:43888955..43995223 |
| CPEB1 | Variation_7075 | chr15:83314833..83317588 |
| CES1 | Variation_7118 | chr16:55843003..55865135 |
| EXOSC6 | Variation_7125 | chr16:70116599..70319873 |
| SPIRE2 | Variation_7140 | chr16:89896969..89898343 |
| MSI2 | Variation_7167 | chr17:55090067..55793467 |
| BCAS3 | Variation_7169 | chr17:58880977..58884827 |
| NPAS1 | Variation_7208 | chr19:47518768..47524624 |
| FAM178B | Variation_7289 | chr2:97616488..97643975 |
| REV1 | Variation_7294 | chr2:100103969..100105017 |
| IGSF11 | Variation_7355 | chr3:118726577..118803600 |
| EEFSEC | Variation_7359 | chr3:127842954..127911683 |
| RPN1 | Variation_7360 | chr3:128330104..128338813 |
| ANAPC13 | Variation_7364 | chr3:134201034..134210747 |
| ZDHHC19 | Variation_7400 | chr3:195916045..195940827 |
| FAM149A | Variation_7436 | chr4:187093564..187098613 |
| PDE10A | Variation_7545 | chr6:166073581..166078754 |
| EXD3 | Variation_7733 | chr9:140230789..140240785 |
| EXD3 | Variation_7734 | chr9:140242333..140246558 |
| TGFBR3 | Variation_8323 | chr1:92356781..92579675 |
| TFG | Variation_8426 | chr3:100342493..100493062 |
| TFG | Variation_8427 | chr3:100299310..100479384 |
| TFG | Variation_8428 | chr3:100342310..100442478 |
| TRIM42 | Variation_8429 | chr3:140009020..140469302 |
| PARK2 | Variation_8531 | chr6:161592100..161855785 |
| PARK2 | Variation_8532 | chr6:162321377..162709948 |
| PARK2 | Variation_8533 | chr6:162507589..162917677 |
| PARK2 | Variation_8534 | chr6:162578792..162709948 |
| PARK2 | Variation_8535 | chr6:162643427..162899571 |
| PARK2 | Variation_8536 | chr6:162731343..162951589 |
| PARK2 | Variation_8537 | chr6:162827464..163057269 |
| LRRTM3 | Variation_8660 | chr10:68689567..68938294 |
| C10orf11 | Variation_8661 | chr10:77555491..77736622 |
| NRG3 | Variation_8664 | chr10:84389603..84437220 |
| BTRC | Variation_8666 | chr10:103054982..103452645 |
| SYCE1 | Variation_8670 | chr10:135269955..135434551 |
| SYCE1 | Variation_8672 | chr10:135324909..135434551 |
| SYCE1 | Variation_8674 | chr10:135371105..135422505 |
| C15orf58 | Variation_8803 | chr15:90622504..90792506 |
| GGNBP2 | Variation_8843 | chr17:34435487..35081387 |
| KIAA1267 | Variation_8848 | chr17:44094471..44364056 |
| KIAA1267 | Variation_8849 | chr17:44187492..44364056 |
| KIAA1267 | Variation_8851 | chr17:44039564..44364056 |
| CRYBB2 | Variation_8903 | chr22:25607785..25962988 |
| PARK2 | Variation_9041 | chr6:162270981..162314554 |
| PARK2 | Variation_9042 | chr6:162709589..162827464 |
| PDGFRL | Variation_9089 | chr8:17346029..17583151 |
| BMS1 | Variation_9147 | chr10:43116194..43329770 |
| PCDH15 | Variation_9154 | chr10:54732555..57315135 |
| NRG3 | Variation_9166 | chr10:84538744..84618651 |
| SYCE1 | Variation_9170 | chr10:135269955..135388119 |
| OR5AS1 | Variation_9189 | chr11:55685799..55842512 |
| UCHL3 | Variation_9224 | chr13:76144199..76288418 |
| KIAA1267 | Variation_9289 | chr17:44157597..44364056 |
| KIAA1267 | Variation_9290 | chr17:44213204..44364056 |
| CLSTN2 | Variation_9455 | chr3:140014763..140182046 |
| TRIM42 | Variation_9456 | chr3:140330108..140454836 |
| CCDC50 | Variation_9464 | chr3:191065921..191069984 |
| DLG1 | Variation_9465 | chr3:197018125..197026927 |
| PDE10A | Variation_9553 | chr6:165877431..165893347 |
| SEMA3E | Variation_9577 | chr7:83193369..83208702 |
| TRAF2 | Variation_9631 | chr9:139634495..139904037 |
| NRG3 | Variation_9644 | chr10:84420273..84430088 |
| SYCE1 | Variation_9647 | chr10:135352013..135434303 |
| SYCE1 | Variation_9648 | chr10:135266389..135377448 |
| PDZRN4 | Variation_9693 | chr12:41784997..41811063 |
| EDC3 | Variation_9749 | chr15:74889163..75019449 |
| C15orf58 | Variation_9752 | chr15:90647856..90784930 |
| KIAA1267 | Variation_9775 | chr17:44169808..44350090 |
| KIAA1267 | Variation_9776 | chr17:44169808..44250108 |
| C1orf150 | Variation_9917 | chr1:247715998..247728962 |
| TGOLN2 | Variation_9953 | chr2:85547429..85555262 |
| KBTBD10 | Variation_9969 | chr2:170354790..170368798 |
| EPHB1 | Variation_10009 | chr3:134733516..134736671 |
| CCDC50 | Variation_10028 | chr3:191065392..191067244 |
| CCDC50 | Variation_10029 | chr3:191065392..191069984 |
| CCDC50 | Variation_10030 | chr3:191066878..191069984 |
| CCDC50 | Variation_10031 | chr3:191068288..191069984 |
| SLIT2 | Variation_10039 | chr4:20557459..20565861 |
| PARK2 | Variation_10169 | chr6:162499177..162506568 |
| PARK2 | Variation_10170 | chr6:162736336..162739765 |
| PARK2 | Variation_10171 | chr6:162738705..162739765 |
| PCLO | Variation_10198 | chr7:82501290..82524663 |
| PDGFRL | Variation_10248 | chr8:17438439..17446444 |
| CNGB3 | Variation_10261 | chr8:87643741..87658739 |
| PCDH15 | Variation_10327 | chr10:56084933..56093271 |
| PCDH15 | Variation_10328 | chr10:56239430..56273600 |
| STOX1 | Variation_10334 | chr10:70616199..70618859 |
| CDH23 | Variation_10335 | chr10:73322629..73328006 |
| NRG3 | Variation_10337 | chr10:84713118..84716523 |
| LHPP | Variation_10343 | chr10:126189866..126192376 |
| SHANK2 | Variation_10370 | chr11:70476810..70496641 |
| CATSPER2 | Variation_10482 | chr15:43914373..43939642 |
| FAH | Variation_10487 | chr15:80453730..80460524 |
| EVPL | Variation_10520 | chr17:74002725..74009211 |
| KREMEN1 | Variation_10622 | chr22:29540297..29568963 |
| UQCR10 | Variation_10623 | chr22:30163526..30169436 |
| RNF103 | Variation_22520 | chr2:86849862..86854021 |
| EPHB1 | Variation_22574 | chr3:134794104..134799571 |
| EMID1 | Variation_22584 | chr22:29632636..29638282 |
| FAM149A | Variation_22588 | chr4:187092843..187098549 |
| SPOCK3 | Variation_22657 | chr4:167676709..167683197 |
| TTC39B | Variation_22662 | chr9:15256031..15262575 |
| SPOCK3 | Variation_22712 | chr4:168108868..168115773 |
| NRG3 | Variation_22723 | chr10:84127603..84134632 |
| SPOCK3 | Variation_22948 | chr4:167892127..168023795 |
| FAM149A | Variation_23016 | chr4:187093484..187098615 |
| C10orf11 | Variation_23049 | chr10:78255293..78261315 |
| SPOCK3 | Variation_23152 | chr4:167675509..167683269 |
| CCDC50 | Variation_23177 | chr3:191064550..191073833 |
| TM4SF4 | Variation_23277 | chr3:149151302..149202302 |
| KIAA1267 | Variation_23317 | chr17:44208144..44364056 |
| TCERG1L | Variation_23381 | chr10:132975174..132975174 |
| RIC3 | Variation_23425 | chr11:8180329..8180329 |
| SCUBE2 | Variation_23426 | chr11:9104550..9104550 |
| PIAS1 | Variation_23499 | chr15:68426020..68428945 |
| SLC39A11 | Variation_23528 | chr17:71047900..71047900 |
| SDK2 | Variation_23529 | chr17:71401349..71401349 |
| TBCD | Variation_23537 | chr17:80759992..80759992 |
| TMEM163 | Variation_23584 | chr2:135365259..135366938 |
| TANC1 | Variation_23586 | chr2:159959177..159961446 |
| BAIAP2L2 | Variation_23652 | chr22:38506061..38506061 |
| SLC12A8 | Variation_23661 | chr3:124808629..124808629 |
| CLSTN2 | Variation_23665 | chr3:140268047..140269460 |
| C1orf94 | Variation_29576 | chr1:34420696..34924596 |
| SYCE1 | Variation_29599 | chr10:135266388..135377448 |
| SYCE1 | Variation_29600 | chr10:135352013..135434303 |
| ECHS1 | Variation_29601 | chr10:135178611..135225665 |
| KIAA1267 | Variation_29671 | chr17:44140748..44350089 |
| ICAM3 | Variation_29685 | chr19:10399904..10471977 |
| ICAM3 | Variation_29686 | chr19:10435493..10472933 |
| TFG | Variation_29730 | chr3:100350745..100433592 |
| IGSF11 | Variation_29731 | chr3:118729114..118801854 |
| DLG1 | Variation_29732 | chr3:196910082..197348090 |
| PARK2 | Variation_29776 | chr6:162709948..162973214 |
| CDH23 | Variation_29865 | chr10:73447281..73509465 |
| ZMIZ1 | Variation_29866 | chr10:80907147..80949553 |
| ZMIZ1 | Variation_29867 | chr10:80940357..80959517 |
| GPR123 | Variation_29875 | chr10:134846982..134917297 |
| GPR123 | Variation_29876 | chr10:134873188..134931784 |
| ECHS1 | Variation_29878 | chr10:135053021..135223018 |
| OR5AS1 | Variation_29912 | chr11:55685556..55819544 |
| TPCN2 | Variation_29915 | chr11:68804451..68899814 |
| TRAPPC2L | Variation_30015 | chr16:88868252..88927221 |
| TRAPPC2L | Variation_30016 | chr16:88877429..89002398 |
| C22orf25 | Variation_30151 | chr22:20006040..20032327 |
| C22orf25 | Variation_30152 | chr22:20006040..20046687 |
| ZDHHC8 | Variation_30155 | chr22:20100596..20189077 |
| LZTR1 | Variation_30158 | chr22:21331556..21353328 |
| CYTH4 | Variation_30163 | chr22:37708004..37753999 |
| EEFSEC | Variation_30182 | chr3:128083332..128159359 |
| SLBP | Variation_30192 | chr4:1563920..1724579 |
| TNIP2 | Variation_30194 | chr4:2743999..2836628 |
| DOK7 | Variation_30196 | chr4:3493215..3584373 |
| HSD17B13 | Variation_30208 | chr4:88186509..88228228 |
| COL19A1 | Variation_30237 | chr6:70654657..70767756 |
| COL19A1 | Variation_30238 | chr6:70736585..70767756 |
| PARK2 | Variation_30245 | chr6:162732259..162946697 |
| PARK2 | Variation_30246 | chr6:162920373..162976039 |
| DENND3 | Variation_30286 | chr8:142192569..142241238 |
| SLC39A4 | Variation_30308 | chr8:145641564..145668443 |
| SLC39A4 | Variation_30309 | chr8:145641564..145690308 |
| TRAF2 | Variation_30359 | chr9:139534703..139823670 |
| SNRNP40 | Variation_30381 | chr1:31734840..31736134 |
| BMS1 | Variation_30483 | chr10:43327343..43354456 |
| SGMS1 | Variation_30501 | chr10:52329526..52345673 |
| C10orf11 | Variation_30511 | chr10:77544669..77701506 |
| NRG3 | Variation_30513 | chr10:84409674..84431225 |
| ATRNL1 | Variation_30517 | chr10:117486948..117489254 |
| DOCK1 | Variation_30522 | chr10:129047461..129048528 |
| SYCE1 | Variation_30527 | chr10:135252350..135505981 |
| PDDC1 | Variation_30529 | chr11:765282..770250 |
| PPP1R13B | Variation_30664 | chr14:104249553..104251521 |
| STRC | Variation_30709 | chr15:43887856..43892278 |
| PCSK6 | Variation_30723 | chr15:101847075..101849708 |
| PCSK6 | Variation_30724 | chr15:101987050..101990302 |
| CES1 | Variation_30784 | chr16:55840672..55865687 |
| NPEPPS | Variation_30840 | chr17:45616222..45670569 |
| TBKBP1 | Variation_30841 | chr17:45772563..45776329 |
| AZI1 | Variation_30855 | chr17:79165395..79171140 |
| DHX34 | Variation_30911 | chr19:47860455..47930044 |
| RMND5A | Variation_30957 | chr2:86944160..87024895 |
| C22orf25 | Variation_31071 | chr22:19019088..21053198 |
| SPECC1L | Variation_31080 | chr22:24670373..24674855 |
| CRYBB2 | Variation_31083 | chr22:25603009..25642424 |
| CACNG2 | Variation_31091 | chr22:36959215..36960450 |
| CACNG2 | Variation_31092 | chr22:36972708..36976431 |
| PHF21B | Variation_31095 | chr22:45379553..45566540 |
| CCDC50 | Variation_31132 | chr3:191064972..191071425 |
| CCDC50 | Variation_31133 | chr3:191076410..191077591 |
| MUC4 | Variation_31138 | chr3:195499435..195515976 |
| DLG1 | Variation_31141 | chr3:196934071..196935649 |
| LMLN | Variation_31145 | chr3:197730574..197732130 |
| LMLN | Variation_31146 | chr3:197735248..197742554 |
| MFSD7 | Variation_31150 | chr4:668081..683843 |
| DOK7 | Variation_31154 | chr4:3468325..3474416 |
| DOK7 | Variation_31155 | chr4:3479973..3565798 |
| KIAA1324L | Variation_31373 | chr7:86664485..86681120 |
| PTK2 | Variation_31454 | chr8:142002775..142004294 |
| HEATR7A | Variation_31456 | chr8:145191472..145218025 |
| EXD3 | Variation_31537 | chr9:140197941..140215364 |
| NELF | Variation_31538 | chr9:140345585..140346739 |
| BMS1 | Variation_31724 | chr10:43325621..43330060 |
| PCDH15 | Variation_31743 | chr10:55925500..56022613 |
| PCDH15 | Variation_31744 | chr10:56448655..56468925 |
| STOX1 | Variation_31753 | chr10:70613310..70620580 |
| HTRA1 | Variation_31777 | chr10:124258479..124262098 |
| BCCIP | Variation_31779 | chr10:127511987..127513629 |
| BCCIP | Variation_31780 | chr10:127522580..127524647 |
| FANK1 | Variation_31781 | chr10:127572676..127608463 |
| GPR123 | Variation_31784 | chr10:134925262..134927817 |
| KNDC1 | Variation_31785 | chr10:135035825..135037303 |
| ART5 | Variation_31799 | chr11:3663343..3664373 |
| PPIP5K1 | Variation_32025 | chr15:43856079..43857117 |
| PPIP5K1 | Variation_32026 | chr15:43862667..43864034 |
| STRC | Variation_32028 | chr15:43893456..43895669 |
| CATSPER2 | Variation_32029 | chr15:43911140..43952044 |
| SPIRE2 | Variation_32144 | chr16:89897068..89901057 |
| NPEPPS | Variation_32186 | chr17:45615186..45670569 |
| NCK2 | Variation_32339 | chr2:106388795..106390908 |
| UBR3 | Variation_32382 | chr2:170649846..170957968 |
| SPECC1L | Variation_32455 | chr22:24670647..24673081 |
| CLSTN2 | Variation_32520 | chr3:140267930..140270484 |
| MED12L | Variation_32525 | chr3:150922607..150924281 |
| CCDC50 | Variation_32544 | chr3:191064972..191071425 |
| MUC4 | Variation_32550 | chr3:195502287..195504432 |
| LMLN | Variation_32559 | chr3:197730574..197732130 |
| MAPK10 | Variation_32620 | chr4:86976579..86979876 |
| PARK2 | Variation_32845 | chr6:162193402..162199443 |
| PARK2 | Variation_32846 | chr6:162365883..162368722 |
| PARK2 | Variation_32847 | chr6:162496656..162506160 |
| PARK2 | Variation_32848 | chr6:162734952..162740385 |
| PARK2 | Variation_32849 | chr6:162917902..162924944 |
| PDGFRL | Variation_33020 | chr8:17437009..17444558 |
| EXD3 | Variation_33156 | chr9:140244909..140248359 |
| TFG | Variation_34429 | chr3:100251674..100442478 |
| LRRTM3 | Variation_34457 | chr10:68726829..68952894 |
| UBQLN3 | Variation_34458 | chr11:5505764..6194475 |
| KIAA1267 | Variation_34480 | chr17:44094463..44364056 |
| PARK2 | Variation_34494 | chr6:162709948..162943061 |
| TFG | Variation_34513 | chr3:100299329..100455310 |
| KIAA1267 | Variation_34553 | chr17:44204373..44364056 |
| SYCE1 | Variation_34564 | chr10:135269955..135434551 |
| CRYBB2 | Variation_34568 | chr22:25606861..25953851 |
| KIAA1267 | Variation_34574 | chr17:44115107..44364056 |
| KIAA1267 | Variation_34651 | chr17:44213204..44364056 |
| KIAA1267 | Variation_34652 | chr17:44166500..44364056 |
| CRYBB2 | Variation_34657 | chr22:25593658..25931372 |
| DOCK1 | Variation_34862 | chr10:128766451..128791151 |
| TCERG1L | Variation_34863 | chr10:132880134..132915426 |
| C10orf11 | Variation_34892 | chr10:78243200..78265460 |
| AGBL2 | Variation_34967 | chr11:47653723..47681660 |
| GLYAT | Variation_34989 | chr11:58451044..58478290 |
| MRPL21 | Variation_35004 | chr11:68643810..68664735 |
| MRPL21 | Variation_35005 | chr11:68648189..68662347 |
| CCDC64 | Variation_35050 | chr12:120492746..120499127 |
| PXN | Variation_35051 | chr12:120681848..120699478 |
| MSRB3 | Variation_35107 | chr12:65687074..65689885 |
| UCHL3 | Variation_35184 | chr13:76116377..76139954 |
| STRC | Variation_35314 | chr15:43878745..43972533 |
| STRC | Variation_35315 | chr15:43878812..43996580 |
| PSTPIP1 | Variation_35346 | chr15:77321714..77344266 |
| PSTPIP1 | Variation_35347 | chr15:77325395..77333596 |
| PSTPIP1 | Variation_35348 | chr15:77326851..77342409 |
| HOMER2 | Variation_35349 | chr15:83546438..83565430 |
| SLCO3A1 | Variation_35352 | chr15:92622348..92625190 |
| SLCO3A1 | Variation_35353 | chr15:92644907..92648729 |
| SLC39A11 | Variation_35490 | chr17:70837614..70858989 |
| SLC39A11 | Variation_35491 | chr17:70841590..70862983 |
| SLC39A11 | Variation_35492 | chr17:70842594..70851643 |
| SLC39A11 | Variation_35493 | chr17:70849835..70879053 |
| SLC39A11 | Variation_35494 | chr17:70851398..70856825 |
| SLC39A11 | Variation_35495 | chr17:70858110..70860649 |
| SLC39A11 | Variation_35496 | chr17:70868585..70880241 |
| SLC39A11 | Variation_35497 | chr17:70868774..70874444 |
| SLC39A11 | Variation_35498 | chr17:70873903..70879704 |
| KATNAL2 | Variation_35546 | chr18:44544654..44545406 |
| KATNAL2 | Variation_35547 | chr18:44545444..44547308 |
| KATNAL2 | Variation_35548 | chr18:44546279..44547650 |
| KATNAL2 | Variation_35549 | chr18:44546539..44547595 |
| KATNAL2 | Variation_35553 | chr18:44550838..44552021 |
| KATNAL2 | Variation_35554 | chr18:44551540..44553665 |
| KATNAL2 | Variation_35555 | chr18:44551603..44552919 |
| KATNAL2 | Variation_35556 | chr18:44551625..44552767 |
| KATNAL2 | Variation_35557 | chr18:44551905..44553391 |
| KATNAL2 | Variation_35558 | chr18:44551977..44553109 |
| KATNAL2 | Variation_35559 | chr18:44552652..44553547 |
| KATNAL2 | Variation_35560 | chr18:44552813..44553831 |
| KATNAL2 | Variation_35561 | chr18:44553083..44553958 |
| ZRANB3 | Variation_35735 | chr2:136063865..136102818 |
| NOSTRIN | Variation_35753 | chr2:169715542..169736964 |
| NOSTRIN | Variation_35754 | chr2:169716824..169729315 |
| NOSTRIN | Variation_35755 | chr2:169717652..169729930 |
| NOSTRIN | Variation_35756 | chr2:169721323..169739365 |
| NOSTRIN | Variation_35757 | chr2:169721583..169730071 |
| PPIG | Variation_35759 | chr2:170465703..170500395 |
| KLHL22 | Variation_35983 | chr22:20833432..20865784 |
| IFT27 | Variation_36022 | chr22:37128019..37155733 |
| IFT27 | Variation_36023 | chr22:37134943..37159320 |
| PLA2G6 | Variation_36026 | chr22:38501542..38509808 |
| MKL1 | Variation_36031 | chr22:40929339..40953204 |
| SLC12A8 | Variation_36049 | chr3:124796993..124825412 |
| RUVBL1 | Variation_36051 | chr3:127836898..127859710 |
| RUVBL1 | Variation_36052 | chr3:127841784..127860588 |
| RUVBL1 | Variation_36053 | chr3:127842301..127863586 |
| EEFSEC | Variation_36055 | chr3:127852329..127882129 |
| TMEM108 | Variation_36069 | chr3:133015463..133032477 |
| MED12L | Variation_36082 | chr3:151141432..151150361 |
| FETUB | Variation_36114 | chr3:186343494..186372743 |
| DLG1 | Variation_36126 | chr3:196933153..196969565 |
| SPOCK3 | Variation_36223 | chr4:167666854..167705354 |
| FAM149A | Variation_36241 | chr4:187086427..187108530 |
| PARK2 | Variation_36474 | chr6:162152970..162186558 |
| PARK2 | Variation_36475 | chr6:162159996..162186581 |
| CD2AP | Variation_36528 | chr6:47419871..47450466 |
| CD2AP | Variation_36530 | chr6:47426510..47458642 |
| BAI3 | Variation_36550 | chr6:69670701..69701360 |
| CDH17 | Variation_36895 | chr8:95172139..95205021 |
| MAMDC2 | Variation_36963 | chr9:72772931..72788972 |
| MAMDC2 | Variation_36964 | chr9:72839970..72858579 |
| CCDC63 | Variation_37130 | chr12:111289607..111369677 |
| CCDC64 | Variation_37131 | chr12:120498481..120509020 |
| PXN | Variation_37132 | chr12:120664040..120682523 |
| VPS37B | Variation_37133 | chr12:123346525..123371767 |
| HEATR5A | Variation_37156 | chr14:31759603..31786517 |
| BTBD1 | Variation_37171 | chr15:83719676..83747141 |
| ZRANB3 | Variation_37413 | chr2:136098341..136101573 |
| DOK7 | Variation_37548 | chr4:3467519..3485786 |
| RPN1 | Variation_37562 | chr3:128364797..128412717 |
| KIAA1267 | Variation_37588 | chr17:44165803..44353885 |
| PCDH15 | Variation_37657 | chr10:56452949..56468437 |
| NRG3 | Variation_37660 | chr10:84410727..84431840 |
| C15orf58 | Variation_37691 | chr15:90632610..90778393 |
| ZRANB3 | Variation_37713 | chr2:136192779..136232551 |
| SYCE1 | Variation_37785 | chr10:135328663..135377278 |
| CES1 | Variation_37794 | chr16:55842382..55862710 |
| DOK7 | Variation_37806 | chr4:3469444..3473635 |
| SLIT2 | Variation_37871 | chr4:20556711..20563942 |
| EPHB1 | Variation_37881 | chr3:134842625..134844843 |
| PCLO | Variation_37933 | chr7:82715960..82717964 |
| STOX1 | Variation_38207 | chr10:70612644..70622117 |
| NRG3 | Variation_38210 | chr10:83884661..83888667 |
| KSR2 | Variation_38246 | chr12:118049324..118051527 |
| SLC39A11 | Variation_38362 | chr17:70792558..70794461 |
| NCKAP5 | Variation_38403 | chr2:133473528..133476565 |
| ABI3BP | Variation_38464 | chr3:100669517..100673226 |
| MAP3K13 | Variation_38483 | chr3:185101422..185107017 |
| MTHFD2L | Variation_38568 | chr4:75081112..75082206 |
| MAPK10 | Variation_38575 | chr4:86976094..86979944 |
| PARK2 | Variation_38631 | chr6:162496291..162503734 |
| PARK2 | Variation_38632 | chr6:162738568..162740440 |
| SEMA3A | Variation_38690 | chr7:83782433..83786688 |
| KIAA1267 | Variation_38756 | chr17:44165801..44364214 |
| C10orf11 | Variation_38792 | chr10:78257061..78260585 |
| NRG3 | Variation_38858 | chr10:84712898..84717172 |
| STRC | Variation_38875 | chr15:43892841..43894806 |
| KIAA1267 | Variation_38979 | chr17:44165726..44289261 |
| TGFBR3 | Variation_39002 | chr1:92232061..92233333 |
| C10orf11 | Variation_39056 | chr10:78255572..78261019 |
| CCDC84 | Variation_39090 | chr11:118876785..118884012 |
| SETD8 | Variation_39118 | chr12:123888177..123889428 |
| SETD8 | Variation_39119 | chr12:123889620..123892038 |
| TSC22D1 | Variation_39132 | chr13:45120911..45125551 |
| PIBF1 | Variation_39139 | chr13:73468909..73476134 |
| SCEL | Variation_39141 | chr13:78167454..78170851 |
| HECTD1 | Variation_39150 | chr14:31591492..31603748 |
| PIAS1 | Variation_39170 | chr15:68426020..68428945 |
| CDC27 | Variation_39202 | chr17:45259002..45266510 |
| MSI2 | Variation_39205 | chr17:55427616..55435581 |
| USP39 | Variation_39256 | chr2:85848998..85860985 |
| REV1 | Variation_39258 | chr2:100103713..100105031 |
| NR1I2 | Variation_39343 | chr3:119506510..119516033 |
| SPOCK3 | Variation_39402 | chr4:167677048..167683083 |
| BAI3 | Variation_39456 | chr6:69642782..69650510 |
| TMEM64 | Variation_39530 | chr8:91617265..91638876 |
| SLIT2 | Variation_43239 | chr4:20407510..20408609 |
| ABI3BP | Variation_43240 | chr3:100521542..100522642 |
| REV1 | Variation_43318 | chr2:100103677..100105127 |
| C12orf51 | Variation_43337 | chr12:112670239..112671778 |
| CDC27 | Variation_43514 | chr17:45215735..45218794 |
| PIAS1 | Variation_43517 | chr15:68425983..68429067 |
| CDC27 | Variation_43548 | chr17:45245033..45248949 |
| TCERG1L | Variation_43550 | chr10:132909017..132912946 |
| C10orf11 | Variation_43586 | chr10:78255543..78261101 |
| SPOCK3 | Variation_43623 | chr4:167677004..167683216 |
| BAI3 | Variation_43641 | chr6:69818249..69825384 |
| CDC27 | Variation_43644 | chr17:45258973..45266558 |
| CDC27 | Variation_43661 | chr17:45223249..45233036 |
| CDC27 | Variation_43672 | chr17:45247093..45261518 |
| EVI5 | Variation_43784 | chr1:93236031..93241462 |
| C10orf11 | Variation_43804 | chr10:78255582..78261017 |
| NRG3 | Variation_43808 | chr10:84127823..84130366 |
| SETD8 | Variation_43839 | chr12:123875288..123879611 |
| PIAS1 | Variation_43913 | chr15:68426018..68428925 |
| FAM104A | Variation_43954 | chr17:71216616..71217679 |
| ZRANB3 | Variation_44001 | chr2:136098324..136101576 |
| REV1 | Variation_44041 | chr2:100103699..100105033 |
| PARVB | Variation_44073 | chr22:44508563..44510147 |
| PARVB | Variation_44074 | chr22:44510738..44511933 |
| ABI3BP | Variation_44078 | chr3:100669307..100670859 |
| SPOCK3 | Variation_44139 | chr4:167677038..167683067 |
| TRAPPC9 | Variation_44337 | chr8:140884325..140885345 |
| KCTD17 | Variation_47855 | chr22:37362090..37585844 |
| SNORA63 | Variation_47856 | chr3:186414884..186754730 |
| IFIT1 | Variation_47891 | chr10:91065949..91174175 |
| SLC12A8 | Variation_47990 | chr3:124764414..124868809 |
| TBC1D8 | Variation_47994 | chr2:101624916..102008313 |
| ADK | Variation_48039 | chr10:75938838..76089123 |
| ATRNL1 | Variation_48416 | chr10:117104888..117121966 |
| HTRA1 | Variation_48442 | chr10:124240785..124242817 |
| LHPP | Variation_48444 | chr10:126171612..126173556 |
| LHPP | Variation_48445 | chr10:126189866..126192376 |
| LHPP | Variation_48446 | chr10:126190609..126192376 |
| MGMT | Variation_48465 | chr10:131471722..131474247 |
| SYCE1 | Variation_48476 | chr10:135266389..135369054 |
| SYCE1 | Variation_48477 | chr10:135266389..135369532 |
| SYCE1 | Variation_48478 | chr10:135266389..135370005 |
| SYCE1 | Variation_48479 | chr10:135266389..135377448 |
| SYCE1 | Variation_48480 | chr10:135266389..135377448 |
| SYCE1 | Variation_48482 | chr10:135352013..135434303 |
| SYCE1 | Variation_48483 | chr10:135352100..135434303 |
| SYCE1 | Variation_48484 | chr10:135357239..135434303 |
| MBL2 | Variation_48565 | chr10:54524658..54531235 |
| PCDH15 | Variation_48574 | chr10:56158812..56163201 |
| PCDH15 | Variation_48575 | chr10:56247631..56262826 |
| PCDH15 | Variation_48576 | chr10:56462471..56467563 |
| LRRTM3 | Variation_48609 | chr10:68741835..68922242 |
| STOX1 | Variation_48613 | chr10:70616199..70618859 |
| KIAA1279 | Variation_48614 | chr10:70691708..70775081 |
| KIAA1274 | Variation_48620 | chr10:72311394..72315214 |
| ADAMTS14 | Variation_48621 | chr10:72514611..72516138 |
| ADAMTS14 | Variation_48623 | chr10:72515691..72517206 |
| KIAA0913 | Variation_48629 | chr10:75549916..75648249 |
| ADK | Variation_48630 | chr10:76007903..76373904 |
| ADK | Variation_48631 | chr10:76372037..76373904 |
| SAMD8 | Variation_48633 | chr10:76873606..76891096 |
| NRG3 | Variation_48643 | chr10:83884894..83888343 |
| NRG3 | Variation_48644 | chr10:83993404..84052222 |
| NRG3 | Variation_48645 | chr10:84123925..84137873 |
| NRG3 | Variation_48646 | chr10:84401508..84429842 |
| NRG3 | Variation_48647 | chr10:84401508..84430088 |
| NRG3 | Variation_48648 | chr10:84420273..84430088 |
| NRG3 | Variation_48649 | chr10:84420273..84433391 |
| NRG3 | Variation_48650 | chr10:84537367..84565851 |
| NRG3 | Variation_48651 | chr10:84713118..84715289 |
| NRG3 | Variation_48652 | chr10:84713118..84716523 |
| EXOC6 | Variation_48673 | chr10:94660409..94725508 |
| OR5AS1 | Variation_48846 | chr11:55685556..55819544 |
| OR5AS1 | Variation_48847 | chr11:55685556..55836620 |
| GLYATL2 | Variation_48858 | chr11:58553771..58632368 |
| KSR2 | Variation_48956 | chr12:118146899..118150989 |
| VPS37B | Variation_48968 | chr12:123335527..123364198 |
| VPS37B | Variation_48969 | chr12:123335527..123387922 |
| VPS37B | Variation_48970 | chr12:123335527..123394850 |
| VPS37B | Variation_48971 | chr12:123335527..123396561 |
| PDZRN4 | Variation_49079 | chr12:41653979..41662453 |
| COPZ1 | Variation_49097 | chr12:54740379..54748834 |
| PIBF1 | Variation_49252 | chr13:73559982..73598865 |
| PIBF1 | Variation_49253 | chr13:73559982..73603693 |
| UBR7 | Variation_49446 | chr14:93695018..93699003 |
| AVEN | Variation_49527 | chr15:34276169..34277907 |
| UBR1 | Variation_49556 | chr15:43319431..43336344 |
| PSTPIP1 | Variation_49591 | chr15:77302969..77327571 |
| PSTPIP1 | Variation_49592 | chr15:77302969..77381222 |
| EFTUD1 | Variation_49601 | chr15:82537354..82540174 |
| FES | Variation_49620 | chr15:91411656..91525197 |
| GGNBP2 | Variation_49849 | chr17:34815551..34905408 |
| CACNB1 | Variation_49852 | chr17:37341235..37349655 |
| WIPF2 | Variation_49856 | chr17:38388040..38405037 |
| KIAA1267 | Variation_49864 | chr17:44118269..44350090 |
| KIAA1267 | Variation_49865 | chr17:44131305..44350090 |
| KIAA1267 | Variation_49866 | chr17:44140748..44250108 |
| KIAA1267 | Variation_49867 | chr17:44169808..44250108 |
| KIAA1267 | Variation_49868 | chr17:44169808..44293020 |
| KIAA1267 | Variation_49869 | chr17:44169808..44350090 |
| KIAA1267 | Variation_49873 | chr17:44189067..44350090 |
| KIAA1267 | Variation_49874 | chr17:44204373..44350090 |
| KIAA1267 | Variation_49875 | chr17:44247017..44350090 |
| SLC39A11 | Variation_49922 | chr17:70734687..70745592 |
| SLC39A11 | Variation_49923 | chr17:71053764..71055866 |
| GRIN2C | Variation_49925 | chr17:72837012..72851133 |
| TBC1D16 | Variation_49942 | chr17:77933071..77940637 |
| TBC1D16 | Variation_49943 | chr17:77982846..77997689 |
| TEX101 | Variation_50177 | chr19:43885085..43960114 |
| DHX34 | Variation_50184 | chr19:47877210..47881508 |
| NCKAP5 | Variation_50292 | chr2:133999831..134054662 |
| NCKAP5 | Variation_50293 | chr2:134004535..134054662 |
| NCKAP5 | Variation_50294 | chr2:134301130..134391214 |
| NOSTRIN | Variation_50330 | chr2:169714893..169721377 |
| PPIG | Variation_50331 | chr2:170486172..170488770 |
| BOLL | Variation_50368 | chr2:198600730..198605514 |
| PLEKHM3 | Variation_50385 | chr2:208716745..208717783 |
| CD8B | Variation_50581 | chr2:87084916..87108211 |
| IL17RA | Variation_50797 | chr22:17585441..17589246 |
| C22orf25 | Variation_50812 | chr22:20029878..20059763 |
| LZTR1 | Variation_50815 | chr22:21331556..21346719 |
| LZTR1 | Variation_50816 | chr22:21331556..21350880 |
| KREMEN1 | Variation_50847 | chr22:29477841..29482221 |
| SFI1 | Variation_50849 | chr22:31977594..32024980 |
| SFI1 | Variation_50850 | chr22:31998612..32024980 |
| SFI1 | Variation_50851 | chr22:32011225..32024980 |
| CYB5R3 | Variation_50870 | chr22:43023980..43027824 |
| SCUBE1 | Variation_50871 | chr22:43624765..43627646 |
| TFG | Variation_50892 | chr3:100324442..100433592 |
| TFG | Variation_50893 | chr3:100350745..100432975 |
| TFG | Variation_50894 | chr3:100350745..100433592 |
| DIRC2 | Variation_50917 | chr3:122595777..122610629 |
| CCDC50 | Variation_51045 | chr3:191065392..191067244 |
| CCDC50 | Variation_51046 | chr3:191065392..191068288 |
| CCDC50 | Variation_51047 | chr3:191065392..191069984 |
| CCDC50 | Variation_51048 | chr3:191065392..191073228 |
| CCDC50 | Variation_51049 | chr3:191065392..191073228 |
| CCDC50 | Variation_51051 | chr3:191065921..191067244 |
| CCDC50 | Variation_51052 | chr3:191065921..191068288 |
| CCDC50 | Variation_51053 | chr3:191065921..191069984 |
| CCDC50 | Variation_51054 | chr3:191065921..191073228 |
| CCDC50 | Variation_51056 | chr3:191066878..191068288 |
| CCDC50 | Variation_51057 | chr3:191066878..191069984 |
| CCDC50 | Variation_51058 | chr3:191067244..191069984 |
| CCDC50 | Variation_51059 | chr3:191068288..191073228 |
| MUC4 | Variation_51069 | chr3:195477791..195505664 |
| SLIT2 | Variation_51359 | chr4:20552510..20565861 |
| SLIT2 | Variation_51360 | chr4:20557459..20563108 |
| SLIT2 | Variation_51361 | chr4:20557459..20565861 |
| MAPK10 | Variation_51473 | chr4:86975062..86979927 |
| MAPK10 | Variation_51475 | chr4:86979082..86990027 |
| MAPK10 | Variation_51477 | chr4:87142248..87165758 |
| HSD17B13 | Variation_51479 | chr4:88186509..88228228 |
| ERGIC1 | Variation_51618 | chr5:172324978..172350638 |
| ERGIC1 | Variation_51619 | chr5:172329734..172341917 |
| ERGIC1 | Variation_51620 | chr5:172336616..172341917 |
| PARK2 | Variation_51837 | chr6:162092501..162095580 |
| PARK2 | Variation_51838 | chr6:162499177..162506568 |
| PARK2 | Variation_51839 | chr6:162584753..162586066 |
| PARK2 | Variation_51840 | chr6:162584753..162592807 |
| PARK2 | Variation_51841 | chr6:162738705..162739765 |
| PARK2 | Variation_51842 | chr6:162795058..162841979 |
| PARK2 | Variation_51843 | chr6:162964645..162966431 |
| BAI3 | Variation_51955 | chr6:69619007..69638504 |
| BAI3 | Variation_51956 | chr6:69688165..69690567 |
| BAI3 | Variation_51958 | chr6:70007450..70018250 |
| BAI3 | Variation_51959 | chr6:70014608..70018250 |
| SEMA3A | Variation_52299 | chr7:83782435..83786460 |
| PDGFRL | Variation_52459 | chr8:17484526..17491730 |
| ADK | Variation_53073 | chr10:76247105..76373904 |
| SYCE1 | Variation_53096 | chr10:135303032..135402200 |
| PARK2 | Variation_53108 | chr6:162716541..162943061 |
| TFG | Variation_53110 | chr3:100310812..100433592 |
| PCDH15 | Variation_53155 | chr10:56084933..56132046 |
| TANC1 | Variation_53156 | chr2:159651734..159953466 |
| TRAPPC9 | Variation_53174 | chr8:140758956..140805932 |
| PCDH15 | Variation_53200 | chr10:56236731..56273552 |
| PDE10A | Variation_53203 | chr6:165910706..165946772 |
| PARK2 | Variation_53223 | chr6:162914986..162976039 |
| C6orf132 | Variation_53255 | chr6:42052751..42099371 |
| AVEN | Variation_53263 | chr15:34276169..34277907 |
| TRAPPC9 | Variation_53328 | chr8:140777251..140822965 |
| DLG1 | Variation_53339 | chr3:196910082..197344176 |
| PCDH15 | Variation_53355 | chr10:56245351..56262826 |
| PCDH15 | Variation_53360 | chr10:56550427..56599297 |
| WFS1 | Variation_53398 | chr4:6256489..6303354 |
| FAM149A | Variation_53588 | chr4:186929226..187131504 |
| SYCE1 | Variation_53611 | chr10:135352013..135434303 |
| KIAA1267 | Variation_53692 | chr17:44109843..44293020 |
| KIAA1267 | Variation_53710 | chr17:44169808..44253623 |
| IGSF11 | Variation_53713 | chr3:118729114..118809508 |
| CCDC50 | Variation_53752 | chr3:191065392..191067244 |
| PARK2 | Variation_53797 | chr6:162691418..163044126 |
| SYCE1 | Variation_53803 | chr10:135266389..135434303 |
| CCDC50 | Variation_53828 | chr3:191059322..191073228 |
| TFG | Variation_53947 | chr3:100350745..100433592 |
| CRYBB2 | Variation_53978 | chr22:25623323..25910667 |
| SYCE1 | Variation_53981 | chr10:135258147..135377448 |
| CCDC50 | Variation_53996 | chr3:191065392..191069984 |
| KIAA1267 | Variation_54001 | chr17:44163925..44293020 |
| CRYBB2 | Variation_54003 | chr22:25623323..25958952 |
| TRIM58 | Variation_58378 | chr1:248028742..248034700 |
| ATRNL1 | Variation_58419 | chr10:117522569..117533843 |
| PTPRE | Variation_58425 | chr10:129882250..129883482 |
| TCERG1L | Variation_58427 | chr10:132909088..132912844 |
| SYCE1 | Variation_58429 | chr10:135352611..135367508 |
| PCDH15 | Variation_58452 | chr10:56445921..56469970 |
| VPS26A | Variation_58463 | chr10:70926512..70928289 |
| ADAMTS14 | Variation_58465 | chr10:72445898..72451156 |
| ADAMTS14 | Variation_58466 | chr10:72512957..72514661 |
| C10orf11 | Variation_58468 | chr10:78252165..78262390 |
| IFIT5 | Variation_58479 | chr10:91165763..91174600 |
| FOXR1 | Variation_58497 | chr11:118829029..118849507 |
| PATL1 | Variation_58530 | chr11:59431720..59434566 |
| EIF1AD | Variation_58534 | chr11:65757756..65764902 |
| PC | Variation_58535 | chr11:66709552..66715025 |
| IFT81 | Variation_58552 | chr12:110573257..110580101 |
| TRIAP1 | Variation_58553 | chr12:120880098..120882476 |
| LCP1 | Variation_58629 | chr13:46752468..46753680 |
| KLF5 | Variation_58641 | chr13:73631867..73636098 |
| SLC12A6 | Variation_58714 | chr15:34585431..34597560 |
| PTPN9 | Variation_58736 | chr15:75819453..75865881 |
| SLCO3A1 | Variation_58742 | chr15:92674111..92677451 |
| CES1 | Variation_58763 | chr16:55827454..55866012 |
| MEOX1 | Variation_58793 | chr17:41739241..41743178 |
| GPATCH8 | Variation_58794 | chr17:42468083..42472651 |
| MSI2 | Variation_58806 | chr17:55686686..55690121 |
| ST6GALNAC1 | Variation_58816 | chr17:74623744..74625209 |
| CYTH1 | Variation_58818 | chr17:76677041..76688069 |
| UBXN4 | Variation_58920 | chr2:136509495..136519435 |
| TANC1 | Variation_58928 | chr2:159959393..159961440 |
| TMEM131 | Variation_59016 | chr2:98418819..98421568 |
| MORC1 | Variation_59084 | chr3:108836760..108838876 |
| SLC12A8 | Variation_59088 | chr3:124803931..124811031 |
| CCDC50 | Variation_59119 | chr3:191064652..191071679 |
| MUC4 | Variation_59127 | chr3:195473594..195478084 |
| TFRC | Variation_59128 | chr3:195770699..195776215 |
| DLG1 | Variation_59130 | chr3:196934414..196938381 |
| FAM149A | Variation_59197 | chr4:187093348..187098235 |
| MAPK10 | Variation_59215 | chr4:87016603..87017642 |
| PARK2 | Variation_59310 | chr6:162738018..162740552 |
| FOXP4 | Variation_59341 | chr6:41566886..41568116 |
| CD2AP | Variation_59344 | chr6:47512446..47522766 |
| PCLO | Variation_59424 | chr7:82783682..82785515 |
| TM2D2 | Variation_59472 | chr8:38848740..38853026 |
| C8orf37 | Variation_59499 | chr8:96259515..96260863 |
| TRAPPC2L | Variation_59564 | chr16:88872229..88927221 |
| TRAPPC2L | Variation_59565 | chr16:88872229..88927221 |
| METRNL | Variation_59566 | chr17:81016049..81060000 |
| FANK1 | Variation_59685 | chr10:127592805..127594438 |
| FANK1 | Variation_59686 | chr10:127594378..127608372 |
| AVEN | Variation_59727 | chr15:34184762..34245543 |
| TIAM2 | Variation_59838 | chr6:155481845..155482620 |
| CES1 | Variation_59912 | chr16:55814091..55850411 |
| C10orf11 | Variation_61037 | chr10:78255376..78261147 |
| NRG3 | Variation_61043 | chr10:84237703..84238722 |
| TCERG1L | Variation_61078 | chr10:132908808..132913036 |
| PIAS1 | Variation_61456 | chr15:68425779..68429144 |
| CDC27 | Variation_61570 | chr17:45234367..45235619 |
| NCKAP5 | Variation_61827 | chr2:133647038..133648263 |
| TMEM163 | Variation_61832 | chr2:135260560..135262356 |
| GRXCR1 | Variation_62182 | chr4:42904974..42906073 |
| SPOCK3 | Variation_62283 | chr4:167676781..167683351 |
| TRAPPC9 | Variation_62864 | chr8:141040289..141043063 |
| SETD8 | Variation_63048 | chr12:123875230..123879670 |
| CDC27 | Variation_63056 | chr17:45219757..45221297 |
| CDC27 | Variation_63057 | chr17:45249307..45266575 |
| CDC27 | Variation_63058 | chr17:45247369..45249359 |
| NCKAP5 | Variation_63199 | chr2:133672188..133674783 |
| ZRANB3 | Variation_63201 | chr2:136099417..136101666 |
| TANC1 | Variation_63222 | chr2:159959794..159960963 |
| ATG7 | Variation_63393 | chr3:11410253..11414707 |
| LARS2 | Variation_63458 | chr3:45543287..45552064 |
| ABI3BP | Variation_63537 | chr3:100669310..100670862 |
| IGSF11 | Variation_63565 | chr3:118855337..119000251 |
| GSK3B | Variation_63567 | chr3:119655507..119656904 |
| TMEM108 | Variation_63595 | chr3:133016100..133025982 |
| EPHB1 | Variation_63596 | chr3:134842590..134844395 |
| CLSTN2 | Variation_63601 | chr3:139699689..139701329 |
| CLSTN2 | Variation_63603 | chr3:140267503..140270906 |
| CCDC50 | Variation_63689 | chr3:191064731..191071632 |
| LMLN | Variation_63718 | chr3:197737815..197742731 |
| DOK7 | Variation_63726 | chr4:3468004..3474478 |
| SLIT2 | Variation_63769 | chr4:20407568..20408857 |
| MTHFD2L | Variation_63863 | chr4:75078561..75083647 |
| MAPK10 | Variation_63882 | chr4:86976391..86980012 |
| MAPK10 | Variation_63883 | chr4:87148074..87152319 |
| SPOCK3 | Variation_64016 | chr4:167875942..167892836 |
| SPOCK3 | Variation_64017 | chr4:168024645..168080542 |
| SPOCK3 | Variation_64018 | chr4:168109174..168115505 |
| FAM149A | Variation_64060 | chr4:187093513..187111440 |
| TGFBR3 | Variation_64233 | chr1:92232083..92233422 |
| CD2AP | Variation_64498 | chr6:47564740..47566899 |
| BAI3 | Variation_64553 | chr6:69687686..69691720 |
| BAI3 | Variation_64554 | chr6:70014088..70018507 |
| PARK2 | Variation_64701 | chr6:161811876..161814807 |
| PARK2 | Variation_64703 | chr6:162384846..162386527 |
| PARK2 | Variation_64704 | chr6:162496365..162506976 |
| PARK2 | Variation_64705 | chr6:162578556..162580303 |
| PARK2 | Variation_64706 | chr6:162583740..162587157 |
| PDE10A | Variation_64711 | chr6:165957499..165958831 |
| THBS2 | Variation_64725 | chr6:169633222..169635277 |
| CNGB3 | Variation_65187 | chr8:87648215..87649641 |
| CNGB3 | Variation_65189 | chr8:87671249..87672604 |
| MMP16 | Variation_65192 | chr8:89098166..89100420 |
| KIAA1429 | Variation_65197 | chr8:95558324..95561351 |
| TRAPPC9 | Variation_65285 | chr8:141204925..141207002 |
| PTK2 | Variation_65286 | chr8:142002613..142005570 |
| MAMDC2 | Variation_65419 | chr9:72762238..72763468 |
| MBL2 | Variation_65680 | chr10:54526625..54529250 |
| PCDH15 | Variation_65684 | chr10:55939729..56047721 |
| PCDH15 | Variation_65685 | chr10:56446076..56469571 |
| ZNF365 | Variation_65702 | chr10:64425664..64430909 |
| STOX1 | Variation_65714 | chr10:70612585..70622298 |
| ADAMTS14 | Variation_65720 | chr10:72448818..72451301 |
| C10orf11 | Variation_65726 | chr10:78255613..78261009 |
| NRG3 | Variation_65735 | chr10:83883604..83889070 |
| NRG3 | Variation_65736 | chr10:84127870..84130301 |
| NRG3 | Variation_65737 | chr10:84712311..84717625 |
| NEURL | Variation_65776 | chr10:105308399..105311973 |
| LHPP | Variation_65807 | chr10:126188285..126192415 |
| MGMT | Variation_65815 | chr10:131562778..131576387 |
| TCERG1L | Variation_65818 | chr10:132908725..132913146 |
| KNDC1 | Variation_65824 | chr10:135035898..135037408 |
| SYCE1 | Variation_65825 | chr10:135339868..135515044 |
| PRDX5 | Variation_65971 | chr11:64085418..64087410 |
| PC | Variation_65976 | chr11:66712149..66713260 |
| TPCN2 | Variation_65985 | chr11:68843734..68844904 |
| CBX5 | Variation_66229 | chr12:54673465..54674611 |
| C12orf34 | Variation_66325 | chr12:110151456..110152801 |
| MED13L | Variation_66339 | chr12:116526479..116528480 |
| PCDH8 | Variation_66444 | chr13:53419990..53422747 |
| KLF5 | Variation_66480 | chr13:73632682..73634039 |
| SCEL | Variation_66487 | chr13:78159769..78161394 |
| STXBP6 | Variation_66600 | chr14:25497781..25499192 |
| HSP90AA1 | Variation_66711 | chr14:102573178..102574378 |
| STRC | Variation_66780 | chr15:43852144..43988641 |
| STRC | Variation_66782 | chr15:43852144..43988641 |
| SLCO3A1 | Variation_66874 | chr15:92577248..92579208 |
| SLCO3A1 | Variation_66875 | chr15:92588543..92606763 |
| SLCO3A1 | Variation_66876 | chr15:92674890..92676996 |
| PCSK6 | Variation_66907 | chr15:102028912..102030796 |
| CES1 | Variation_67001 | chr16:55842125..55865379 |
| ZDHHC7 | Variation_67069 | chr16:85040399..85043171 |
| KLHDC4 | Variation_67077 | chr16:87759623..87760962 |
| SPIRE2 | Variation_67088 | chr16:89896098..89898402 |
| STAT5B | Variation_67200 | chr17:40427345..40428983 |
| ARHGAP27 | Variation_67208 | chr17:43496222..43497302 |
| KIAA1267 | Variation_67211 | chr17:44271126..44368872 |
| KIAA1267 | Variation_67212 | chr17:44212815..44270230 |
| SKAP1 | Variation_67214 | chr17:46400513..46402257 |
| CALCOCO2 | Variation_67216 | chr17:46904886..46908430 |
| MSI2 | Variation_67231 | chr17:55687846..55689837 |
| APPBP2 | Variation_67237 | chr17:58579742..58581293 |
| BCAS3 | Variation_67239 | chr17:59118927..59128738 |
| CACNG4 | Variation_67246 | chr17:64959657..64961650 |
| CACNG4 | Variation_67247 | chr17:64984874..64986080 |
| PITPNC1 | Variation_67248 | chr17:65388196..65401678 |
| PITPNC1 | Variation_67249 | chr17:65438392..65443542 |
| SLC39A11 | Variation_67255 | chr17:70789985..70795175 |
| SLC39A11 | Variation_67256 | chr17:70815169..70821126 |
| PRPSAP1 | Variation_67264 | chr17:74335187..74336674 |
| TBC1D16 | Variation_67278 | chr17:77992048..78000962 |
| METRNL | Variation_67284 | chr17:81045738..81060000 |
| SETBP1 | Variation_67348 | chr18:42624662..42626047 |
| TYK2 | Variation_67465 | chr19:10489139..10505017 |
| BCL2L13 | Variation_67829 | chr22:18126982..18129450 |
| BCL2L13 | Variation_67830 | chr22:18141502..18144456 |
| CRYBB2 | Variation_67861 | chr22:25619034..25928990 |
| ZNRF3 | Variation_67862 | chr22:29383848..29386970 |
| EMID1 | Variation_67863 | chr22:29633118..29635222 |
| EWSR1 | Variation_67864 | chr22:29680283..29681805 |
| TMEM131 | Variation_68092 | chr2:98544799..98547910 |
| REV1 | Variation_68094 | chr2:100103724..100105212 |
| NCK2 | Variation_68103 | chr2:106438847..106440674 |
| NCK2 | Variation_68104 | chr2:106508989..106546685 |
| TMEM163 | Variation_68182 | chr2:135364265..135367580 |
| ZXDC | Variation_68449 | chr3:126194061..126196104 |
| UROC1 | Variation_68451 | chr3:126234394..126235747 |
| EPHB1 | Variation_68472 | chr3:134647088..134648608 |
| CLSTN2 | Variation_68474 | chr3:139923606..139924666 |
| TSC22D2 | Variation_68482 | chr3:150124574..150128485 |
| VPS8 | Variation_68519 | chr3:184766850..184768075 |
| VPS8 | Variation_68520 | chr3:184766850..184786757 |
| RTP1 | Variation_68523 | chr3:186906378..186968680 |
| MUC4 | Variation_68547 | chr3:195471313..195477817 |
| MUC4 | Variation_68550 | chr3:195501958..195515607 |
| MUC4 | Variation_68551 | chr3:195501958..195504963 |
| MFI2 | Variation_68560 | chr3:196729023..196730780 |
| DLG1 | Variation_68562 | chr3:196934570..196939351 |
| LMLN | Variation_68572 | chr3:197730419..197732220 |
| NAT8L | Variation_68620 | chr4:2060387..2061782 |
| DOK7 | Variation_68629 | chr4:3492020..3493441 |
| EVC2 | Variation_68640 | chr4:5614696..5620033 |
| REST | Variation_68729 | chr4:57772270..57775584 |
| ACSL1 | Variation_68866 | chr4:185746453..185747593 |
| PARK2 | Variation_69644 | chr6:162150845..162152544 |
| DNAH11 | Variation_69821 | chr7:21763945..21769647 |
| EPHX2 | Variation_70327 | chr8:27381785..27383010 |
| FAM135B | Variation_70445 | chr8:139173685..139175125 |
| TRAPPC9 | Variation_70449 | chr8:140759587..140761010 |
| TRAPPC9 | Variation_70450 | chr8:140759282..140770035 |
| TRAPPC9 | Variation_70451 | chr8:140767060..140769620 |
| SYK | Variation_70766 | chr9:93603341..93605009 |
| EXD3 | Variation_70842 | chr9:140223009..140224284 |
| EXD3 | Variation_70843 | chr9:140221018..140224869 |
| EXD3 | Variation_70845 | chr9:140223009..140225119 |
| EXD3 | Variation_70846 | chr9:140253772..140255871 |
| NELF | Variation_70849 | chr9:140344836..140346737 |
| BMS1 | Variation_70974 | chr10:43301539..43355457 |
| BMS1 | Variation_70975 | chr10:43325598..43331203 |
| PCDH15 | Variation_71016 | chr10:56469623..56503557 |
| STOX1 | Variation_71035 | chr10:70586985..70588121 |
| CDH23 | Variation_71038 | chr10:73156217..73158587 |
| ZMIZ1 | Variation_71052 | chr10:81002132..81003538 |
| NRG3 | Variation_71065 | chr10:83634119..83635215 |
| LHPP | Variation_71128 | chr10:126195215..126196530 |
| LHPP | Variation_71129 | chr10:126188375..126196450 |
| FANK1 | Variation_71131 | chr10:127577329..127591234 |
| DOCK1 | Variation_71135 | chr10:129145693..129146869 |
| FOXI2 | Variation_71138 | chr10:129488612..129553456 |
| PTPRE | Variation_71139 | chr10:129830202..129831283 |
| MGMT | Variation_71141 | chr10:131303674..131308469 |
| TCERG1L | Variation_71153 | chr10:132988416..132991199 |
| TCERG1L | Variation_71154 | chr10:133026399..133027469 |
| INPP5A | Variation_71178 | chr10:134365671..134367846 |
| INPP5A | Variation_71179 | chr10:134424215..134425935 |
| INPP5A | Variation_71181 | chr10:134584997..134586392 |
| GPR123 | Variation_71189 | chr10:134924440..134925685 |
| KNDC1 | Variation_71193 | chr10:134987972..134989752 |
| SYCE1 | Variation_71198 | chr10:135379416..135381177 |
| SYCE1 | Variation_71203 | chr10:135350470..135477925 |
| OR51M1 | Variation_71261 | chr11:5409794..5412609 |
| NRXN2 | Variation_71341 | chr11:64407674..64409875 |
| TPCN2 | Variation_71345 | chr11:68835696..68837291 |
| SHANK2 | Variation_71348 | chr11:70830303..70849553 |
| ARHGAP20 | Variation_71377 | chr11:110488938..110490511 |
| ARHGAP20 | Variation_71378 | chr11:110488938..110492858 |
| PDZRN4 | Variation_71497 | chr12:41685767..41687157 |
| ESYT1 | Variation_71517 | chr12:56524824..56527928 |
| CCDC64 | Variation_71597 | chr12:120426639..120427988 |
| TDRD3 | Variation_71726 | chr13:61068566..61088065 |
| LMO7 | Variation_71741 | chr13:76209699..76211118 |
| ZNF692 | Variation_71816 | chr1:249144921..249150547 |
| TMEM55B | Variation_71865 | chr14:20800765..21257878 |
| PPP1R13B | Variation_71974 | chr14:104312355..104315015 |
| KIAA0284 | Variation_71986 | chr14:105331121..105332456 |
| AVEN | Variation_72231 | chr15:34200448..34201748 |
| EDC3 | Variation_72286 | chr15:74911304..74925497 |
| KIAA1024 | Variation_72297 | chr15:79728493..79731981 |
| MESDC1 | Variation_72302 | chr15:81292494..81295779 |
| EFTUD1 | Variation_72303 | chr15:82483011..82486833 |
| AP3B2 | Variation_72331 | chr15:83327137..83332372 |
| HOMER2 | Variation_72332 | chr15:83602318..83604788 |
| EXOSC6 | Variation_72607 | chr16:70145134..70285081 |
| EXOSC6 | Variation_72608 | chr16:70234205..70284981 |
| DBNDD1 | Variation_72708 | chr16:90074225..90075225 |
| UBTF | Variation_72941 | chr17:42295583..42299041 |
| CDC27 | Variation_72967 | chr17:45212900..45214698 |
| NPEPPS | Variation_72968 | chr17:45616210..45620061 |
| NPEPPS | Variation_72969 | chr17:45616210..45671173 |
| NPEPPS | Variation_72970 | chr17:45620569..45633308 |
| SLC39A11 | Variation_73013 | chr17:70910723..70911793 |
| RNF157 | Variation_73018 | chr17:74235793..74237089 |
| METRNL | Variation_73080 | chr17:81012068..81060000 |
| ZNF516 | Variation_73173 | chr18:74104897..74106762 |
| PQLC1 | Variation_73235 | chr18:77679469..77682189 |
| CECR5 | Variation_73694 | chr22:17627214..17628539 |
| HIC2 | Variation_73764 | chr22:21772188..21797403 |
| HIC2 | Variation_73765 | chr22:21653692..21797733 |
| SPECC1L | Variation_73797 | chr22:24670499..24675012 |
| SPECC1L | Variation_73800 | chr22:24633691..24675073 |
| MTMR3 | Variation_73810 | chr22:30280400..30298271 |
| EIF3D | Variation_73820 | chr22:36918738..36923490 |
| TTLL1 | Variation_73831 | chr22:43431422..43436218 |
| SCUBE1 | Variation_73833 | chr22:43676579..43677609 |
| PARVB | Variation_73835 | chr22:44508554..44512124 |
| KIAA1644 | Variation_73836 | chr22:44698495..44699540 |
| RFX8 | Variation_74184 | chr2:102052665..102055743 |
| TGFBR3 | Variation_74567 | chr1:92232092..92233298 |
| C10orf11 | Variation_75315 | chr10:78254338..78261050 |
| C10orf11 | Variation_75316 | chr10:78253362..78260571 |
| C10orf11 | Variation_75317 | chr10:78260679..78264431 |
| NRG3 | Variation_75330 | chr10:84127858..84130238 |
| NRG3 | Variation_75331 | chr10:84126906..84130238 |
| CNNM2 | Variation_75372 | chr10:104748742..104750506 |
| FANK1 | Variation_75411 | chr10:127578531..127599293 |
| FANK1 | Variation_75412 | chr10:127594198..127615074 |
| SYCE1 | Variation_75465 | chr10:135343170..135431688 |
| SYCE1 | Variation_75467 | chr10:135249289..135391722 |
| SYCE1 | Variation_75473 | chr10:135371730..135431688 |
| NUP98 | Variation_75519 | chr11:3716814..3750378 |
| UCHL3 | Variation_76419 | chr13:76107011..76130414 |
| NOVA1 | Variation_76603 | chr14:27066060..27067884 |
| NOVA1 | Variation_76604 | chr14:27065601..27067884 |
| NOVA1 | Variation_76605 | chr14:27064464..27070050 |
| NOVA1 | Variation_76606 | chr14:27065601..27068682 |
| NOVA1 | Variation_76607 | chr14:27066060..27067314 |
| HSP90AA1 | Variation_76736 | chr14:102561056..102564388 |
| TTBK2 | Variation_76957 | chr15:43163485..43175049 |
| PSTPIP1 | Variation_77012 | chr15:77328237..77347445 |
| C15orf58 | Variation_77066 | chr15:90623760..90786590 |
| C15orf58 | Variation_77067 | chr15:90723651..90786590 |
| KLHDC4 | Variation_77453 | chr16:87748532..87750226 |
| PPP1R1B | Variation_77591 | chr17:37761921..37788355 |
| KIAA1267 | Variation_77623 | chr17:44300845..44309175 |
| KIAA1267 | Variation_77624 | chr17:44300845..44301865 |
| KIAA1267 | Variation_77625 | chr17:44301695..44303225 |
| KIAA1267 | Variation_77626 | chr17:44302035..44303055 |
| NPEPPS | Variation_77646 | chr17:45635209..45677158 |
| NPEPPS | Variation_77647 | chr17:45603639..45677158 |
| NPEPPS | Variation_77648 | chr17:45619602..45671025 |
| NPEPPS | Variation_77649 | chr17:45619602..45638923 |
| NPEPPS | Variation_77650 | chr17:45616454..45632505 |
| CBX1 | Variation_77651 | chr17:46147551..46148629 |
| MSI2 | Variation_77675 | chr17:55687900..55689762 |
| APPBP2 | Variation_77691 | chr17:58579901..58581224 |
| TBC1D16 | Variation_77750 | chr17:77993569..77995405 |
| TBC1D16 | Variation_77751 | chr17:77995275..77998924 |
| TBC1D16 | Variation_77752 | chr17:77997853..77998924 |
| ZNF516 | Variation_77937 | chr18:74108481..74116521 |
| ZNF516 | Variation_77938 | chr18:74083691..74116521 |
| ZNF516 | Variation_77939 | chr18:74101111..74107811 |
| ZNF516 | Variation_77940 | chr18:74101781..74116521 |
| ZNF516 | Variation_77941 | chr18:74103791..74107141 |
| ZNF516 | Variation_77942 | chr18:74108481..74113163 |
| AKT2 | Variation_78187 | chr19:40763584..40767589 |
| AKT2 | Variation_78188 | chr19:40756799..40760113 |
| AKT2 | Variation_78189 | chr19:40756799..40769191 |
| AKT2 | Variation_78190 | chr19:40761181..40762516 |
| AKT2 | Variation_78191 | chr19:40763317..40766120 |
| TSGA10 | Variation_78665 | chr2:99711813..99737706 |
| TSGA10 | Variation_78666 | chr2:99713733..99733870 |
| TSGA10 | Variation_78667 | chr2:99718526..99731952 |
| REV1 | Variation_78668 | chr2:100103361..100105152 |
| NCK2 | Variation_78678 | chr2:106428544..106435110 |
| NCK2 | Variation_78679 | chr2:106438883..106440647 |
| RAB3GAP1 | Variation_78805 | chr2:135839984..135843360 |
| ZRANB3 | Variation_78806 | chr2:136100022..136101394 |
| TANC1 | Variation_78836 | chr2:159959748..159961042 |
| TANC1 | Variation_78837 | chr2:159959137..159961042 |
| TANC1 | Variation_78838 | chr2:159959137..159961947 |
| TANC1 | Variation_78839 | chr2:159960648..159961947 |
| UBR3 | Variation_78851 | chr2:170707601..170726215 |
| COMMD7 | Variation_79175 | chr20:31312856..31316137 |
| COMMD7 | Variation_79176 | chr20:31311505..31316137 |
| CTNNBL1 | Variation_79187 | chr20:36474626..36477174 |
| BCL2L13 | Variation_79424 | chr22:18127960..18128968 |
| HIC2 | Variation_79461 | chr22:21465811..21792788 |
| HIC2 | Variation_79463 | chr22:21705933..21792788 |
| SPECC1L | Variation_79517 | chr22:24632828..24673939 |
| SPECC1L | Variation_79520 | chr22:24662193..24668038 |
| CRYBB2 | Variation_79528 | chr22:25620157..25920450 |
| KREMEN1 | Variation_79538 | chr22:29507936..29530917 |
| KREMEN1 | Variation_79539 | chr22:29517316..29522006 |
| KREMEN1 | Variation_79540 | chr22:29507936..29520130 |
| KREMEN1 | Variation_79541 | chr22:29514983..29526227 |
| KREMEN1 | Variation_79542 | chr22:29517316..29520130 |
| KREMEN1 | Variation_79543 | chr22:29517316..29530917 |
| CACNG2 | Variation_79568 | chr22:37027643..37035914 |
| CACNG2 | Variation_79569 | chr22:36989086..37040112 |
| MKL1 | Variation_79581 | chr22:40874590..40876020 |
| SCUBE1 | Variation_79592 | chr22:43670143..43677585 |
| SCUBE1 | Variation_79593 | chr22:43673838..43677353 |
| SCUBE1 | Variation_79594 | chr22:43675073..43679441 |
| SCUBE1 | Variation_79595 | chr22:43675680..43677585 |
| SCUBE1 | Variation_79596 | chr22:43729262..43736759 |
| SCUBE1 | Variation_79597 | chr22:43735688..43736759 |
| EFCAB6 | Variation_79599 | chr22:43974735..43975764 |
| EFCAB6 | Variation_79601 | chr22:44125018..44131486 |
| EFCAB6 | Variation_79602 | chr22:44125018..44126206 |
| EFCAB6 | Variation_79603 | chr22:44184743..44191769 |
| HSPBAP1 | Variation_79870 | chr3:122501628..122511830 |
| TMEM108 | Variation_79912 | chr3:133016241..133023161 |
| PPP2R3A | Variation_79924 | chr3:135697227..135700286 |
| CLSTN2 | Variation_79936 | chr3:140267761..140270355 |
| RAP2B | Variation_79969 | chr3:152878440..152880694 |
| RAP2B | Variation_79970 | chr3:152879544..152880694 |
| MUC4 | Variation_80087 | chr3:195518397..195527999 |
| MUC4 | Variation_80088 | chr3:195522903..195527999 |
| MAPK10 | Variation_80389 | chr4:86976454..86980060 |
| SPOCK3 | Variation_80560 | chr4:167875867..167888853 |
| SPOCK3 | Variation_80561 | chr4:167888853..167890536 |
| SPOCK3 | Variation_80562 | chr4:168022352..168029486 |
| SPOCK3 | Variation_80563 | chr4:168022352..168080613 |
| SPOCK3 | Variation_80564 | chr4:168113253..168115035 |
| SPOCK3 | Variation_80565 | chr4:168110013..168114063 |
| BAI3 | Variation_81436 | chr6:70014070..70018333 |
| PARK2 | Variation_81698 | chr6:162493653..162498161 |
| PARK2 | Variation_81700 | chr6:162498331..162501281 |
| PARK2 | Variation_81701 | chr6:162499087..162500249 |
| PARK2 | Variation_81702 | chr6:162501453..162503861 |
| PARK2 | Variation_81703 | chr6:162578570..162580138 |
| PARK2 | Variation_81704 | chr6:162582833..162586655 |
| PDE10A | Variation_81715 | chr6:165876662..165893371 |
| PDE10A | Variation_81716 | chr6:165885528..165887564 |
| THBS2 | Variation_81771 | chr6:169633491..169636529 |
| PCLO | Variation_82070 | chr7:82462275..82464137 |
| UQCRB | Variation_82698 | chr8:97239539..97242185 |
| TRAPPC9 | Variation_82804 | chr8:141205077..141206743 |
| TRAPPC9 | Variation_82805 | chr8:141205077..141206233 |
| PTK2 | Variation_82807 | chr8:142003018..142004893 |
| TGFBR3 | Variation_84364 | chr1:92232092..92233298 |
| C1orf150 | Variation_84984 | chr1:247712533..247729879 |
| MBL2 | Variation_85293 | chr10:54526745..54529097 |
| PCDH15 | Variation_85300 | chr10:56520310..56530845 |
| STOX1 | Variation_85356 | chr10:70613081..70622951 |
| STOX1 | Variation_85357 | chr10:70621271..70622951 |
| C10orf11 | Variation_85383 | chr10:78253960..78260571 |
| C10orf11 | Variation_85384 | chr10:78249458..78260571 |
| C10orf11 | Variation_85386 | chr10:78255603..78258735 |
| NRG3 | Variation_85398 | chr10:84126906..84130238 |
| NRG3 | Variation_85399 | chr10:84127858..84129626 |
| NRG3 | Variation_85401 | chr10:84711623..84717405 |
| CNNM2 | Variation_85454 | chr10:104748742..104750506 |
| CNNM2 | Variation_85455 | chr10:104752760..104756582 |
| FANK1 | Variation_85514 | chr10:127576467..127588111 |
| FANK1 | Variation_85515 | chr10:127576467..127615074 |
| FANK1 | Variation_85516 | chr10:127583768..127592112 |
| TCERG1L | Variation_85529 | chr10:133099104..133104249 |
| TCERG1L | Variation_85530 | chr10:133101834..133104249 |
| TCERG1L | Variation_85531 | chr10:133100364..133101729 |
| OR5AS1 | Variation_85786 | chr11:55683109..55833490 |
| TWF1 | Variation_86347 | chr12:44180098..44199220 |
| TWF1 | Variation_86348 | chr12:44180098..44265340 |
| MED13L | Variation_86578 | chr12:116690390..116692133 |
| UCHL3 | Variation_86912 | chr13:76103954..76130414 |
| NOVA1 | Variation_87153 | chr14:27065946..27067770 |
| NOVA1 | Variation_87154 | chr14:27064464..27070050 |
| NOVA1 | Variation_87157 | chr14:27066402..27067656 |
| HSP90AA1 | Variation_87364 | chr14:102561056..102564388 |
| SLC12A6 | Variation_87616 | chr15:34588632..34592552 |
| TTBK2 | Variation_87663 | chr15:43163485..43175049 |
| STRC | Variation_87664 | chr15:43833982..44039630 |
| PSTPIP1 | Variation_87753 | chr15:77328237..77347445 |
| PSTPIP1 | Variation_87754 | chr15:77328237..77330981 |
| PSTPIP1 | Variation_87755 | chr15:77328237..77339605 |
| PSTPIP1 | Variation_87756 | chr15:77329413..77332549 |
| SLCO3A1 | Variation_87840 | chr15:92588764..92606306 |
| SLCO3A1 | Variation_87841 | chr15:92588764..92590912 |
| SLCO3A1 | Variation_87842 | chr15:92675004..92676572 |
| ST8SIA2 | Variation_87845 | chr15:92996346..92997816 |
| STARD3 | Variation_88454 | chr17:37801613..37814861 |
| KIAA1267 | Variation_88492 | chr17:44300845..44309175 |
| KIAA1267 | Variation_88493 | chr17:44301865..44303395 |
| KIAA1267 | Variation_88494 | chr17:44300845..44302205 |
| NPEPPS | Variation_88512 | chr17:45616454..45632505 |
| NPEPPS | Variation_88513 | chr17:45629295..45677158 |
| NPEPPS | Variation_88514 | chr17:45613167..45677158 |
| CBX1 | Variation_88517 | chr17:46147551..46148629 |
| MSI2 | Variation_88552 | chr17:55687900..55689762 |
| PPM1E | Variation_88553 | chr17:57003277..57014008 |
| APPBP2 | Variation_88574 | chr17:58579901..58581224 |
| FAM20A | Variation_88590 | chr17:66563262..66565075 |
| ABCA6 | Variation_88591 | chr17:67085700..67094182 |
| SLC39A11 | Variation_88596 | chr17:70790002..70795000 |
| EVPL | Variation_88610 | chr17:74015868..74016995 |
| TBC1D16 | Variation_88642 | chr17:77992804..78000301 |
| TBC1D16 | Variation_88643 | chr17:77997853..77999077 |
| TBC1D16 | Variation_88644 | chr17:77992804..77994640 |
| TBC1D16 | Variation_88645 | chr17:77992804..77996935 |
| ZNF516 | Variation_88930 | chr18:74083691..74116521 |
| ZNF516 | Variation_88931 | chr18:74107141..74113163 |
| ZNF516 | Variation_88932 | chr18:74105131..74116521 |
| ZNF516 | Variation_88933 | chr18:74101781..74107811 |
| AKT2 | Variation_89231 | chr19:40756799..40769191 |
| AKT2 | Variation_89232 | chr19:40763584..40767055 |
| AKT2 | Variation_89233 | chr19:40759846..40762516 |
| AKT2 | Variation_89234 | chr19:40763843..40765720 |
| TMEM131 | Variation_89829 | chr2:98544836..98548045 |
| TSGA10 | Variation_89831 | chr2:99712772..99736750 |
| TSGA10 | Variation_89832 | chr2:99717392..99739624 |
| TSGA10 | Variation_89833 | chr2:99700306..99747308 |
| TSGA10 | Variation_89834 | chr2:99717392..99729075 |
| TSGA10 | Variation_89835 | chr2:99719485..99727128 |
| REV1 | Variation_89836 | chr2:100103330..100105206 |
| REV1 | Variation_89837 | chr2:100103700..100105018 |
| NCK2 | Variation_89856 | chr2:106421782..106428250 |
| NCK2 | Variation_89857 | chr2:106424686..106426534 |
| NCK2 | Variation_89858 | chr2:106428544..106435110 |
| NCK2 | Variation_89859 | chr2:106438883..106440647 |
| RAB3GAP1 | Variation_90033 | chr2:135821433..135822633 |
| RAB3GAP1 | Variation_90034 | chr2:135825720..135839040 |
| RAB3GAP1 | Variation_90035 | chr2:135831120..135843360 |
| RAB3GAP1 | Variation_90036 | chr2:135834000..135839760 |
| RAB3GAP1 | Variation_90037 | chr2:135839040..135843360 |
| RAB3GAP1 | Variation_90038 | chr2:135839984..135841920 |
| ZRANB3 | Variation_90039 | chr2:135972181..135976248 |
| ZRANB3 | Variation_90040 | chr2:136100022..136101394 |
| TANC1 | Variation_90096 | chr2:159959137..159961947 |
| TANC1 | Variation_90097 | chr2:159959703..159961042 |
| TANC1 | Variation_90098 | chr2:159959137..159961413 |
| TANC1 | Variation_90099 | chr2:159959838..159961847 |
| UBR3 | Variation_90125 | chr2:170657417..170690977 |
| UBR3 | Variation_90126 | chr2:170670664..170694342 |
| PLEKHM3 | Variation_90238 | chr2:208790882..208820768 |
| MAP2 | Variation_90245 | chr2:210248429..210297989 |
| MAP2 | Variation_90246 | chr2:210271557..210297989 |
| COMMD7 | Variation_90537 | chr20:31312856..31316137 |
| CTNNBL1 | Variation_90547 | chr20:36474626..36477174 |
| BCL2L13 | Variation_90862 | chr22:18127960..18128968 |
| HIC2 | Variation_90898 | chr22:21465811..21792788 |
| HIC2 | Variation_90900 | chr22:21712653..21792788 |
| SPECC1L | Variation_90985 | chr22:24632828..24673939 |
| CRYBB2 | Variation_90997 | chr22:25620157..25920450 |
| KREMEN1 | Variation_91016 | chr22:29507936..29530917 |
| KREMEN1 | Variation_91017 | chr22:29516378..29522944 |
| KREMEN1 | Variation_91018 | chr22:29512626..29527185 |
| KREMEN1 | Variation_91019 | chr22:29516847..29521068 |
| KREMEN1 | Variation_91020 | chr22:29517316..29520130 |
| EWSR1 | Variation_91021 | chr22:29678701..29681739 |
| CACNG2 | Variation_91052 | chr22:37024493..37040112 |
| MKL1 | Variation_91065 | chr22:40873589..40880596 |
| MKL1 | Variation_91066 | chr22:40877164..40880024 |
| TTLL1 | Variation_91074 | chr22:43430068..43435801 |
| SCUBE1 | Variation_91076 | chr22:43673228..43677585 |
| SCUBE1 | Variation_91077 | chr22:43676156..43677585 |
| SCUBE1 | Variation_91078 | chr22:43676974..43680369 |
| SCUBE1 | Variation_91082 | chr22:43729262..43736759 |
| EFCAB6 | Variation_91085 | chr22:43974735..43975764 |
| EFCAB6 | Variation_91086 | chr22:44071951..44073666 |
| EFCAB6 | Variation_91087 | chr22:44125018..44131486 |
| EFCAB6 | Variation_91088 | chr22:44125414..44129374 |
| ATG7 | Variation_91189 | chr3:11410353..11414812 |
| ATG7 | Variation_91190 | chr3:11412082..11413629 |
| ABI3BP | Variation_91447 | chr3:100669283..100670802 |
| C3orf17 | Variation_91474 | chr3:112728281..112752797 |
| IQCB1 | Variation_91497 | chr3:121493891..121518832 |
| IQCB1 | Variation_91498 | chr3:121501017..121518832 |
| IQCB1 | Variation_91499 | chr3:121498472..121509670 |
| IQCB1 | Variation_91500 | chr3:121501503..121513233 |
| IQCB1 | Variation_91501 | chr3:121508652..121518832 |
| HSPBAP1 | Variation_91503 | chr3:122438244..122519976 |
| HSPBAP1 | Variation_91504 | chr3:122461596..122479955 |
| HSPBAP1 | Variation_91505 | chr3:122476608..122489952 |
| TMEM108 | Variation_91565 | chr3:132808677..132821172 |
| TMEM108 | Variation_91566 | chr3:133016241..133024718 |
| EPHB1 | Variation_91576 | chr3:134842471..134844235 |
| PPP2R3A | Variation_91580 | chr3:135697227..135701091 |
| PPP2R3A | Variation_91581 | chr3:135697227..135705116 |
| CLSTN2 | Variation_91600 | chr3:140267653..140270355 |
| RAP2B | Variation_91644 | chr3:152878440..152880694 |
| CCDC50 | Variation_91779 | chr3:191064701..191071692 |
| CCDC50 | Variation_91780 | chr3:191064701..191082658 |
| CCDC50 | Variation_91781 | chr3:191064701..191068743 |
| CCDC50 | Variation_91782 | chr3:191064701..191073142 |
| MUC4 | Variation_91823 | chr3:195518397..195527999 |
| SLIT2 | Variation_91964 | chr4:20556742..20565826 |
| MTHFD2L | Variation_92202 | chr4:75081452..75083461 |
| MAPK10 | Variation_92235 | chr4:86976454..86980060 |
| SPOCK3 | Variation_92527 | chr4:167881204..167885856 |
| SPOCK3 | Variation_92528 | chr4:168022352..168030675 |
| SPOCK3 | Variation_92529 | chr4:168107097..168115035 |
| SPOCK3 | Variation_92530 | chr4:168109203..168115035 |
| SPOCK3 | Variation_92531 | chr4:168109203..168110985 |
| NEK1 | Variation_92539 | chr4:170341498..170345368 |
| FAM149A | Variation_92622 | chr4:187093560..187101136 |
| FAM149A | Variation_92623 | chr4:187093560..187099134 |
| BAI3 | Variation_93799 | chr6:70014070..70018333 |
| PARK2 | Variation_94156 | chr6:161812014..161814611 |
| PARK2 | Variation_94158 | chr6:162385216..162386511 |
| PARK2 | Variation_94161 | chr6:162496229..162498161 |
| PARK2 | Variation_94162 | chr6:162493653..162498161 |
| PARK2 | Variation_94164 | chr6:162498331..162506785 |
| PARK2 | Variation_94165 | chr6:162501453..162503861 |
| PARK2 | Variation_94166 | chr6:162501453..162506097 |
| PARK2 | Variation_94167 | chr6:162578570..162580138 |
| PARK2 | Variation_94169 | chr6:162582833..162586655 |
| PARK2 | Variation_94170 | chr6:162583613..162586655 |
| PARK2 | Variation_94171 | chr6:162584549..162586655 |
| PARK2 | Variation_94175 | chr6:162983879..162992037 |
| PARK2 | Variation_94176 | chr6:162976503..163015462 |
| PARK2 | Variation_94177 | chr6:162999414..163007555 |
| PARK2 | Variation_94178 | chr6:163001658..163013624 |
| PARK2 | Variation_94179 | chr6:162983879..162987575 |
| PARK2 | Variation_94180 | chr6:162983879..162995683 |
| PARK2 | Variation_94181 | chr6:163005335..163011995 |
| PDE10A | Variation_94191 | chr6:165878708..165880754 |
| THBS2 | Variation_94253 | chr6:169633491..169636529 |
| THBS2 | Variation_94255 | chr6:169634793..169635847 |
| THBS2 | Variation_94256 | chr6:169633491..169634607 |
| PCLO | Variation_94656 | chr7:82734323..82806353 |
| SEMA3A | Variation_94658 | chr7:83782188..83786707 |
| PDGFRL | Variation_95294 | chr8:17437467..17446394 |
| ESCO2 | Variation_95339 | chr8:27621290..27701118 |
| MMP16 | Variation_95557 | chr8:89098396..89099768 |
| KIAA1429 | Variation_95570 | chr8:95558349..95561289 |
| UQCRB | Variation_95582 | chr8:97239539..97242185 |
| TRAPPC9 | Variation_95734 | chr8:140962380..140967280 |
| TRAPPC9 | Variation_95735 | chr8:140965476..140967280 |
| TRAPPC9 | Variation_95736 | chr8:141205077..141206743 |
| TRAPPC9 | Variation_95738 | chr8:141344972..141346376 |
| TRAPPC9 | Variation_95739 | chr8:141344972..141347618 |
| MAMDC2 | Variation_96103 | chr9:72762326..72763496 |
| MAMDC2 | Variation_96104 | chr9:72762326..72764237 |
| SPOCD1 | Variation_97328 | chr1:32272603..32273628 |
| TMEM131 | Variation_97918 | chr2:98572005..98573421 |
| TSGA10 | Variation_97920 | chr2:99737464..99738502 |
| REV1 | Variation_97921 | chr2:100102701..100105388 |
| CREG2 | Variation_97922 | chr2:101974610..101976404 |
| NCK2 | Variation_97930 | chr2:106452076..106453084 |
| NCKAP5 | Variation_97991 | chr2:133873134..133874626 |
| TMEM163 | Variation_97995 | chr2:135370267..135371658 |
| ZRANB3 | Variation_97996 | chr2:136097994..136102356 |
| UBR3 | Variation_98054 | chr2:170819797..170821200 |
| C2orf88 | Variation_98080 | chr2:191035912..191037410 |
| C2orf88 | Variation_98081 | chr2:191065475..191066733 |
| PLCL1 | Variation_98099 | chr2:198730108..198731570 |
| PLCL1 | Variation_98100 | chr2:198762771..198763790 |
| FAM117B | Variation_98108 | chr2:203633021..203634346 |
| MAP2 | Variation_98124 | chr2:210507937..210509248 |
| ABI3BP | Variation_98392 | chr3:100668595..100671661 |
| GSK3B | Variation_98419 | chr3:119601185..119602595 |
| GSK3B | Variation_98420 | chr3:119683848..119685428 |
| GPR156 | Variation_98421 | chr3:119893011..119894850 |
| IQCB1 | Variation_98423 | chr3:121522240..121524036 |
| DIRC2 | Variation_98426 | chr3:122559734..122561364 |
| SLC12A8 | Variation_98433 | chr3:124889317..124891418 |
| EPHB1 | Variation_98456 | chr3:134673390..134675383 |
| CLSTN2 | Variation_98459 | chr3:139673883..139674971 |
| CLSTN2 | Variation_98460 | chr3:140267241..140270128 |
| VPS8 | Variation_98556 | chr3:184613030..184614126 |
| DLG1 | Variation_98593 | chr3:196810825..196812211 |
| DLG1 | Variation_98594 | chr3:196880100..196881567 |
| LRCH3 | Variation_98596 | chr3:197590573..197592129 |
| NCAPG | Variation_98638 | chr4:17829859..17831473 |
| SLIT2 | Variation_98646 | chr4:20527710..20529201 |
| FAM13A | Variation_98792 | chr4:89680841..89682346 |
| FAM13A | Variation_98793 | chr4:89685434..89687035 |
| SPOCK3 | Variation_98951 | chr4:167676204..167683524 |
| SPOCK3 | Variation_98952 | chr4:168109584..168115813 |
| NEK1 | Variation_98957 | chr4:170450316..170451787 |
| STOX2 | Variation_98984 | chr4:184852821..184854318 |
| SNX25 | Variation_98991 | chr4:186154816..186156197 |
| SNX25 | Variation_98992 | chr4:186198447..186199881 |
| FAM149A | Variation_98996 | chr4:187093075..187098728 |
| SH3PXD2B | Variation_99355 | chr5:171795072..171796754 |
| BAI3 | Variation_99578 | chr6:69470728..69472243 |
| PARK2 | Variation_99782 | chr6:161856622..161857626 |
| PARK2 | Variation_99783 | chr6:162150710..162152398 |
| WDR27 | Variation_99816 | chr6:170037674..170039010 |
| PCLO | Variation_100007 | chr7:82546963..82548317 |
| SEMA3E | Variation_100008 | chr7:83018407..83019984 |
| SEMA3A | Variation_100009 | chr7:83750456..83752054 |
| TMEM55A | Variation_100421 | chr8:92036722..92038168 |
| TRAPPC9 | Variation_100527 | chr8:140759377..140761252 |
| TRAPPC9 | Variation_100528 | chr8:140884159..140885741 |
| TRAPPC9 | Variation_100529 | chr8:141119873..141121390 |
| FREM1 | Variation_100591 | chr9:14860384..14861998 |
| EXD3 | Variation_100811 | chr9:140252992..140256615 |
| WDFY4 | Variation_100941 | chr10:49916628..49918127 |
| SGMS1 | Variation_100945 | chr10:52132081..52133610 |
| PCDH15 | Variation_100953 | chr10:55957523..55959080 |
| PCDH15 | Variation_100954 | chr10:56516828..56518428 |
| STOX1 | Variation_100973 | chr10:70586686..70588335 |
| ADK | Variation_100982 | chr10:76023897..76025251 |
| ADK | Variation_100983 | chr10:76267951..76269446 |
| ADK | Variation_100984 | chr10:76288827..76290355 |
| C10orf11 | Variation_100985 | chr10:77703336..77704729 |
| C10orf11 | Variation_100986 | chr10:78255420..78259649 |
| TSPAN14 | Variation_100994 | chr10:82241383..82243153 |
| NRG3 | Variation_100996 | chr10:84127090..84130827 |
| C10orf26 | Variation_101029 | chr10:104527632..104529299 |
| AS3MT | Variation_101030 | chr10:104637475..104638734 |
| ADD3 | Variation_101043 | chr10:111813760..111815018 |
| ATRNL1 | Variation_101049 | chr10:117147774..117149213 |
| FANK1 | Variation_101065 | chr10:127575297..127595487 |
| FANK1 | Variation_101066 | chr10:127595487..127616432 |
| DOCK1 | Variation_101071 | chr10:129160716..129161831 |
| INPP5A | Variation_101092 | chr10:134584855..134586687 |
| KNDC1 | Variation_101098 | chr10:135035290..135037604 |
| DEAF1 | Variation_101102 | chr11:676904..678528 |
| ZNF215 | Variation_101121 | chr11:6955757..6957218 |
| TMEM41B | Variation_101128 | chr11:9323235..9325585 |
| PHF21A | Variation_101208 | chr11:46036001..46037776 |
| PATL1 | Variation_101231 | chr11:59412151..59413213 |
| PC | Variation_101244 | chr11:66710780..66713909 |
| C12orf40 | Variation_101486 | chr12:40103627..40107201 |
| PDZRN4 | Variation_101493 | chr12:41659285..41660883 |
| PDZRN4 | Variation_101494 | chr12:41847300..41848331 |
| PDZRN4 | Variation_101495 | chr12:41872658..41874071 |
| GLTP | Variation_101616 | chr12:110305455..110306465 |
| PDS5B | Variation_101695 | chr13:33261394..33262694 |
| PDS5B | Variation_101696 | chr13:33339652..33341199 |
| LCP1 | Variation_101725 | chr13:46719190..46720677 |
| DIS3 | Variation_101775 | chr13:73342892..73344268 |
| HEATR5A | Variation_101913 | chr14:31813177..31814588 |
| SPATA7 | Variation_102005 | chr14:88859184..88860625 |
| SPATA7 | Variation_102006 | chr14:88889232..88890717 |
| GNB5 | Variation_102103 | chr15:52429466..52430919 |
| PIAS1 | Variation_102124 | chr15:68425887..68429963 |
| PTPN9 | Variation_102137 | chr15:75866167..75867820 |
| SPIRE2 | Variation_102365 | chr16:89909406..89910456 |
| AATF | Variation_102449 | chr17:35378327..35379870 |
| WIPF2 | Variation_102452 | chr17:38377416..38378912 |
| STAT5B | Variation_102459 | chr17:40393008..40394563 |
| CDC27 | Variation_102470 | chr17:45213766..45216342 |
| CBX1 | Variation_102471 | chr17:46159193..46162780 |
| LUC7L3 | Variation_102477 | chr17:48821163..48822467 |
| LUC7L3 | Variation_102478 | chr17:48828939..48830439 |
| TOM1L1 | Variation_102484 | chr17:52996472..52997927 |
| MSI2 | Variation_102492 | chr17:55687919..55689919 |
| PITPNC1 | Variation_102512 | chr17:65520307..65521593 |
| NOL11 | Variation_102513 | chr17:65726356..65728015 |
| FAM104A | Variation_102529 | chr17:71216746..71218435 |
| SDK2 | Variation_102530 | chr17:71369656..71371469 |
| SRP68 | Variation_102537 | chr17:74046132..74047180 |
| TBC1D16 | Variation_102552 | chr17:77997289..77998319 |
| TBCD | Variation_102563 | chr17:80722120..80724040 |
| SLC14A2 | Variation_102651 | chr18:43260612..43261967 |
| ZNF516 | Variation_102717 | chr18:74104289..74107159 |
| NPAS1 | Variation_102855 | chr19:47527817..47529631 |
| COMMD7 | Variation_102953 | chr20:31310129..31313534 |
| VSTM2L | Variation_102963 | chr20:36542171..36543217 |
| CACNG2 | Variation_103170 | chr22:37076400..37077407 |
| CYB5R3 | Variation_103183 | chr22:43030020..43031642 |
| SCUBE1 | Variation_103185 | chr22:43676328..43678084 |
| CES1 | Variation_103337 | chr16:55813721..55850377 |
| SPOCK3 | Variation_103358 | chr4:168114868..168116272 |
| ZRANB3 | Variation_103398 | chr2:136098433..136101806 |
| ABI3BP | Variation_103463 | chr3:100669517..100673226 |
| EPHB1 | Variation_103472 | chr3:134842625..134844843 |
| PPP2R3A | Variation_103473 | chr3:135697373..135699877 |
| CCDC50 | Variation_103495 | chr3:191065392..191069984 |
| SLIT2 | Variation_103507 | chr4:20556711..20565861 |
| MTHFD2L | Variation_103529 | chr4:75081112..75082206 |
| MAPK10 | Variation_103535 | chr4:86976094..86979944 |
| BAI3 | Variation_103670 | chr6:69687698..69691368 |
| PARK2 | Variation_103693 | chr6:162496291..162506568 |
| PARK2 | Variation_103694 | chr6:162737217..162740440 |
| PTK2 | Variation_103828 | chr8:142003000..142004909 |
| NRG3 | Variation_103895 | chr10:83884660..83888667 |
| NRG3 | Variation_103897 | chr10:84712898..84717172 |
| SYCE1 | Variation_103909 | chr10:135242873..135377448 |
| STRC | Variation_104079 | chr15:43885118..43988553 |
| KIAA1267 | Variation_104125 | chr17:44165803..44362009 |
| MKL1 | Variation_104223 | chr22:40873576..40880692 |
| NPEPPS | Variation_104306 | chr17:45611031..45671632 |
| MSI2 | Variation_104312 | chr17:55687760..55689904 |
| PQLC1 | Variation_104381 | chr18:77679094..77682254 |
| BCL2L13 | Variation_104507 | chr22:18141502..18144784 |
| SCUBE1 | Variation_104527 | chr22:43676579..43677609 |
| PARVB | Variation_104528 | chr22:44508424..44512124 |
| REV1 | Variation_104600 | chr2:100103534..100105532 |
| TMEM163 | Variation_104623 | chr2:135364265..135367580 |
| TANC1 | Variation_104629 | chr2:159959794..159961032 |
| CCDC50 | Variation_104721 | chr3:191064641..191071711 |
| MUC4 | Variation_104732 | chr3:195474253..195477817 |
| MUC4 | Variation_104734 | chr3:195501958..195504963 |
| MUC4 | Variation_104735 | chr3:195504964..195515622 |
| DLG1 | Variation_104737 | chr3:196934570..196939504 |
| LMLN | Variation_104742 | chr3:197730419..197732220 |
| SLIT2 | Variation_104765 | chr4:20407568..20408857 |
| SPOCK3 | Variation_104815 | chr4:168109009..168115505 |
| FAM149A | Variation_104825 | chr4:187093421..187098288 |
| THBS2 | Variation_104987 | chr6:169633222..169635277 |
| SEMA3E | Variation_105041 | chr7:83018451..83019679 |
| PCDH15 | Variation_105235 | chr10:56445892..56469623 |
| ADAMTS14 | Variation_105244 | chr10:72448465..72451426 |
| C10orf11 | Variation_105245 | chr10:78255480..78261009 |
| LHPP | Variation_105259 | chr10:126194790..126196530 |
| FANK1 | Variation_105262 | chr10:127573369..127617461 |
| INPP5A | Variation_105270 | chr10:134584997..134586392 |
| UTF1 | Variation_105271 | chr10:135043532..135045028 |
| PC | Variation_105311 | chr11:66711949..66713416 |
| KIAA0284 | Variation_105463 | chr14:105350248..105351582 |
| SLCO3A1 | Variation_105499 | chr15:92674890..92677336 |
| TGFBR3 | Variation_105661 | chr1:92227910..92236935 |
| PCLO | Variation_105743 | chr7:82783538..82786843 |
| SEMA3A | Variation_105744 | chr7:83749438..83759598 |
| CYHR1 | Variation_105781 | chr8:145679339..145687926 |
| PDGFRL | Variation_105784 | chr8:17425061..17435328 |
| FAM160B2 | Variation_105788 | chr8:21944161..21950037 |
| SLC39A14 | Variation_105789 | chr8:22232651..22244316 |
| EXTL3 | Variation_105792 | chr8:28573707..28579325 |
| TCERG1L | Variation_105794 | chr10:132987990..132995588 |
| INPP5A | Variation_105827 | chr10:134564949..134573295 |
| EXD3 | Variation_105853 | chr9:140253441..140262760 |
| TGFBR3 | Variation_105920 | chr1:92232063..92233334 |
| PIAS1 | Variation_105972 | chr15:68426002..68428926 |
| SLCO3A1 | Variation_105974 | chr15:92674574..92677070 |
| SPIRE2 | Variation_105978 | chr16:89896057..89898389 |
| MUC4 | Variation_106024 | chr3:195513136..195515007 |
| SAMD8 | Variation_106147 | chr10:76886533..76895069 |
| SPIRE2 | Variation_106193 | chr16:89896467..89898443 |
| SPIRE2 | Variation_106416 | chr16:89896363..89898402 |
| CLSTN2 | Variation_106495 | chr3:139699689..139700865 |
| LMLN | Variation_106504 | chr3:197730393..197732154 |
| YPEL4 | Variation_106670 | chr11:57408294..57419351 |
| EHD1 | Variation_106736 | chr11:64624637..64626194 |
| LHPP | Variation_106746 | chr10:126193644..126197922 |
| EFEMP2 | Variation_106759 | chr11:65636167..65646034 |
| SGMS1 | Variation_106761 | chr10:52316959..52319772 |
| KIAA1279 | Variation_106767 | chr10:70753093..70754041 |
| SH2D4B | Variation_106771 | chr10:82385340..82389811 |
| EXOC6 | Variation_106775 | chr10:94803998..94804843 |
| SHANK2 | Variation_106803 | chr11:70707512..70720840 |
| MRPL21 | Variation_106806 | chr11:68654481..68658820 |
| RIC3 | Variation_106815 | chr11:8180112..8184789 |
| SCUBE2 | Variation_106816 | chr11:9104014..9107607 |
| IFT81 | Variation_106828 | chr12:110583263..110586744 |
| KSR2 | Variation_106830 | chr12:118186880..118186998 |
| TMEM41B | Variation_106925 | chr11:9316963..9326487 |
| ADAMTS7 | Variation_106944 | chr15:79094475..79098614 |
| CBX1 | Variation_106990 | chr17:46166496..46167737 |
| APPBP2 | Variation_106999 | chr17:58529494..58533091 |
| SLC39A11 | Variation_107002 | chr17:70856121..70858636 |
| C12orf51 | Variation_107069 | chr12:112603929..112610748 |
| NOSTRIN | Variation_107081 | chr2:169720738..169728304 |
| KSR2 | Variation_107102 | chr12:118240136..118249923 |
| MGAT4A | Variation_107119 | chr2:99272430..99276851 |
| PLA2G6 | Variation_107169 | chr22:38504989..38508331 |
| SLC12A8 | Variation_107180 | chr3:124808349..124810048 |
| ZXDC | Variation_107182 | chr3:126179634..126180184 |
| MED12L | Variation_107188 | chr3:151133607..151150535 |
| MUC4 | Variation_107199 | chr3:195511871..195512569 |
| MTHFD2L | Variation_107278 | chr4:75090225..75095112 |
| WDFY3 | Variation_107284 | chr4:85729594..85733898 |
| PARK2 | Variation_107358 | chr6:162717232..162720484 |
| WDR27 | Variation_107362 | chr6:170067846..170068512 |
| BAI3 | Variation_107378 | chr6:69915372..69915795 |
| TRAPPC9 | Variation_107443 | chr8:140972347..140973376 |
| FRMD3 | Variation_107509 | chr9:85931170..85931213 |
| PCDH15 | Variation_107547 | chr10:56484331..56485762 |
| C10orf11 | Variation_107548 | chr10:78256184..78258893 |
| PC | Variation_107554 | chr11:66711949..66713416 |
| MSI2 | Variation_107577 | chr17:55687760..55689836 |
| REV1 | Variation_107600 | chr2:100103740..100105065 |
| CCDC50 | Variation_107610 | chr3:191064641..191071649 |
| FAM149A | Variation_107616 | chr4:187093489..187098205 |
| PCDH15 | Variation_107699 | chr10:56475412..56486949 |
| C10orf11 | Variation_107700 | chr10:78255876..78261009 |
| NPEPPS | Variation_107764 | chr17:45632210..45673517 |
| MSI2 | Variation_107765 | chr17:55687846..55689722 |
| REV1 | Variation_107812 | chr2:100103724..100105472 |
| CCDC50 | Variation_107839 | chr3:191064635..191071632 |
| MUC4 | Variation_107840 | chr3:195505473..195515557 |
| SPOCK3 | Variation_107849 | chr4:168109174..168115058 |
| FAM149A | Variation_107850 | chr4:187093611..187098288 |
| EXD3 | Variation_107939 | chr9:140223139..140224239 |
| PIAS1 | Variation_108668 | chr15:68424185..68430193 |
| SLCO3A1 | Variation_108801 | chr15:92674359..92677641 |
| SPIRE2 | Variation_109257 | chr16:89892502..89899680 |
| CNTNAP1 | Variation_109557 | chr17:40832914..40846845 |
| MSI2 | Variation_109756 | chr17:55634417..55645099 |
| MSI2 | Variation_109767 | chr17:55682182..55691964 |
| PPM1E | Variation_109778 | chr17:57035250..57044310 |
| BCAS3 | Variation_110110 | chr17:59368321..59386514 |
| NCK2 | Variation_110118 | chr2:106383990..106399576 |
| TSGA10 | Variation_110125 | chr2:99621242..99638545 |
| SETBP1 | Variation_110186 | chr18:42434771..42446121 |
| SLC14A2 | Variation_110187 | chr18:43258840..43265441 |
| TANC1 | Variation_110299 | chr2:159959220..159962368 |
| PLEKHA3 | Variation_110311 | chr2:179357045..179365143 |
| PLCL1 | Variation_110324 | chr2:198943569..198951256 |
| ALS2CR12 | Variation_110327 | chr2:202146558..202153252 |
| RNF181 | Variation_110384 | chr2:85822210..85825244 |
| COMMD7 | Variation_110397 | chr20:31306861..31313624 |
| CECR5 | Variation_110455 | chr22:17625310..17628587 |
| BCL2L13 | Variation_110457 | chr22:18139914..18150356 |
| SPECC1L | Variation_110464 | chr22:24805586..24812738 |
| SUN2 | Variation_110473 | chr22:39125941..39136890 |
| ATG7 | Variation_110488 | chr3:11469780..11478322 |
| ATG7 | Variation_110489 | chr3:11587312..11596051 |
| MYLK | Variation_110493 | chr3:123353556..123359270 |
| SPOCK3 | Variation_110590 | chr4:167676464..167683095 |
| FAM149A | Variation_110608 | chr4:187092077..187099467 |
| SLIT2 | Variation_110617 | chr4:20399974..20409944 |
| ADD1 | Variation_110621 | chr4:2904137..2914601 |
| EVC2 | Variation_110630 | chr4:5656937..5664583 |
| WFS1 | Variation_110635 | chr4:6277551..6293246 |
| MAP3K13 | Variation_110743 | chr3:185106729..185109266 |
| MFI2 | Variation_110779 | chr3:196676698..196754319 |
| DLG1 | Variation_110781 | chr3:196933924..196939504 |
| SLBP | Variation_110794 | chr4:1494917..1897303 |
| WHSC2 | Variation_110798 | chr4:1992967..2082167 |
| REST | Variation_110881 | chr4:57768655..57779715 |
| HNRPDL | Variation_110925 | chr4:83344392..83346058 |
| SPOCK3 | Variation_111060 | chr4:167664428..167670849 |
| MFAP3L | Variation_111065 | chr4:170946477..170948893 |
| STOX2 | Variation_111104 | chr4:184826103..184828561 |
| ACSL1 | Variation_111106 | chr4:185745099..185748742 |
| HELT | Variation_111107 | chr4:185937760..185942783 |
| FAM149A | Variation_111112 | chr4:187093421..187098288 |
| MAP1B | Variation_111238 | chr5:71484306..71485465 |
| PARK2 | Variation_111744 | chr6:162583879..162586804 |
| PARK2 | Variation_111745 | chr6:162706014..162707222 |
| PARK2 | Variation_111746 | chr6:162734724..162741078 |
| PARK2 | Variation_111747 | chr6:162737988..162740241 |
| PDE10A | Variation_111750 | chr6:166072518..166078519 |
| TSPAN13 | Variation_111816 | chr7:16794489..16800589 |
| SEMA3E | Variation_111913 | chr7:83018451..83019679 |
| SEMA3E | Variation_111915 | chr7:83151475..83152744 |
| SEMA3A | Variation_111916 | chr7:83708277..83709998 |
| CNPY1 | Variation_112046 | chr7:155124154..155307408 |
| PDGFRL | Variation_112132 | chr8:17480466..17481629 |
| DPYSL2 | Variation_112145 | chr8:26431509..26441275 |
| TRAPPC9 | Variation_112258 | chr8:140695133..140743056 |
| KIAA1875 | Variation_112300 | chr8:145160213..145284762 |
| KIAA1875 | Variation_112301 | chr8:145171456..145218648 |
| TUSC1 | Variation_112368 | chr9:25667989..25682355 |
| MAMDC2 | Variation_112400 | chr9:72762006..72764750 |
| KIAA1984 | Variation_112559 | chr9:139700812..139718065 |
| NRARP | Variation_112567 | chr9:140192743..140197617 |
| PCDH15 | Variation_112693 | chr10:56445892..56469590 |
| ZNF365 | Variation_112706 | chr10:64159270..64160432 |
| SIRT1 | Variation_112724 | chr10:69649937..69651124 |
| ADAMTS14 | Variation_112730 | chr10:72448465..72451426 |
| ADAMTS14 | Variation_112732 | chr10:72425578..72484902 |
| ADAMTS14 | Variation_112733 | chr10:72448668..72449925 |
| C10orf11 | Variation_112739 | chr10:78255480..78261009 |
| C10orf11 | Variation_112740 | chr10:78256184..78258893 |
| NRG3 | Variation_112746 | chr10:83776588..83780093 |
| NRG3 | Variation_112747 | chr10:84127856..84129645 |
| SUFU | Variation_112801 | chr10:104289771..104290836 |
| RBM20 | Variation_112813 | chr10:112438364..112441451 |
| HTRA1 | Variation_112837 | chr10:124193780..124265225 |
| FANK1 | Variation_112849 | chr10:127573369..127617461 |
| GLRX3 | Variation_112856 | chr10:131948334..131981375 |
| TCERG1L | Variation_112860 | chr10:132909004..132912774 |
| TCERG1L | Variation_112863 | chr10:133080536..133133835 |
| TCERG1L | Variation_112865 | chr10:133097512..133113315 |
| UTF1 | Variation_112881 | chr10:135043532..135045028 |
| PRRG4 | Variation_112983 | chr11:32849904..32852165 |
| SLC39A13 | Variation_113012 | chr11:47376020..47457537 |
| SF1 | Variation_113058 | chr11:64538092..64550927 |
| MEN1 | Variation_113059 | chr11:64577366..64591157 |
| CATSPER1 | Variation_113063 | chr11:65792840..65803470 |
| PC | Variation_113067 | chr11:66711949..66713416 |
| TPCN2 | Variation_113078 | chr11:68811909..68817253 |
| TPCN2 | Variation_113079 | chr11:68783467..68856440 |
| FGF3 | Variation_113080 | chr11:69434047..69666065 |
| ORAOV1 | Variation_113081 | chr11:69444561..69524415 |
| ARHGAP20 | Variation_113156 | chr11:110581104..110584444 |
| GRIN2B | Variation_113263 | chr12:13785488..13878736 |
| PDZRN4 | Variation_113324 | chr12:41752718..41754247 |
| PDZRN4 | Variation_113325 | chr12:41878846..41911495 |
| ANO6 | Variation_113330 | chr12:45691091..45740769 |
| MBNL1 | Variation_0037 | chr3:152019577..152215010 |
| TFRC | Variation_0042 | chr3:195722630..195886300 |
| LMLN | Variation_0044 | chr3:197673380..197873380 |
| KIAA1324L | Variation_0106 | chr7:86655838..86802722 |
| SLC39A5 | Variation_0164 | chr12:56553078..56902907 |
| TEX2 | Variation_0210 | chr17:62118842..62273472 |
| SFI1 | Variation_0236 | chr22:31797009..31965118 |
| NOSTRIN | Variation_0533 | chr2:169721323..169739365 |
| SPECC1L | Variation_0688 | chr22:24509077..24722933 |
| ARNT2 | Variation_0787 | chr15:80823202..81003389 |
| FSD2 | Variation_0789 | chr15:83417765..83560629 |
| NPEPPS | Variation_0804 | chr17:45511584..45698942 |
| CATSPER2 | Variation_1282 | chr15:43888976..43939642 |
| IQCJ | Variation_1646 | chr3:158774340..158792286 |
| WFS1 | Variation_2067 | chr4:6211749..6386097 |
| ZMIZ1 | Variation_2158 | chr10:80802829..80972426 |
| TSPAN14 | Variation_2160 | chr10:82166752..82315290 |
| SYCE1 | Variation_2162 | chr10:135228871..135390508 |
| TRIM21 | Variation_2164 | chr11:4333543..4474341 |
| GGNBP2 | Variation_2223 | chr17:34875147..35040221 |
| KIAA1267 | Variation_2225 | chr17:44046672..44204487 |
| NPEPPS | Variation_2229 | chr17:45511584..45698942 |
| ZNF45 | Variation_2245 | chr19:44405994..44555426 |
| COMT | Variation_2261 | chr22:19879187..20055258 |
| CRYBB3 | Variation_2270 | chr22:25598912..25762849 |
| TRIM42 | Variation_2475 | chr3:140009220..140460228 |
| FETUB | Variation_2488 | chr3:186370350..186427340 |
| PARK2 | Variation_2665 | chr6:162840922..163233261 |
| EFHA2 | Variation_2742 | chr8:16259141..16905525 |
| PRRG4 | Variation_2915 | chr11:32792784..32958609 |
| PPIP5K1 | Variation_3079 | chr15:43831923..44058634 |
| GGNBP2 | Variation_3143 | chr17:34944593..35030559 |
| EIF1 | Variation_3145 | chr17:39653122..39896843 |
| ZCCHC17 | Variation_3287 | chr1:31722140..32356850 |
| TRIM42 | Variation_3450 | chr3:139925764..140524730 |
| MED12L | Variation_3456 | chr3:150791625..150957485 |
| PTX3 | Variation_3459 | chr3:157090166..157254684 |
| SNORA63 | Variation_3467 | chr3:186337134..186567326 |
| DLG1 | Variation_3473 | chr3:196855053..197497868 |
| GABBR1 | Variation_3598 | chr6:29469101..29678670 |
| BRD2 | Variation_3604 | chr6:32865789..32964096 |
| EFHA2 | Variation_3723 | chr8:16187558..16966262 |
| BMS1 | Variation_3797 | chr10:42965131..43510191 |
| HTRA1 | Variation_3823 | chr10:124263075..124454815 |
| INPP5A | Variation_3827 | chr10:134226230..134427067 |
| SYCE1 | Variation_3830 | chr10:135222326..135390508 |
| DEAF1 | Variation_3831 | chr11:409846..1357392 |
| ASCL3 | Variation_3837 | chr11:8784813..9009573 |
| PRRG4 | Variation_3843 | chr11:32714643..33049940 |
| PSTPIP1 | Variation_3969 | chr15:77170550..77373448 |
| C15orf58 | Variation_3976 | chr15:90565956..90789225 |
| SMARCE1 | Variation_4035 | chr17:38748300..38850104 |
| KPNB1 | Variation_4039 | chr17:45520374..45764956 |
| EIF3D | Variation_4126 | chr22:36906563..37042034 |
| TRIOBP | Variation_4128 | chr22:38003877..38265878 |
| PKDREJ | Variation_4133 | chr22:46654943..46697976 |
| C2orf55 | Variation_4301 | chr2:99533833..99709999 |
| DOK7 | Variation_4371 | chr4:3444216..3623031 |
| SLIT2 | Variation_4380 | chr4:20502895..20680956 |
| BEST2 | Variation_4400 | chr19:12847630..13058491 |
| PARK2 | Variation_4515 | chr6:163064811..163245813 |
| FREM1 | Variation_4615 | chr9:14596657..14739902 |
| INPP5A | Variation_4718 | chr10:134557240..134684459 |
| C10orf125 | Variation_4721 | chr10:134903013..135181736 |
| KNDC1 | Variation_4723 | chr10:134975213..135081168 |
| C10orf125 | Variation_4724 | chr10:135115064..135226145 |
| PTPMT1 | Variation_4741 | chr11:47517955..47681310 |
| MED19 | Variation_4744 | chr11:57299354..57481050 |
| MAP4K2 | Variation_4753 | chr11:64487183..64620199 |
| ORAOV1 | Variation_4755 | chr11:69423683..69588478 |
| C12orf49 | Variation_4788 | chr12:117090343..117273810 |
| FBXO21 | Variation_4789 | chr12:117612666..117652676 |
| PITPNM2 | Variation_4790 | chr12:123395927..123589290 |
| MTRF1 | Variation_4799 | chr13:41604931..41798808 |
| TDP1 | Variation_4844 | chr14:90285287..90456676 |
| RCCD1 | Variation_4909 | chr15:91454426..91641749 |
| SPIRE2 | Variation_4978 | chr16:89792499..89916214 |
| KIAA1267 | Variation_5011 | chr17:44163719..44288338 |
| KIAA1267 | Variation_5012 | chr17:44235152..44363802 |
| SPATA20 | Variation_5017 | chr17:48594242..48755830 |
| CACNG5 | Variation_5027 | chr17:64855019..65023735 |
| AATK | Variation_5036 | chr17:78986372..79147582 |
| ZSWIM4 | Variation_5088 | chr19:13836718..14062800 |
| VSTM2L | Variation_5136 | chr20:36445761..36537911 |
| COMT | Variation_5168 | chr22:19887966..20069970 |
| SCARF2 | Variation_5169 | chr22:20759359..20951242 |
| CYTH4 | Variation_5184 | chr22:37689046..37815500 |
| PLA2G6 | Variation_5185 | chr22:38388465..38549017 |
| SLC16A8 | Variation_5186 | chr22:38459503..38636510 |
| ZXDC | Variation_5213 | chr3:126185386..126236700 |
| RILPL1 | Variation_5313 | chr12:124009676..124085689 |
| CRYBB2 | Variation_5357 | chr22:25512251..25799870 |
| TRAF2 | Variation_5415 | chr9:139478587..140244550 |
| C17orf104 | Variation_5864 | chr17:42747927..42754916 |
| SYCE1 | Variation_6852 | chr10:135236012..135387833 |
| SCUBE2 | Variation_6953 | chr11:9007612..9311131 |
| NPEPPS | Variation_7163 | chr17:45551959..45664575 |
| CEP63 | Variation_7364 | chr3:134201034..134210747 |
| CLSTN2 | Variation_8429 | chr3:140009020..140469302 |
| MUC4 | Variation_8439 | chr3:195433543..195651349 |
| PCLO | Variation_8571 | chr7:81957637..82460949 |
| EFHA2 | Variation_8599 | chr8:16285891..16888339 |
| SYCE1 | Variation_8671 | chr10:135184119..135434551 |
| SYCE1 | Variation_8673 | chr10:135079862..135434551 |
| QSER1 | Variation_8685 | chr11:32796092..33004024 |
| CDC45 | Variation_8901 | chr22:19423250..19766782 |
| MYO18B | Variation_8904 | chr22:25658546..26150646 |
| LMO7 | Variation_9224 | chr13:76144199..76288418 |
| FSD2 | Variation_9266 | chr15:83283555..83453495 |
| RASGRP2 | Variation_9667 | chr11:64345448..64546391 |
| CYTH4 | Variation_9846 | chr22:37679671..37807303 |
| SCHIP1 | Variation_10016 | chr3:159461456..159466206 |
| FSD2 | Variation_10488 | chr15:83299461..83457424 |
| PTGER1 | Variation_10543 | chr19:14580113..14590612 |
| HEATR7A | Variation_23291 | chr8:145276232..145513753 |
| CYHR1 | Variation_23292 | chr8:145682115..145750506 |
| KIAA1984 | Variation_23294 | chr9:139690144..139754110 |
| SYCE1 | Variation_29602 | chr10:135202594..135434303 |
| ANKRD9 | Variation_29634 | chr14:102933347..103031831 |
| FDX1L | Variation_29685 | chr19:10399904..10471977 |
| RAVER1 | Variation_29686 | chr19:10435493..10472933 |
| INPP5A | Variation_29874 | chr10:134512520..134627882 |
| RANBP1 | Variation_30155 | chr22:20100596..20189077 |
| AIFM3 | Variation_30158 | chr22:21331556..21353328 |
| LZTR1 | Variation_30159 | chr22:21344316..21387967 |
| CYTH4 | Variation_30162 | chr22:37692938..37771985 |
| EEFSEC | Variation_30181 | chr3:128046643..128159359 |
| TNIP2 | Variation_30193 | chr4:2701098..2804476 |
| DOK7 | Variation_30195 | chr4:3429856..3498170 |
| VPS28 | Variation_30308 | chr8:145641564..145668443 |
| VPS28 | Variation_30309 | chr8:145641564..145690308 |
| TRAF2 | Variation_30350 | chr9:139254317..139950351 |
| ZNF365 | Variation_30508 | chr10:63712167..64312757 |
| KNDC1 | Variation_30525 | chr10:135035825..135068932 |
| TSSK2 | Variation_31071 | chr22:19019088..21053198 |
| CRYBB3 | Variation_31083 | chr22:25603009..25642424 |
| DOK7 | Variation_31153 | chr4:3438682..3467338 |
| EFHA2 | Variation_31431 | chr8:16289362..16889243 |
| SSB | Variation_32382 | chr2:170649846..170957968 |
| SYCE1 | Variation_34537 | chr10:135184421..135434551 |
| SYCE1 | Variation_34865 | chr10:135202013..135386618 |
| POLD4 | Variation_35003 | chr11:67106503..67122531 |
| IPO4 | Variation_35245 | chr14:24653203..24665973 |
| CIRH1A | Variation_35397 | chr16:69196363..69229421 |
| EXOC7 | Variation_35500 | chr17:74066266..74083883 |
| KATNAL2 | Variation_35551 | chr18:44547795..44549761 |
| KATNAL2 | Variation_35552 | chr18:44548847..44549701 |
| KATNAL2 | Variation_35562 | chr18:44553664..44554582 |
| KATNAL2 | Variation_35563 | chr18:44553857..44555636 |
| BAIAP2L2 | Variation_36026 | chr22:38501542..38509808 |
| HSD17B13 | Variation_36311 | chr4:88237813..88279021 |
| PDE10A | Variation_36486 | chr6:165715169..165747217 |
| EDC3 | Variation_37170 | chr15:74364360..75569130 |
| CES1 | Variation_37182 | chr16:55758124..55863908 |
| GGNBP2 | Variation_37189 | chr17:34814328..36297053 |
| ELMOD3 | Variation_37223 | chr2:85570014..85585825 |
| DLG1 | Variation_37243 | chr3:195397785..197386290 |
| HIC2 | Variation_38956 | chr22:21725998..21845294 |
| CECR2 | Variation_39309 | chr22:18030637..18044579 |
| SNORA4 | Variation_47856 | chr3:186414884..186754730 |
| EGLN3 | Variation_47862 | chr14:34232772..34629603 |
| OTUB2 | Variation_47863 | chr14:94490701..94627188 |
| CCDC130 | Variation_47866 | chr19:13414594..13866866 |
| PTPRE | Variation_47892 | chr10:129870293..130361981 |
| UBXN4 | Variation_47933 | chr2:136049982..136537952 |
| ZNF365 | Variation_47941 | chr10:63780945..64187564 |
| HEG1 | Variation_47990 | chr3:124764414..124868809 |
| RAB36 | Variation_48008 | chr22:23216762..23499513 |
| SYCE1 | Variation_48474 | chr10:135217956..135377448 |
| SYCE1 | Variation_48475 | chr10:135217956..135434303 |
| WDFY4 | Variation_48561 | chr10:50122181..50132639 |
| WDFY4 | Variation_48562 | chr10:50122181..50133442 |
| DDX21 | Variation_48614 | chr10:70691708..70775081 |
| PEX16 | Variation_48807 | chr11:45903194..45939641 |
| PEX16 | Variation_48808 | chr11:45903194..45975130 |
| PITPNM2 | Variation_48972 | chr12:123335527..123595163 |
| PITPNM2 | Variation_48973 | chr12:123335527..123626982 |
| SPRYD3 | Variation_49089 | chr12:53431010..53614993 |
| SPRYD3 | Variation_49090 | chr12:53431010..53621711 |
| SPRYD3 | Variation_49091 | chr12:53437806..53614993 |
| SPRYD3 | Variation_49092 | chr12:53437806..53621711 |
| SPRYD3 | Variation_49093 | chr12:53449321..53614993 |
| SPRYD3 | Variation_49094 | chr12:53449321..53621711 |
| CDAN1 | Variation_49555 | chr15:42983923..43023482 |
| PPP1R1B | Variation_49853 | chr17:37781849..37831035 |
| STARD3 | Variation_49854 | chr17:37814080..37831035 |
| STARD3 | Variation_49855 | chr17:37817482..37831035 |
| MRPL27 | Variation_49879 | chr17:48433958..48450536 |
| AIFM3 | Variation_50815 | chr22:21331556..21346719 |
| AIFM3 | Variation_50816 | chr22:21331556..21350880 |
| PMM1 | Variation_50866 | chr22:41648502..41981957 |
| C3orf37 | Variation_50924 | chr3:129012744..129035969 |
| TNIP2 | Variation_51374 | chr4:2723531..2836628 |
| DOK7 | Variation_51384 | chr4:3436588..3504562 |
| EFTUD1 | Variation_53109 | chr15:82454283..82573255 |
| SLC39A4 | Variation_53129 | chr8:145641564..145750506 |
| MEN1 | Variation_53146 | chr11:64477050..64584959 |
| DAPL1 | Variation_53156 | chr2:159651734..159953466 |
| C12orf40 | Variation_53524 | chr12:39946354..40042220 |
| TLR3 | Variation_53588 | chr4:186929226..187131504 |
| SYCE1 | Variation_53644 | chr10:135202594..135434303 |
| SYCE1 | Variation_53842 | chr10:135217956..135377448 |
| UPK2 | Variation_58497 | chr11:118829029..118849507 |
| IQCJ | Variation_59787 | chr3:158795690..158796173 |
| TGOLN2 | Variation_59896 | chr2:85543767..85570337 |
| IQCJ | Variation_63629 | chr3:158808243..158813097 |
| IQCJ | Variation_63630 | chr3:158898209..158900072 |
| SCHIP1 | Variation_63632 | chr3:159461407..159463400 |
| SECISBP2 | Variation_65451 | chr9:91963403..92343382 |
| SYCE1 | Variation_65826 | chr10:135237022..135400723 |
| APOBEC3F | Variation_67892 | chr22:39426703..39443920 |
| RMND5A | Variation_68073 | chr2:86972214..87174121 |
| MUC4 | Variation_68548 | chr3:195449862..195477757 |
| CYHR1 | Variation_70492 | chr8:145686297..145692418 |
| ZMIZ1 | Variation_71051 | chr10:80825979..80831421 |
| PARP2 | Variation_71865 | chr14:20800765..21257878 |
| KATNAL2 | Variation_73139 | chr18:44541908..44557900 |
| KATNAL2 | Variation_73140 | chr18:44545086..44552701 |
| ZP4 | Variation_74949 | chr1:238039394..238044833 |
| ZP4 | Variation_84920 | chr1:238039394..238044833 |
| SAMD14 | Variation_88524 | chr17:48182544..48197952 |
| SSB | Variation_90125 | chr2:170657417..170690977 |
| ST13 | Variation_91067 | chr22:41154286..41258558 |
| DIRC2 | Variation_91503 | chr3:122438244..122519976 |
| IQCJ | Variation_91666 | chr3:158808318..158813136 |
| IQCJ | Variation_91667 | chr3:158898478..158899801 |
| SCHIP1 | Variation_91668 | chr3:159261768..159308082 |
| MUC4 | Variation_91822 | chr3:195452126..195474781 |
| C5orf34 | Variation_93009 | chr5:43486813..43555021 |
| PTCD2 | Variation_93098 | chr5:71608278..71626898 |
| SCHIP1 | Variation_98504 | chr3:159240023..159241414 |
| SCHIP1 | Variation_98505 | chr3:159428123..159429500 |
| NCAPG | Variation_98637 | chr4:17811656..17813150 |
| IQCJ | Variation_103480 | chr3:158808348..158811805 |
| KATNAL2 | Variation_104348 | chr18:44541754..44557372 |
| MUC4 | Variation_104731 | chr3:195456795..195474252 |
| HEATR7A | Variation_105780 | chr8:145289437..145493482 |
| BAIAP2L2 | Variation_107169 | chr22:38504989..38508331 |
| KATNAL2 | Variation_107582 | chr18:44559112..44560266 |
| HEATR1 | Variation_107684 | chr1:236701848..236721696 |
| HARBI1 | Variation_110080 | chr11:46633028..46649957 |
| CES1 | Variation_110103 | chr16:55792555..55871702 |
| CES1 | Variation_110104 | chr16:55803275..55849656 |
| TGOLN2 | Variation_110124 | chr2:85542442..85572433 |
| AKT2 | Variation_110240 | chr19:40784738..40790835 |
| MUC4 | Variation_110772 | chr3:195438740..195725565 |
| HEATR7A | Variation_112300 | chr8:145160213..145284762 |
| HEATR7A | Variation_112301 | chr8:145171456..145218648 |
| SLC39A4 | Variation_112303 | chr8:145617232..145642015 |
| NRARP | Variation_112566 | chr9:140176583..140197712 |
| INPP5A | Variation_112873 | chr10:134579130..134621929 |
| INPP5A | Variation_112874 | chr10:134595929..134607602 |
| CELF1 | Variation_113013 | chr11:47565425..47660330 |
| FAM180B | Variation_113014 | chr11:47609306..47616463 |
| ORAOV1 | Variation_113080 | chr11:69434047..69666065 |
| TRAPPC4 | Variation_113164 | chr11:118888523..118889879 |
| AGAP2 | Variation_113352 | chr12:58118080..58123647 |
| GSK3B | Variation_0035 | chr3:119443790..119608882 |
| C12orf51 | Variation_0166 | chr12:112504265..112680932 |
| DRG1 | Variation_0236 | chr22:31797009..31965118 |
| SMPD4 | Variation_0269 | chr2:129496838..131092249 |
| CES1 | Variation_0487 | chr16:55776280..55845963 |
| SNORA70 | Variation_0674 | chrX:153605647..153628847 |
| MUC4 | Variation_0696 | chr3:195417665..195554358 |
| GGNBP2 | Variation_0800 | chr17:34756126..34930376 |
| HIC2 | Variation_0817 | chr22:21690197..21896229 |
| MUC4 | Variation_2064 | chr3:195417665..195554358 |
| SH2D4B | Variation_2160 | chr10:82166752..82315290 |
| GGNBP2 | Variation_2222 | chr17:34756126..34930376 |
| CACNG4 | Variation_2232 | chr17:64962472..65148682 |
| TBC1D16 | Variation_2234 | chr17:77744149..77931577 |
| HIC2 | Variation_2263 | chr22:21690197..21896229 |
| ZCCHC17 | Variation_2304 | chr1:31682380..32466331 |
| ZP4 | Variation_2353 | chr1:238001069..238728498 |
| SMPD4 | Variation_2408 | chr2:130774518..131484635 |
| APOD | Variation_2491 | chr3:195102787..195479748 |
| POLR3D | Variation_2746 | chr8:21673762..22802380 |
| SYCE1 | Variation_2896 | chr10:135018168..135432685 |
| C15orf58 | Variation_3094 | chr15:90623052..90809633 |
| SNRPA1 | Variation_3099 | chr15:101720143..102022334 |
| CYB5R3 | Variation_3242 | chr22:42835727..43040434 |
| ZP4 | Variation_3336 | chr1:237941794..238768102 |
| SLBP | Variation_3476 | chr4:1625392..1814775 |
| C5orf52 | Variation_3583 | chr5:156997608..157233044 |
| UBTD2 | Variation_3586 | chr5:171540423..171735272 |
| POLR3D | Variation_3726 | chr8:20533044..23111609 |
| TMEM55A | Variation_3741 | chr8:91974499..92273724 |
| CYC1 | Variation_3751 | chr8:145131184..145285644 |
| TRAF2 | Variation_3785 | chr9:139250829..139826485 |
| STOX1 | Variation_3808 | chr10:70457719..70631312 |
| FANK1 | Variation_3825 | chr10:127453900..127786702 |
| C10orf125 | Variation_3829 | chr10:134844422..135222302 |
| C11orf68 | Variation_3855 | chr11:65559893..65722363 |
| SERINC4 | Variation_3960 | chr15:43823158..44148641 |
| SNRPA1 | Variation_3983 | chr15:101732816..101916486 |
| SPIRE2 | Variation_4019 | chr16:89870732..89967949 |
| TTC9B | Variation_4080 | chr19:40612420..40839520 |
| DHX34 | Variation_4083 | chr19:47857756..48025505 |
| CYB5R3 | Variation_4129 | chr22:42767301..43083306 |
| GLMN | Variation_4234 | chr1:92669282..92805629 |
| MUC4 | Variation_4366 | chr3:195419364..195589697 |
| LACE1 | Variation_4505 | chr6:108487814..108663706 |
| EPB49 | Variation_4586 | chr8:21888599..22041926 |
| CA1 | Variation_4601 | chr8:86263108..86444141 |
| INPP5A | Variation_4719 | chr10:134575045..134753830 |
| GPR123 | Variation_4721 | chr10:134903013..135181736 |
| KNDC1 | Variation_4722 | chr10:134957100..135134251 |
| ECHS1 | Variation_4724 | chr10:135115064..135226145 |
| AGBL2 | Variation_4741 | chr11:47517955..47681310 |
| YPEL4 | Variation_4744 | chr11:57299354..57481050 |
| NRXN2 | Variation_4753 | chr11:64487183..64620199 |
| KBTBD7 | Variation_4799 | chr13:41604931..41798808 |
| TNFAIP2 | Variation_4848 | chr14:103436947..103609181 |
| RCN2 | Variation_4905 | chr15:77235896..77393640 |
| STAC2 | Variation_5006 | chr17:37320807..37476796 |
| DHX8 | Variation_5007 | chr17:41508997..41689929 |
| C17orf104 | Variation_5009 | chr17:42717156..42903241 |
| MYCBPAP | Variation_5017 | chr17:48594242..48755830 |
| PRPSAP1 | Variation_5032 | chr17:74204580..74395308 |
| PTGER1 | Variation_5090 | chr19:14492599..14614873 |
| DGCR8 | Variation_5168 | chr22:19887966..20069970 |
| CNGB3 | Variation_5257 | chr8:87547393..88826926 |
| C10orf125 | Variation_5269 | chr10:135128234..135264045 |
| C10orf125 | Variation_5270 | chr10:135085168..135264045 |
| C10orf125 | Variation_5271 | chr10:135079801..135264045 |
| ALKBH2 | Variation_5312 | chr12:109186454..109556165 |
| CRYBB3 | Variation_5357 | chr22:25512251..25799870 |
| NRXN2 | Variation_5422 | chr11:64312491..64483418 |
| EIF4EBP2 | Variation_6879 | chr10:71263986..72546603 |
| ADAMTS7 | Variation_7073 | chr15:79053484..79293881 |
| DGKE | Variation_7166 | chr17:54760895..55079493 |
| CD86 | Variation_7357 | chr3:121500400..122091831 |
| MUC4 | Variation_7399 | chr3:195419197..195477831 |
| LMLN | Variation_7403 | chr3:197711297..197895198 |
| EGFL7 | Variation_7732 | chr9:139561016..139568542 |
| ZP4 | Variation_8350 | chr1:238012770..238725959 |
| ABI3BP | Variation_8426 | chr3:100342493..100493062 |
| ABI3BP | Variation_8427 | chr3:100299310..100479384 |
| RPL35A | Variation_8440 | chr3:197475440..197833758 |
| ECHS1 | Variation_8671 | chr10:135184119..135434551 |
| ECHS1 | Variation_8673 | chr10:135079862..135434551 |
| PRRG4 | Variation_8685 | chr11:32796092..33004024 |
| CPEB1 | Variation_9266 | chr15:83283555..83453495 |
| KIAA1984 | Variation_9631 | chr9:139634495..139904037 |
| SEMA4B | Variation_9752 | chr15:90647856..90784930 |
| CPEB1 | Variation_10488 | chr15:83299461..83457424 |
| KIAA1875 | Variation_23290 | chr8:145123429..145227100 |
| TYK2 | Variation_29686 | chr19:10435493..10472933 |
| GPRIN3 | Variation_29744 | chr4:89644931..90643144 |
| ZNF511 | Variation_29877 | chr10:135085754..135157955 |
| KNDC1 | Variation_29879 | chr10:135000159..135086380 |
| AATK | Variation_30032 | chr17:79012346..79264160 |
| TMEM129 | Variation_30192 | chr4:1563920..1724579 |
| CYHR1 | Variation_30309 | chr8:145641564..145690308 |
| EGFL7 | Variation_30348 | chr9:139499710..139570617 |
| EGFL7 | Variation_30352 | chr9:139483954..139583155 |
| EGFL7 | Variation_30358 | chr9:139534703..139590933 |
| ARID5B | Variation_30508 | chr10:63712167..64312757 |
| UTF1 | Variation_30525 | chr10:135035825..135068932 |
| FBXL6 | Variation_31457 | chr8:145293531..145584222 |
| MAMDC4 | Variation_31536 | chr9:139740369..139755472 |
| ECHS1 | Variation_34537 | chr10:135184421..135434551 |
| CRYBB3 | Variation_34657 | chr22:25593658..25931372 |
| CATSPER2 | Variation_35314 | chr15:43878745..43972533 |
| SRP68 | Variation_35500 | chr17:74066266..74083883 |
| KATNAL2 | Variation_35550 | chr18:44547638..44590333 |
| KATNAL2 | Variation_35564 | chr18:44553905..44566471 |
| KATNAL2 | Variation_35565 | chr18:44554482..44570031 |
| KATNAL2 | Variation_35566 | chr18:44554658..44569726 |
| KATNAL2 | Variation_35567 | chr18:44554911..44562289 |
| AATF | Variation_37189 | chr17:34814328..36297053 |
| ARID5B | Variation_47941 | chr10:63780945..64187564 |
| C2orf29 | Variation_47994 | chr2:101624916..102008313 |
| GNAZ | Variation_48008 | chr22:23216762..23499513 |
| DDX50 | Variation_48614 | chr10:70691708..70775081 |
| C11orf94 | Variation_48807 | chr11:45903194..45939641 |
| ZNF740 | Variation_49095 | chr12:53493387..53614993 |
| ZNF740 | Variation_49096 | chr12:53493387..53621711 |
| STARD3 | Variation_49853 | chr17:37781849..37831035 |
| NPEPPS | Variation_49876 | chr17:45419139..45702280 |
| YDJC | Variation_50817 | chr22:21982892..21997070 |
| TBX10 | Variation_53128 | chr11:67372477..67433869 |
| VPS28 | Variation_53129 | chr8:145641564..145750506 |
| MAP4K2 | Variation_53146 | chr11:64477050..64584959 |
| EGFL7 | Variation_53532 | chr9:139477530..139570617 |
| ANKRD9 | Variation_53624 | chr14:102933347..103070845 |
| ZNF511 | Variation_53744 | chr10:135071004..135146714 |
| CD8B | Variation_68073 | chr2:86972214..87174121 |
| MUC4 | Variation_68545 | chr3:195417695..195477757 |
| KIAA1267 | Variation_72954 | chr17:44165296..44434235 |
| NPTX1 | Variation_73056 | chr17:78370328..78630657 |
| SEMA4B | Variation_77067 | chr15:90723651..90786590 |
| SAMD14 | Variation_77656 | chr17:48174804..48215956 |
| KATNAL2 | Variation_77873 | chr18:44541825..44559024 |
| MUC4 | Variation_80084 | chr3:195421663..195474781 |
| WDR27 | Variation_81775 | chr6:170098140..170129460 |
| PATL1 | Variation_85808 | chr11:59311461..59475710 |
| ARHGAP27 | Variation_88487 | chr17:43489560..43519239 |
| KATNAL2 | Variation_88828 | chr18:44541825..44559024 |
| MUC4 | Variation_91819 | chr3:195416034..195474781 |
| WDR27 | Variation_94258 | chr6:170084046..170160780 |
| TMEM129 | Variation_110794 | chr4:1494917..1897303 |
| NAT8L | Variation_110798 | chr4:1992967..2082167 |
| MAF1 | Variation_112300 | chr8:145160213..145284762 |
| CPSF1 | Variation_112303 | chr8:145617232..145642015 |
| C9orf86 | Variation_112559 | chr9:139700812..139718065 |
| KIAA0913 | Variation_112735 | chr10:75475447..75682321 |
| C11orf24 | Variation_113072 | chr11:67763676..68197054 |
| FGF4 | Variation_113080 | chr11:69434047..69666065 |
| FGF19 | Variation_113081 | chr11:69444561..69524415 |
| RNF26 | Variation_113167 | chr11:119193027..119238670 |
| ANKRD33 | Variation_113343 | chr12:52201114..52282359 |
| RPL35A | Variation_0044 | chr3:197673380..197873380 |
| SLC12A6 | Variation_0187 | chr15:34610451..34671482 |
| SNORA76 | Variation_0210 | chr17:62118842..62273472 |
| DLG1 | Variation_0568 | chr3:195433152..197378091 |
| HOMER2 | Variation_0789 | chr15:83417765..83560629 |
| KIAA1267 | Variation_0802 | chr17:44216748..44378253 |
| KIAA1267 | Variation_2227 | chr17:44216748..44378253 |
| CRYBB2 | Variation_2270 | chr22:25598912..25762849 |
| SLC39A14 | Variation_2746 | chr8:21673762..22802380 |
| DMXL2 | Variation_3081 | chr15:51352249..51783981 |
| DNAJB8 | Variation_3447 | chr3:128057693..128437817 |
| ARMC8 | Variation_3449 | chr3:137611789..137933835 |
| CCDC50 | Variation_3469 | chr3:190936834..191279107 |
| MFSD7 | Variation_3475 | chr4:603208..931354 |
| SLC39A14 | Variation_3726 | chr8:20533044..23111609 |
| GPR123 | Variation_3829 | chr10:134844422..135222302 |
| FIBP | Variation_3855 | chr11:65559893..65722363 |
| PPP1R13B | Variation_3949 | chr14:104147525..104433135 |
| STOML1 | Variation_3968 | chr15:74215114..74464916 |
| RCN2 | Variation_3969 | chr15:77170550..77373448 |
| SEMA4B | Variation_3976 | chr15:90565956..90789225 |
| EIF1 | Variation_4036 | chr17:39301375..39869610 |
| CRYBB3 | Variation_4120 | chr22:25366801..25956907 |
| CRYBA4 | Variation_4121 | chr22:26898499..27029896 |
| CDCP1 | Variation_4333 | chr3:44970199..45190848 |
| PSTK | Variation_4714 | chr10:124600607..124797901 |
| ECHS1 | Variation_4721 | chr10:134903013..135181736 |
| SF1 | Variation_4753 | chr11:64487183..64620199 |
| FGF19 | Variation_4755 | chr11:69423683..69588478 |
| KBTBD6 | Variation_4799 | chr13:41604931..41798808 |
| MAN2A2 | Variation_4909 | chr15:91454426..91641749 |
| EXOSC6 | Variation_4964 | chr16:70185694..70353334 |
| SF3B3 | Variation_4965 | chr16:70369770..70569723 |
| EPN3 | Variation_5017 | chr17:48594242..48755830 |
| RNF157 | Variation_5032 | chr17:74204580..74395308 |
| KATNAL2 | Variation_5054 | chr18:44406424..44554153 |
| PRKD2 | Variation_5104 | chr19:47206549..47370304 |
| HIC2 | Variation_5173 | chr22:21775069..21950089 |
| BAIAP2L2 | Variation_5185 | chr22:38388465..38549017 |
| PLA2G6 | Variation_5186 | chr22:38459503..38636510 |
| ECHS1 | Variation_5269 | chr10:135128234..135264045 |
| ECHS1 | Variation_5270 | chr10:135085168..135264045 |
| ECHS1 | Variation_5271 | chr10:135079801..135264045 |
| C10orf125 | Variation_5272 | chr10:135003417..135264045 |
| C10orf125 | Variation_5273 | chr10:135003417..135264045 |
| CDK2AP2 | Variation_5275 | chr11:67234322..67626901 |
| SVOP | Variation_5312 | chr12:109186454..109556165 |
| TMEM41B | Variation_6953 | chr11:9007612..9311131 |
| CATSPER2 | Variation_7061 | chr15:43888955..43995223 |
| COIL | Variation_7166 | chr17:54760895..55079493 |
| TSPO | Variation_7352 | chr22:43509764..43722128 |
| IQCB1 | Variation_7357 | chr3:121500400..122091831 |
| LRCH3 | Variation_8440 | chr3:197475440..197833758 |
| SEMA4B | Variation_8803 | chr15:90622504..90792506 |
| SNRPA1 | Variation_8807 | chr15:101517477..102022756 |
| NRXN2 | Variation_9667 | chr11:64345448..64546391 |
| HEATR7A | Variation_23290 | chr8:145123429..145227100 |
| MAMDC4 | Variation_23294 | chr9:139690144..139754110 |
| RAVER1 | Variation_29685 | chr19:10399904..10471977 |
| FAM13A | Variation_29744 | chr4:89644931..90643144 |
| UTF1 | Variation_29879 | chr10:135000159..135086380 |
| KIAA1984 | Variation_30359 | chr9:139534703..139823670 |
| KATNAL2 | Variation_30870 | chr18:44495994..44635156 |
| HEATR7A | Variation_31457 | chr8:145293531..145584222 |
| KATNAL2 | Variation_32213 | chr18:44495994..44635156 |
| APOD | Variation_32549 | chr3:195286212..195477752 |
| PCSK6 | Variation_34506 | chr15:101991277..102393237 |
| PPIP5K1 | Variation_35314 | chr15:43878745..43972533 |
| CATSPER2 | Variation_35315 | chr15:43878812..43996580 |
| IPO4 | Variation_37155 | chr14:24627509..24651331 |
| SEMA4B | Variation_37691 | chr15:90632610..90778393 |
| ZRANB3 | Variation_47933 | chr2:136049982..136537952 |
| POU6F1 | Variation_47982 | chr12:51326702..51671911 |
| HEATR1 | Variation_48281 | chr1:236602323..236781939 |
| HEATR1 | Variation_48282 | chr1:236602323..236787789 |
| PHF21A | Variation_48808 | chr11:45903194..45975130 |
| RCCD1 | Variation_49620 | chr15:91411656..91525197 |
| TCAP | Variation_49854 | chr17:37814080..37831035 |
| TCAP | Variation_49855 | chr17:37817482..37831035 |
| CYHR1 | Variation_53129 | chr8:145641564..145750506 |
| RASGRP2 | Variation_53146 | chr11:64477050..64584959 |
| HEATR1 | Variation_53259 | chr1:236604211..236781939 |
| CATSPER2 | Variation_66780 | chr15:43852144..43988641 |
| CATSPER2 | Variation_66782 | chr15:43852144..43988641 |
| MUC4 | Variation_68544 | chr3:195379517..195477817 |
| ART5 | Variation_71243 | chr11:3426081..3674989 |
| SAMD4B | Variation_73367 | chr19:39811570..39886212 |
| SEMA4B | Variation_77066 | chr15:90623760..90786590 |
| MUC4 | Variation_80082 | chr3:195341108..195474781 |
| MUC4 | Variation_80085 | chr3:195387894..195474781 |
| CATSPER2 | Variation_87664 | chr15:43833982..44039630 |
| MUC4 | Variation_91816 | chr3:195341108..195474781 |
| TCTE3 | Variation_94258 | chr6:170084046..170160780 |
| CATSPER2 | Variation_104079 | chr15:43885118..43988553 |
| MUC4 | Variation_110770 | chr3:195341611..195487543 |
| CHKA | Variation_113072 | chr11:67763676..68197054 |
| AGAP2 | Variation_113354 | chr12:58124758..58157850 |
| CPEB1 | Variation_0190 | chr15:83116859..83285841 |
| OR5AS1 | Variation_0308 | chr11:55153930..55816828 |
| SLC12A6 | Variation_0783 | chr15:34610451..34671482 |
| C10orf125 | Variation_2896 | chr10:135018168..135432685 |
| CATSPER2 | Variation_3079 | chr15:43831923..44058634 |
| SEMA4B | Variation_3094 | chr15:90623052..90809633 |
| GGNBP2 | Variation_3142 | chr17:34405314..34942750 |
| CRYBB2 | Variation_3239 | chr22:25608162..26046513 |
| FAM178B | Variation_3378 | chr2:97571109..98336507 |
| SMPD4 | Variation_3393 | chr2:130346838..133198003 |
| KNG1 | Variation_3467 | chr3:186337134..186567326 |
| DOK7 | Variation_3477 | chr4:3394344..4350986 |
| ANKRD37 | Variation_3530 | chr4:186313125..186474696 |
| KIAA1875 | Variation_3751 | chr8:145131184..145285644 |
| ECHS1 | Variation_3829 | chr10:134844422..135222302 |
| MPEG1 | Variation_3853 | chr11:58700092..59008673 |
| TDRD9 | Variation_3949 | chr14:104147525..104433135 |
| PPIP5K1 | Variation_3960 | chr15:43823158..44148641 |
| DMXL2 | Variation_3963 | chr15:51282257..51805426 |
| CES1 | Variation_4007 | chr16:55669396..55983590 |
| EXOSC6 | Variation_4009 | chr16:69855122..70313222 |
| AATK | Variation_4048 | chr17:79126431..79319162 |
| DGCR8 | Variation_4117 | chr22:20026493..20309447 |
| LPO | Variation_4410 | chr17:56312860..56498404 |
| ZNF511 | Variation_4722 | chr10:134957100..135134251 |
| UTF1 | Variation_4723 | chr10:134975213..135081168 |
| CACNB1 | Variation_5006 | chr17:37320807..37476796 |
| UBTF | Variation_5008 | chr17:42185921..42374668 |
| KATNAL2 | Variation_5055 | chr18:44546354..44714872 |
| IL27RA | Variation_5089 | chr19:14130013..14288003 |
| AIFM3 | Variation_5170 | chr22:21334133..21524524 |
| POLR3H | Variation_5189 | chr22:41759617..41950032 |
| ECHS1 | Variation_5272 | chr10:135003417..135264045 |
| ECHS1 | Variation_5273 | chr10:135003417..135264045 |
| SSH1 | Variation_5312 | chr12:109186454..109556165 |
| KIAA1274 | Variation_6879 | chr10:71263986..72546603 |
| HEATR5A | Variation_7031 | chr14:30800223..31911745 |
| TRIM25 | Variation_7166 | chr17:54760895..55079493 |
| SCUBE1 | Variation_7352 | chr22:43509764..43722128 |
| ZNF692 | Variation_8355 | chr1:248522680..249158239 |
| REEP1 | Variation_8380 | chr2:86253542..86505453 |
| SMPD4 | Variation_8387 | chr2:130363731..131143136 |
| OR5AS1 | Variation_8690 | chr11:54835623..55879426 |
| EIF2S1 | Variation_8772 | chr14:67364747..67886781 |
| DMXL2 | Variation_8798 | chr15:51276128..51752856 |
| PCSK6 | Variation_8807 | chr15:101517477..102022756 |
| TM2D3 | Variation_8808 | chr15:102036377..102393237 |
| AP3B2 | Variation_9266 | chr15:83283555..83453495 |
| ARHGAP27 | Variation_9287 | chr17:43470760..43666906 |
| SF1 | Variation_9667 | chr11:64345448..64546391 |
| AP3B2 | Variation_10488 | chr15:83299461..83457424 |
| MAF1 | Variation_23290 | chr8:145123429..145227100 |
| DEAF1 | Variation_29884 | chr11:549119..710558 |
| AZI1 | Variation_30032 | chr17:79012346..79264160 |
| KIAA1875 | Variation_30304 | chr8:144992102..145175528 |
| KIAA1984 | Variation_30350 | chr9:139254317..139950351 |
| DGCR8 | Variation_31071 | chr22:19019088..21053198 |
| MUC4 | Variation_32549 | chr3:195286212..195477752 |
| ARHGAP27 | Variation_34654 | chr17:43493101..43666906 |
| PPIP5K1 | Variation_35315 | chr15:43878812..43996580 |
| SMAGP | Variation_47982 | chr12:51326702..51671911 |
| CREG2 | Variation_47994 | chr2:101624916..102008313 |
| C11orf94 | Variation_48808 | chr11:45903194..45975130 |
| VPS37B | Variation_48972 | chr12:123335527..123595163 |
| VPS37B | Variation_48973 | chr12:123335527..123626982 |
| ZNF740 | Variation_49093 | chr12:53449321..53614993 |
| ZNF740 | Variation_49094 | chr12:53449321..53621711 |
| UNC45A | Variation_49620 | chr15:91411656..91525197 |
| TCAP | Variation_49853 | chr17:37781849..37831035 |
| NRXN2 | Variation_53146 | chr11:64477050..64584959 |
| EGFL7 | Variation_53172 | chr9:139513538..139620311 |
| PPIP5K1 | Variation_66780 | chr15:43852144..43988641 |
| PPIP5K1 | Variation_66782 | chr15:43852144..43988641 |
| CCNB1IP1 | Variation_71865 | chr14:20800765..21257878 |
| CPEB1 | Variation_72323 | chr15:83096440..83219640 |
| TBCD | Variation_73078 | chr17:79785999..81060000 |
| DGKZ | Variation_113007 | chr11:46303184..46425613 |
| PTPMT1 | Variation_113013 | chr11:47565425..47660330 |
| FGF19 | Variation_113080 | chr11:69434047..69666065 |
| SLC26A11 | Variation_2235 | chr17:78059123..78267285 |
| TRIM58 | Variation_2358 | chr1:247753334..248322316 |
| MUC4 | Variation_2491 | chr3:195102787..195479748 |
| TRIM58 | Variation_3339 | chr1:247714585..248277146 |
| RPN1 | Variation_3447 | chr3:128057693..128437817 |
| SNORA81 | Variation_3467 | chr3:186337134..186567326 |
| CPSF1 | Variation_3752 | chr8:145523693..145697678 |
| TRIM21 | Variation_3834 | chr11:4055945..4510188 |
| EFEMP2 | Variation_3855 | chr11:65559893..65722363 |
| XRCC3 | Variation_3949 | chr14:104147525..104433135 |
| CRYBB2 | Variation_4120 | chr22:25366801..25956907 |
| DNASE2 | Variation_4400 | chr19:12847630..13058491 |
| RASGRP2 | Variation_4753 | chr11:64487183..64620199 |
| UNC45A | Variation_4909 | chr15:91454426..91641749 |
| NANOS3 | Variation_5088 | chr19:13836718..14062800 |
| CD97 | Variation_5090 | chr19:14492599..14614873 |
| BAIAP2L2 | Variation_5186 | chr22:38459503..38636510 |
| HECTD1 | Variation_7031 | chr14:30800223..31911745 |
| KIAA1267 | Variation_7162 | chr17:44162433..44694253 |
| DPCD | Variation_8666 | chr10:103054982..103452645 |
| KIAA1267 | Variation_8850 | chr17:44004972..44811116 |
| CYC1 | Variation_23290 | chr8:145123429..145227100 |
| TYK2 | Variation_29685 | chr19:10399904..10471977 |
| KIAA1875 | Variation_30305 | chr8:144992102..145513752 |
| SLC39A4 | Variation_30306 | chr8:144992102..145744618 |
| SNORA17 | Variation_30351 | chr9:139395473..139622024 |
| KIAA1267 | Variation_30837 | chr17:44165754..44780028 |
| TM2D3 | Variation_34506 | chr15:101991277..102393237 |
| SNORA70 | Variation_37365 | chrX:153577679..153652031 |
| ZNF740 | Variation_49089 | chr12:53431010..53614993 |
| ZNF740 | Variation_49090 | chr12:53431010..53621711 |
| ZNF740 | Variation_49091 | chr12:53437806..53614993 |
| ZNF740 | Variation_49092 | chr12:53437806..53621711 |
| MAN2A2 | Variation_49620 | chr15:91411656..91525197 |
| KIAA1267 | Variation_49870 | chr17:44169808..44788310 |
| KIAA1267 | Variation_49871 | chr17:44169808..44790203 |
| KIAA1267 | Variation_49872 | chr17:44169808..44793283 |
| NUDT8 | Variation_53128 | chr11:67372477..67433869 |
| SF1 | Variation_53146 | chr11:64477050..64584959 |
| KIAA1875 | Variation_53614 | chr8:144927272..145216223 |
| FAM160B2 | Variation_53638 | chr8:21952226..22021037 |
| KIAA1267 | Variation_72953 | chr17:44165296..44784771 |
| PPIP5K1 | Variation_87664 | chr15:43833982..44039630 |
| OBFC2B | Variation_0164 | chr12:56553078..56902907 |
| KIAA1267 | Variation_0327 | chr17:44159555..44770359 |
| DOK2 | Variation_2746 | chr8:21673762..22802380 |
| ECHS1 | Variation_2896 | chr10:135018168..135432685 |
| ARHGAP27 | Variation_3146 | chr17:43457886..43733983 |
| KIAA1267 | Variation_3147 | chr17:44162284..44868187 |
| EEFSEC | Variation_3447 | chr3:128057693..128437817 |
| SNORA4 | Variation_3467 | chr3:186337134..186567326 |
| TFRC | Variation_3472 | chr3:195379483..196071593 |
| HEATR7A | Variation_3751 | chr8:145131184..145285644 |
| ART5 | Variation_3833 | chr11:3236512..3801429 |
| MFAP1 | Variation_3960 | chr15:43823158..44148641 |
| CDC27 | Variation_4038 | chr17:44083897..45277333 |
| AZI1 | Variation_4048 | chr17:79126431..79319162 |
| LEUTX | Variation_4079 | chr19:40227904..40521130 |
| TEX101 | Variation_4082 | chr19:43042747..43899053 |
| ZDHHC8 | Variation_4117 | chr22:20026493..20309447 |
| CPSF1 | Variation_4613 | chr8:145565803..145769410 |
| KNDC1 | Variation_4721 | chr10:134903013..135181736 |
| UTF1 | Variation_4722 | chr10:134957100..135134251 |
| ZNF511 | Variation_4724 | chr10:135115064..135226145 |
| FAM180B | Variation_4741 | chr11:47517955..47681310 |
| CDC42BPG | Variation_4753 | chr11:64487183..64620199 |
| TMUB2 | Variation_5008 | chr17:42185921..42374668 |
| PITPNM1 | Variation_5275 | chr11:67234322..67626901 |
| CD300LB | Variation_5336 | chr17:71671182..72561678 |
| TSPAN15 | Variation_6879 | chr10:71263986..72546603 |
| AVEN | Variation_7058 | chr15:31982066..34867298 |
| LMLN | Variation_8440 | chr3:197475440..197833758 |
| MRM1 | Variation_8843 | chr17:34435487..35081387 |
| FOXH1 | Variation_23292 | chr8:145682115..145750506 |
| C9orf86 | Variation_23294 | chr9:139690144..139754110 |
| KIAA0284 | Variation_29962 | chr14:105154105..105348552 |
| FAM160B2 | Variation_30283 | chr8:21952226..22028668 |
| MAF1 | Variation_30304 | chr8:144992102..145175528 |
| VPS28 | Variation_30306 | chr8:144992102..145744618 |
| SNORA17 | Variation_30357 | chr9:139513538..139642960 |
| C22orf13 | Variation_31081 | chr22:24861624..25052702 |
| KIAA1267 | Variation_34570 | chr17:44104510..44941033 |
| PLCD3 | Variation_34646 | chr17:43183028..43693538 |
| MPST | Variation_47855 | chr22:37362090..37585844 |
| KNG1 | Variation_47856 | chr3:186414884..186754730 |
| CHMP6 | Variation_73071 | chr17:78632841..79703517 |
| CHCHD1 | Variation_112735 | chr10:75475447..75682321 |
| FAM180B | Variation_113013 | chr11:47565425..47660330 |
| SUV420H1 | Variation_113072 | chr11:67763676..68197054 |
| ZDHHC19 | Variation_3472 | chr3:195379483..196071593 |
| MAF1 | Variation_3751 | chr8:145131184..145285644 |
| KIAA1984 | Variation_3785 | chr9:139250829..139826485 |
| SLC12A6 | Variation_3957 | chr15:34543021..34988247 |
| CPEB1 | Variation_3972 | chr15:82879646..83310525 |
| FAM160B2 | Variation_4586 | chr8:21888599..22041926 |
| LZTR1 | Variation_5170 | chr22:21334133..21524524 |
| KIAA1984 | Variation_5415 | chr9:139478587..140244550 |
| SLC12A6 | Variation_7058 | chr15:31982066..34867298 |
| CD300LF | Variation_7171 | chr17:72257192..72728232 |
| SH3BP5L | Variation_8355 | chr1:248522680..249158239 |
| MAMDC4 | Variation_9631 | chr9:139634495..139904037 |
| ZNF511 | Variation_29878 | chr10:135053021..135223018 |
| HEATR7A | Variation_30305 | chr8:144992102..145513752 |
| EGFL7 | Variation_30351 | chr9:139395473..139622024 |
| ARHGAP27 | Variation_34629 | chr17:43338941..43693538 |
| SNORA81 | Variation_47856 | chr3:186414884..186754730 |
| POLR3H | Variation_50867 | chr22:41651034..41961831 |
| HEATR7A | Variation_53614 | chr8:144927272..145216223 |
| SYT6 | Variation_64644 | chr1:113862952..114901117 |
| SEC24C | Variation_112735 | chr10:75475447..75682321 |
| COQ10A | Variation_0164 | chr12:56553078..56902907 |
| SORBS3 | Variation_2746 | chr8:21673762..22802380 |
| C1orf150 | Variation_3339 | chr1:247714585..248277146 |
| DOK2 | Variation_3726 | chr8:20533044..23111609 |
| KNDC1 | Variation_3829 | chr10:134844422..135222302 |
| CATSPER2 | Variation_3960 | chr15:43823158..44148641 |
| GGNBP2 | Variation_4031 | chr17:34285285..34907282 |
| ZNF511 | Variation_4721 | chr10:134903013..135181736 |
| CC2D1A | Variation_5088 | chr19:13836718..14062800 |
| ZNF511 | Variation_5270 | chr10:135085168..135264045 |
| ZNF511 | Variation_5271 | chr10:135079801..135264045 |
| KNDC1 | Variation_5272 | chr10:135003417..135264045 |
| KNDC1 | Variation_5273 | chr10:135003417..135264045 |
| TMEM134 | Variation_5275 | chr11:67234322..67626901 |
| CD300LB | Variation_7171 | chr17:72257192..72728232 |
| CD300LF | Variation_8852 | chr17:71834001..72712362 |
| CYC1 | Variation_30304 | chr8:144992102..145175528 |
| MAF1 | Variation_30305 | chr8:144992102..145513752 |
| CYHR1 | Variation_30306 | chr8:144992102..145744618 |
| EGFL7 | Variation_30357 | chr9:139513538..139642960 |
| MAMDC4 | Variation_30359 | chr9:139534703..139823670 |
| PDDC1 | Variation_30530 | chr11:776338..834514 |
| CD300LF | Variation_34522 | chr17:71825523..72723445 |
| C17orf46 | Variation_34629 | chr17:43338941..43693538 |
| POLR3H | Variation_50866 | chr22:41648502..41981957 |
| FOXH1 | Variation_53129 | chr8:145641564..145750506 |
| MAF1 | Variation_53614 | chr8:144927272..145216223 |
| AATK | Variation_73071 | chr17:78632841..79703517 |
| MFSD7 | Variation_110784 | chr4:540105..1413799 |
| EPB49 | Variation_2746 | chr8:21673762..22802380 |
| RANBP1 | Variation_4117 | chr22:20026493..20309447 |
| NUDT8 | Variation_5275 | chr11:67234322..67626901 |
| ZNF692 | Variation_8354 | chr1:248060959..249167690 |
| ZNF511 | Variation_8673 | chr10:135079862..135434551 |
| CD300LB | Variation_8852 | chr17:71834001..72712362 |
| C10orf125 | Variation_29878 | chr10:135053021..135223018 |
| KLHL22 | Variation_31071 | chr22:19019088..21053198 |
| SNORA17 | Variation_31535 | chr9:139401206..139654107 |
| CD300LB | Variation_34522 | chr17:71825523..72723445 |
| ARHGAP27 | Variation_34646 | chr17:43183028..43693538 |
| MRM1 | Variation_37189 | chr17:34814328..36297053 |
| CD300LB | Variation_53066 | chr17:71822155..72681941 |
| KLHL33 | Variation_71865 | chr14:20800765..21257878 |
| SNRNP40 | Variation_3287 | chr1:31722140..32356850 |
| FETUB | Variation_3467 | chr3:186337134..186567326 |
| PCYT1A | Variation_3472 | chr3:195379483..196071593 |
| FBXL6 | Variation_3752 | chr8:145523693..145697678 |
| ZNF511 | Variation_3829 | chr10:134844422..135222302 |
| KIAA1267 | Variation_4038 | chr17:44083897..45277333 |
| SYCE2 | Variation_4400 | chr19:12847630..13058491 |
| UTF1 | Variation_4721 | chr10:134903013..135181736 |
| C19orf53 | Variation_5088 | chr19:13836718..14062800 |
| ZNF511 | Variation_5272 | chr10:135003417..135264045 |
| ZNF511 | Variation_5273 | chr10:135003417..135264045 |
| ADAMTS14 | Variation_6879 | chr10:71263986..72546603 |
| C10orf125 | Variation_8673 | chr10:135079862..135434551 |
| SCARF2 | Variation_31071 | chr22:19019088..21053198 |
| C17orf46 | Variation_34646 | chr17:43183028..43693538 |
| CYC1 | Variation_53614 | chr8:144927272..145216223 |
| TFRC | Variation_0568 | chr3:195433152..197378091 |
| SNRNP40 | Variation_2304 | chr1:31682380..32466331 |
| KNDC1 | Variation_2896 | chr10:135018168..135432685 |
| MUC4 | Variation_3472 | chr3:195379483..196071593 |
| SORBS3 | Variation_3726 | chr8:20533044..23111609 |
| SNORA17 | Variation_3785 | chr9:139250829..139826485 |
| ELL3 | Variation_3960 | chr15:43823158..44148641 |
| CCDC130 | Variation_5088 | chr19:13836718..14062800 |
| CYC1 | Variation_30305 | chr8:144992102..145513752 |
| KIAA1875 | Variation_30306 | chr8:144992102..145744618 |
| EGFL7 | Variation_31535 | chr9:139401206..139654107 |
| KIAA1267 | Variation_37194 | chr17:43544138..44633937 |
| TRIM58 | Variation_48302 | chr1:247849473..248277638 |
| TEP1 | Variation_71865 | chr14:20800765..21257878 |
| SPRYD4 | Variation_0164 | chr12:56553078..56902907 |
| ZDHHC19 | Variation_0568 | chr3:195433152..197378091 |
| SPOCD1 | Variation_3287 | chr1:31722140..32356850 |
| EPB49 | Variation_3726 | chr8:20533044..23111609 |
| UTF1 | Variation_3829 | chr10:134844422..135222302 |
| ASNA1 | Variation_4400 | chr19:12847630..13058491 |
| FBXL6 | Variation_4613 | chr8:145565803..145769410 |
| UTF1 | Variation_5272 | chr10:135003417..135264045 |
| UTF1 | Variation_5273 | chr10:135003417..135264045 |
| TBX10 | Variation_5275 | chr11:67234322..67626901 |
| DEAF1 | Variation_29883 | chr11:378188..870446 |
| UBQLNL | Variation_34458 | chr11:5505764..6194475 |
| RPP25 | Variation_37170 | chr15:74364360..75569130 |
| TFRC | Variation_37243 | chr3:195397785..197386290 |
| TRIM58 | Variation_48303 | chr1:247849473..248285441 |
| AZI1 | Variation_73071 | chr17:78632841..79703517 |
| SNORD22 | Variation_0150 | chr11:62514284..62691489 |
| SPOCD1 | Variation_2304 | chr1:31682380..32466331 |
| PDLIM2 | Variation_2746 | chr8:21673762..22802380 |
| SLC39A4 | Variation_3752 | chr8:145523693..145697678 |
| ZNF511 | Variation_2896 | chr10:135018168..135432685 |
| VPS28 | Variation_3752 | chr8:145523693..145697678 |
| FBXL6 | Variation_30306 | chr8:144992102..145744618 |
| ZDHHC8 | Variation_31071 | chr22:19019088..21053198 |
| SCAMP2 | Variation_37170 | chr15:74364360..75569130 |
| METRNL | Variation_73078 | chr17:79785999..81060000 |
| MYO15A | Variation_0495 | chr17:16717959..18322297 |
| SH3BP5L | Variation_8354 | chr1:248060959..249167690 |
| HEATR7A | Variation_30306 | chr8:144992102..145744618 |
| SNORA17 | Variation_30359 | chr9:139534703..139823670 |
| CDC45 | Variation_31071 | chr22:19019088..21053198 |
| MFI2 | Variation_37243 | chr3:195397785..197386290 |
| CNPY2 | Variation_0164 | chr12:56553078..56902907 |
| UTF1 | Variation_2896 | chr10:135018168..135432685 |
| PDLIM2 | Variation_3726 | chr8:20533044..23111609 |
| C9orf86 | Variation_3785 | chr9:139250829..139826485 |
| FOXH1 | Variation_4613 | chr8:145565803..145769410 |
| TRIM58 | Variation_8353 | chr1:247753207..248376070 |
| MAF1 | Variation_30306 | chr8:144992102..145744618 |
| MAMDC4 | Variation_30350 | chr9:139254317..139950351 |
| STRA6 | Variation_37170 | chr15:74364360..75569130 |
| FN3KRP | Variation_73078 | chr17:79785999..81060000 |
| MFI2 | Variation_0568 | chr3:195433152..197378091 |
| SNORA17 | Variation_30355 | chr9:139254317..139656516 |
| C9orf86 | Variation_30359 | chr9:139534703..139823670 |
| MUC4 | Variation_37243 | chr3:195397785..197386290 |
| OSGEP | Variation_71865 | chr14:20800765..21257878 |
| SYT6 | Variation_2322 | chr1:113246630..116699268 |
| EGFL7 | Variation_30359 | chr9:139534703..139823670 |
| ULK3 | Variation_37170 | chr15:74364360..75569130 |
| SYT6 | Variation_3306 | chr1:113157135..116741372 |
| SLC39A4 | Variation_4613 | chr8:145565803..145769410 |
| MAMDC4 | Variation_5415 | chr9:139478587..140244550 |
| C9orf86 | Variation_9631 | chr9:139634495..139904037 |
| CYC1 | Variation_30306 | chr8:144992102..145744618 |
| EGFL7 | Variation_30355 | chr9:139254317..139656516 |
| PCYT1A | Variation_0568 | chr3:195433152..197378091 |
| FAM160B2 | Variation_2746 | chr8:21673762..22802380 |
| VPS28 | Variation_4613 | chr8:145565803..145769410 |
| SNORA11 | Variation_23256 | chrX:52891008..55679746 |
| OR56A1 | Variation_34458 | chr11:5505764..6194475 |
| C1orf150 | Variation_6816 | chr1:247074430..248328835 |
| CPSF1 | Variation_30306 | chr8:144992102..145744618 |
| MUC4 | Variation_0568 | chr3:195433152..197378091 |
| EGFL7 | Variation_3785 | chr9:139250829..139826485 |
| UBL7 | Variation_37170 | chr15:74364360..75569130 |
| KIAA1967 | Variation_2746 | chr8:21673762..22802380 |
| PDDC1 | Variation_3831 | chr11:409846..1357392 |
| FOXH1 | Variation_30306 | chr8:144992102..145744618 |
| ZDHHC19 | Variation_37243 | chr3:195397785..197386290 |
| EXD3 | Variation_5415 | chr9:139478587..140244550 |
| TRIM58 | Variation_6816 | chr1:247074430..248328835 |
| RANBP1 | Variation_31071 | chr22:19019088..21053198 |
| FAM160B2 | Variation_3726 | chr8:20533044..23111609 |
| PCYT1A | Variation_37243 | chr3:195397785..197386290 |
| COMT | Variation_31071 | chr22:19019088..21053198 |
| KIAA1967 | Variation_3726 | chr8:20533044..23111609 |
| PDDC1 | Variation_29883 | chr11:378188..870446 |
| SNORA17 | Variation_30350 | chr9:139254317..139950351 |

**Table S5:** Significant GO terms of the Genes

| Term | Count | % | PValue | Benjamini |
| --- | --- | --- | --- | --- |
| Alternative splicing | 597 | 45.3303 | 5.58E-09 | 2.86E-06 |
| Splice variant | 599 | 45.48216 | 4.02E-09 | 1.18E-05 |
| Cytoplasm | 291 | 22.09567 | 2.59E-07 | 6.63E-05 |
| Phosphoprotein | 564 | 42.8246 | 1.35E-06 | 2.31E-04 |
| RNA-binding | 60 | 4.555809 | 1.18E-04 | 0.01497394 |
| Translation regulation | 14 | 1.063022 | 4.10E-04 | 0.04110072 |
| Membrane-enclosed lumen | 154 | 11.69324 | 5.42E-04 | 0.0504102 |

**Table S6:** Significant KEGG pathways of the Genes

| Term | Count | % | PValue | Benjamini |
| --- | --- | --- | --- | --- |
| Axon guidance | 15 | 1.138952 | 0.009362 | 0.780042 |
| ErbB signaling pathway | 11 | 0.835232 | 0.01816 | 0.771302 |
| Endocytosis | 17 | 1.290812 | 0.039426 | 0.884527 |
| Homologous recombination | 5 | 0.379651 | 0.061616 | 0.922676 |

**Table S7:** List of the overlapping QTLs

| Chr | Start | End | Relative traits |
| --- | --- | --- | --- |
| 1 | 2701298 | 7964370 | Reproduction_QTL |
| 1 | 2701298 | 11729223 | Production_QTL |
| 1 | 2701298 | 16114132 | Meat_Quality_QTL |
| 1 | 2701298 | 16114132 | Exterior_QTL |
| 1 | 7150722 | 7565527 | Meat_Quality_eQTL |
| 1 | 7756967 | 8171773 | Meat_Quality_QTL |
| 1 | 7756967 | 8171773 | Meat_Quality_eQTL |
| 1 | 7964370 | 16114132 | Meat_Quality_QTL |
| 1 | 7964370 | 22514169 | Meat_Quality_QTL |
| 1 | 7964370 | 2.58E+08 | Meat_Quality_QTL |
| 1 | 7964370 | 2.89E+08 | Meat_Quality_QTL |
| 1 | 7964370 | 2.99E+08 | Production_QTL |
| 1 | 10692209 | 16114132 | Production_QTL |
| 1 | 10692209 | 52874641 | Health_QTL |
| 1 | 11771656 | 16114132 | Production_QTL |
| 1 | 11771656 | 16114132 | Health_Association |
| 1 | 11979059 | 12393864 | Meat_Quality_QTL |
| 1 | 12351432 | 12766237 | Health_QTL |
| 1 | 12816427 | 13231233 | Meat_Quality_eQTL |
| 1 | 13264004 | 13678810 | Meat_Quality_eQTL |
| 1 | 14869715 | 15284521 | Meat_Quality_eQTL |
| 1 | 15077118 | 15491924 | Meat_Quality_QTL |
| 1 | 15284521 | 50034644 | Meat_Quality_QTL |
| 1 | 15317292 | 15732097 | Meat_Quality_eQTL |
| 1 | 15380465 | 15795270 | Meat_Quality_eQTL |
| 1 | 16114132 | 22514169 | Meat_Quality_QTL |
| 1 | 16114132 | 52874641 | Meat_Quality_QTL |
| 1 | 16114132 | 62261447 | Meat_Quality_QTL |
| 1 | 16114132 | 2.89E+08 | Production_QTL |
| 1 | 16114132 | 2.95E+08 | Meat_Quality_QTL |
| 1 | 16114132 | 2.99E+08 | Meat_Quality_QTL |
| 1 | 16114132 | 3.11E+08 | Meat_Quality_QTL |
| 1 | 16334518 | 16749323 | Health_QTL |
| 1 | 16736340 | 2.42E+08 | Health_QTL |
| 1 | 21100467 | 39724691 | Meat_Quality_eQTL |
| 1 | 22514169 | 52874641 | Meat_Quality_QTL |
| 1 | 24173391 | 2.99E+08 | Production_QTL |
| 1 | 24647055 | 52874641 | Meat_Quality_QTL |
| 1 | 24647055 | 2.58E+08 | Meat_Quality_QTL |
| 1 | 26484272 | 2.42E+08 | Health_QTL |
| 1 | 34352834 | 52045030 | Meat_Quality_QTL |
| 1 | 35389848 | 52874641 | Meat_Quality_QTL |
| 1 | 35389848 | 52874641 | Meat_Quality_QTL |
| 1 | 35389848 | 1.69E+08 | Production_QTL |
| 1 | 35389848 | 2.54E+08 | Health_QTL |
| 1 | 35597251 | 53289447 | Meat_Quality_QTL |
| 1 | 36144885 | 1.54E+08 | Meat_Quality_eQTL |
| 1 | 36352288 | 2.95E+08 | Reproduction_QTL |
| 1 | 36974496 | 52874641 | Meat_Quality_QTL |
| 1 | 38198173 | 38612978 | Meat_Quality_eQTL |
| 1 | 51153198 | 51568003 | Meat_Quality_eQTL |
| 1 | 52045030 | 2.56E+08 | Health_QTL |
| 1 | 52667238 | 53082044 | Health_QTL |
| 1 | 52874641 | 1.69E+08 | Meat_Quality_QTL |
| 1 | 52874641 | 1.69E+08 | Production_QTL |
| 1 | 52874641 | 2.34E+08 | Meat_Quality_QTL |
| 1 | 53227226 | 53642031 | Meat_Quality_eQTL |
| 1 | 62261447 | 1.33E+08 | Production_QTL |
| 1 | 62261447 | 1.33E+08 | Health_QTL |
| 1 | 62261447 | 2.34E+08 | Meat_Quality_QTL |
| 1 | 62261447 | 2.34E+08 | Exterior_QTL |
| 1 | 81895889 | 2.58E+08 | Exterior_QTL |
| 1 | 97537601 | 1.54E+08 | Meat_Quality_QTL |
| 1 | 97537601 | 1.69E+08 | Meat_Quality_QTL |
| 1 | 97537601 | 2.81E+08 | Meat_Quality_QTL |
| 1 | 97537601 | 2.89E+08 | Meat_Quality_QTL |
| 1 | 97537601 | 2.94E+08 | Production_QTL |
| 1 | 1.33E+08 | 1.33E+08 | Meat_Quality_eQTL |
| 1 | 1.33E+08 | 1.33E+08 | Production_Association |
| 1 | 1.33E+08 | 1.33E+08 | Meat_Quality_Association |
| 1 | 1.33E+08 | 1.33E+08 | Exterior_QTL |
| 1 | 1.33E+08 | 1.54E+08 | Meat_Quality_QTL |
| 1 | 1.33E+08 | 2.42E+08 | Meat_Quality_QTL |
| 1 | 1.33E+08 | 2.77E+08 | Health_QTL |
| 1 | 1.54E+08 | 1.54E+08 | Health_QTL |
| 1 | 1.56E+08 | 2.82E+08 | Meat_Quality_QTL |
| 1 | 1.57E+08 | 3.15E+08 | Reproduction_QTL |
| 1 | 1.58E+08 | 2.34E+08 | Exterior_QTL |
| 1 | 1.61E+08 | 2.77E+08 | Meat_Quality_QTL |
| 1 | 1.69E+08 | 2.34E+08 | Meat_Quality_QTL |
| 1 | 1.69E+08 | 2.81E+08 | Production_QTL |
| 1 | 1.69E+08 | 2.93E+08 | Meat_Quality_QTL |
| 1 | 1.69E+08 | 2.99E+08 | Meat_Quality_QTL |
| 1 | 1.72E+08 | 1.72E+08 | Meat_Quality_eQTL |
| 1 | 1.72E+08 | 1.73E+08 | Meat_Quality_Association |
| 1 | 1.73E+08 | 2.45E+08 | Meat_Quality_QTL |
| 1 | 1.91E+08 | 2.93E+08 | Health_QTL |
| 1 | 1.97E+08 | 2.34E+08 | Meat_Quality_QTL |
| 1 | 1.97E+08 | 2.34E+08 | Production_QTL |
| 1 | 1.97E+08 | 2.34E+08 | Exterior_QTL |
| 1 | 1.97E+08 | 2.58E+08 | Health_Association |
| 1 | 2.11E+08 | 2.81E+08 | Meat_Quality_QTL |
| 1 | 2.11E+08 | 2.89E+08 | Meat_Quality_QTL |
| 1 | 2.11E+08 | 2.99E+08 | Production_QTL |
| 1 | 2.11E+08 | 2.58E+08 | Reproduction_QTL |
| 1 | 2.16E+08 | 2.52E+08 | Meat_Quality_QTL |
| 1 | 2.16E+08 | 2.53E+08 | Meat_Quality_QTL |
| 1 | 2.27E+08 | 2.34E+08 | Meat_Quality_QTL |
| 1 | 2.27E+08 | 2.94E+08 | Meat_Quality_QTL |
| 1 | 2.31E+08 | 2.42E+08 | Meat_Quality_eQTL |
| 1 | 2.34E+08 | 2.34E+08 | Health_QTL |
| 1 | 2.34E+08 | 2.34E+08 | Meat_Quality_eQTL |
| 1 | 2.34E+08 | 2.42E+08 | Meat_Quality_QTL |
| 1 | 2.34E+08 | 2.54E+08 | Health_QTL |
| 1 | 2.34E+08 | 2.58E+08 | Health_QTL |
| 1 | 2.34E+08 | 2.58E+08 | Health_QTL |
| 1 | 2.34E+08 | 2.81E+08 | Exterior_QTL |
| 1 | 2.43E+08 | 2.44E+08 | Production_QTL |
| 1 | 2.44E+08 | 2.99E+08 | Meat_Quality_QTL |
| 1 | 2.44E+08 | 2.44E+08 | Meat_Quality_eQTL |
| 1 | 2.48E+08 | 2.58E+08 | Meat_Quality_QTL |
| 1 | 2.48E+08 | 2.77E+08 | Meat_Quality_QTL |
| 1 | 2.48E+08 | 2.77E+08 | Production_QTL |
| 1 | 2.52E+08 | 2.93E+08 | Reproduction_QTL |
| 1 | 2.55E+08 | 2.56E+08 | Meat_Quality_QTL |
| 1 | 2.58E+08 | 2.89E+08 | Exterior_QTL |
| 1 | 2.58E+08 | 2.89E+08 | Exterior_QTL |
| 1 | 2.58E+08 | 2.92E+08 | Production_QTL |
| 1 | 2.58E+08 | 2.92E+08 | Meat_Quality_QTL |
| 1 | 2.58E+08 | 2.99E+08 | Exterior_QTL |
| 1 | 2.58E+08 | 2.99E+08 | Production_QTL |
| 1 | 2.58E+08 | 3.15E+08 | Health_QTL |
| 1 | 2.65E+08 | 2.99E+08 | Reproduction_QTL |
| 1 | 2.65E+08 | 2.99E+08 | Meat_Quality_QTL |
| 1 | 2.65E+08 | 2.99E+08 | Production_QTL |
| 1 | 2.65E+08 | 2.99E+08 | Meat_Quality_QTL |
| 1 | 2.66E+08 | 2.77E+08 | Meat_Quality_QTL |
| 1 | 2.77E+08 | 2.81E+08 | Exterior_QTL |
| 1 | 2.77E+08 | 2.92E+08 | Meat_Quality_QTL |
| 1 | 2.77E+08 | 2.92E+08 | Meat_Quality_QTL |
| 1 | 2.81E+08 | 2.94E+08 | Production_QTL |
| 1 | 2.81E+08 | 2.94E+08 | Meat_Quality_QTL |
| 1 | 2.81E+08 | 2.99E+08 | Meat_Quality_QTL |
| 1 | 2.82E+08 | 2.89E+08 | Meat_Quality_QTL |
| 1 | 2.9E+08 | 2.91E+08 | Meat_Quality_QTL |
| 1 | 2.91E+08 | 2.91E+08 | Production_QTL |
| 1 | 2.91E+08 | 2.92E+08 | Reproduction_QTL |
| 1 | 2.91E+08 | 2.92E+08 | Meat_Quality_eQTL |
| 1 | 2.92E+08 | 2.92E+08 | Meat_Quality_QTL |
| 1 | 2.92E+08 | 2.92E+08 | Meat_Quality_eQTL |
| 1 | 2.92E+08 | 2.99E+08 | Meat_Quality_QTL |
| 1 | 2.92E+08 | 2.93E+08 | Meat_Quality_eQTL |
| 1 | 2.92E+08 | 2.93E+08 | Meat_Quality_QTL |
| 1 | 2.92E+08 | 2.99E+08 | Production_QTL |
| 1 | 2.92E+08 | 2.99E+08 | Meat_Quality_QTL |
| 1 | 2.92E+08 | 3.06E+08 | Meat_Quality_QTL |
| 1 | 2.92E+08 | 3.06E+08 | Production_QTL |
| 1 | 2.92E+08 | 3.15E+08 | Reproduction_QTL |
| 1 | 2.93E+08 | 2.94E+08 | Meat_Quality_eQTL |
| 1 | 2.93E+08 | 2.94E+08 | Meat_Quality_QTL |
| 1 | 2.94E+08 | 2.94E+08 | Meat_Quality_QTL |
| 1 | 2.94E+08 | 2.99E+08 | Production_QTL |
| 1 | 2.94E+08 | 2.94E+08 | Meat_Quality_QTL |
| 1 | 2.94E+08 | 2.95E+08 | Production_Association |
| 1 | 2.94E+08 | 2.95E+08 | Meat_Quality_Association |
| 1 | 2.94E+08 | 2.95E+08 | Meat_Quality_eQTL |
| 1 | 2.94E+08 | 2.99E+08 | Production_QTL |
| 1 | 2.94E+08 | 2.99E+08 | Meat_Quality_QTL |
| 1 | 2.95E+08 | 2.95E+08 | Meat_Quality_QTL |
| 1 | 2.95E+08 | 2.96E+08 | Meat_Quality_eQTL |
| 1 | 2.95E+08 | 2.96E+08 | Meat_Quality_QTL |
| 1 | 2.96E+08 | 3.06E+08 | Exterior_QTL |
| 1 | 2.98E+08 | 3.15E+08 | Meat_Quality_QTL |
| 1 | 2.99E+08 | 3.06E+08 | Meat_Quality_QTL |
| 1 | 3E+08 | 3.15E+08 | Meat_Quality_QTL |
| 1 | 3.01E+08 | 3.11E+08 | Meat_Quality_QTL |
| 1 | 3.02E+08 | 3.06E+08 | Meat_Quality_QTL |
| 1 | 3.02E+08 | 3.15E+08 | Meat_Quality_eQTL |
| 1 | 3.04E+08 | 3.15E+08 | Meat_Quality_QTL |
| 1 | 3.08E+08 | 3.15E+08 | Meat_Quality_QTL |
| 1 | 3.1E+08 | 3.15E+08 | Production_QTL |
| 1 | 3.15E+08 | 3.15E+08 | Meat_Quality_QTL |
| 1 | 3.15E+08 | 3.15E+08 | Production_QTL |
| 1 | 3.15E+08 | 3.15E+08 | Meat_Quality_eQTL |
| 1 | 3.15E+08 | 3.15E+08 | Meat_Quality_Association |
| 2 | 0 | 2091144 | Meat_Quality_QTL |
| 2 | 0 | 2259808 | Meat_Quality_QTL |
| 2 | 0 | 2387169 | Meat_Quality_QTL |
| 2 | 0 | 2387169 | Meat_Quality_QTL |
| 2 | 0 | 2641892 | Meat_Quality_QTL |
| 2 | 0 | 2641892 | Reproduction_QTL |
| 2 | 0 | 5655742 | Meat_Quality_QTL |
| 2 | 0 | 5911986 | Meat_Quality_QTL |
| 2 | 0 | 10464301 | Reproduction_QTL |
| 2 | 0 | 10846385 | Meat_Quality_QTL |
| 2 | 0 | 10973746 | Meat_Quality_QTL |
| 2 | 0 | 11838195 | Meat_Quality_QTL |
| 2 | 0 | 13002292 | Meat_Quality_QTL |
| 2 | 0 | 13366532 | Meat_Quality_QTL |
| 2 | 0 | 1.53E+08 | Production_Association |
| 2 | 222025 | 476748 | Production_Association |
| 2 | 222025 | 476748 | Meat_Quality_Association |
| 2 | 349387 | 1.48E+08 | Meat_Quality_QTL |
| 2 | 1495639 | 1750362 | Meat_Quality_QTL |
| 2 | 1623001 | 1877723 | Meat_Quality_QTL |
| 2 | 1963783 | 2218506 | Meat_Quality_QTL |
| 2 | 2005085 | 2259808 | Meat_Quality_QTL |
| 2 | 2345867 | 2600590 | Meat_Quality_QTL |
| 2 | 2387169 | 2641892 | Meat_Quality_QTL |
| 2 | 2387169 | 4256288 | Meat_Quality_QTL |
| 2 | 2387169 | 13366532 | Production_QTL |
| 2 | 2387169 | 13366532 | Production_QTL |
| 2 | 2387169 | 13366532 | Meat_Quality_QTL |
| 2 | 2387169 | 21136167 | Meat_Quality_QTL |
| 2 | 2387169 | 21136167 | Health_QTL |
| 2 | 2387169 | 38367026 | Health_QTL |
| 2 | 2387169 | 1.33E+08 | Health_QTL |
| 2 | 2387169 | 1.53E+08 | Health_QTL |
| 2 | 2473228 | 2727951 | Meat_Quality_QTL |
| 2 | 2514530 | 2769253 | Meat_Quality_QTL |
| 2 | 2982674 | 3237397 | Production_QTL |
| 2 | 3746842 | 4001565 | Meat_Quality_QTL |
| 2 | 4042867 | 4297590 | Meat_Quality_QTL |
| 2 | 5189120 | 5443843 | Meat_Quality_QTL |
| 2 | 5443843 | 1.46E+08 | Production_QTL |
| 2 | 5655742 | 10751719 | Health_QTL |
| 2 | 5783104 | 10879081 | Meat_Quality_QTL |
| 2 | 5784625 | 6039348 | Meat_Quality_QTL |
| 2 | 6166709 | 6421432 | Meat_Quality_QTL |
| 2 | 6166709 | 6421432 | Production_QTL |
| 2 | 6419911 | 21506294 | Health_QTL |
| 2 | 7440323 | 13366532 | Meat_Quality_QTL |
| 2 | 7440323 | 38367026 | Health_QTL |
| 2 | 7820886 | 21136167 | Meat_Quality_QTL |
| 2 | 9445410 | 10750198 | Meat_Quality_QTL |
| 2 | 10495475 | 10750198 | Reproduction_QTL |
| 2 | 10846385 | 11101108 | Health_QTL |
| 2 | 10846385 | 21136167 | Meat_Quality_QTL |
| 2 | 13366532 | 21136167 | Meat_Quality_QTL |
| 2 | 13366532 | 21136167 | Reproduction_QTL |
| 2 | 13366532 | 26780855 | Meat_Quality_QTL |
| 2 | 13366532 | 44483418 | Meat_Quality_QTL |
| 2 | 13366532 | 44483418 | Meat_Quality_QTL |
| 2 | 13366532 | 1E+08 | Meat_Quality_QTL |
| 2 | 13366532 | 1.53E+08 | Health_QTL |
| 2 | 14021183 | 14275906 | Meat_Quality_QTL |
| 2 | 15902392 | 26780855 | Meat_Quality_QTL |
| 2 | 21008806 | 21263528 | Exterior_QTL |
| 2 | 21136167 | 35687241 | Exterior_QTL |
| 2 | 21136167 | 38367026 | Meat_Quality_QTL |
| 2 | 21136167 | 44483418 | Meat_Quality_QTL |
| 2 | 21136167 | 59648728 | Meat_Quality_QTL |
| 2 | 21136167 | 75040316 | Meat_Quality_QTL |
| 2 | 21136167 | 84721802 | Meat_Quality_QTL |
| 2 | 21136167 | 1.19E+08 | Meat_Quality_QTL |
| 2 | 21136167 | 1.25E+08 | Meat_Quality_QTL |
| 2 | 21251571 | 21506294 | Meat_Quality_QTL |
| 2 | 21518251 | 53611728 | Meat_Quality_QTL |
| 2 | 25252518 | 64352885 | Meat_Quality_QTL |
| 2 | 26653493 | 26908216 | Exterior_QTL |
| 2 | 26780855 | 75082926 | Production_QTL |
| 2 | 26780855 | 1E+08 | Production_QTL |
| 2 | 26780855 | 1.33E+08 | Meat_Quality_QTL |
| 2 | 30953889 | 31208612 | Meat_Quality_QTL |
| 2 | 31335973 | 75040316 | Meat_Quality_QTL |
| 2 | 32864310 | 35687241 | Meat_Quality_QTL |
| 2 | 35432518 | 35687241 | Meat_Quality_QTL |
| 2 | 38112303 | 38367026 | Production_QTL |
| 2 | 38366026 | 38367026 | Health_QTL |
| 2 | 38367026 | 59648728 | Meat_Quality_QTL |
| 2 | 38367026 | 59648728 | Meat_Quality_QTL |
| 2 | 38367026 | 1E+08 | Meat_Quality_QTL |
| 2 | 38367026 | 1.19E+08 | Production_QTL |
| 2 | 43611141 | 59648728 | Reproduction_QTL |
| 2 | 44483418 | 75040316 | Meat_Quality_QTL |
| 2 | 48775112 | 75082926 | Meat_Quality_QTL |
| 2 | 53147173 | 1.24E+08 | Meat_Quality_QTL |
| 2 | 53357005 | 53611728 | Health_QTL |
| 2 | 53357005 | 53611728 | Meat_Quality_QTL |
| 2 | 54121174 | 54375896 | Meat_Quality_QTL |
| 2 | 59394005 | 59648728 | Health_QTL |
| 2 | 59521367 | 64607608 | Meat_Quality_QTL |
| 2 | 64225524 | 64480246 | Meat_Quality_QTL |
| 2 | 64225524 | 64480246 | Health_QTL |
| 2 | 74785593 | 75040316 | Meat_Quality_QTL |
| 2 | 75040316 | 1.19E+08 | Meat_Quality_QTL |
| 2 | 75082926 | 1E+08 | Meat_Quality_QTL |
| 2 | 1.33E+08 | 1.53E+08 | Meat_Quality_QTL |
| 2 | 1.35E+08 | 1.53E+08 | Meat_Quality_QTL |
| 2 | 1.39E+08 | 1.46E+08 | Reproduction_QTL |
| 2 | 1.44E+08 | 1.53E+08 | Meat_Quality_QTL |
| 2 | 1.44E+08 | 1.53E+08 | Production_QTL |
| 2 | 1.44E+08 | 1.53E+08 | Health_QTL |
| 2 | 1.5E+08 | 1.53E+08 | Meat_Quality_QTL |
| 3 | 14776389 | 1E+08 | Reproduction_QTL |
| 3 | 21401456 | 46660362 | Meat_Quality_QTL |
| 3 | 21824455 | 56651690 | Meat_Quality_QTL |
| 3 | 21824455 | 1.15E+08 | Meat_Quality_QTL |
| 3 | 21947993 | 46839405 | Reproduction_QTL |
| 3 | 21947993 | 46839405 | Meat_Quality_QTL |
| 3 | 21947993 | 56651690 | Production_QTL |
| 3 | 21947993 | 56651690 | Meat_Quality_QTL |
| 3 | 21947993 | 76622784 | Health_QTL |
| 3 | 21947993 | 76622784 | Meat_Quality_QTL |
| 3 | 37870100 | 47426409 | Meat_Quality_QTL |
| 3 | 41794716 | 46627906 | Meat_Quality_QTL |
| 3 | 46448862 | 46660362 | Meat_Quality_QTL |
| 3 | 46733655 | 46945155 | Health_QTL |
| 3 | 46839405 | 1E+08 | Health_QTL |
| 3 | 54430946 | 54642446 | Meat_Quality_QTL |
| 3 | 54748196 | 54959695 | Meat_Quality_QTL |
| 3 | 56017192 | 1.03E+08 | Exterior_QTL |
| 3 | 56651690 | 76622784 | Meat_Quality_QTL |
| 3 | 56651690 | 1.15E+08 | Meat_Quality_QTL |
| 3 | 56651690 | 1.2E+08 | Production_QTL |
| 3 | 62780348 | 1E+08 | Health_QTL |
| 3 | 76622784 | 1.2E+08 | Meat_Quality_QTL |
| 3 | 76622784 | 1.2E+08 | Production_QTL |
| 3 | 1.02E+08 | 1.03E+08 | Meat_Quality_QTL |
| 3 | 1.03E+08 | 1.15E+08 | Exterior_QTL |
| 3 | 1.12E+08 | 1.15E+08 | Meat_Quality_QTL |
| 3 | 1.15E+08 | 1.2E+08 | Production_QTL |
| 4 | 0 | 12618993 | Meat_Quality_QTL |
| 4 | 0 | 12618993 | Meat_Quality_QTL |
| 4 | 1625349 | 1843156 | Meat_Quality_Association |
| 4 | 1625349 | 1843156 | Exterior_Association |
| 4 | 2866848 | 5047256 | Production_QTL |
| 4 | 3846980 | 4064787 | Health_QTL |
| 4 | 4064787 | 12618993 | Meat_Quality_QTL |
| 4 | 4064787 | 12618993 | Meat_Quality_QTL |
| 4 | 4064787 | 12688770 | Production_QTL |
| 4 | 4064787 | 23809394 | Reproduction_QTL |
| 4 | 4064787 | 23809394 | Meat_Quality_QTL |
| 4 | 4064787 | 23809394 | Reproduction_QTL |
| 4 | 4064787 | 35398368 | Meat_Quality_QTL |
| 4 | 4064787 | 52626380 | Production_QTL |
| 4 | 4064787 | 79858331 | Reproduction_QTL |
| 4 | 4064787 | 82084284 | Meat_Quality_Association |
| 4 | 6584218 | 12618993 | Production_QTL |
| 4 | 7237639 | 12618993 | Meat_Quality_QTL |
| 4 | 7237639 | 12618993 | Production_QTL |
| 4 | 11908177 | 45038434 | Health_QTL |
| 4 | 12401186 | 12618993 | Production_QTL |
| 4 | 12618993 | 23809394 | Production_QTL |
| 4 | 12618993 | 71310074 | Meat_Quality_QTL |
| 4 | 12618993 | 1.02E+08 | Meat_Quality_QTL |
| 4 | 12618993 | 1.23E+08 | Meat_Quality_QTL |
| 4 | 14860884 | 1.28E+08 | Production_QTL |
| 4 | 16391489 | 1.29E+08 | Production_QTL |
| 4 | 22254690 | 1.19E+08 | Production_QTL |
| 4 | 23809394 | 35289465 | Meat_Quality_QTL |
| 4 | 23809394 | 82084284 | Production_QTL |
| 4 | 23809394 | 82084284 | Production_QTL |
| 4 | 23809394 | 96936045 | Production_QTL |
| 4 | 23809394 | 1.02E+08 | Production_QTL |
| 4 | 23809394 | 1.02E+08 | Reproduction_QTL |
| 4 | 32292291 | 1.12E+08 | Meat_Quality_QTL |
| 4 | 32292291 | 1.19E+08 | Meat_Quality_QTL |
| 4 | 33054616 | 97629582 | Meat_Quality_QTL |
| 4 | 34252554 | 52626380 | Production_QTL |
| 4 | 34252554 | 1.02E+08 | Meat_Quality_QTL |
| 4 | 34252554 | 1.23E+08 | Production_QTL |
| 4 | 34309333 | 47587471 | Meat_Quality_QTL |
| 4 | 34309333 | 48676506 | Meat_Quality_QTL |
| 4 | 35289465 | 69458714 | Meat_Quality_QTL |
| 4 | 35289465 | 84951475 | Meat_Quality_QTL |
| 4 | 38684579 | 71310074 | Meat_Quality_QTL |
| 4 | 46825146 | 62904164 | Production_QTL |
| 4 | 46825146 | 62904164 | Meat_Quality_QTL |
| 4 | 46912269 | 47696374 | Meat_Quality_Association |
| 4 | 47696374 | 47914182 | Meat_Quality_QTL |
| 4 | 47805278 | 1.13E+08 | Production_QTL |
| 4 | 48676506 | 48894313 | Exterior_QTL |
| 4 | 48894313 | 1.1E+08 | Production_QTL |
| 4 | 49438830 | 49656638 | Meat_Quality_QTL |
| 4 | 49438830 | 49656638 | Reproduction_QTL |
| 4 | 49656638 | 49874444 | Meat_Quality_QTL |
| 4 | 49656638 | 49874444 | Exterior_QTL |
| 4 | 49689309 | 1.07E+08 | Production_Association |
| 4 | 49689309 | 1.07E+08 | Meat_Quality_Association |
| 4 | 49983348 | 1.07E+08 | Meat_Quality_QTL |
| 4 | 51755152 | 51972959 | Meat_Quality_QTL |
| 4 | 51755152 | 51972959 | Meat_Quality_Association |
| 4 | 51755152 | 51972959 | Production_Association |
| 4 | 51864056 | 61134234 | Meat_Quality_Association |
| 4 | 51864056 | 61134234 | Production_Association |
| 4 | 52517476 | 61924032 | Meat_Quality_QTL |
| 4 | 52517476 | 61924032 | Production_QTL |
| 4 | 52517476 | 61924032 | Meat_Quality_Association |
| 4 | 52517476 | 61924032 | Production_Association |
| 4 | 52626380 | 62032936 | Meat_Quality_QTL |
| 4 | 52626380 | 71310074 | Production_QTL |
| 4 | 52626380 | 71310074 | Meat_Quality_QTL |
| 4 | 52626380 | 1.02E+08 | Meat_Quality_QTL |
| 4 | 52626380 | 1.02E+08 | Production_QTL |
| 4 | 52626380 | 1.4E+08 | Meat_Quality_QTL |
| 4 | 60807524 | 1.1E+08 | Production_QTL |
| 4 | 60807524 | 1.19E+08 | Meat_Quality_QTL |
| 4 | 60916427 | 61134234 | Production_QTL |
| 4 | 60916427 | 61134234 | Exterior_QTL |
| 4 | 61924032 | 82084284 | Meat_Quality_QTL |
| 4 | 70983364 | 1.07E+08 | Meat_Quality_QTL |
| 4 | 71310074 | 82084284 | Meat_Quality_QTL |
| 4 | 71310074 | 82084284 | Meat_Quality_QTL |
| 4 | 71310074 | 1.23E+08 | Production_QTL |
| 4 | 71310074 | 1.4E+08 | Meat_Quality_QTL |
| 4 | 76464120 | 96500431 | Meat_Quality_QTL |
| 4 | 77094703 | 1.02E+08 | Meat_Quality_QTL |
| 4 | 77094703 | 1.07E+08 | Exterior_QTL |
| 4 | 79640524 | 79858331 | Meat_Quality_QTL |
| 4 | 79640524 | 79858331 | Exterior_QTL |
| 4 | 82084284 | 1.07E+08 | Meat_Quality_QTL |
| 4 | 84733668 | 84951475 | Meat_Quality_QTL |
| 4 | 84733668 | 84951475 | Meat_Quality_QTL |
| 4 | 84733668 | 1.13E+08 | Meat_Quality_QTL |
| 4 | 84951475 | 96936045 | Meat_Quality_QTL |
| 4 | 84951475 | 1.07E+08 | Meat_Quality_QTL |
| 4 | 84951475 | 1.23E+08 | Production_QTL |
| 4 | 89529616 | 96936045 | Meat_Quality_QTL |
| 4 | 89529616 | 96936045 | Meat_Quality_QTL |
| 4 | 89529616 | 98283003 | Meat_Quality_QTL |
| 4 | 89529616 | 98283003 | Production_QTL |
| 4 | 90618651 | 1.19E+08 | Meat_Quality_QTL |
| 4 | 95738106 | 95955914 | Meat_Quality_QTL |
| 4 | 95738106 | 98718617 | Meat_Quality_QTL |
| 4 | 96391528 | 96609334 | Meat_Quality_QTL |
| 4 | 96500431 | 1.12E+08 | Meat_Quality_QTL |
| 4 | 96609334 | 96827141 | Meat_Quality_QTL |
| 4 | 96827141 | 97044948 | Meat_Quality_Association |
| 4 | 96827141 | 97044948 | Meat_Quality_QTL |
| 4 | 96936045 | 98283003 | Meat_Quality_QTL |
| 4 | 96936045 | 1.02E+08 | Meat_Quality_QTL |
| 4 | 96936045 | 1.02E+08 | Production_QTL |
| 4 | 96936045 | 1.07E+08 | Reproduction_QTL |
| 4 | 96936045 | 1.07E+08 | Meat_Quality_QTL |
| 4 | 96936045 | 1.07E+08 | Meat_Quality_QTL |
| 4 | 96936045 | 1.23E+08 | Production_QTL |
| 4 | 97153852 | 97371659 | Meat_Quality_QTL |
| 4 | 97262756 | 97480562 | Exterior_QTL |
| 4 | 97480562 | 97698369 | Meat_Quality_QTL |
| 4 | 97480562 | 97698369 | Health_QTL |
| 4 | 97589466 | 1.12E+08 | Meat_Quality_QTL |
| 4 | 98720794 | 1.3E+08 | Health_QTL |
| 4 | 1.02E+08 | 1.07E+08 | Meat_Quality_QTL |
| 4 | 1.02E+08 | 1.35E+08 | Health_QTL |
| 4 | 1.02E+08 | 1.4E+08 | Meat_Quality_QTL |
| 4 | 1.07E+08 | 1.4E+08 | Meat_Quality_QTL |
| 4 | 1.07E+08 | 1.29E+08 | Meat_Quality_QTL |
| 4 | 1.13E+08 | 1.19E+08 | Meat_Quality_QTL |
| 4 | 1.16E+08 | 1.3E+08 | Meat_Quality_QTL |
| 4 | 1.19E+08 | 1.19E+08 | Production_QTL |
| 4 | 1.19E+08 | 1.19E+08 | Meat_Quality_QTL |
| 4 | 1.19E+08 | 1.19E+08 | Meat_Quality_Association |
| 4 | 1.19E+08 | 1.19E+08 | Production_Association |
| 4 | 1.19E+08 | 1.23E+08 | Meat_Quality_QTL |
| 4 | 1.19E+08 | 1.35E+08 | Meat_Quality_QTL |
| 4 | 1.2E+08 | 1.2E+08 | Meat_Quality_QTL |
| 4 | 1.2E+08 | 1.2E+08 | Meat_Quality_QTL |
| 4 | 1.21E+08 | 1.4E+08 | Health_QTL |
| 4 | 1.23E+08 | 1.35E+08 | Meat_Quality_QTL |
| 4 | 1.25E+08 | 1.35E+08 | Meat_Quality_QTL |
| 4 | 1.25E+08 | 1.4E+08 | Exterior_QTL |
| 4 | 1.25E+08 | 1.4E+08 | Meat_Quality_QTL |
| 4 | 1.28E+08 | 1.29E+08 | Meat_Quality_QTL |
| 4 | 1.28E+08 | 1.35E+08 | Meat_Quality_QTL |
| 4 | 1.29E+08 | 1.3E+08 | Production_QTL |
| 4 | 1.3E+08 | 1.31E+08 | Meat_Quality_QTL |
| 4 | 1.31E+08 | 1.31E+08 | Meat_Quality_QTL |
| 4 | 1.31E+08 | 1.31E+08 | Production_QTL |
| 4 | 1.31E+08 | 1.31E+08 | Health_QTL |
| 4 | 1.31E+08 | 1.35E+08 | Meat_Quality_QTL |
| 4 | 1.32E+08 | 1.32E+08 | Meat_Quality_QTL |
| 4 | 1.35E+08 | 1.35E+08 | Meat_Quality_QTL |
| 4 | 1.4E+08 | 1.4E+08 | Meat_Quality_QTL |
| 5 | 0 | 6647815 | Meat_Quality_QTL |
| 5 | 0 | 6647815 | Meat_Quality_QTL |
| 5 | 0 | 6647815 | Meat_Quality_QTL |
| 5 | 0 | 7985523 | Meat_Quality_QTL |
| 5 | 0 | 7985523 | Exterior_QTL |
| 5 | 0 | 35377762 | Health_QTL |
| 5 | 0 | 68590401 | Reproduction_QTL |
| 5 | 494956 | 674268 | Meat_Quality_QTL |
| 5 | 584612 | 77414253 | Meat_Quality_QTL |
| 5 | 1391516 | 1570828 | Meat_Quality_QTL |
| 5 | 1839796 | 6647815 | Exterior_QTL |
| 5 | 3632916 | 3812228 | Health_QTL |
| 5 | 3722572 | 7985523 | Meat_Quality_QTL |
| 5 | 3722572 | 35377762 | Meat_Quality_QTL |
| 5 | 4170852 | 33789566 | Meat_Quality_QTL |
| 5 | 4668251 | 71054179 | Exterior_QTL |
| 5 | 5246724 | 69217993 | Exterior_QTL |
| 5 | 5385499 | 5564811 | Meat_Quality_QTL |
| 5 | 6002292 | 6647815 | Meat_Quality_Association |
| 5 | 6468503 | 6647815 | Meat_Quality_QTL |
| 5 | 6558159 | 6737471 | Health_QTL |
| 5 | 6647815 | 7985523 | Production_QTL |
| 5 | 6647815 | 19558279 | Reproduction_QTL |
| 5 | 6647815 | 19558279 | Production_QTL |
| 5 | 6647815 | 22412792 | Exterior_QTL |
| 5 | 6647815 | 33251630 | Exterior_QTL |
| 5 | 6647815 | 35377762 | Exterior_QTL |
| 5 | 6647815 | 71054179 | Health_QTL |
| 5 | 6737471 | 62167964 | Health_QTL |
| 5 | 6770876 | 6950188 | Production_QTL |
| 5 | 7096095 | 71907673 | Meat_Quality_QTL |
| 5 | 7268275 | 7447587 | Meat_Quality_QTL |
| 5 | 7634031 | 7813343 | Production_QTL |
| 5 | 7895867 | 8075179 | Reproduction_QTL |
| 5 | 7895867 | 8075179 | Health_QTL |
| 5 | 7985523 | 19558279 | Meat_Quality_QTL |
| 5 | 7985523 | 44572091 | Health_QTL |
| 5 | 7985523 | 63117217 | Meat_Quality_Association |
| 5 | 15158003 | 15337315 | Meat_Quality_QTL |
| 5 | 17031813 | 78579054 | Meat_Quality_QTL |
| 5 | 17847683 | 18026995 | Exterior_QTL |
| 5 | 18392751 | 18572063 | Meat_Quality_QTL |
| 5 | 18392751 | 1.1E+08 | Health_QTL |
| 5 | 19468623 | 19647935 | Health_QTL |
| 5 | 19558279 | 35377762 | Meat_Quality_QTL |
| 5 | 19558279 | 44572091 | Meat_Quality_QTL |
| 5 | 19558279 | 63117217 | Meat_Quality_Association |
| 5 | 21971859 | 22151171 | Meat_Quality_QTL |
| 5 | 22412792 | 63117217 | Reproduction_QTL |
| 5 | 35377762 | 63117217 | Exterior_QTL |
| 5 | 44572091 | 85756451 | Health_QTL |
| 5 | 45199683 | 55667807 | Reproduction_QTL |
| 5 | 55667807 | 55847119 | Meat_Quality_QTL |
| 5 | 55667807 | 55847119 | Exterior_QTL |
| 5 | 61988652 | 62167964 | Meat_Quality_QTL |
| 5 | 63117217 | 71054179 | Production_QTL |
| 5 | 63117217 | 71907673 | Meat_Quality_QTL |
| 5 | 63117217 | 85756451 | Meat_Quality_QTL |
| 5 | 63117217 | 85756451 | Health_QTL |
| 5 | 63117217 | 85756451 | Meat_Quality_Association |
| 5 | 63834465 | 92844460 | Meat_Quality_QTL |
| 5 | 64731025 | 92844460 | Meat_Quality_QTL |
| 5 | 65495507 | 65674819 | Exterior_QTL |
| 5 | 65585163 | 77055629 | Meat_Quality_QTL |
| 5 | 65986209 | 66165521 | Reproduction_Association |
| 5 | 68231777 | 68411089 | Meat_Quality_QTL |
| 5 | 68590401 | 86316071 | Meat_Quality_QTL |
| 5 | 68769713 | 68949025 | Meat_Quality_QTL |
| 5 | 69171403 | 69350715 | Reproduction_QTL |
| 5 | 69486961 | 69666273 | Health_QTL |
| 5 | 70652489 | 70831801 | Exterior_QTL |
| 5 | 71054179 | 81352956 | Meat_Quality_QTL |
| 5 | 71054179 | 85756451 | Meat_Quality_QTL |
| 5 | 71412803 | 92844460 | Meat_Quality_QTL |
| 5 | 71907673 | 85756451 | Meat_Quality_QTL |
| 5 | 71907673 | 85756451 | Health_QTL |
| 5 | 84846693 | 1.1E+08 | Health_QTL |
| 5 | 85384629 | 86316071 | Meat_Quality_QTL |
| 6 | 119691 | 1.45E+08 | Meat_Quality_QTL |
| 6 | 848555 | 1036570 | Meat_Quality_QTL |
| 6 | 1027170 | 1215185 | Meat_Quality_Association |
| 6 | 1290392 | 1478408 | Meat_Quality_Association |
| 6 | 1318594 | 1506610 | Meat_Quality_Association |
| 6 | 1685225 | 1873241 | Meat_Quality_Association |
| 6 | 1858837 | 2046853 | Meat_Quality_Association |
| 6 | 1868238 | 2056254 | Meat_Quality_Association |
| 6 | 1868238 | 2056254 | Exterior_Association |
| 6 | 2258673 | 2446689 | Reproduction_QTL |
| 6 | 2258673 | 2446689 | Meat_Quality_QTL |
| 6 | 2352681 | 8442460 | Production_QTL |
| 6 | 2352681 | 19536155 | Meat_Quality_QTL |
| 6 | 2352681 | 19536155 | Health_QTL |
| 6 | 2352681 | 29979924 | Meat_Quality_QTL |
| 6 | 2352681 | 29979924 | Production_QTL |
| 6 | 2352681 | 1.3E+08 | Exterior_QTL |
| 6 | 3913775 | 9872228 | Meat_Quality_QTL |
| 6 | 3913775 | 14271713 | Meat_Quality_QTL |
| 6 | 4212104 | 9872228 | Meat_Quality_QTL |
| 6 | 4588136 | 9872228 | Health_QTL |
| 6 | 4588136 | 1.3E+08 | Health_Association |
| 6 | 4682144 | 4870160 | Health_QTL |
| 6 | 8442460 | 14271713 | Production_QTL |
| 6 | 8442460 | 19536155 | Meat_Quality_QTL |
| 6 | 9872228 | 19536155 | Health_QTL |
| 6 | 9872228 | 19536155 | Meat_Quality_QTL |
| 6 | 14271713 | 29979924 | Meat_Quality_QTL |
| 6 | 16452781 | 35210299 | Meat_Quality_QTL |
| 6 | 19536155 | 29979924 | Meat_Quality_QTL |
| 6 | 19536155 | 44290269 | Meat_Quality_QTL |
| 6 | 23378630 | 32048098 | Meat_Quality_QTL |
| 6 | 23942677 | 33430067 | Meat_Quality_QTL |
| 6 | 25634820 | 32048098 | Meat_Quality_QTL |
| 6 | 25634820 | 32048098 | Meat_Quality_Association |
| 6 | 29979924 | 44290269 | Meat_Quality_QTL |
| 6 | 31954090 | 32142106 | Meat_Quality_QTL |
| 6 | 31954090 | 32142106 | Exterior_QTL |
| 6 | 32010495 | 32198510 | Meat_Quality_Association |
| 6 | 32048098 | 44290269 | Meat_Quality_QTL |
| 6 | 32048098 | 1.47E+08 | Meat_Quality_QTL |
| 6 | 32113956 | 32301972 | Meat_Quality_QTL |
| 6 | 32160907 | 32348923 | Meat_Quality_Association |
| 6 | 33618083 | 33806099 | Meat_Quality_QTL |
| 6 | 34464154 | 1.3E+08 | Exterior_QTL |
| 6 | 34464154 | 1.3E+08 | Health_QTL |
| 6 | 34652170 | 34840186 | Health_QTL |
| 6 | 34746178 | 53509801 | Health_QTL |
| 6 | 35216217 | 1.42E+08 | Meat_Quality_QTL |
| 6 | 35492322 | 1.25E+08 | Meat_Quality_QTL |
| 6 | 35586330 | 35774346 | Reproduction_QTL |
| 6 | 35586330 | 35774346 | Meat_Quality_QTL |
| 6 | 35686257 | 69698783 | Meat_Quality_QTL |
| 6 | 35686257 | 89620789 | Meat_Quality_QTL |
| 6 | 35686257 | 1.35E+08 | Meat_Quality_QTL |
| 6 | 36714425 | 1.33E+08 | Production_QTL |
| 6 | 36714425 | 1.39E+08 | Production_QTL |
| 6 | 36714425 | 1.48E+08 | Production_QTL |
| 6 | 36808433 | 36996449 | Exterior_Association |
| 6 | 36902441 | 1.45E+08 | Meat_Quality_QTL |
| 6 | 36980507 | 44478285 | Meat_Quality_QTL |
| 6 | 53603809 | 64103712 | Meat_Quality_QTL |
| 6 | 64291728 | 64479743 | Health_QTL |
| 6 | 74437331 | 1.35E+08 | Meat_Quality_QTL |
| 6 | 74531339 | 1.3E+08 | Reproduction_QTL |
| 6 | 78995338 | 1.25E+08 | Meat_Quality_QTL |
| 6 | 84812063 | 1.35E+08 | Health_QTL |
| 6 | 85846150 | 1.35E+08 | Meat_Quality_QTL |
| 6 | 1.16E+08 | 1.3E+08 | Meat_Quality_QTL |
| 6 | 1.16E+08 | 1.47E+08 | Meat_Quality_QTL |
| 6 | 1.26E+08 | 1.3E+08 | Health_QTL |
| 6 | 1.45E+08 | 1.45E+08 | Meat_Quality_QTL |
| 7 | 2214120 | 2379226 | Meat_Quality_Association |
| 7 | 2214120 | 2379226 | Exterior_Association |
| 7 | 3733086 | 1.28E+08 | Health_QTL |
| 7 | 4790294 | 18621333 | Meat_Quality_QTL |
| 7 | 10634784 | 1.23E+08 | Production_QTL |
| 7 | 11625414 | 24805594 | Meat_Quality_QTL |
| 7 | 11625414 | 24805594 | Meat_Quality_QTL |
| 7 | 11625414 | 25548566 | Meat_Quality_QTL |
| 7 | 11625414 | 45102432 | Meat_Quality_QTL |
| 7 | 11625414 | 45102432 | Production_QTL |
| 7 | 11625414 | 48448805 | Production_QTL |
| 7 | 11625414 | 50310118 | Health_QTL |
| 7 | 11625414 | 55731128 | Meat_Quality_QTL |
| 7 | 11625414 | 55880470 | Exterior_QTL |
| 7 | 11625414 | 1.05E+08 | Meat_Quality_QTL |
| 7 | 11872314 | 64962096 | Meat_Quality_QTL |
| 7 | 11872314 | 99614552 | Meat_Quality_QTL |
| 7 | 12337141 | 18621333 | Meat_Quality_QTL |
| 7 | 12337141 | 24805594 | Meat_Quality_QTL |
| 7 | 12337141 | 25548566 | Meat_Quality_QTL |
| 7 | 12337141 | 32380448 | Meat_Quality_QTL |
| 7 | 12337141 | 45102432 | Meat_Quality_QTL |
| 7 | 12337141 | 48448805 | Meat_Quality_QTL |
| 7 | 12337141 | 50310118 | Meat_Quality_QTL |
| 7 | 12337141 | 1.21E+08 | Meat_Quality_QTL |
| 7 | 13420526 | 1.18E+08 | Meat_Quality_QTL |
| 7 | 15401785 | 50310118 | Meat_Quality_QTL |
| 7 | 17713256 | 1.28E+08 | Meat_Quality_QTL |
| 7 | 18621333 | 24805594 | Meat_Quality_QTL |
| 7 | 18621333 | 25548566 | Production_QTL |
| 7 | 18621333 | 32380448 | Meat_Quality_QTL |
| 7 | 18621333 | 45102432 | Meat_Quality_QTL |
| 7 | 18621333 | 45102432 | Health_QTL |
| 7 | 20565007 | 42873515 | Production_QTL |
| 7 | 20932803 | 32380448 | Meat_Quality_QTL |
| 7 | 21060322 | 99943040 | Meat_Quality_QTL |
| 7 | 21142874 | 24805594 | Meat_Quality_QTL |
| 7 | 21142874 | 45102432 | Meat_Quality_QTL |
| 7 | 21263013 | 42378200 | Exterior_QTL |
| 7 | 21758328 | 60007982 | Production_QTL |
| 7 | 23041582 | 45102432 | Meat_Quality_QTL |
| 7 | 23454344 | 25371016 | Meat_Quality_QTL |
| 7 | 23536897 | 45102432 | Meat_Quality_QTL |
| 7 | 23536897 | 50310118 | Meat_Quality_QTL |
| 7 | 23536897 | 56308996 | Meat_Quality_QTL |
| 7 | 23536897 | 88946228 | Meat_Quality_QTL |
| 7 | 23536897 | 1.21E+08 | Meat_Quality_QTL |
| 7 | 24557936 | 48448805 | Meat_Quality_QTL |
| 7 | 24640488 | 24805594 | Meat_Quality_QTL |
| 7 | 24723041 | 24888146 | Meat_Quality_QTL |
| 7 | 24805594 | 24970698 | Meat_Quality_QTL |
| 7 | 24805594 | 45102432 | Meat_Quality_QTL |
| 7 | 24805594 | 45102432 | Production_QTL |
| 7 | 24805594 | 48448805 | Meat_Quality_QTL |
| 7 | 24805594 | 55483470 | Health_QTL |
| 7 | 24805594 | 64962096 | Exterior_QTL |
| 7 | 24805594 | 64962096 | Production_QTL |
| 7 | 24805594 | 64962096 | Meat_Quality_QTL |
| 7 | 24805594 | 99614552 | Meat_Quality_QTL |
| 7 | 24970698 | 25135803 | Exterior_QTL |
| 7 | 24970698 | 25135803 | Meat_Quality_QTL |
| 7 | 25135803 | 25300908 | Production_QTL |
| 7 | 25383461 | 25548566 | Meat_Quality_QTL |
| 7 | 25453568 | 25618673 | Exterior_QTL |
| 7 | 25453568 | 25618673 | Meat_Quality_QTL |
| 7 | 25548566 | 48448805 | Production_QTL |
| 7 | 25548566 | 1.02E+08 | Health_QTL |
| 7 | 25618673 | 64962096 | Exterior_QTL |
| 7 | 25631118 | 45845405 | Meat_Quality_QTL |
| 7 | 25713671 | 25878776 | Reproduction_QTL |
| 7 | 25796224 | 29873246 | Meat_Quality_QTL |
| 7 | 29873246 | 30038352 | Production_QTL |
| 7 | 30368562 | 86637768 | Reproduction_QTL |
| 7 | 30781324 | 45102432 | Meat_Quality_QTL |
| 7 | 30781324 | 45102432 | Production_QTL |
| 7 | 30781324 | 1.23E+08 | Meat_Quality_QTL |
| 7 | 31967686 | 60007982 | Meat_Quality_QTL |
| 7 | 32215343 | 32380448 | Meat_Quality_QTL |
| 7 | 32380448 | 45102432 | Meat_Quality_QTL |
| 7 | 32710658 | 1.28E+08 | Meat_Quality_QTL |
| 7 | 35765100 | 41552674 | Reproduction_QTL |
| 7 | 35847653 | 41635227 | Meat_Quality_QTL |
| 7 | 41552674 | 41717780 | Health_QTL |
| 7 | 41635227 | 41800332 | Meat_Quality_QTL |
| 7 | 41717780 | 41882885 | Meat_Quality_QTL |
| 7 | 41717780 | 41882885 | Exterior_QTL |
| 7 | 41717780 | 41882885 | Reproduction_QTL |
| 7 | 41717780 | 41882885 | Production_QTL |
| 7 | 42460752 | 42625857 | Health_QTL |
| 7 | 42625857 | 42790962 | Meat_Quality_QTL |
| 7 | 44937327 | 45102432 | Production_QTL |
| 7 | 44937327 | 45102432 | Meat_Quality_QTL |
| 7 | 45102432 | 55483470 | Meat_Quality_QTL |
| 7 | 45102432 | 55880470 | Meat_Quality_QTL |
| 7 | 45102432 | 55880470 | Production_QTL |
| 7 | 45102432 | 1.07E+08 | Meat_Quality_QTL |
| 7 | 45376884 | 49979908 | Meat_Quality_QTL |
| 7 | 48448805 | 99614552 | Meat_Quality_QTL |
| 7 | 49979908 | 50145013 | Meat_Quality_QTL |
| 7 | 50310118 | 1.02E+08 | Meat_Quality_QTL |
| 7 | 50310118 | 1.02E+08 | Meat_Quality_QTL |
| 7 | 50310118 | 1.05E+08 | Production_QTL |
| 7 | 50310118 | 1.21E+08 | Meat_Quality_QTL |
| 7 | 50887986 | 56308996 | Meat_Quality_QTL |
| 7 | 53445812 | 53610917 | Meat_Quality_QTL |
| 7 | 53610917 | 53776022 | Reproduction_QTL |
| 7 | 53693470 | 53858575 | Health_QTL |
| 7 | 54849205 | 55318366 | Meat_Quality_Association |
| 7 | 54931758 | 55400918 | Meat_Quality_QTL |
| 7 | 55302602 | 55467708 | Production_QTL |
| 7 | 55302602 | 99614552 | Health_QTL |
| 7 | 55318366 | 55483470 | Production_QTL |
| 7 | 55385155 | 55550260 | Meat_Quality_QTL |
| 7 | 55400918 | 55566023 | Meat_Quality_QTL |
| 7 | 55467708 | 1.13E+08 | Meat_Quality_QTL |
| 7 | 55483470 | 64962096 | Meat_Quality_QTL |
| 7 | 55632812 | 55797918 | Meat_Quality_QTL |
| 7 | 55648576 | 55813681 | Exterior_QTL |
| 7 | 55731128 | 99225754 | Meat_Quality_QTL |
| 7 | 55880470 | 1.05E+08 | Meat_Quality_QTL |
| 7 | 55880470 | 1.05E+08 | Meat_Quality_QTL |
| 7 | 55880470 | 1.05E+08 | Health_QTL |
| 7 | 55880470 | 1.07E+08 | Meat_Quality_Association |
| 7 | 56128128 | 60007982 | Meat_Quality_QTL |
| 7 | 56226443 | 56391548 | Meat_Quality_QTL |
| 7 | 60255640 | 99614552 | Reproduction_QTL |
| 7 | 64879544 | 65044649 | Exterior_QTL |
| 7 | 64879544 | 65044649 | Meat_Quality_QTL |
| 7 | 64962096 | 1.05E+08 | Meat_Quality_QTL |
| 7 | 64962096 | 1.07E+08 | Meat_Quality_QTL |
| 7 | 64962096 | 1.21E+08 | Exterior_QTL |
| 7 | 71405466 | 1.12E+08 | Meat_Quality_QTL |
| 7 | 71570570 | 71735676 | Production_QTL |
| 7 | 71653123 | 71818228 | Meat_Quality_QTL |
| 7 | 71871352 | 72036456 | Meat_Quality_QTL |
| 7 | 72036456 | 78624440 | Exterior_QTL |
| 7 | 88285808 | 88450913 | Meat_Quality_QTL |
| 7 | 88368360 | 88533466 | Meat_Quality_QTL |
| 7 | 88863676 | 89028780 | Reproduction_QTL |
| 7 | 88946228 | 1.02E+08 | Meat_Quality_QTL |
| 7 | 88946228 | 1.07E+08 | Reproduction_QTL |
| 7 | 99143202 | 99614552 | Meat_Quality_QTL |
| 7 | 99143202 | 99614552 | Meat_Quality_Association |
| 7 | 99225754 | 99697104 | Meat_Quality_QTL |
| 7 | 99225754 | 99697104 | Reproduction_QTL |
| 7 | 99614552 | 1.02E+08 | Meat_Quality_QTL |
| 7 | 99614552 | 1.07E+08 | Health_QTL |
| 7 | 1.02E+08 | 1.05E+08 | Reproduction_QTL |
| 7 | 1.02E+08 | 1.18E+08 | Meat_Quality_QTL |
| 7 | 1.04E+08 | 1.28E+08 | Health_QTL |
| 7 | 1.05E+08 | 1.18E+08 | Production_QTL |
| 7 | 1.05E+08 | 1.21E+08 | Meat_Quality_QTL |
| 7 | 1.05E+08 | 1.21E+08 | Meat_Quality_QTL |
| 7 | 1.05E+08 | 1.28E+08 | Health_QTL |
| 7 | 1.07E+08 | 1.29E+08 | Exterior_QTL |
| 7 | 1.07E+08 | 1.29E+08 | Reproduction_QTL |
| 7 | 1.12E+08 | 1.21E+08 | Meat_Quality_QTL |
| 7 | 1.12E+08 | 1.21E+08 | Exterior_QTL |
| 7 | 1.17E+08 | 1.18E+08 | Meat_Quality_QTL |
| 7 | 1.17E+08 | 1.21E+08 | Meat_Quality_QTL |
| 7 | 1.18E+08 | 1.18E+08 | Production_QTL |
| 7 | 1.18E+08 | 1.18E+08 | Reproduction_QTL |
| 7 | 1.18E+08 | 1.24E+08 | Meat_Quality_QTL |
| 7 | 1.18E+08 | 1.24E+08 | Health_QTL |
| 7 | 1.19E+08 | 1.2E+08 | Meat_Quality_QTL |
| 7 | 1.21E+08 | 1.29E+08 | Production_QTL |
| 7 | 1.21E+08 | 1.29E+08 | Meat_Quality_QTL |
| 7 | 1.21E+08 | 1.29E+08 | Meat_Quality_QTL |
| 7 | 1.21E+08 | 1.29E+08 | Meat_Quality_Association |
| 7 | 1.23E+08 | 1.23E+08 | Meat_Quality_Association |
| 7 | 1.24E+08 | 1.24E+08 | Production_QTL |
| 7 | 1.24E+08 | 1.29E+08 | Exterior_QTL |
| 7 | 1.27E+08 | 1.28E+08 | Production_QTL |
| 7 | 1.27E+08 | 1.28E+08 | Meat_Quality_QTL |
| 7 | 1.28E+08 | 1.28E+08 | Meat_Quality_QTL |
| 7 | 1.29E+08 | 1.29E+08 | Health_QTL |
| 7 | 1.3E+08 | 1.3E+08 | Reproduction_QTL |
| 7 | 1.3E+08 | 1.3E+08 | Meat_Quality_QTL |
| 8 | 1972820 | 6562304 | Reproduction_Association |
| 8 | 4663629 | 15636344 | Reproduction_QTL |
| 8 | 6063313 | 9960778 | Health_Association |
| 8 | 6063313 | 26534249 | Health_QTL |
| 8 | 6651169 | 19749801 | Meat_Quality_QTL |
| 8 | 9853317 | 10074155 | Meat_Quality_QTL |
| 8 | 10623291 | 10844129 | Meat_Quality_QTL |
| 8 | 10626249 | 99338918 | Meat_Quality_QTL |
| 8 | 10733710 | 10954548 | Meat_Quality_QTL |
| 8 | 10844129 | 15636344 | Production_QTL |
| 8 | 10844129 | 19749801 | Meat_Quality_QTL |
| 8 | 10844129 | 26534249 | Production_QTL |
| 8 | 10844129 | 26534249 | Meat_Quality_QTL |
| 8 | 10844129 | 29471436 | Production_QTL |
| 8 | 10844129 | 29471436 | Meat_Quality_QTL |
| 8 | 10844129 | 41095628 | Meat_Quality_QTL |
| 8 | 10844129 | 1.48E+08 | Health_QTL |
| 8 | 10847087 | 11067925 | Meat_Quality_QTL |
| 8 | 11067925 | 15746763 | Meat_Quality_QTL |
| 8 | 11308413 | 18349963 | Meat_Quality_QTL |
| 8 | 11308413 | 26534249 | Exterior_QTL |
| 8 | 15448482 | 26534249 | Reproduction_QTL |
| 8 | 19639382 | 19860220 | Exterior_QTL |
| 8 | 19674990 | 19895828 | Meat_Quality_QTL |
| 8 | 19749801 | 46427317 | Meat_Quality_QTL |
| 8 | 19749801 | 46427317 | Health_QTL |
| 8 | 19896638 | 46427317 | Meat_Quality_QTL |
| 8 | 26202992 | 1.16E+08 | Meat_Quality_QTL |
| 8 | 46316898 | 46537736 | Health_QTL |
| 8 | 46427317 | 92419438 | Health_QTL |
| 8 | 46537736 | 46758574 | Exterior_QTL |
| 8 | 46648155 | 46868993 | Meat_Quality_QTL |
| 8 | 52276421 | 52497259 | Meat_Quality_QTL |
| 8 | 52497259 | 52718097 | Exterior_QTL |
| 8 | 52718097 | 1.34E+08 | Reproduction_QTL |
| 8 | 72039691 | 92419438 | Meat_Quality_QTL |
| 8 | 92419438 | 1.34E+08 | Health_QTL |
| 8 | 1.1E+08 | 1.37E+08 | Exterior_QTL |
| 8 | 1.12E+08 | 1.18E+08 | Reproduction_QTL |
| 8 | 1.23E+08 | 1.34E+08 | Reproduction_QTL |
| 8 | 1.39E+08 | 1.48E+08 | Reproduction_QTL |
| 8 | 1.48E+08 | 1.48E+08 | Health_QTL |
| 8 | 1.48E+08 | 1.48E+08 | Reproduction_QTL |
| 9 | 0 | 197465 | Exterior_QTL |
| 9 | 0 | 197465 | Production_QTL |
| 9 | 0 | 197465 | Meat_Quality_Association |
| 9 | 88508 | 11066889 | Meat_Quality_QTL |
| 9 | 88508 | 11066889 | Production_QTL |
| 9 | 88508 | 23144816 | Meat_Quality_QTL |
| 9 | 88508 | 1.23E+08 | Meat_Quality_QTL |
| 9 | 88508 | 1.41E+08 | Meat_Quality_QTL |
| 9 | 88508 | 1.47E+08 | Meat_Quality_QTL |
| 9 | 11066889 | 23144816 | Production_QTL |
| 9 | 11066889 | 23144816 | Exterior_QTL |
| 9 | 11066889 | 23144816 | Reproduction_QTL |
| 9 | 11066889 | 45282513 | Meat_Quality_QTL |
| 9 | 11066889 | 48711260 | Meat_Quality_QTL |
| 9 | 18659348 | 23144816 | Meat_Quality_QTL |
| 9 | 18659348 | 23144816 | Meat_Quality_QTL |
| 9 | 23144816 | 37993834 | Production_QTL |
| 9 | 23144816 | 45282513 | Meat_Quality_QTL |
| 9 | 37340093 | 37558007 | Exterior_QTL |
| 9 | 37884861 | 38102775 | Meat_Quality_QTL |
| 9 | 37993834 | 48711260 | Production_QTL |
| 9 | 44612072 | 44829985 | Meat_Quality_QTL |
| 9 | 45064599 | 45282513 | Health_QTL |
| 9 | 45173556 | 1.39E+08 | Reproduction_QTL |
| 9 | 45282513 | 56664370 | Meat_Quality_QTL |
| 9 | 45282513 | 63050049 | Meat_Quality_QTL |
| 9 | 45282513 | 67062987 | Meat_Quality_QTL |
| 9 | 45282513 | 1.1E+08 | Meat_Quality_QTL |
| 9 | 45282513 | 1.1E+08 | Meat_Quality_QTL |
| 9 | 45282513 | 1.29E+08 | Meat_Quality_QTL |
| 9 | 45282513 | 1.4E+08 | Production_QTL |
| 9 | 53311036 | 1.29E+08 | Health_QTL |
| 9 | 65059079 | 67062987 | Meat_Quality_QTL |
| 9 | 71892071 | 72109985 | Production_Association |
| 9 | 71892071 | 72109985 | Meat_Quality_Association |
| 9 | 1.25E+08 | 1.4E+08 | Meat_Quality_QTL |
| 9 | 1.25E+08 | 1.4E+08 | Health_Association |
| 9 | 1.27E+08 | 1.4E+08 | Meat_Quality_QTL |
| 9 | 1.29E+08 | 1.4E+08 | Meat_Quality_QTL |
| 9 | 1.29E+08 | 1.4E+08 | Health_QTL |
| 10 | 2121522 | 10227060 | Meat_Quality_QTL |
| 10 | 2121522 | 20005898 | Production_QTL |
| 10 | 2121522 | 20005898 | Meat_Quality_QTL |
| 10 | 2121522 | 32088890 | Production_QTL |
| 10 | 2121522 | 32088890 | Production_QTL |
| 10 | 2121522 | 46049434 | Meat_Quality_QTL |
| 10 | 2121522 | 58907094 | Health_QTL |
| 10 | 3140391 | 3249947 | Exterior_Association |
| 10 | 4476972 | 11093314 | Meat_Quality_QTL |
| 10 | 4476972 | 32088890 | Health_Association |
| 10 | 6401430 | 10227060 | Meat_Quality_QTL |
| 10 | 6401430 | 11093314 | Meat_Quality_QTL |
| 10 | 11093314 | 20005898 | Meat_Quality_QTL |
| 10 | 11093314 | 27889269 | Health_QTL |
| 10 | 12627095 | 20005898 | Meat_Quality_QTL |
| 10 | 28168636 | 32088890 | Meat_Quality_QTL |
| 10 | 32088890 | 58907094 | Production_QTL |
| 10 | 32088890 | 58907094 | Meat_Quality_QTL |
| 10 | 41334738 | 58907094 | Meat_Quality_QTL |
| 10 | 41334738 | 58907094 | Health_QTL |
| 10 | 41334738 | 58907094 | Exterior_QTL |
| 10 | 46980658 | 58907094 | Meat_Quality_QTL |
| 10 | 58062668 | 58907094 | Meat_Quality_QTL |
| 11 | 3920148 | 31594979 | Meat_Quality_QTL |
| 11 | 3920148 | 31594979 | Reproduction_QTL |
| 11 | 3920148 | 41670839 | Health_QTL |
| 11 | 3920148 | 41897196 | Exterior_QTL |
| 11 | 3920148 | 48885503 | Meat_Quality_QTL |
| 11 | 3920148 | 52966278 | Exterior_QTL |
| 11 | 4882972 | 31597602 | Meat_Quality_QTL |
| 11 | 7902465 | 41897196 | Meat_Quality_QTL |
| 11 | 7902465 | 48885503 | Meat_Quality_QTL |
| 11 | 9315926 | 9508491 | Meat_Quality_QTL |
| 11 | 16627299 | 16819864 | Meat_Quality_QTL |
| 11 | 18360382 | 28899072 | Meat_Quality_QTL |
| 11 | 18360382 | 41897196 | Health_QTL |
| 11 | 18360382 | 43692769 | Health_QTL |
| 11 | 18695760 | 41897196 | Meat_Quality_QTL |
| 11 | 18695760 | 78901241 | Meat_Quality_QTL |
| 11 | 23028468 | 23221033 | Meat_Quality_QTL |
| 11 | 28899072 | 31597602 | Meat_Quality_QTL |
| 11 | 29671954 | 52966278 | Health_QTL |
| 11 | 30535873 | 30728438 | Meat_Quality_QTL |
| 11 | 31116190 | 31308754 | Meat_Quality_QTL |
| 11 | 31594979 | 41897196 | Exterior_QTL |
| 11 | 31594979 | 78227264 | Health_QTL |
| 11 | 31594979 | 78227264 | Exterior_QTL |
| 11 | 33520627 | 33713192 | Production_QTL |
| 11 | 34098322 | 34290886 | Health_QTL |
| 11 | 35253710 | 35446275 | Meat_Quality_QTL |
| 11 | 36216534 | 51003715 | Reproduction_QTL |
| 11 | 39490136 | 39682701 | Health_QTL |
| 11 | 40549242 | 40741807 | Meat_Quality_QTL |
| 11 | 40645525 | 40838090 | Production_QTL |
| 11 | 40645525 | 40838090 | Meat_Quality_QTL |
| 11 | 41897196 | 52388584 | Meat_Quality_QTL |
| 11 | 41897196 | 52388584 | Production_QTL |
| 11 | 41897196 | 75366022 | Meat_Quality_QTL |
| 11 | 41897196 | 75366022 | Meat_Quality_QTL |
| 11 | 41897196 | 79769559 | Reproduction_QTL |
| 11 | 41959686 | 52388584 | Health_QTL |
| 11 | 43692769 | 78901241 | Meat_Quality_QTL |
| 11 | 43789052 | 43981616 | Meat_Quality_QTL |
| 11 | 50040891 | 50233456 | Reproduction_QTL |
| 11 | 51655482 | 51848047 | Production_QTL |
| 11 | 51764346 | 81069519 | Meat_Quality_QTL |
| 11 | 51848047 | 52040612 | Meat_Quality_QTL |
| 11 | 52062822 | 52166230 | Meat_Quality_QTL |
| 11 | 52388584 | 78227264 | Meat_Quality_QTL |
| 11 | 52388584 | 81069519 | Reproduction_QTL |
| 11 | 52522024 | 52714589 | Meat_Quality_QTL |
| 11 | 52840207 | 53032772 | Production_QTL |
| 11 | 54245356 | 75366022 | Production_QTL |
| 11 | 54245356 | 75366022 | Meat_Quality_QTL |
| 11 | 58224045 | 58416610 | Health_QTL |
| 12 | 2999557 | 4823589 | Meat_Quality_QTL |
| 12 | 2999557 | 8723537 | Meat_Quality_QTL |
| 12 | 2999557 | 18253452 | Exterior_QTL |
| 12 | 2999557 | 34985025 | Health_QTL |
| 12 | 3812832 | 3909094 | Meat_Quality_Association |
| 12 | 3894799 | 3991062 | Meat_Quality_Association |
| 12 | 3894799 | 3991062 | Exterior_Association |
| 12 | 4246013 | 4342276 | Meat_Quality_QTL |
| 12 | 4250971 | 63588571 | Reproduction_QTL |
| 12 | 4775458 | 4871720 | Production_QTL |
| 12 | 4775458 | 4871720 | Health_QTL |
| 12 | 4775458 | 4871720 | Reproduction_QTL |
| 12 | 4823589 | 4919852 | Meat_Quality_QTL |
| 12 | 4823589 | 7135204 | Meat_Quality_QTL |
| 12 | 4823589 | 8723537 | Meat_Quality_QTL |
| 12 | 4823589 | 13953416 | Meat_Quality_QTL |
| 12 | 4823589 | 23513990 | Reproduction_Association |
| 12 | 4823589 | 23672762 | Health_QTL |
| 12 | 6411922 | 6508184 | Exterior_QTL |
| 12 | 6942679 | 7807730 | Production_QTL |
| 12 | 7807730 | 44090840 | Meat_Quality_QTL |
| 12 | 8001568 | 8097830 | Meat_Quality_QTL |
| 12 | 8675406 | 8771668 | Reproduction_QTL |
| 12 | 8723537 | 16456243 | Meat_Quality_QTL |
| 12 | 8723537 | 16456243 | Meat_Quality_QTL |
| 12 | 8723537 | 23672762 | Reproduction_QTL |
| 12 | 9445507 | 47013108 | Meat_Quality_QTL |
| 12 | 10745052 | 10841314 | Production_QTL |
| 12 | 11130102 | 63588571 | Reproduction_QTL |
| 12 | 11274496 | 11370759 | Meat_Quality_QTL |
| 12 | 11274496 | 58350625 | Health_QTL |
| 12 | 11539218 | 11876137 | Reproduction_QTL |
| 12 | 11996466 | 12092728 | Health_QTL |
| 12 | 12188991 | 12285253 | Meat_Quality_QTL |
| 12 | 12439274 | 12602920 | Reproduction_QTL |
| 12 | 12528729 | 12740507 | Reproduction_QTL |
| 12 | 12653870 | 12750133 | Health_QTL |
| 12 | 12670304 | 12766566 | Production_QTL |
| 12 | 12814698 | 12910960 | Production_QTL |
| 12 | 12894527 | 12990790 | Health_QTL |
| 12 | 12894527 | 15638011 | Meat_Quality_QTL |
| 12 | 12910960 | 13007223 | Meat_Quality_QTL |
| 12 | 13905284 | 14001547 | Exterior_QTL |
| 12 | 13953416 | 14049678 | Meat_Quality_QTL |
| 12 | 13953416 | 17226344 | Health_QTL |
| 12 | 13953416 | 23672762 | Reproduction_QTL |
| 12 | 13953416 | 23672762 | Health_QTL |
| 12 | 13953416 | 47927603 | Exterior_QTL |
| 12 | 14306768 | 15297222 | Meat_Quality_QTL |
| 12 | 14671516 | 14767778 | Production_QTL |
| 12 | 14884344 | 14980606 | Health_QTL |
| 12 | 15056566 | 15152829 | Production_QTL |
| 12 | 15060436 | 15156698 | Reproduction_QTL |
| 12 | 15252961 | 15349223 | Meat_Quality_Association |
| 12 | 15489748 | 15586010 | Health_QTL |
| 12 | 15830536 | 15926799 | Health_QTL |
| 12 | 15926799 | 16023062 | Meat_Quality_QTL |
| 12 | 15971061 | 16067323 | Reproduction_QTL |
| 12 | 16408112 | 16504374 | Health_QTL |
| 12 | 16456243 | 23672762 | Meat_Quality_QTL |
| 12 | 16937556 | 21973788 | Meat_Quality_QTL |
| 12 | 17081950 | 17178213 | Meat_Quality_QTL |
| 12 | 17226344 | 23672762 | Meat_Quality_QTL |
| 12 | 17226344 | 23672762 | Reproduction_QTL |
| 12 | 18729952 | 58124464 | Meat_Quality_QTL |
| 12 | 19745522 | 19841785 | Meat_Quality_QTL |
| 12 | 20226835 | 20323098 | Meat_Quality_QTL |
| 12 | 20611886 | 20708148 | Meat_Quality_QTL |
| 12 | 21718906 | 53930665 | Meat_Quality_QTL |
| 12 | 22743889 | 22840152 | Meat_Quality_Association |
| 12 | 22743889 | 22840152 | Meat_Quality_QTL |
| 12 | 23465859 | 23562121 | Exterior_QTL |
| 12 | 23610253 | 57980070 | Meat_Quality_QTL |
| 12 | 23624631 | 23720893 | Exterior_QTL |
| 12 | 23672762 | 26242726 | Reproduction_QTL |
| 12 | 23672762 | 38822400 | Meat_Quality_QTL |
| 12 | 23672762 | 38822400 | Reproduction_QTL |
| 12 | 23672762 | 40771258 | Meat_Quality_QTL |
| 12 | 23672762 | 47927603 | Exterior_QTL |
| 12 | 23672762 | 58124464 | Production_QTL |
| 12 | 23672762 | 58124464 | Meat_Quality_QTL |
| 12 | 23672762 | 58124464 | Meat_Quality_Association |
| 12 | 24480596 | 24576858 | Meat_Quality_QTL |
| 12 | 25106303 | 25202565 | Reproduction_QTL |
| 12 | 26242726 | 38822400 | Meat_Quality_QTL |
| 12 | 26242726 | 41830147 | Health_QTL |
| 12 | 26290857 | 26387120 | Meat_Quality_QTL |
| 12 | 35755126 | 35851388 | Meat_Quality_QTL |
| 12 | 35995782 | 36092045 | Meat_Quality_QTL |
| 12 | 38822400 | 43272608 | Production_QTL |
| 12 | 38822400 | 47927603 | Meat_Quality_QTL |
| 12 | 38822400 | 47927603 | Meat_Quality_QTL |
| 12 | 38822400 | 47927603 | Health_QTL |
| 12 | 38822400 | 57436130 | Exterior_QTL |
| 12 | 39159319 | 39255582 | Meat_Quality_QTL |
| 12 | 40771258 | 54315716 | Meat_Quality_QTL |
| 12 | 41396965 | 41493228 | Reproduction_QTL |
| 12 | 41830147 | 47927603 | Meat_Quality_QTL |
| 12 | 41830147 | 47927603 | Health_QTL |
| 12 | 43609527 | 47927603 | Meat_Quality_QTL |
| 12 | 43657658 | 57387999 | Health_QTL |
| 12 | 44235234 | 44331497 | Reproduction_QTL |
| 12 | 44524022 | 44620284 | Meat_Quality_QTL |
| 12 | 46579927 | 57387999 | Meat_Quality_QTL |
| 12 | 46676189 | 46772452 | Reproduction_QTL |
| 12 | 47590684 | 47686946 | Health_QTL |
| 12 | 47735078 | 47831340 | Meat_Quality_QTL |
| 12 | 47783209 | 47879472 | Health_QTL |
| 12 | 47879472 | 47975734 | Health_QTL |
| 12 | 47927603 | 56728656 | Meat_Quality_QTL |
| 12 | 47927603 | 57436130 | Health_QTL |
| 12 | 47927603 | 57436130 | Meat_Quality_QTL |
| 12 | 48360785 | 48457047 | Reproduction_Association |
| 12 | 54508241 | 55482035 | Health_QTL |
| 12 | 57387999 | 57484261 | Meat_Quality_QTL |
| 12 | 57917443 | 58013706 | Reproduction_QTL |
| 12 | 61719816 | 61816078 | Production_QTL |
| 12 | 63587571 | 63588571 | Health_QTL |
| 13 | 3278794 | 2.15E+08 | Health_QTL |
| 13 | 9843648 | 22581169 | Reproduction_QTL |
| 13 | 18707782 | 1.31E+08 | Meat_Quality_QTL |
| 13 | 18707782 | 1.31E+08 | Meat_Quality_QTL |
| 13 | 23000486 | 23337302 | Health_QTL |
| 13 | 23674118 | 24010934 | Meat_Quality_Association |
| 13 | 24516158 | 24852975 | Meat_Quality_QTL |
| 13 | 27210688 | 1.95E+08 | Reproduction_QTL |
| 13 | 32577445 | 1.31E+08 | Meat_Quality_QTL |
| 13 | 39145361 | 1.31E+08 | Meat_Quality_QTL |
| 13 | 54380425 | 54717242 | Meat_Quality_QTL |
| 13 | 58927444 | 79114433 | Meat_Quality_QTL |
| 13 | 60443117 | 60779933 | Meat_Quality_QTL |
| 13 | 75056893 | 75393709 | Exterior_QTL |
| 13 | 75113053 | 75449869 | Reproduction_QTL |
| 13 | 75225301 | 75562117 | Reproduction_QTL |
| 13 | 75281461 | 1.95E+08 | Meat_Quality_QTL |
| 13 | 75393709 | 75730525 | Meat_Quality_QTL |
| 13 | 75955093 | 1.28E+08 | Production_QTL |
| 13 | 76744778 | 77081594 | Health_QTL |
| 13 | 79114433 | 1.31E+08 | Production_QTL |
| 13 | 79114433 | 1.31E+08 | Meat_Quality_QTL |
| 13 | 79788065 | 80124881 | Production_QTL |
| 13 | 89503549 | 1.95E+08 | Meat_Quality_QTL |
| 13 | 1.3E+08 | 1.31E+08 | Meat_Quality_QTL |
| 13 | 1.31E+08 | 1.95E+08 | Meat_Quality_QTL |
| 13 | 1.31E+08 | 1.95E+08 | Production_QTL |
| 13 | 1.31E+08 | 1.95E+08 | Exterior_QTL |
| 13 | 1.59E+08 | 1.95E+08 | Meat_Quality_QTL |
| 13 | 1.71E+08 | 1.71E+08 | Health_QTL |
| 13 | 1.84E+08 | 1.84E+08 | Health_QTL |
| 13 | 1.84E+08 | 1.85E+08 | Meat_Quality_QTL |
| 13 | 1.84E+08 | 1.85E+08 | Health_QTL |
| 13 | 1.85E+08 | 1.86E+08 | Exterior_QTL |
| 13 | 1.86E+08 | 1.86E+08 | Meat_Quality_QTL |
| 13 | 1.95E+08 | 1.95E+08 | Health_QTL |
| 13 | 2.16E+08 | 2.16E+08 | Meat_Quality_QTL |
| 13 | 2.16E+08 | 2.16E+08 | Production_Association |
| 13 | 2.16E+08 | 2.16E+08 | Meat_Quality_Association |
| 14 | 6757193 | 7039507 | Meat_Quality_QTL |
| 14 | 6757193 | 7039507 | Health_QTL |
| 14 | 6898350 | 16066507 | Meat_Quality_QTL |
| 14 | 6898350 | 16066507 | Meat_Quality_QTL |
| 14 | 6898350 | 41217524 | Meat_Quality_QTL |
| 14 | 6898350 | 41487784 | Meat_Quality_QTL |
| 14 | 6898350 | 66305680 | Production_QTL |
| 14 | 6898350 | 1.32E+08 | Meat_Quality_QTL |
| 14 | 6898350 | 1.32E+08 | Exterior_QTL |
| 14 | 6898350 | 1.32E+08 | Health_QTL |
| 14 | 6898350 | 1.33E+08 | Meat_Quality_QTL |
| 14 | 8528044 | 1.43E+08 | Meat_Quality_QTL |
| 14 | 8528044 | 1.5E+08 | Meat_Quality_QTL |
| 14 | 9681757 | 60644503 | Health_QTL |
| 14 | 9757277 | 1.39E+08 | Meat_Quality_QTL |
| 14 | 10962367 | 1.54E+08 | Production_QTL |
| 14 | 13210690 | 13493004 | Meat_Quality_QTL |
| 14 | 13351847 | 93372131 | Health_QTL |
| 14 | 15219563 | 15501878 | Meat_Quality_QTL |
| 14 | 16066507 | 24169455 | Meat_Quality_QTL |
| 14 | 16066507 | 27887165 | Meat_Quality_QTL |
| 14 | 16066507 | 41217524 | Meat_Quality_QTL |
| 14 | 16066507 | 41217524 | Meat_Quality_QTL |
| 14 | 16066507 | 1.32E+08 | Exterior_QTL |
| 14 | 18836994 | 19119309 | Meat_Quality_QTL |
| 14 | 19260466 | 29711236 | Meat_Quality_QTL |
| 14 | 19260466 | 60644503 | Meat_Quality_QTL |
| 14 | 19260466 | 65129650 | Meat_Quality_QTL |
| 14 | 19260466 | 81745465 | Reproduction_QTL |
| 14 | 19401623 | 1.32E+08 | Meat_Quality_QTL |
| 14 | 20248567 | 20530882 | Meat_Quality_QTL |
| 14 | 21518983 | 23604826 | Meat_Quality_QTL |
| 14 | 21628623 | 21910938 | Meat_Quality_QTL |
| 14 | 27531879 | 48541571 | Meat_Quality_QTL |
| 14 | 27531879 | 60644503 | Health_QTL |
| 14 | 27531879 | 81745465 | Meat_Quality_QTL |
| 14 | 27604850 | 60644503 | Meat_Quality_QTL |
| 14 | 27604850 | 66305680 | Meat_Quality_QTL |
| 14 | 29570079 | 29852393 | Meat_Quality_QTL |
| 14 | 29838278 | 29993551 | Reproduction_QTL |
| 14 | 32915907 | 1.32E+08 | Production_QTL |
| 14 | 33895930 | 34178244 | Meat_Quality_QTL |
| 14 | 34460559 | 39736403 | Health_QTL |
| 14 | 41217524 | 81745465 | Meat_Quality_QTL |
| 14 | 41217524 | 81745465 | Exterior_QTL |
| 14 | 48216747 | 61209132 | Production_QTL |
| 14 | 48216747 | 1.53E+08 | Meat_Quality_QTL |
| 14 | 48259257 | 48541571 | Meat_Quality_QTL |
| 14 | 53465935 | 81745465 | Health_Association |
| 14 | 62620705 | 63012290 | Meat_Quality_QTL |
| 14 | 81604308 | 81886622 | Meat_Quality_Association |
| 14 | 81745465 | 1.26E+08 | Production_QTL |
| 14 | 81745465 | 1.32E+08 | Meat_Quality_QTL |
| 14 | 81745465 | 1.32E+08 | Meat_Quality_QTL |
| 14 | 81745465 | 1.32E+08 | Production_QTL |
| 14 | 81745465 | 1.32E+08 | Meat_Quality_QTL |
| 14 | 81745465 | 1.4E+08 | Meat_Quality_QTL |
| 14 | 81745465 | 1.41E+08 | Health_Association |
| 14 | 82027780 | 83308619 | Production_QTL |
| 14 | 83732091 | 84014405 | Meat_Quality_QTL |
| 14 | 83873248 | 93372131 | Health_Association |
| 14 | 85849450 | 1.11E+08 | Meat_Quality_QTL |
| 14 | 90000278 | 90282593 | Exterior_QTL |
| 14 | 90282593 | 1.3E+08 | Meat_Quality_QTL |
| 14 | 90972457 | 91254772 | Meat_Quality_QTL |
| 14 | 90972457 | 91254772 | Exterior_QTL |
| 14 | 93230974 | 93513288 | Health_QTL |
| 14 | 93372131 | 1.32E+08 | Health_QTL |
| 14 | 93372131 | 1.38E+08 | Health_Association |
| 14 | 93372131 | 1.38E+08 | Health_QTL |
| 14 | 93654446 | 93936760 | Exterior_QTL |
| 14 | 1.02E+08 | 1.35E+08 | Meat_Quality_QTL |
| 14 | 1.02E+08 | 1.03E+08 | Reproduction_QTL |
| 14 | 1.02E+08 | 1.15E+08 | Meat_Quality_QTL |
| 14 | 1.11E+08 | 1.12E+08 | Meat_Quality_QTL |
| 14 | 1.25E+08 | 1.25E+08 | Production_QTL |
| 14 | 1.26E+08 | 1.38E+08 | Meat_Quality_QTL |
| 14 | 1.32E+08 | 1.32E+08 | Reproduction_QTL |
| 14 | 1.32E+08 | 1.32E+08 | Meat_Quality_QTL |
| 14 | 1.32E+08 | 1.32E+08 | Health_QTL |
| 14 | 1.32E+08 | 1.38E+08 | Meat_Quality_QTL |
| 14 | 1.32E+08 | 1.43E+08 | Meat_Quality_QTL |
| 14 | 1.32E+08 | 1.54E+08 | Meat_Quality_Association |
| 14 | 1.32E+08 | 1.5E+08 | Meat_Quality_QTL |
| 14 | 1.32E+08 | 1.32E+08 | Production_QTL |
| 14 | 1.33E+08 | 1.33E+08 | Production_QTL |
| 14 | 1.34E+08 | 1.43E+08 | Meat_Quality_QTL |
| 14 | 1.35E+08 | 1.43E+08 | Meat_Quality_QTL |
| 14 | 1.37E+08 | 1.37E+08 | Meat_Quality_QTL |
| 14 | 1.38E+08 | 1.38E+08 | Health_QTL |
| 14 | 1.38E+08 | 1.43E+08 | Meat_Quality_QTL |
| 14 | 1.38E+08 | 1.5E+08 | Health_QTL |
| 14 | 1.38E+08 | 1.39E+08 | Exterior_QTL |
| 14 | 1.39E+08 | 1.39E+08 | Reproduction_Association |
| 14 | 1.4E+08 | 1.41E+08 | Production_QTL |
| 14 | 1.41E+08 | 1.41E+08 | Exterior_QTL |
| 14 | 1.42E+08 | 1.42E+08 | Production_QTL |
| 14 | 1.43E+08 | 1.43E+08 | Reproduction_QTL |
| 14 | 1.43E+08 | 1.5E+08 | Meat_Quality_QTL |
| 14 | 1.46E+08 | 1.54E+08 | Meat_Quality_QTL |
| 14 | 1.47E+08 | 1.48E+08 | Health_QTL |
| 14 | 1.48E+08 | 1.54E+08 | Meat_Quality_QTL |
| 14 | 1.49E+08 | 1.49E+08 | Meat_Quality_QTL |
| 14 | 1.5E+08 | 1.5E+08 | Reproduction_QTL |
| 14 | 1.54E+08 | 1.54E+08 | Production_QTL |
| 14 | 1.54E+08 | 1.54E+08 | Reproduction_QTL |
| 14 | 1.54E+08 | 1.54E+08 | Health_QTL |
| 15 | 2752020 | 21177432 | Meat_Quality_QTL |
| 15 | 8000145 | 25021683 | Meat_Quality_QTL |
| 15 | 8000145 | 1.46E+08 | Exterior_QTL |
| 15 | 20610304 | 43065120 | Meat_Quality_QTL |
| 15 | 21177432 | 43065120 | Meat_Quality_QTL |
| 15 | 25021683 | 38976106 | Meat_Quality_QTL |
| 15 | 25021683 | 54487177 | Meat_Quality_QTL |
| 15 | 25021683 | 57165536 | Meat_Quality_QTL |
| 15 | 25021683 | 71973307 | Health_QTL |
| 15 | 25021683 | 1.13E+08 | Exterior_QTL |
| 15 | 26508597 | 26756416 | Meat_Quality_QTL |
| 15 | 31896907 | 1.49E+08 | Meat_Quality_QTL |
| 15 | 33137272 | 1.46E+08 | Reproduction_QTL |
| 15 | 33137272 | 1.46E+08 | Meat_Quality_Association |
| 15 | 37241374 | 1.49E+08 | Health_QTL |
| 15 | 38976106 | 43065120 | Meat_Quality_QTL |
| 15 | 42185363 | 1.46E+08 | Meat_Quality_QTL |
| 15 | 42941210 | 43189029 | Meat_Quality_Association |
| 15 | 43065120 | 99227812 | Meat_Quality_QTL |
| 15 | 43065120 | 1.06E+08 | Meat_Quality_QTL |
| 15 | 43065120 | 1.41E+08 | Meat_Quality_QTL |
| 15 | 43560758 | 1.14E+08 | Meat_Quality_QTL |
| 15 | 45915038 | 1.4E+08 | Reproduction_QTL |
| 15 | 53136564 | 1.46E+08 | Meat_Quality_QTL |
| 15 | 61488719 | 61736538 | Production_QTL |
| 15 | 61612628 | 61860448 | Production_QTL |
| 15 | 66799112 | 99227812 | Meat_Quality_QTL |
| 15 | 71849398 | 1.15E+08 | Meat_Quality_QTL |
| 15 | 71973307 | 1.14E+08 | Meat_Quality_QTL |
| 15 | 71973307 | 1.28E+08 | Meat_Quality_QTL |
| 15 | 79844324 | 1.15E+08 | Meat_Quality_QTL |
| 15 | 88544663 | 1.41E+08 | Meat_Quality_QTL |
| 15 | 99227812 | 1.28E+08 | Meat_Quality_QTL |
| 15 | 1.06E+08 | 1.39E+08 | Meat_Quality_QTL |
| 15 | 1.06E+08 | 1.06E+08 | Meat_Quality_QTL |
| 15 | 1.13E+08 | 1.41E+08 | Health_QTL |
| 15 | 1.14E+08 | 1.28E+08 | Reproduction_QTL |
| 15 | 1.28E+08 | 1.39E+08 | Meat_Quality_QTL |
| 15 | 1.28E+08 | 1.41E+08 | Meat_Quality_QTL |
| 15 | 1.28E+08 | 1.41E+08 | Exterior_QTL |
| 15 | 1.35E+08 | 1.41E+08 | Meat_Quality_QTL |
| 15 | 1.35E+08 | 1.46E+08 | Meat_Quality_QTL |
| 15 | 1.35E+08 | 1.46E+08 | Production_QTL |
| 15 | 1.4E+08 | 1.46E+08 | Meat_Quality_QTL |
| 15 | 1.41E+08 | 1.46E+08 | Meat_Quality_QTL |
| 15 | 1.45E+08 | 1.46E+08 | Health_QTL |
| 15 | 1.49E+08 | 1.49E+08 | Reproduction_QTL |
| 16 | 1077318 | 1258336 | Meat_Quality_QTL |
| 16 | 1167827 | 18970761 | Meat_Quality_QTL |
| 16 | 1167827 | 20168075 | Meat_Quality_QTL |
| 16 | 1167827 | 20982652 | Meat_Quality_QTL |
| 16 | 1167827 | 39957561 | Production_QTL |
| 16 | 1167827 | 42857275 | Reproduction_QTL |
| 16 | 1167827 | 66781192 | Production_QTL |
| 16 | 1167827 | 67324243 | Reproduction_QTL |
| 16 | 1167827 | 67649164 | Exterior_QTL |
| 16 | 1167827 | 67649164 | Health_QTL |
| 16 | 1167827 | 67649164 | Health_QTL |
| 16 | 1167827 | 68644759 | Meat_Quality_QTL |
| 16 | 1167827 | 71940182 | Meat_Quality_QTL |
| 16 | 1167827 | 77499099 | Meat_Quality_QTL |
| 16 | 1167827 | 79904939 | Meat_Quality_QTL |
| 16 | 1167827 | 80266973 | Meat_Quality_QTL |
| 16 | 1167827 | 82348671 | Meat_Quality_QTL |
| 16 | 6890949 | 18970761 | Health_QTL |
| 16 | 24826912 | 48492012 | Reproduction_QTL |
| 16 | 25953165 | 67649164 | Meat_Quality_QTL |
| 16 | 48492012 | 58970415 | Meat_Quality_QTL |
| 16 | 58970415 | 67649164 | Meat_Quality_QTL |
| 16 | 67649164 | 80266973 | Meat_Quality_QTL |
| 16 | 71797057 | 80266973 | Meat_Quality_QTL |
| 16 | 71797057 | 80266973 | Production_QTL |
| 17 | 4378268 | 4514039 | Meat_Quality_Association |
| 17 | 4378268 | 4514039 | Exterior_Association |
| 17 | 5149731 | 33032559 | Health_QTL |
| 17 | 21371964 | 33032559 | Health_QTL |
| 18 | 8236580 | 25298314 | Meat_Quality_QTL |
| 18 | 13073783 | 13224946 | Meat_Quality_QTL |
| 18 | 24222671 | 25280809 | Meat_Quality_QTL |
| 18 | 24600578 | 24751740 | Reproduction_QTL |
| 18 | 24995988 | 25147151 | Meat_Quality_QTL |
| X | 2854344 | 3575005 | Meat_Quality_QTL |
| X | 2899062 | 2954960 | Meat_Quality_Association |
| X | 2899062 | 2954960 | Exterior_Association |
| X | 3049986 | 9074358 | Meat_Quality_QTL |
| X | 3155773 | 9074358 | Meat_Quality_QTL |
| X | 3155773 | 9074358 | Production_QTL |
| X | 3155773 | 12574847 | Reproduction_QTL |
| X | 3155773 | 16292037 | Production_QTL |
| X | 3155773 | 16292037 | Meat_Quality_QTL |
| X | 3155773 | 16292037 | Meat_Quality_QTL |
| X | 3155773 | 16292037 | Meat_Quality_QTL |
| X | 3155773 | 16292037 | Meat_Quality_QTL |
| X | 3155773 | 59081676 | Meat_Quality_QTL |
| X | 3155773 | 86652048 | Health_QTL |
| X | 3155773 | 86791792 | Exterior_QTL |
| X | 3155773 | 1.21E+08 | Meat_Quality_QTL |
| X | 3407312 | 3463210 | Meat_Quality_QTL |
| X | 4028194 | 4084091 | Meat_Quality_QTL |
| X | 8794870 | 8850768 | Meat_Quality_QTL |
| X | 9074358 | 16292037 | Meat_Quality_QTL |
| X | 9074358 | 16292037 | Production_QTL |
| X | 9074358 | 39265643 | Meat_Quality_QTL |
| X | 13972287 | 45675093 | Exterior_QTL |
| X | 14084082 | 38203589 | Health_QTL |
| X | 14838699 | 14894597 | Meat_Quality_QTL |
| X | 14866648 | 14922546 | Meat_Quality_QTL |
| X | 15788959 | 46038428 | Exterior_QTL |
| X | 16292037 | 42238776 | Production_QTL |
| X | 16292037 | 42238776 | Meat_Quality_QTL |
| X | 16292037 | 86763843 | Meat_Quality_QTL |
| X | 16292037 | 86763843 | Health_QTL |
| X | 16292037 | 1.26E+08 | Meat_Quality_QTL |
| X | 16515627 | 1.44E+08 | Meat_Quality_QTL |
| X | 39265643 | 42238776 | Meat_Quality_QTL |
| X | 42126981 | 86652048 | Meat_Quality_QTL |
| X | 42238776 | 86763843 | Meat_Quality_QTL |
| X | 42238776 | 86763843 | Meat_Quality_QTL |
| X | 42238776 | 1.44E+08 | Production_QTL |
| X | 59025778 | 59081676 | Meat_Quality_QTL |
| X | 60115782 | 86763843 | Meat_Quality_QTL |
| X | 86596150 | 86652048 | Meat_Quality_QTL |
| X | 86735894 | 86791792 | Exterior_QTL |
| X | 87770000 | 1.21E+08 | Meat_Quality_QTL |
| X | 1.18E+08 | 1.18E+08 | Meat_Quality_QTL |
| X | 1.18E+08 | 1.18E+08 | Meat_Quality_QTL |
| X | 1.18E+08 | 1.18E+08 | Meat_Quality_QTL |
| X | 1.18E+08 | 1.18E+08 | Exterior_QTL |
| X | 1.19E+08 | 1.26E+08 | Meat_Quality_QTL |
| X | 1.27E+08 | 1.44E+08 | Meat_Quality_QTL |
| X | 1.27E+08 | 1.44E+08 | Meat_Quality_QTL |

**Table S8:** Genome-wide significant SNPs associated with intramuscular fat (IMF)

| NO. | SNP | Chr.^1^ | Mapinfo | GWAS  *P*-value | NO. | SNP | Chr.^1^ | Mapinfo | GWAS  *P*-value |
| --- | --- | --- | --- | --- | --- | --- | --- | --- | --- |
| 1 | MARC0017000 | 12 | 60018478 | 2.82E-15 | 21 | M1GA0026465 | 12 | 67735786 | 8.63E-08 |
| 2 | ASGA0094812 | 12 | 58702424 | 3.69E-15 | 22 | ASGA0099873 | 12 | 28644797 | 9.53E-08 |
| 3 | ALGA0066945 | 12 | 26354444 | 1.27E-14 | 23 | DIAS0000861 | 12 | 27811612 | 1.21E-07 |
| 4 | ASGA0102838 | 0 | 0 | 1.92E-13 | 24 | ASGA0096690 | 12 | 28310456 | 1.48E-07 |
| 5 | M1GA0016908 | 12 | 25721207 | 1.99E-11 | 25 | ALGA0066986 | 12 | 26867891 | 1.80E-07 |
| 6 | ALGA0067189 | 12 | 28425124 | 7.90E-10 | 26 | MARC0004712 | 12 | 27642227 | 1.87E-07 |
| 7 | ALGA0109745 | 12 | 28798150 | 8.54E-10 | 27 | ALGA0119023 | 12 | 28970566 | 2.11E-07 |
| 8 | ALGA0107077 | 0 | 0 | 4.76E-09 | 28 | H3GA0022758 | 12 | 28966025 | 3.19E-07 |
| 9 | ASGA0055256 | 12 | 27929613 | 7.91E-09 | 29 | M1GA0017151 | 12 | 28387593 | 3.34E-07 |
| 10 | ALGA0067220 | 12 | 28545741 | 1.09E-08 | 30 | ALGA0067099 | 12 | 27582482 | 4.00E-07 |
| 11 | ASGA0089507 | 0 | 0 | 1.24E-08 | 31 | MARC0009546 | 12 | 65387966 | 4.27E-07 |
| 12 | ASGA0054854 | 12 | 23559238 | 2.73E-08 | 32 | M1GA0017055 | 12 | 27361619 | 4.38E-07 |
| 13 | ALGA0067173 | 12 | 28352940 | 2.99E-08 | 33 | MARC0030345 | 12 | 28468297 | 5.02E-07 |
| 14 | ALGA0108818 | 0 | 0 | 4.11E-08 | 34 | ASGA0055169 | 12 | 27175442 | 5.31E-07 |
| 15 | ASGA0085522 | 12 | 28080708 | 4.63E-08 | 35 | ALGA0067072 | 12 | 27419404 | 5.72E-07 |
| 16 | ALGA0107518 | 12 | 27942795 | 7.62E-08 | 36 | ASGA0055225 | 12 | 27555707 | 6.84E-07 |
| 17 | H3GA0056170 | 12 | 28081698 | 7.62E-08 | 37 | ALGA0067016 | 12 | 27066050 | 7.31E-07 |
| 18 | ASGA0084548 | 0 | 0 | 8.17E-08 | 38 | ALGA0117904 | 0 | 0 | 8.67E-07 |
| 19 | ASGA0093543 | 0 | 0 | 8.63E-08 | 39 | CASI0008458 | 12 | 3259334 | 9.09E-07 |
| 20 | M1GA0026329 | 12 | 67510304 | 8.63E-08 | 40 | ASGA0100525 | 12 | 27485992 | 1.03E-06 |

**Table S9:** Genome-wide significant SNPs associated with marbling

| NO. | SNP | Chr.^1^ | Mapinfo | GWAS  *P*-value | NO. | SNP | Chr.^1^ | Mapinfo | GWAS  *P*-value |
| --- | --- | --- | --- | --- | --- | --- | --- | --- | --- |
| 1 | ASGA0094812 | 12 | 58702424 | 2.70E-12 | 20 | ASGA0035681 | 7 | 1.08E+08 | 2.74E-08 |
| 2 | MARC0017000 | 12 | 60018478 | 7.00E-11 | 21 | M1GA0017151 | 12 | 28387593 | 2.79E-08 |
| 3 | ASGA0099873 | 12 | 28644797 | 5.53E-10 | 22 | ASGA0093543 | 0 | 0 | 3.96E-08 |
| 4 | ALGA0109745 | 12 | 28798150 | 5.54E-10 | 23 | M1GA0026329 | 12 | 67510304 | 3.96E-08 |
| 5 | ASGA0084548 | 0 | 0 | 6.99E-10 | 24 | M1GA0026465 | 12 | 67735786 | 3.96E-08 |
| 6 | ALGA0066945 | 12 | 26354444 | 2.72E-09 | 25 | ASGA0055256 | 12 | 27929613 | 4.41E-08 |
| 7 | ALGA0067072 | 12 | 27419404 | 3.84E-09 | 26 | ASGA0055225 | 12 | 27555707 | 4.49E-08 |
| 8 | MARC0004712 | 12 | 27642227 | 4.97E-09 | 27 | ALGA0067189 | 12 | 28425124 | 1.05E-07 |
| 9 | DIAS0000861 | 12 | 27811612 | 5.09E-09 | 28 | ASGA0100497 | 12 | 28854313 | 2.67E-07 |
| 10 | ALGA0067099 | 12 | 27582482 | 5.91E-09 | 29 | ASGA0054989 | 12 | 25327915 | 3.04E-07 |
| 11 | ASGA0100525 | 12 | 27485992 | 6.45E-09 | 30 | MARC0027759 | 12 | 27482373 | 3.13E-07 |
| 12 | MARC0009546 | 12 | 65387966 | 6.82E-09 | 31 | MARC0048623 | 0 | 0 | 4.26E-07 |
| 13 | ASGA0102838 | 0 | 0 | 7.00E-09 | 32 | ALGA0067220 | 12 | 28545741 | 5.03E-07 |
| 14 | ALGA0107518 | 12 | 27942795 | 7.37E-09 | 33 | ALGA0066986 | 12 | 26867891 | 7.54E-07 |
| 15 | H3GA0056170 | 12 | 28081698 | 7.37E-09 | 34 | ALGA0119023 | 12 | 28970566 | 8.40E-07 |
| 16 | MARC0030345 | 12 | 28468297 | 1.45E-08 | 35 | ALGA0066905 | 12 | 25833677 | 8.70E-07 |
| 17 | H3GA0022758 | 12 | 28966025 | 1.99E-08 | 36 | MARC0093869 | 0 | 0 | 9.42E-07 |
| 18 | ASGA0085522 | 12 | 28080708 | 2.49E-08 | 37 | MARC0051399 | 0 | 0 | 9.94E-07 |
| 19 | MARC0009817 | 0 | 0 | 2.60E-08 |  |  |  |  |  |

**Table S10:** Genome-wide significant SNPs associated with moisture

| NO. | SNP | Chr.^1^ | Mapinfo | GWAS  *P*-value | NO. | SNP | Chr.^1^ | Mapinfo | GWAS  *P*-value |
| --- | --- | --- | --- | --- | --- | --- | --- | --- | --- |
| 1 | ALGA0067173 | 12 | 28352940 | 4.37E-09 | 4 | ALGA0067189 | 12 | 28425124 | 7.69E-08 |
| 2 | ASGA0094812 | 12 | 58702424 | 8.02E-09 | 5 | ALGA0067220 | 12 | 28545741 | 1.00E-07 |
| 3 | MARC0017000 | 12 | 60018478 | 6.76E-08 | 6 | ALGA0066945 | 12 | 26354444 | 7.41E-07 |

**Table S11:** Genome-wide significant SNPs associated with color score

| NO. | SNP | Chr.^1^ | Mapinfo | GWAS  *P*-value | NO. | SNP | Chr.^1^ | Mapinfo | GWAS  *P*-value |
| --- | --- | --- | --- | --- | --- | --- | --- | --- | --- |
| 1 | ASGA0089507 | 0 | 0 | 1.68E-07 | 4 | ALGA0067072 | 12 | 27419404 | 4.34E-07 |
| 2 | M1GA0016964 | 12 | 26643372 | 2.83E-07 | 5 | ASGA0094812 | 12 | 58702424 | 5.94E-07 |
| 3 | ASGA0100525 | 12 | 27485992 | 4.08E-07 | 6 | MARC0093869 | 0 | 0 | 1.03E-06 |

**Table S12:** Genome-wide significant SNPs associated with lean meat in ham

| NO. | SNP | Chr.^1^ | Mapinfo | GWAS  *P*-value | NO. | SNP | Chr.^1^ | Mapinfo | GWAS  *P*-value |
| --- | --- | --- | --- | --- | --- | --- | --- | --- | --- |
| 1 | MARC0033927 | 2 | 1 | 2.61E-10 | 13 | H3GA0005551 | 2 | 356856 | 3.95E-07 |
| 2 | MARC0053324 | 2 | 3000 | 2.82E-09 | 14 | ALGA0103099 | 2 | 6000 | 3.98E-07 |
| 3 | M1GA0002246 | 2 | 602605 | 1.84E-08 | 15 | ALGA0116722 | 0 | 0 | 3.99E-07 |
| 4 | M1GA0027267 | 0 | 0 | 2.23E-08 | 16 | M1GA0002294 | 2 | 1007082 | 4.04E-07 |
| 5 | H3GA0005590 | 2 | 649713 | 2.39E-08 | 17 | H3GA0005627 | 2 | 1044957 | 4.76E-07 |
| 6 | MARC0113696 | 0 | 0 | 3.28E-08 | 18 | H3GA0005630 | 2 | 1069059 | 6.45E-07 |
| 7 | MARC0045154 | 2 | 10000 | 4.70E-08 | 19 | ASGA0008471 | 2 | 399335 | 6.94E-07 |
| 8 | ALGA0104369 | 0 | 0 | 5.62E-08 | 20 | ASGA0086466 | 0 | 0 | 7.98E-07 |
| 9 | ASGA0008534 | 2 | 933460 | 8.35E-08 | 21 | ASGA0102470 | 0 | 0 | 8.36E-07 |
| 10 | ASGA0089068 | 0 | 0 | 1.02E-07 | 22 | MARC0044928 | 0 | 0 | 8.39E-07 |
| 11 | MARC0071616 | 0 | 0 | 1.10E-07 | 23 | ASGA0008665 | 2 | 2387581 | 9.65E-07 |
| 12 | ASGA0008564 | 2 | 1194224 | 1.68E-07 |  |  |  |  |  |

**Table S13:** Genome-wide significant SNPs associated with lean meat weight

| NO. | SNP | Chr.^1^ | Mapinfo | GWAS  *P*-value | NO. | SNP | Chr.^1^ | Mapinfo | GWAS  *P*-value |
| --- | --- | --- | --- | --- | --- | --- | --- | --- | --- |
| 1 | ALGA0010777 | 1 | 241466579 | 6.05E-06 | 8 | M1GA0002246 | 2 | 602605 | 5.73E-06 |
| 2 | ALGA0010788 | 1 | 241600993 | 6.37E-06 | 9 | ALGA0104369 | 0 | 0 | 5.77E-06 |
| 3 | MARC0053324 | 2 | 3000 | 3.69E-07 | 10 | ALGA0103099 | 2 | 6000 | 5.91E-06 |
| 4 | ASGA0008705 | 2 | 2561671 | 3.77E-06 | 11 | MARC0045154 | 2 | 10000 | 6.07E-06 |
| 5 | H3GA0005590 | 2 | 649713 | 3.85E-06 | 12 | ASGA0008665 | 2 | 2387581 | 8.41E-06 |
| 6 | MARC0033927 | 2 | 1 | 4.68E-06 | 13 | ASGA0065455 | 14 | 100744736 | 7.41E-06 |
| 7 | MARC0071616 | 0 | 0 | 4.84E-06 | 14 | ASGA0065444 | 14 | 100323176 | 1.50E-05 |

**Table S14:** Genome-wide significant SNPs associated with mean corpuscular volume (MCV)

| SNP | Chr^1^ | Position^2^ | Closed Gene | distance（bp） | Pvalue |
| --- | --- | --- | --- | --- | --- |
| DRGA0008574 | 8 | 19407813 | *ENSSSCP00000009423* | 112244 | 7.62E-07 |
| ALGA0115258 | 8 | 35878420 | *KIT* | 51004 | 2.70E-07 |
| ALGA0047798 | 8 | 19593136 | *KIT* | 129777 | 5.37E-07 |
| MARC0053405 | 8 | 19787030 | *KDR* | within | 1.40E-07 |
| ALGA0047813 | 8 | 20029206 | *TLL1* | 6928 | 1.53E-07 |
| ASGA0038785 | 8 | 20153485 | *CPE* | 84772 | 1.25E-07 |
| MARC0039159 | 8 | 46447819 | *GUCY1A3* | 42366 | 8.66E-08 |
| MARC0075425 | 8 | 20831006 | *TDO* | within | 8.66E-08 |
| MARC0029724 | 8 | 47035822 | *GUCY1B3* | 270324 | 7.98E-08 |
| ALGA0047834 | 8 | 21038452 | *ENSSSCP00000009463* | 179502 | 4.03E-07 |
| ALGA0047829 | 8 | 20982241 | *ENSSSCP00000009463* | 117823 | 2.11E-07 |
| INRA0029810 | 8 | 21967480 | *ENSSSCP00000009465* | 266787 | 3.01E-07 |
| MARC0085941 | 8 | 49027286 | *ENSSSCP00000009465* | 240134 | 3.01E-07 |
| MARC0007151 | 8 | 22792312 | *LOC100517409* | 64593 | 2.68E-07 |
| ALGA0108841 | 8 | 50670176 | *LOC100517409* | within | 2.91E-07 |
| MARC0063673 | 8 | 23181660 | *ENSSSCP00000009476* | 148401 | 4.26E-07 |
| MARC0038980 | 8 | 23259080 | *LOC100517409* | 388568 | 3.40E-07 |
| ASGA0038810 | 8 | 23282033 | *ENSSSCP00000009476* | 223895 | 5.35E-07 |
| INRA0029816 | 8 | 23424013 | *ENSSSCP00000009476* | 365945 | 5.35E-07 |
| MARC0056888 | 8 | 23810304 | *ENSSSCP00000009476* | 753747 | 5.51E-07 |
| ALGA0047879 | 8 | 24068348 | *NONE* | >1Mb | 6.15E-07 |
| DBWU0000619 | 8 | 24166584 | *NONE* | >1Mb | 5.94E-07 |
| MARC0087739 | 8 | 24424725 | *ENSSSCP00000009478* | 635959 | 6.15E-07 |
| MARC0071439 | 8 | 24464495 | *ENSSSCP00000009478* | 39600 | 2.80E-07 |
| MARC0024662 | 8 | 24665960 | *O62706_1* | 310552 | 8.40E-07 |
| MARC0076384 | 8 | 24880142 | *O62706_1* | 45375 | 7.44E-07 |
| ALGA0047889 | 8 | 25038146 | *LOC100518673* | 32491 | 7.36E-07 |
| INRA0029827 | 8 | 25189969 | *ENSSSCP00000009481* | 22346 | 6.15E-07 |
| MARC0017124 | 8 | 55529571 | *LOC100517956* | 31601 | 6.15E-07 |
| ALGA0047893 | 8 | 25347613 | *ENSSSCP00000009483* | 29672 | 6.15E-07 |
| ALGA0047912 | 8 | 26164140 | *HOPX* | 40950 | 3.42E-07 |
| ALGA0047931 | 8 | 26664882 | *LOC100523263* | 81173 | 6.15E-07 |
| ASGA0100508 | 8 | 59002762 | *REST* | 13690 | 6.15E-07 |
| MARC0041089 | 8 | 59080961 | *IGFBP7* | within | 6.15E-07 |
| MARC0036671 | 8 | 27352645 | *IGFBP7* | 438883 | 6.15E-07 |
| ALGA0047954 | 8 | 27594895 | *IGFBP7* | 673868 | 6.15E-07 |
| MARC0020164 | 8 | 27781789 | *IGFBP7* | 962522 | 6.15E-07 |
| DRGA0008593 | 8 | 27911274 | *NONE* | >1Mb | 2.30E-07 |
| H3GA0024898 | 8 | 28216748 | *NONE* | >1Mb | 2.30E-07 |
| H3GA0024902 | 8 | 29230614 | *ENSSSCP00000009510* | 504438 | 2.30E-07 |
| H3GA0024903 | 8 | 29369327 | *ENSSSCP00000009510* | within | 2.30E-07 |
| MARC0051033 | 8 | 30893161 | *NONE* | >1Mb | 2.88E-07 |
| DRGA0008614 | 8 | 32164503 | *ENSSSCP00000009514* | 984471 | 5.75E-07 |
| MARC0005619 | 8 | 70772472 | *ENSSSCP00000009529* | 69642 | 5.55E-07 |
| MARC0070956 | 8 | 70907832 | *ENSSSCP00000009529* | 205002 | 5.55E-07 |
| INRA0029885 | 8 | 33295075 | *LOC100517174* | within | 6.35E-07 |
| ASGA0038909 | 8 | 33731819 | *ADAMTS3* | within | 3.80E-07 |
| ASGA0038910 | 8 | 33920942 | *ADAMTS3* | 112533 | 5.57E-07 |
| ASGA0038912 | 8 | 34044651 | *LOC100520317* | 77758 | 5.57E-07 |
| MARC0029095 | 8 | 73279054 | *LOC100520317* | 64527 | 7.58E-07 |
| ALGA0048087 | 8 | 34064884 | *LOC100520317* | 57525 | 5.57E-07 |
| DIAS0000495 | 8 | 34129053 | *LOC100520317* | within | 5.57E-07 |
| MARC0109837 | 8 | 73611944 | *ALB* | 903 | 5.57E-07 |
| ASGA0100173 | 8 | 73675216 | *ALB* | 45136 | 5.57E-07 |
| DIAS0002183 | 8 | 76813413 | *AFP* | within | 8.26E-07 |

**Table S15:** Genes in CNVR149

| Start | End | Gene Biotype | Gene Name | Description |
| --- | --- | --- | --- | --- |
| 19950609 | 19970663 | Protein cod | CASC3 | cancer susceptibility candidate 3 |
| 19975310 | 19988087 | Protein cod | RAPGEFL1 | Rap guanine nucleotide exchange factor (GEF)-like 1 |
| 20006236 | 20047678 | Protein cod | WIPF2 | WAS interacting protein family, member 2 |
| 20066410 | 20115048 | Protein cod | F1RXC4 | thyroid hormone receptor alpha |
| 20149317 | 20151340 | Protein cod | CSF3 | Granulocyte colony-stimulating factor |
| 20259192 | 20259269 | miRNA |  |  |
| 20287488 | 20287591 | snoRNA | SNORD124 | Small nucleolar RNA SNORD124 |
| 20429240 | 20521874 | Protein cod | IKZF3 | IKAROS family zinc finger 3 (Aiolos) |
| 20553936 | 20555633 | Protein cod | MIEN1 | migration and invasion enhancer 1 |
| 20579679 | 20587050 | Protein cod | PPP1R1B | protein phosphatase 1, regulatory (inhibitor) subunit 1B |
| 20587727 | 20613581 | Protein cod | STARD3 | stAR-related lipid transfer protein 3 |
| 20615599 | 20616848 | Protein cod | TCAP | telethonin |
| 20618539 | 20620121 | Protein cod | PNMT | Phenylethanolamine N-methyltransferase |
| 20672273 | 20673418 | pseudogene |  |  |
| 20802497 | 20902249 | Protein cod | MED1 | mediator complex subunit 1 |
| 21054363 | 21060781 | Protein cod | STAC2 | SH3 and cysteine rich domain 2 |
| 21078286 | 21099106 | Protein cod | CACNB1 | calcium channel, voltage-dependent, beta 1 subunit |
| 21113802 | 21174151 | Protein cod | PLXDC1 | plexin domain containing 1 |
| 21306343 | 21392895 | Protein cod | NPEPPS | aminopeptidase puromycin sensitive |
| 21418408 | 21445001 | Protein cod | KPNB1 | karyopherin (importin) beta 1 |
| 21457362 | 21472440 | Protein cod | TBKBP1 | TBK1 binding protein 1 |
| 21485201 | 21661479 | Protein cod | SP2 | Sp2 transcription factor |
| 21499519 | 21511736 | Protein cod | TBX21 | T-box 21 |
| 21593394 | 21599806 | Protein cod | LRRC46 | leucine rich repeat containing 46 |
| 21743207 | 21743292 | miRNA |  |  |
| 21743509 | 21754904 | Protein cod | COPZ2 | coatomer protein complex, subunit zeta 2 |
| 21831008 | 21883082 | Protein cod | SKAP1 | src kinase associated phosphoprotein 1 |
| 21912862 | 21939181 | Protein cod | CBX1 | chromobox homolog 1 |
| 22434918 | 22435019 | miRNA |  |  |
| 22603567 | 22633877 | Protein cod | CALCOCO2 | calcium binding and coiled-coil domain 2 |
| 22662246 | 22662479 | snoRNA | snoU89 | Small nucleolar RNA U89 |
| 22664940 | 22667646 | Protein cod | F1RWF6 | ATP synthase lipid-binding protein, mitochondrial |
| 22684979 | 22685108 | snoRNA | SNORA11 | Small nucleolar RNA SNORA11 |
| 22922300 | 22932267 | Protein cod | SPOP | speckle-type POZ protein |
| 22987595 | 23005905 | Protein cod | NGFR | nerve growth factor receptor |
| 23394660 | 23427719 | Protein cod | MYST2 | histone acetyltransferase MYST2 |
| 23813841 | 23829053 | Protein cod | LUC7L3 | LUC7-like 3 (S. cerevisiae) |
| 23879855 | 23884702 | Protein cod | WFIKKN2 | WAP, follistatin/kazal, immunoglobulin, kunitz and netrin domain containing2 |
| 23976973 | 23992345 | Protein cod | ANKRD40 | ankyrin repeat domain 40 |
| 24135232 | 24155023 | Protein cod | MYCBPAP | MYCBP associated protein |
| 24160771 | 24166981 | Protein cod | EPN3 | epsin 3 |
| 24171778 | 24181644 | Protein cod | SPATA20 | spermatogenesis-associated protein 20 |
| 24210110 | 24211162 | Protein cod | ACSF2 | acyl-CoA synthetase family member 2 |
| 24214235 | 24222582 | Protein cod | RSAD1 | radical S-adenosyl methionine domain containing 1 |
| 24292491 | 24307183 | Protein cod | LRRC59 | leucine rich repeat containing 59 |
| 24316379 | 24322636 | Protein cod | MRPL27 | mitochondrial ribosomal protein L27 |
| 24759158 | 24764661 | Protein cod | SAMD14 | sterile alpha motif domain containing 14 [Source:HGNC Symbol;Acc:27312] |
| 24858243 | 24858349 | snRNA | U6 | U6 spliceosomal RNA |
| 25059937 | 25089119 | Protein cod | NDKB_PIG | Nucleoside diphosphate kinase B |
| 25170730 | 25209079 | Protein cod | UTP18 | UTP18 small subunit (SSU) processome component homolog (yeast) |
| 28744823 | 28760307 | Protein cod | TOM1L1 | target of myb1 (chicken)-like 1 |
| 29242356 | 29269303 | Protein cod | MMD | monocyte to macrophage differentiation protein |
| 30352923 | 30616668 | Protein cod | ANKFN1 | ankyrin-repeat and fibronectin type III domain containing 1 |
| 30727119 | 30728003 | Protein cod | B8XVN4 | NogginUncharacterized protein |
| 30731742 | 30731860 | rRNA | 5S_rRNA | 5S ribosomal RNA |
| 30970298 | 30981372 | Protein cod | COIL | coilin |
| 31001929 | 31007597 | Protein cod | TRIM25 | tripartite motif containing 25 |
| 31010743 | 31036581 | Protein cod | DGKE | diacylglycerol kinase epsilon |
| 31150066 | 31150975 | pseudogene |  |  |
| 31313144 | 31548463 | Protein cod | MSI2 | musashi homolog 2 (Drosophila) |
| 31625448 | 31625557 | rRNA | 5S_rRNA | 5S ribosomal RNA |
| 31821607 | 31831017 | Protein cod | VEZF1 | vascular endothelial zinc finger 1 |
| 32016578 | 32018530 | Protein cod | SRSF1 | Serine/arginine-rich splicing factor 1 |
| 32308701 | 32313938 | Protein cod | DYNLL2 | dynein, light chain, LC8-type 2 |
| 32515991 | 32574585 | Protein cod | LPO | lactoperoxidase |
| 32981853 | 33014598 | Protein cod | RAD51C | RAD51 homolog C (S. cerevisiae) |
| 33049038 | 33049201 | snRNA | U1 | U1 spliceosomal RNA |
| 33058276 | 33058439 | snRNA | U1 | U1 spliceosomal RNA |
| 33059376 | 33059539 | snRNA | U1 | U1 spliceosomal RNA |
| 33062609 | 33062772 | snRNA | U1 | U1 spliceosomal RNA |
| 33068688 | 33068840 | snRNA | U1 | U1 spliceosomal RNA |
| 33069794 | 33069957 | snRNA | U1 | U1 spliceosomal RNA |
| 33081674 | 33081888 | snoRNA | U3 | Small nucleolar RNA U3 |
| 33085443 | 33085657 | snoRNA | U3 | Small nucleolar RNA U3 |
| 33090841 | 33091004 | snRNA | U1 | U1 spliceosomal RNA |
| 33272112 | 33295911 | Protein cod | PPM1E | protein phosphatase, Mg2+/Mn2+ dependent, 1E |
| 33527929 | 33528035 | snRNA | U6 | U6 spliceosomal RNA |
| 33579056 | 33580010 | pseudogene |  |  |
| 33917589 | 33963642 | Protein cod | DHX40 | DEAH (Asp-Glu-Ala-His) box polypeptide 40 |
| 33985185 | 34054684 | Protein cod | C0MHR2 | clathrin heavy chain 1 |
| 34069469 | 34200586 | Protein cod | VMP1 | vacuole membrane protein 1 |
| 34201565 | 34201656 | miRNA |  | ssc-mir-21 |
| 35277764 | 35322510 | Protein cod | BCAS3 | breast carcinoma amplified sequence 3 |
| 35623636 | 35683948 | Protein cod | APPBP2 | amyloid beta precursor protein (cytoplasmic tail) binding protein 2 |
| 35660135 | 35660258 | snoRNA | SNORA17 | Small nucleolar RNA SNORA17 |
| 35966771 | 35966898 | snoRNA | SNORA11 | Small nucleolar RNA SNORA11 |
| 36018428 | 36098860 | Protein cod | F1S201 | Ubiquitin carboxyl-terminal hydrolase |
| 36181764 | 36247051 | Protein cod | GGNBP2 | gametogenetin binding protein 2 |
| 36214783 | 36214910 | snoRNA | SNORA11 | Small nucleolar RNA SNORA11 |
| 36259817 | 36265133 | Protein cod | MRM1 | mitochondrial rRNA methyltransferase 1 homolog (S. cerevisiae) |
| 36781687 | 36883430 | Protein cod | AATF | apoptosis antagonizing transcription factor |
| 36928552 | 36928658 | snRNA | U6 | U6 spliceosomal RNA |

**Table S16:** Genes in CNVR31

| Start | End | Gene Biotype | Gene Name | Description |
| --- | --- | --- | --- | --- |
| 162398 | 167169 | Protein cod | PIDD | p53-induced death domain protein |
| 176014 | 178780 | Protein cod | CEND_PIG | Cell cycle exit and neuronal differentiation protein 1 |
| 185919 | 191739 | Protein cod | PDDC1 | Parkinson disease 7 domain containing 1 |
| 238775 | 261809 | Protein cod | DEAF1 | deformed epidermal autoregulatory factor 1 (Drosophila) |
| 282142 | 290232 | Protein cod | SECR_PIG | Secretin |
| 291676 | 294411 | Protein cod | A0ZVR0 | interferon regulatory factor 7 |
| 331299 | 331396 | miRNA |  | ssc-mir-210 |
| 564821 | 565855 | pseudogene |  |  |
| 1273564 | 1283402 | Protein cod | SHANK2 | SH3 and multiple ankyrin repeat domains 2 |
| 1354813 | 1355118 | Protein cod | Q56VC2 | protein FADD |
| 1686278 | 1688039 | Protein cod | FGF4 | fibroblast growth factor 4 |
| 1742745 | 1746468 | Protein cod | FGF19 | fibroblast growth factor 19 |
| 1757505 | 1765881 | Protein cod | FGF3 | fibroblast growth factor 3 |
| 1833419 | 1840936 | Protein cod | ORAOV1 | oral cancer overexpressed 1 |
| 2298255 | 2314866 | Protein cod | TPCN2 | two pore segment channel 2 |
| 2397331 | 2398278 | Protein cod | MRGPRD | MAS-related GPR, member D |
| 2436853 | 2448144 | Protein cod | MRPL21 | mitochondrial ribosomal protein L21 |
| 2540801 | 2641517 | Protein cod | MTL5 | metallothionein-like 5, testis-specific (tesmin) |
| 2964562 | 2974104 | Protein cod | C11orf24 | chromosome 11 open reading frame 24 |
| 3012437 | 3110208 | Protein cod | SUV420H1 | suppressor of variegation 4-20 homolog 1 (Drosophila) |
| 3133038 | 3293093 | Protein cod | TBX10 | T-box 10 |
| 3133576 | 3190746 | Protein cod | CHKA | choline kinase alpha |
| 3294498 | 3309019 | Protein cod | NUDT8 | nudix (nucleoside diphosphate linked moiety X)-type motif 8 |
| 3331731 | 3350753 | Protein cod | F1RVM9 | Aldehyde dehydrogenase |
| 3406772 | 3408962 | Protein cod | CDK2AP2 | cyclin-dependent kinase 2 associated protein 2 |
| 3411264 | 3423542 | Protein cod | PITPNM1 | phosphatidylinositol transfer protein, membrane-associated 1 |
| 3438571 | 3443453 | Protein cod | TMEM134 | transmembrane protein 134 |
| 3456129 | 3456956 | Protein cod | GPR152 | G protein-coupled receptor 152 |
| 3468942 | 3469568 | Protein cod | PTPRCAP | protein tyrosine phosphatase, receptor type, C-associated protein |
| 3501365 | 3504471 | Protein cod | F1RUX8 | protein phosphatase 1 catalytic subunit alpha isoform |
| 3562263 | 3564074 | Protein cod | POLD4 | polymerase (DNA-directed), delta 4 |
| 3943363 | 3948616 | Protein cod | PC | pyruvate carboxylase, mitochondrial |
| 4043508 | 4051227 | Protein cod | SPTBN2 | spectrin, beta, non-erythrocytic 2 |
| 4121849 | 4163669 | Protein cod | CCS | copper chaperone for superoxide dismutase |
| 4282080 | 4286831 | Protein cod | NPAS4 | neuronal PAS domain protein 4 |
| 4318313 | 4327076 | Protein cod | PELI3 | pellino homolog 3 (Drosophila) |
| 4331535 | 4371006 | Protein cod | DPP3 | dipeptidyl-peptidase 3 |
| 4432363 | 4436964 | Protein cod | RIN1 | Ras and Rab interactor 1 |
| 4475173 | 4479363 | Protein cod | YIF1A | Yip1 interacting factor homolog A (S. cerevisiae) |
| 4686131 | 4694266 | Protein cod | CATSPER1 | cation channel, sperm associated 1 |
| 4707899 | 4710966 | Protein cod | EIF1AD | eukaryotic translation initiation factor 1A domain containing |
| 4790094 | 4790849 | Protein cod | C11orf68 | chromosome 11 open reading frame 68 |
| 4817859 | 4823304 | Protein cod | FIBP | fibroblast growth factor (acidic) intracellular binding protein |
| 4832868 | 4865376 | Protein cod | EFEMP2 | EGF containing fibulin-like extracellular matrix protein 2 |
| 4873775 | 4876747 | Protein cod | COFILIN | cofilin-1 |
| 4961454 | 4963251 | Protein cod | RNASEH2C | ribonuclease H2, subunit C |
| 5053264 | 5066070 | Protein cod | SCYL1 | SCY1-like 1 (S. cerevisiae) |
| 5450772 | 5450875 | snRNA | U6 | U6 spliceosomal RNA |
| 5471274 | 5473282 | Protein cod | RS30_PIG | 40S ribosomal protein S30 |
| 5475869 | 5477068 | Protein cod | ZNHIT2 | zinc finger, HIT-type containing 2 |
| 5504232 | 5509330 | Protein cod | CDCA5 | cell division cycle associated 5 |
| 5528118 | 5538804 | Protein cod | NAALADL1 | N-acetylated alpha-linked acidic dipeptidase-like 1 |
| 5621411 | 5642123 | Protein cod | C11orf85 | chromosome 11 open reading frame 85 |
| 5646978 | 5647633 | Protein cod | GPHA2 | glycoprotein hormone alpha 2 |
| 5685208 | 5685291 | miRNA |  |  |
| 5685399 | 5685508 | miRNA |  |  |
| 5699171 | 5726031 | Protein cod | EHD1 | EH-domain containing 1 |
| 5734333 | 5752406 | Protein cod | CDC42BPG | CDC42 binding protein kinase gamma (DMPK-like) |
| 5762005 | 5765616 | Protein cod | MEN1 | multiple endocrine neoplasia I |
| 5767117 | 5778518 | Protein cod | MAP4K2 | mitogen-activated protein kinase kinase kinase 2 |
| 5792161 | 5805583 | Protein cod | SF1 | splicing factor 1 |
| 5810420 | 5821868 | Protein cod | F1RQQ7 | Phosphorylase |
| 5823661 | 5839410 | Protein cod | RASGRP2 | RAS guanyl releasing protein 2 (calcium and DAG-regulated) |
| 5852514 | 5951864 | Protein cod | NRXN2 | neurexin 2 |
| 6156279 | 6159332 | Protein cod | C11orf20 | chromosome 11 open reading frame 20 |
| 6161598 | 6168487 | Protein cod | F1RQP2 | steroid hormone receptor ERR1 |
| 6170436 | 6173823 | Protein cod | PRDX5 | peroxiredoxin-5, mitochondrial |

**Table S17:** Primers and probes used in QPCR validation

| No. | CNVR No. | gene | +5’………………3’+ |
| --- | --- | --- | --- |
| 1 | CNVR42 | C1ORF150 | F tggcagtggatctgaagaagtg  R gaagggcatattctgtttctgacc  Probe FAM-ccctatcggagaccctccctgagct-BHQ |
| 2 | CNVR79 | CES1 | F cgatgtacgaccaggaagaagg  R ggacaggagatcgttccagaag  Probe FAM-tcggcgtcaacacccaggca-BHQ |
| 3 | CNVR184 | CHR14 | F gcgtagcgggaccgatt  R ggaacgtggtcggaggtaag  Probe FAM -cggcaagcccggagctgc- BHQ |
| 4 | CNVR243 | CHR18 | F atcctgactgaatgcccctatc  R atggtgtcagaaatctaccctgg  Probe FAM -cagccctcctcgtactgcactgatga- BHQ |
| 5 | CNVR86 | ECI2 | F tccaagaaggcgaagctatgac  R gcacgatgacgcctctttg  Probe FAM -acagcccaggacttcccagcagc- BHQ |
| 6 | CNVR16 | ELL2 | F atccccaagccccgaag  R caatatcattaccccccaaagc  Probe FAM -acctcttgtgagtgccctgccatttc- BHQ |
| 7 | CNVR64 | FISGK0 | F tgatgcgggagtgctgg  R gggaagttcaggcatcggt  Probe FAM -cgctgcggatcaagaagacgctaca- BHQ |
| 8 | CNVR67 | HMGA2 | F cagacactcttgccacaacagc  R tggagggactcttgtttttgct  Probe FAM -tttcccctgaaactaggagccaaccg- BHQ |
| 9 | CNVR167 | RETRO | F gtgtctgcctccttcctgagtag  R ggtctggaactcccgcct  Probe FAM -cagaaatcccatctgaacagcctccct- BHQ |
| 10 | CNVR3 | TIAM2 | F gaactgaagctcgatgtctttgg  R cggacctctctgacagcctc  Probe FAM - cgtcgtcgctcaggatgtcgctct- BHQ |
| 11 | CONTROL | GCG | F gcaatatggctttagaatacacctctta  R gtcactaatcaagatcgtgttcacaac  Probe FAM- tcaaacatcccacatggctggcag-BHQ |
