# Supplementary material for: Genome-Wide Copy Number Variations Inferred from SNP Genotyping Arrays Using a Large White and Minzhu Intercross Population
Source: PLoS One. 2013 Oct 1;8(10):e74879. doi: 10.1371/journal.pone.0074879 (PMC3787955; doi:10.1371/journal.pone.0074879)
Supplement: File S2 — Additional Figures: Figure S1: Distribution of CNVRs in Minzhu pig F0 generation. Figure S2: Distribution of CNVRs in Large White pig F0 generation. Figure S3: Relative quantification (RQ) value by Quantitative PCR (QPCR) for CNVR3. Figure S4: Relative quantification (RQ) value by Quantitative PCR (QPCR) for CNVR16. Figure S5: Relative quantification (RQ) value by Quantitative PCR (QPCR) for CNVR42. Figure S6: Relative quantification (RQ) value by Quantitative PCR (QPCR) for CNVR67. Figure S7: Relative quantification (RQ) value by Quantitative PCR (QPCR) for CNVR86. Figure S8: Relative quantification (RQ) value by Quantitative PCR (QPCR) for CNVR167. Figure S9: Relative quantification (RQ) value by Quantitative PCR (QPCR) for CNVR184. Figure S10: Relative quantification (RQ) value by Quantitative PCR (QPCR) for CNVR243. (DOCX) [file pone.0074879.s002.docx]

**
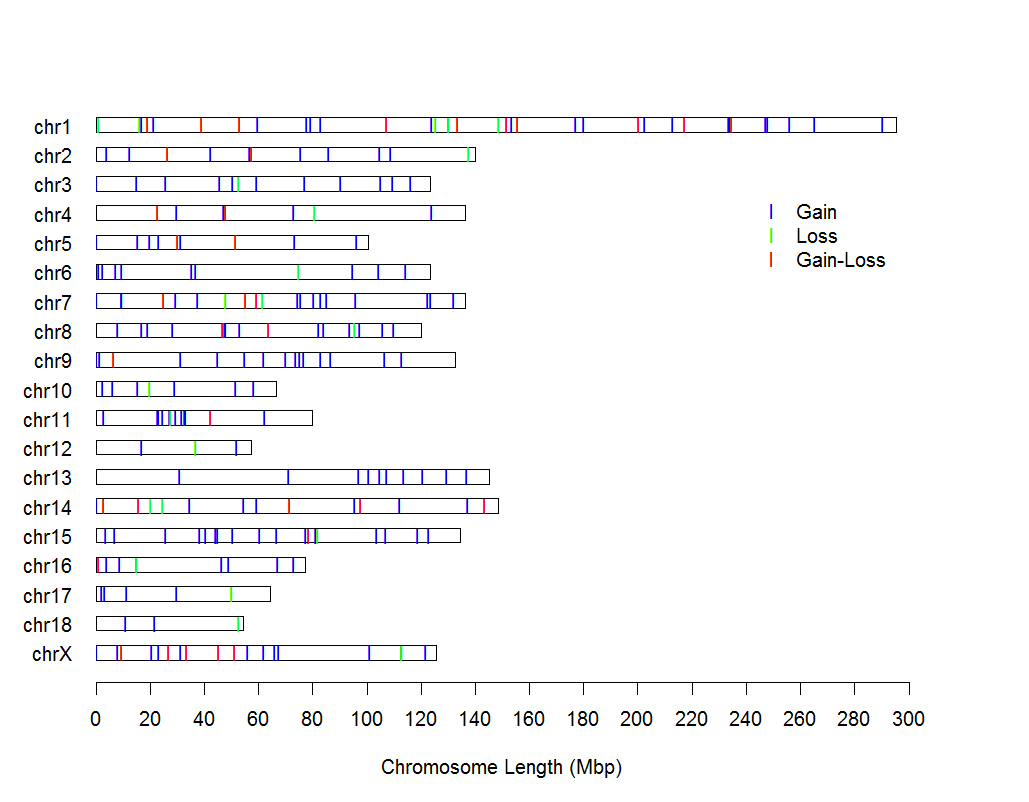
**

**Figure S1:** Distribution of CNVRs in Minzhu pig F0 generation

Red, green and blue lines represent Gain, loss and either gain or loss predicted status. Y-axis values are chromosome names, and X-axis values are chromosome position in Mb.

**
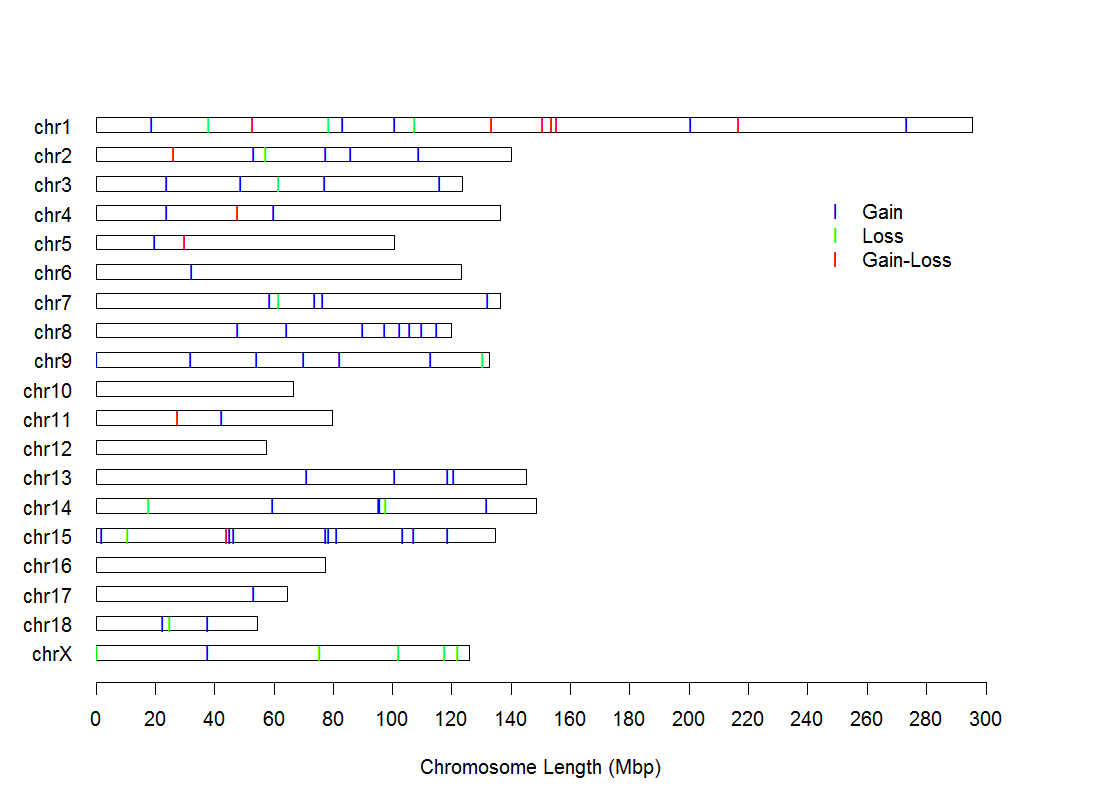
**

**Figure S2:** Distribution of CNVRs in Large White pig F0 generation

Red, green and blue lines represent Gain, loss and either gain or loss predicted status. Y-axis values are chromosome names, and X-axis values are chromosome position in Mb.

**

**

**Figure S3:** Relative quantification (RQ) value by Quantitative PCR (QPCR) for CNVR3.

Twenty animals with Relative quantification (RQ) value are showed in this figure. Each dot represents the relative copy number in comparison to the reference individual. Y-axis shows the RQ obtained by QPCR. Samples with RQ about 1 denote normal individuals (two copy), samples with RQ below 0.59 (ln^1.5^) denote copy number loss individuals, and samples with RQ about 1.59 (ln^3^) or more denote copy number gain individuals (≧three copy).

**

**

**Figure S4:** Relative quantification (RQ) value by Quantitative PCR (QPCR) for CNVR16.

Twenty animals with Relative quantification (RQ) value are showed in this figure. Each dot represents the relative copy number in comparison to the reference individual. Y-axis shows the RQ obtained by QPCR. Samples with RQ about 1 denote normal individuals (two copy), samples with RQ below 0.59 (ln^1.5^) denote copy number loss individuals, and samples with RQ about 1.59 (ln^3^) or more denote copy number gain individuals (≧three copy).

**

**

**Figure S5:** Relative quantification (RQ) value by Quantitative PCR (QPCR) for CNVR42.

Twenty animals with Relative quantification (RQ) value are showed in this figure. Each dot represents the relative copy number in comparison to the reference individual. Y-axis shows the RQ obtained by QPCR. Samples with RQ about 1 denote normal individuals (two copy), and samples with RQ about 1.59 (ln^3^) or more denote copy number gain individuals (≧three copy).

**

**

**Figure S6:** Relative quantification (RQ) value by Quantitative PCR (QPCR) for CNVR67.

Twenty animals with Relative quantification (RQ) value are showed in this figure. Each dot represents the relative copy number in comparison to the reference individual. Y-axis shows the RQ obtained by QPCR. Samples with RQ about 1 denote normal individuals (two copy), samples with RQ below 0.59 (ln^1.5^) denote copy number loss individuals, and samples with RQ about 1.59 (ln^3^) or more denote copy number gain individuals (≧three copy).

**

**

**Figure S7:** Relative quantification (RQ) value by Quantitative PCR (QPCR) for CNVR86.

Twenty animals with Relative quantification (RQ) value are showed in this figure. Each dot represents the relative copy number in comparison to the reference individual. Y-axis shows the RQ obtained by QPCR. Samples with RQ about 1 denote normal individuals (two copy), samples with RQ below 0.59 (ln^1.5^) denote copy number loss individuals, and samples with RQ about 1.59 (ln^3^) or more denote copy number gain individuals (≧three copy).

**

**

**Figure S8:** Relative quantification (RQ) value by Quantitative PCR (QPCR) for CNVR167.

Twenty animals with Relative quantification (RQ) value are showed in this figure. Each dot represents the relative copy number in comparison to the reference individual. Y-axis shows the RQ obtained by QPCR. Samples with RQ about 1 denote normal individuals (two copy), samples with RQ below 0.59 (ln^1.5^) denote copy number loss individuals.

**

**

**Figure S9:** Relative quantification (RQ) value by Quantitative PCR (QPCR) for CNVR184.

Twenty animals with Relative quantification (RQ) value are showed in this figure. Each dot represents the relative copy number in comparison to the reference individual. Y-axis shows the RQ obtained by QPCR. Samples with RQ about 1 denote normal individuals (two copy).

**

**

**Figure S10:** Relative quantification (RQ) value by Quantitative PCR (QPCR) for CNVR243.

Twenty animals with Relative quantification (RQ) value are showed in this figure. Each dot represents the relative copy number in comparison to the reference individual. Y-axis shows the RQ obtained by QPCR. Samples with RQ about 1 denote normal individuals (two copy), samples with RQ below 0.59 (ln^1.5^) denote copy number loss individuals, and samples with RQ about 1.59 (ln^3^) or more denote copy number gain individuals (≧three copy).
